# Synthesis, Antimicrobial and Antibiofilm Activities, and Molecular Docking Investigations of 2-(1*H*-Indol-3-yl)-1*H*-benzo[*d*]imidazole Derivatives

**DOI:** 10.3390/molecules28207095

**Published:** 2023-10-14

**Authors:** Elena Y. Mendogralo, Larisa Y. Nesterova, Ekaterina R. Nasibullina, Roman O. Shcherbakov, Danil A. Myasnikov, Alexander G. Tkachenko, Roman Y. Sidorov, Maxim G. Uchuskin

**Affiliations:** 1 Department of Chemistry, Perm State University, Bukireva St. 15, 614990 Perm, Russia; kat.nasibullina@yandex.ru (E.R.N.); romanshcherbakov00@gmail.com (R.O.S.); mda@psu.ru (D.A.M.); sidorov.r@iegm.ru (R.Y.S.); mu@psu.ru (M.G.U.); 2 Department of Biology, Perm State University, Bukireva St. 15, 614990 Perm, Russia; larisa.nesterova@bk.ru (L.Y.N.); agtkachenko@iegm.ru (A.G.T.); 3 Institute of Ecology and Genetics of Microorganisms, Perm Federal Research Center, The Ural Branch of Russian Academy of Sciences, Goleva St. 13, 614081 Perm, Russia

**Keywords:** azaheterocycle, indole, antibacterial activity, resistance, molecular docking

## Abstract

The treatment of many bacterial and fungal infections remains a problem due to increasing antibiotic resistance and biofilm formation by pathogens. In the present article, a methodology for the chemoselective synthesis of 2-(1*H*-indol-3-yl)-1*H*-benzo[*d*]imidazole derivatives is presented. We report on the antimicrobial activity of synthesized 2-(1*H*-indol-3-yl)-1*H*-benzo[*d*]imidazoles with significant activity against *Staphylococcus aureus* ATCC 25923, *Staphylococcus aureus* ATCC 43300 (MRSA), *Mycobacterium smegmatis* (mc(2)155/ATCC 700084), and *Candida albicans* ATCC 10231. High activity against staphylococci was shown by indolylbenzo[*d*]imidazoles **3ao** and **3aq** (minimum inhibitory concentration (MIC) < 1 µg/mL) and **3aa** and **3ad** (MIC 3.9–7.8 µg/mL). A low MIC was demonstrated by 2-(1*H*-indol-3-yl)-1-methyl-1*H*-benzo[*d*]imidazole (**3ag**) against *M. smegmatis* and against *C. albicans* (3.9 µg/mL and 3.9 µg/mL, respectively). 2-(5-Bromo-1*H*-indol-3-yl)-6,7-dimethyl-1*H*-benzo[*d*]imidazole (**3aq**) showed a low MIC of 3.9 µg/mL against *C. albicans*. Compounds **3aa**, **3ad**, **3ao**, and **3aq** exhibited excellent antibiofilm activity, inhibiting biofilm formation and killing cells in mature biofilms. Molecular docking analysis identified three potential interaction models for the investigated compounds, implicating (p)ppGpp synthetases/hydrolases, FtsZ proteins, or pyruvate kinases in their antibacterial action mechanism.

## 1. Introduction

The global health system has worked hard to protect and promote human health. However, the world continues to face newly emerging infectious disease threats. The lack of effective antimicrobial drugs for the prevention and treatment of infections complicates many medical procedures, including cancer and diabetes therapy and organ transplantation. The biggest problem in the fight against infectious diseases is the rapid formation of resistance to existing drugs [1,2]. Many infections remain critical due to the increasing antibiotic resistance of pathogens belonging to different groups of microorganisms. One of the most important infectious agents is *Staphylococcus aureus*. This Gram-positive bacterium is a widespread pathogen that can colonize many biotopes in the human and animal body, causing various diseases. *S. aureus* shows increasing antibiotic resistance to current antibiotics, especially β-lactams [3,4]. Insensitivity to many antibiotics is also widespread among a special group of microorganisms—mycobacteria. These bacteria have a special cell wall structure, which has low permeability to various substances, including antibacterial agents. Mycobacteria cause a number of difficult-to-treat diseases, primarily tuberculosis, which has long been one of the leading causes of death [5]. It should also be noted that fungal infections, including candidiasis, are quite widespread at present. *Candida albicans* is the most implicated fungal species, and grows as an opportunistic pathogen in the human host. It is associated with many life-threatening infections, especially in immunocompromised persons, and can cause sepsis [6].

An important feature of microorganisms is their ability to form biofilms, which is another challenge associated with the treatment of infectious diseases. Biofilms are multicellular microbial assemblages surrounded by a matrix composed of polysaccharides containing extracellular DNA, proteins, and lipids. Biofilms allow bacteria to grow on medical devices, leading to chronic infections. Cells in the biofilm have reduced sensitivity to antibiotics and effectors of the host immune system. Antibiotics that show antibiofilm activity, especially against *S. aureus*, are limited. This is a very important task—the search for agents that can effectively destroy microorganisms resistant to traditional antibiotics, as well as cells in biofilms.

**Figure 1:**
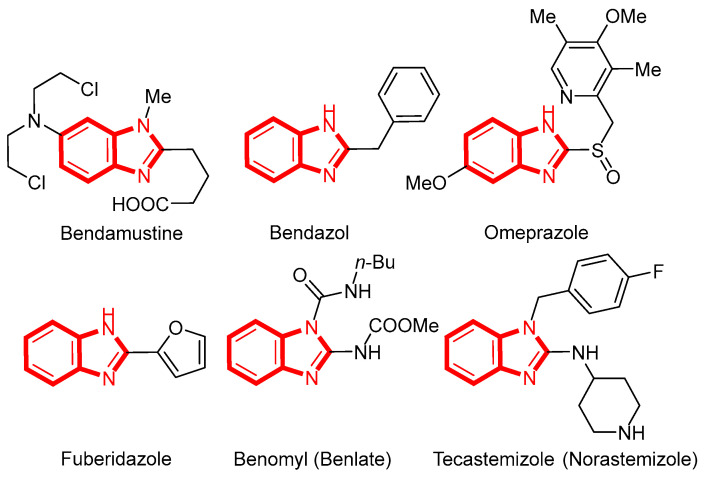
Drug molecules that have a benzimidazole pharmacophore.

Benzimidazole is an important pharmacophore fragment in the drug discovery process since it exhibits various therapeutic properties [7,8]. For example, bendamustine (Treanda) is a chemotherapy medication used in the treatment of chronic lymphocytic leukemia (Figure 1) [9]. Bendazol (Dibazol) demonstrates immunostimulating as well as vasodilating and antispasmodic effects [10]. Omeprazole is a drug that inhibits gastric acid secretion and is used in the treatment of gastric ulcers [11]. Fuberidazole and Benomyl (Benlate) are the active ingredients of fungicides [12,13]. Tecastemizole (Norastemizole) shows anti-inflammatory activity [14]. It is worth noting that indole derivatives exhibit a wide range of biological activities [15]: antiproliferative, antioxidant [16], antiviral [17], antibacterial, and antifungal [18]. The combination of a benzimidazole moiety with an additional azaheterocycle in a single molecule may promise a synergistic enhancement of therapeutic effect.

Derivatives of benzimidazole exhibit diverse activities by interacting with numerous biomolecular targets. It is known that 2,5,6-trisubstituted benzimidazoles demonstrate antitubercular activity by targeting filamenting temperature-sensitive protein Z (FtsZ) [19]. This protein plays a crucial role in bacterial cell division, making it a promising target for the development of antibacterial agents against various bacterial pathogens. Moreover, compounds containing a benzimidazole fragment have been identified as inhibitors of essential pyruvate kinase enzymes, exhibiting antistaphylococcal activity [20,21]. Molecular modelling and thermal shift assays have demonstrated that substituted indoles and benzimidazoles can bind to Rel_Seq_ (p)ppGpp synthetase/hydrolase. This protein of the RSH superfamily participates in bacterial non-essential regulatory pathways carrying out (p)ppGpp synthesis and hydrolysis. Increased concentrations of intracellular (p)ppGpp alarmones have been linked to the emergence of bacterial persistence, tolerance, and resistance, while the inability of bacteria to generate these alarmones leads to the suppression of these processes [22]. Alarmone synthetase inhibitors, which specifically target this protein, have been found to exhibit activity against bacterial cells experiencing nutrient deprivation [23,24]. Therefore, the study of the biological properties of novel benzimidazole derivatives has been intensified in recent years.

In continuation of our endeavors toward the development of effective antibacterial agents, in this paper we report on the application of our previously developed direct approach [25] to 2-(1*H*-indol-3-yl)-1*H*-benzo[*d*]imidazole and its analogs. Moreover, the prepared compounds were tested for antibacterial activity, including tests against *Escherichia coli*; *S. aureus*, including MRSA; *Mycobacterium smegmatis*; and *C. albicans*, as well as their influence on the formation and survival of biofilms. We describe the results of molecular docking studies on the ability of substituted benzimidazoles to bind to potential targets: (p)ppGpp synthetases/hydrolases, FtsZ proteins, and pyruvate kinases from *E. coli*, *S. aureus*, *M. smegmatis*, and *C. albicans*.

## 2. Results and Discussion

### 2.1. Chemistry

**Scheme 1:**
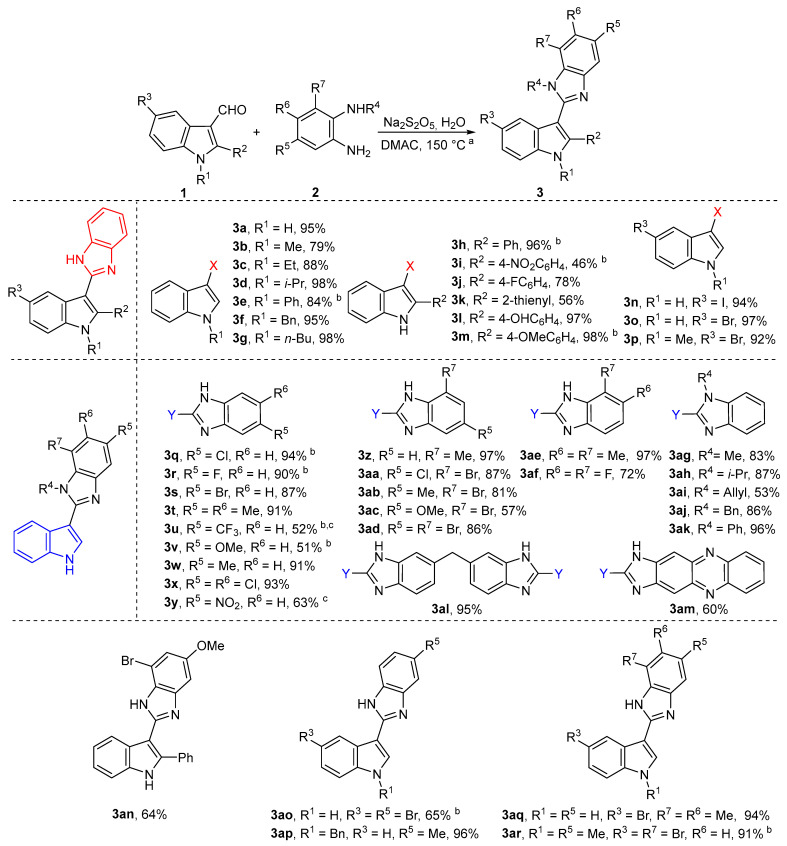
The synthesis of substituted benzimidazoles **3**. ^a^ All reactions were performed at 1.3 mmol scale of indole-3-carboxaldehyde **1** and 1.3 mmol of *o*-phenylenediamine **2**. Isolated yields. ^b^ The product was isolated as a solvate with DMAC. ^c^ Mixture of isomers.

In continuation of our previous work on the development of an effective strategy for the synthesis of antibacterial compounds [25], we have become interested in finding the chemoselective pathway of 2-(1*H*-indol-3-yl)-1*H*-benzo[*d*]imidazole derivatives [26]. We have previously shown that the condensation of anthranilamides with aldehydes on heating in *N*,*N*-dimethylacetamide (DMAC) in the presence of sodium metabisulfite leads to the formation of antibacterial 2-(1*H*-indol-3-yl)quinazolin-4(3*H*)-ones and their analogs. In the present study, we have expanded the scope of this reaction to obtain 2-(1*H*-indol-3-yl)-1*H*-benzo[*d*]imidazole derivatives **3** (Scheme 1). As shown in Scheme 1, we investigated the substrate scope of substituted indole-3-carboxaldehyde **1** starting with varying the substituents on the nitrogen atom of the indole ring. Aliphatic substituents, benzyl, and phenyl were well tolerated under this protocol and resulted in the corresponding products **3b**-**g** in 79–98% yields. Target compounds **3h**-**m** containing a substituent at the C(2) atom of the indole moiety have been obtained in good yields. A slightly reduced yield was observed for functionalized indoles that had *para*-nitrophenyl and thienyl groups (**3i**, **3k**). 5-Substituted indoles **1n**-**p** were converted to the corresponding benzimidazoles **3n**-**p** in excellent isolated yields (92–97%). Next, the scope of the protocol was investigated using substituted phenylenediamines. The reaction was well suited for diamines containing halogens **2b**-**d**, **i**, **l**, **o**, **q**; methyl **2e**, **h**, **k**, **m**, **p**; and for *N*-substituted starting substrates **2r**-**v**. Target products **3q**-**t**, **w**, **x**, **z**-**ab**, **ad**-**ah**, **aj**, and **3ak** were isolated as the sole product in good to excellent yields (72–97%). Surprisingly, the CF_3_- and NO_2_-substituted 2-(1*H*-indol-3-yl)-1*H*-benzo[*d*]imidazoles **3u** and **3y** were formed as mixtures of two isomers with a total yield of 52% and 63%, respectively (Scheme 1). The observed reactivity of unsymmetrical phenylenediamines is described in the literature [27,28]. In the case of a methoxy group in the benzene ring and an allyl group at the nitrogen atom, we observed the formation of benzimidazoles **3v**, **ac**, and **3ai** with a slightly decreased yield (51%, 57%, and 53%), together with a mixture of unidentified by-products. The method proved to be applicable to compounds of more challenging architecture such as 4,4’-methylenedibenzene-1,2-diamine (**2w**) and phenazine-2,3-diamine (**2x**). As a result of the cyclocondensation reaction of tetramine **2w** with 1*H*-indole-3-carboxaldehyde (**1a**), the bis-derivative **3al** was obtained in an excellent yield of 95%. The phenazine-2,3-diamine (**2x**) offered corresponding imidazole **3am** in a 60% yield. This choice was not accidental, since phenazine derivatives are distinguished by strong bacteriostatic properties [29]. The scope and limitations were investigated with respect to the synthesis of a variety of polysubstituted benzimidazoles. The target compounds **3an**-**3ar** were obtained in 64–96% yields.

**Scheme 2:**
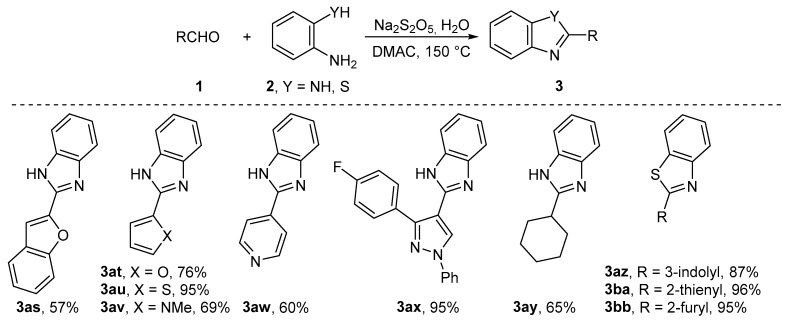
The synthesis of substituted benzimidazoles **3as**-**ay** and benzothiazoles **3az**-**bb**.

The limits of the applicability of the present protocol were expanded to various heterocyclic aldehydes **1** as starting substrates. As shown in Scheme 2, a wide range of heterocyclic aldehydes **1**, such as benzofuran-2-carbaldehyde, furfural, thiophene-2-carbaldehyde, 1-methyl-1*H*-pyrrole-2-carbaldehyde, isonicotinaldehyde, and pyrazolecarbaldehyde, gave good yields of respective products. The cyclohexanecarbaldehyde **1w** provided the product **3ay** with a yield of 65%. 2-Aminothiophenol (**2y**) reacted with 1*H*-indole-3-carboxaldehyde (**1a**) with the formation of benzo[*d*]thiazole **3az** in an 87% yield. Similarly, through the reaction of 2-aminothiophenol (**2y**) and heterocyclic aldehydes **1r**, **s** occurred, yielding benzo[d]thiazoles **3ba** and **3bb** in 95–96% yields.

**Scheme 3:**
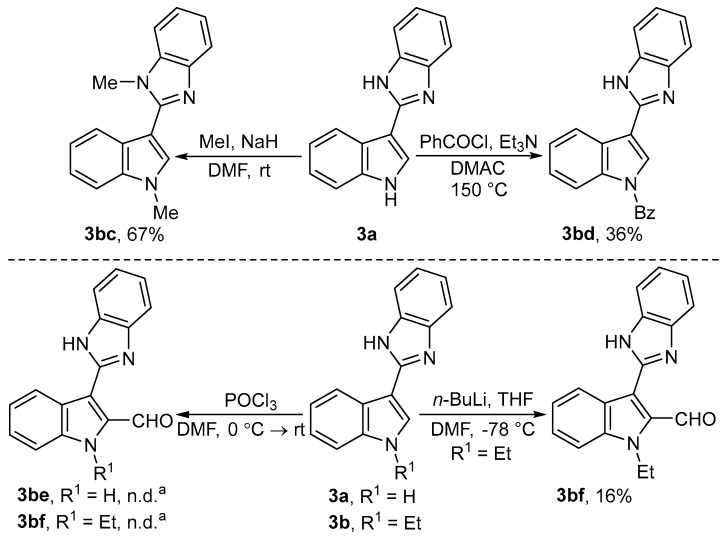
The functionalization of indolylbenzo[*d*]imidazoles **3a**, **b**. ^a^ n.d.—not detected.

Finally, we studied the reactivity of the model 2-(1*H*-indol-3-yl)-1*H*-benzo[*d*]imidazole (**3a**). In particular, we carried out alkylation, acylation, and formylation reactions according to known procedures (Scheme 3). *N*-Methylated indolylbenzimidazole **3bc** can be formed via sequential treatment of starting substrate **3a** with sodium hydride and methyl iodide at room temperature for 5 min. As a result of the reaction, we observed the formation of disubstituted indolylbenzimidazole **3bc** in a good yield (67%). The acylation reaction of indolylbenzimidazole **3a** was accompanied by the formation of a mixture of unidentified by-products and incomplete conversion of the starting substrate. Monosubstituted indolylbenzimidazole **3bd** was isolated in a 36% yield. An attempt to obtain formylated 2-(1*H*-indole-3-yl)-1*H*-benzo[*d*]imidazole **3be** under standard conditions of the Vilsmeier–Haack reaction was unsuccessful. In this case, we observed abundant tarring and decomposition of the reaction mixture. We assumed that protecting the nitrogen atom of the indole fragment would minimize side reactions. But this hypothesis did not lead to a positive result. Next, we found that the treatment of 2-(1-ethyl-1*H*-indol-3-yl)-1*H*-benzo[*d*]imidazole (**3b**) with *n*-butyllithium in absolute tetrahydrofuran with dimethylformamide at −78 °C afforded the product **3bf** in a 16% yield.

### 2.2. In Vitro Biological Evaluation

#### 2.2.1. Antimicrobial Activity

The vast majority of synthesized compounds were tested for their antimicrobial activity against *Candida albicans*, *Escherichia coli*, *Staphylococcus aureus*, and *Mycobacterium smegmatis*. It was shown that most of the studied substances demonstrated antimicrobial activity against two or more microbial species from different groups. In our study, 10 compounds were found to have high activity against *C. albicans*, while 22 compounds demonstrated a moderate level of activity (see Supplementary Materials). The most active compounds are indolylbenzo[*d*]imidazoles **3ad**, **3ag**, and **3aq** (Table 1). Previously, it was shown that some derivatives of benzimidazoles exhibit antimycotic activity [30,31,32].

None of the compounds showed high activity against the Gram-negative bacterium *E. coli*. Some of the tested compounds, **3q**, **3r**, **3v**, **3w**, and **3af**, showed moderate activity (MIC 125 µg/mL) against this microorganism. Benzimidazoles **3z** and **3at** showed an MIC of 250 μg/mL. At the same time, 2-(furan-2-yl)-1*H*-benzo[*d*]imidazole (**3at**) was only bacteriostatic but not bactericidal against *E. coli.* Most of the compounds that have shown antibacterial activity against *E. coli* are the 5-substituted benzimidazoles **3q**, **3r**, **3v**, and **3w**. The low activity of benzimidazoles against Gram-negative bacteria was also observed in other studies [26,30]. This is probably due to the structure of the cell wall of this group of bacteria, which contains an outer membrane. The outer membrane effectively prevents the transport of hydrophobic compounds into the cell.

Nine compounds from the library showed high and twenty-two showed moderate inhibitory effects against Gram-positive *S. aureus* (MIC < 16 µg/mL and 17–125 µg/mL, respectively). The most active substances **3ao** and **3aq** showed an MIC < 1 µg/mL against staphylococci, including MRSA. The MIC against *S. aureus* for compounds **3aa** and **3ad** did not exceed 7.8 µg/mL, which was a promising result. Activity against staphylococci was found in most tested compounds. This result deserves attention since benzimidazole derivatives were previously thought to have predominantly antifungal activity.

The target of benzimidazole binding in most eukaryotic parasites is tubulin, which is involved in the formation of the cytoskeleton [33,34,35,36]. Upon exposure of eukaryotic cells to benzimidazoles, the process of cell division is damaged. However, bacteria do not contain tubulin, and the target for these substances in prokaryotic cells is obviously different. It is even more interesting that the synthesized library contains some substances **3j**, **3y**, and **3al** with selective activity against staphylococci, which do not have an inhibitory effect against fungi, Gram-negative bacteria, or mycobacteria. Previously, the antistaphylococcal activity of benzimidazoles was registered in some studies; however, MIC values were 10s and 100s of µg/mL [26,30,37]. There are data on the activity of these compounds with MICs of 4 µg/mL [38], 3.12 µg/mL [39], and 6 µg/mL [40]. In our studies, nine compounds showed the high activity against staphylococci: **3j**, **3n**, **3aa**, **3ab**, **3ad**, **3ag**, **3ao**, **3aq**, and **3az**, and two of them (**3ao** and **3aq**) demonstrated an MIC < 1 µg/mL.

A special group of microorganisms are mycobacteria. They include the causative agents of dangerous diseases, primarily tuberculosis. Bacteria in this group have a unique cell wall structure and some metabolic features that provide them with reduced sensitivity to many antibiotics. To test the antimycobacterial activity of the compounds, we used the model object *M. smegmatis* mc^2^155, which has a high growth rate and is not pathogenic. Seventeen compounds demonstrated moderate antimicrobial activity against *M. smegmatis* (MIC 17–125 µg/mL). Two of the tested substances **3i** and **3ag** showed a high ability to inhibit the growth of mycobacteria (MIC 7.8 and 3.9 µg/mL, respectively). Since mycobacteria have probably been the most “problematic” group of bacteria in terms of antibiotic therapy for decades, the discovery of a sufficiently high antimycobacterial activity in synthesized compounds seems promising.

A comparative evaluation of the activity of compounds **3o** and **3ao** showed that the introduction of the bromine atom into the core of benzimidazole leads to a significant increase in antimicrobial activity. In this case, the MIC against *S. aureus* decreased from 31.1 to 0.98 µg/mL, and against *C. albicans* from 62.5 to 15.6 µg/mL. It should be noted that 2-(1*H*-indol-3-yl)-6,7-dimethyl-1*H*-benzo[*d*]imidazole (**3ae**) showed moderate activity against *C. albicans*; *S. aureus*, including MRSA; and *M. smegmatis*. The introduction of a bromine atom into the imidazole fragment (for **3aq**) significantly improved the antimicrobial activity: it increased by 32 times against *S. aureus*, by 16 times against MRSA, and by 8 times against *C. albicans*.

*N*-Methylated indolylbenzimidazole **3b** exhibited moderate antimicrobial activity against *C. albicans*; *S. aureus*, including MRSA; and *M. smegmatis*. The closest structural analogue **3ag**, containing methyl in the imidazole fragment, demonstrated significantly higher bacteriostatic and mycostatic activity: the MIC against *S. aureus* decreased by 2 times, against MRSA it decreased by 4 times, against mycobacteria it decreased by 16 times, and against *C. albicans* it decreased by 32 times. However, in this case, improving the ability to inhibit bacterial growth was not accompanied by an increase in the bactericidal effect. On the contrary, a strong increase in mycostatic activity (MIC reduced by 32 times) coincided with an increase in lethal activity against *C. albicans* (MFC reduced by 32 times).

Comparing the antimicrobial activity in the group of 5,7-disubstituted 2-(1*H*-indol-3-yl)-1*H*-benzo[*d*]imidazoles **3z**-**ad**, it should be noted that the 7-bromo-2-(1*H*-indol-3-yl)-5-methyl-1*H*-benzo[*d*]imidazole (**3ab**) demonstrated higher antimicrobial activity against *C. albicans*; *S. aureus*, MRSA; and *M. smegmatis* than 7-bromo-2-(1*H*-indol-3-yl)-5-methoxy-1*H*-benzo[*d*]imidazole (**3ac**). An increase in antistaphylococcal activity was observed for 7-bromo-5-chloro-2-(1*H*-indol-3-yl)-1*H*-benzo[*d*]imidazole (**3aa**): the MIC against *S. aureus* ATCC 25923 was 7.8 μg/mL and MRSA was 3.9 μg/mL. At the same time, the replacement of a chlorine atom with bromine also led to an increase in the antimicrobial activity of the compound **3ad**.

Interestingly, the furo- and thieno-derivatives **3at** and **3au**, respectively, of benzimidazoles show a decrease in activity against all studied microorganisms, with the exception of *E. coli* in the case of **3at**, where we observed a slight increase in bacteriostatic action. We also analyzed the antimicrobial properties of the target molecule after replacing the imidazole cycle with a thiazole cycle. Replacement of the imidazole fragment in compound **3a** with a thiazole one, **3az**, led to a significant increase in inhibitory but not bactericidal activity against staphylococci (16- and 8-times decrease in MIC against *S. aureus* ATCC 25923 and MRSA, respectively). The inhibitory and fungicidal activities against *C. albicans* were not changed in this case.

2-(5-Iodo-1*H*-indol-3-yl)-1*H*-benzo[*d*]imidazole (**3n**) can be compared with the structurally close 2-(5-iodo-1*H*-indol-3-yl)quinazolin-4(3*H*)-one [25] that has a pyrimidinone cycle instead of imidazole ring and similar spectrum of antimicrobial activity. The antimycobacterial activity of compound **3n** was higher than in its nearest homolog.

#### 2.2.2. Antibiofilm Activity

Many microorganisms are able to form biofilms. The cells in biofilms are much less sensitive to antimicrobial agents than planktonic ones. It is known that staphylococci and fungi of the genus *Candida* are able to form robust biofilms [41,42]. With regard to the high activity of a number of the synthesized compounds against *S. aureus* and *C. albicans*, we tested their ability to kill cells within mature biofilms and to affect the biofilm formation process. Minimal biofilm eradication concentrations (MBECs) against *S. aureus* and *C. albicans* were determined for the most active compounds **3aa**, **3ad**, **3ao**, and **3aq**. All tested compounds were able to effectively kill cells in biofilms of staphylococci and fungi of the genus *Candida*. The most effective against staphylococci was indolylbenzimidazole **3aq** (Table 2).

**Figure 2:**
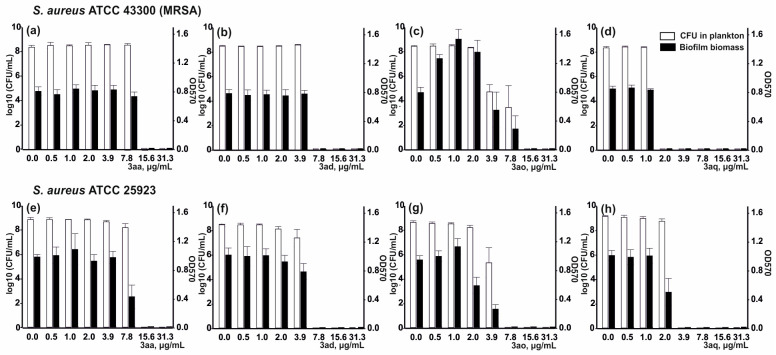
Influence of sublethal concentrations of 7-bromo-5-chloro-2-(1*H*-indol-3-yl)-1*H*-benzo[*d*]imidazole (**3aa**), 5,7-dibromo-2-(1*H*-indol-3-yl)-1*H*-benzo[*d*]imidazole (**3ad**), 5-bromo-2-(5-bromo-1*H*-indol-3-yl)-1*H*-benzo[*d*]imidazole (**3ao**), and 2-(5-bromo-1*H*-indol-3-yl)-6,7-dimethyl-1*H*-benzo[*d*]imidazole (**3aq**) on the *S. aureus* ATCC 43300 (MRSA) (**a**–**d**) and *S. aureus* ATCC 25923 (**e**–**h**) biofilm biomass (OD570) and the number of colony-forming units (CFU) in plankton. Diagrams show the mean values (±SE) of three to five experiments.

The study of the effect of compounds **3aa**, **3ad**, **3ao**, and **3aq** on biofilm formation showed that they effectively prevented the biofilm formation of *S. aureus* ATCC 21923 and MRSA by reducing the number of living cells in the planktonic culture (Figure 2). The activity of the synthesized substances against staphylococcal biofilms significantly exceeded the effect of the control antibiotic amikacin (see Supplementary Materials).

The sublethal concentrations of 5-bromo-2-(5-bromo-1*H*-indol-3-yl)-1*H*-benzo[*d*]imidazole (**3ao**) stimulated staphylococci biofilm formation. This effect may be caused by the stress action of the antibacterial agent, since it is known that biofilm formation can be one of the components of bacterial cell stress response. However, in this case, the regulatory effect of sublethal concentrations of benzimidazole **3ao** on genes encoding metabolites involved in the process of bacteria biofilm formation cannot be completely excluded (Figure 2).

### 2.3. In Silico Characterization of Benzimidazole Derivatives Binding (p)ppGpp Synthetases/Hydrolases, Pyruvate Kinases, and FtsZ Proteins

#### Molecular Docking of Benzimidazole Derivatives

In this study, we employed in silico molecular docking calculations to unravel the possible mechanisms of action and potential targets of the investigated compounds. Initially, we conducted virtual screening on (p)ppGpp synthetase/hydrolases: *E. coli* RelA, *S. aureus* Rel, and *M. smegmatis* Rel_Msm_. The respective predicted 3D structures (P0AG20, P0A0F0, A0QWJ6) were obtained from the AlphaFold database [43]. To identify the active site of the synthetase domain, we utilized the Rel_Seq_ crystal structure (1VJ7), which included a co-crystallized GDP substrate. It is worth noting that (p)ppGpp synthetase/hydrolases belong to non-essential bacterial pathways and their inhibition may not necessarily correlate with strong antibacterial activity. The results of our molecular docking analysis revealed that the majority of the compounds were capable of binding to the active sites of (p)ppGpp synthetases (Table 3). 

**Figure 3:**
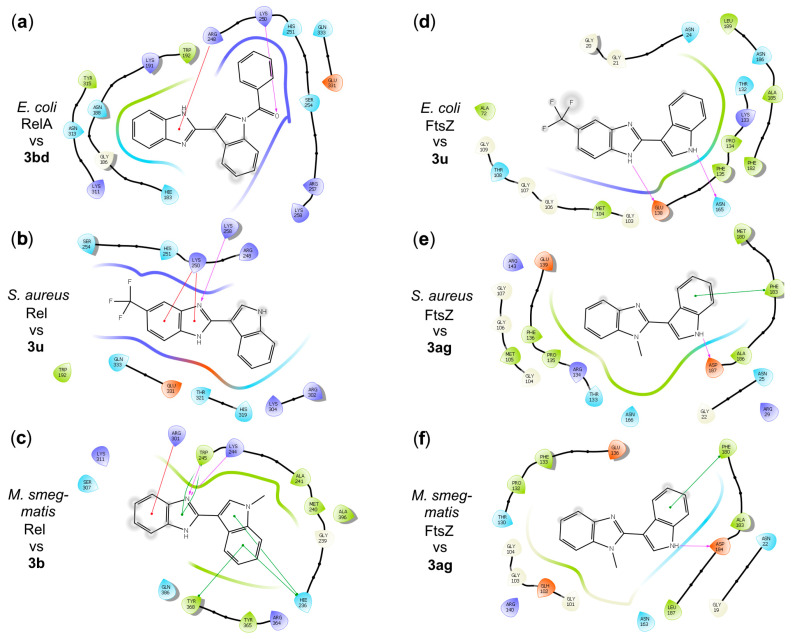
The compounds that exhibit the most favorable binding energies in molecular docking calculations in their interaction with (p)ppGpp synthetases/hydrolases: (**a**) *E. coli* RelA, (**b**) *S. aureus* Rel, (**c**) *M. smegmatis* Rel; with FtsZ proteins: (**d**) *E. coli* FtsZ, (**e**) *S. aureus* FtsZ, (**f**) *M. smegmatis* FtsZ.

Among them, the compound **3bd** demonstrated the most favorable binding energy of −6.101 kcal/mol with *E. coli* RelA (Figure 3a). This value was slightly less favorable than the binding energy of the reference ligand relacin (−6.757 kcal/mol), which has been demonstrated to inhibit this enzyme in vitro [23]. Compound **3u** displayed the most favorable binding energy, measuring −4.719 kcal/mol, in its interaction with *S. aureus* Rel (Figure 3b). Furthermore, for *M. smegmatis* Rel, compound **3b** exhibited a more favorable binding energy of −5.615 kcal/mol (Figure 3c) compared to the reference ligand DMNP (−3.309 kcal/mol), which has been shown to inhibit this target in vitro [44]. The compounds **3** exhibited an average binding energy of −4.57 kcal/mol for *E. coli* RelA, −4.01 kcal/mol for *S. aureus* Rel, and −4.54 kcal/mol for *M. smegmatis* Rel. The results indicate the possibility to use compounds **3** to inhibit the (p)ppGpp-synthesizing activity of RSH proteins.

In attempts to provide explanation for the observed antibacterial activity of some investigated compounds, we conducted molecular docking on essential proteins FtsZ and pyruvate kinase. The protein structures of *E. coli*, *S. aureus*, and *M. smegmatis* FtsZ were obtained from AlphaFold (P0A9A6, P0A031, A0A8B4R473). Compounds lacking substantial antibacterial or antifungal activity (MIC ≥ 250 mg/mL) or lacking activity data were excluded from this analysis. In order to evaluate the ability of benzimidazoles to bind to the FtsZ protein of *S. aureus* (crystal structure PDB 4DXD), we selected its allosteric site specific to a known inhibitor PC190723. [45]. However, the synthesized compounds **3** were unable to fit in this binding pocket via this mechanism. Thus, we decided to investigate the possibility of interaction in the active site. The GTP-binding site was located using the structure of *S. aureus* FtsZ (PDB 3WGN) [46]. The results demonstrated a strong interaction of benzimidazoles and their analogs **3** in the GTP-binding site of FtsZ proteins from all three species (Table 4). The native substrate GTP possessed favorable low binding energies in its binding site (−7.500 kcal/mol). The lowest binding energy to *E. coli* FtsZ was demonstrated by **3u** (−6.546 kcal/mol) (Figure 3d). For both *S. aureus* and *M. smegmatis* FtsZ, the strongest binding was observed for **3ag** (−5.756 and −6.139 kcal/mol) (Figure 3e,f). The tested compounds **3** demonstrated an average binding energy of −5.66 kcal/mol in interaction with *E. coli* FtsZ, −4.97 kcal/mol with *S. aureus* FtsZ, and −5.46 kcal/mol with *M. smegmatis* FtsZ.

**Figure 4:**
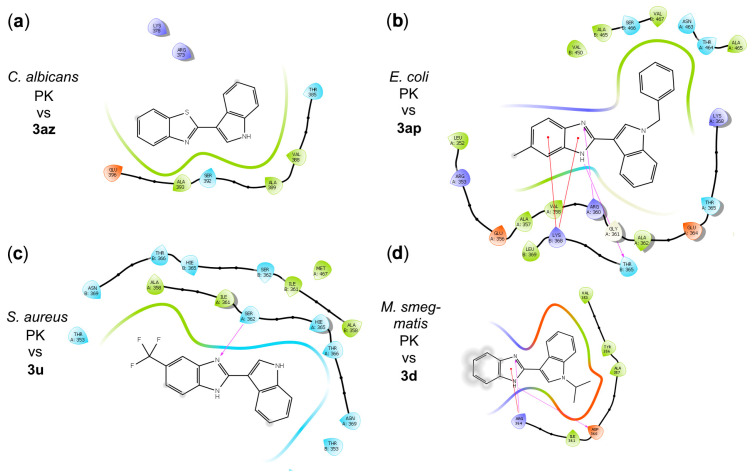
The compounds that exhibit the most favorable binding energies in molecular docking calculations in their interaction with pyruvate kinases (PK): (**a**) *C. albicans* PK; (**b**) *E. coli* PK; (**c**) *S. aureus* PK; (**d**) *M. smegmatis* PK.

The pyruvate kinase (PK) enzyme is present in both bacterial and fungal cells. Targeting this enzyme could explain the observed antibacterial and antifungal properties of the investigated compounds. We evaluated the possibility of an interaction of benzimidazole derivatives **3** with PK according to the mechanism described earlier [20]. Crystal structures of *E. coli* PK1 (PDB 1PKY) and *S. aureus* PK (PDB 3T0T) were employed for molecular docking. *M. smegmatis* PK was obtained using structural alignment of AlphaFold-predicted PK homomer with *M. tuberculosis* PK. Similarly, *C. albicans* PK was obtained by aligning with *S. aureus* PK (PDB 3T0T). The strongest binders turned out to be *C. albicans* PK—**3az** (−6.224 kcal/mol); *E. coli* PK—**3ap** (−8.114 kcal/mol); *S. aureus* PK—**3u** (−8.628 kcal/mol); and *M. smegmatis* PK—**3d** (−5.982 kcal/mol) (Figure 4).

Target identification via molecular docking provided three plausible models of interaction for the investigated compounds. (p)ppGpp synthetases/hydrolases, FtsZ proteins, or pyruvate kinases could be involved in the mechanism of antibacterial action of the studied compounds. It is worth noting that the compounds that frequently attained favorable binding energies when interacting with the investigated targets were **3d**, **3o**, **3t**, **3u**, **3x**, **3aa**, **3af,** and **3ag**. With the exception of **3d**, these compounds exhibited notable antibacterial activities. However, differences in the predicted binding energies of different structures were not always fully consistent with the observed antibacterial activity of the compounds. Molecular docking produced favorable binding energies for the compounds to interact with *E. coli* target proteins, but *E. coli* cells were found to be susceptible to only several compounds. This could be attributed to the poor permeability of *E. coli* cells wall for hydrophobic compounds. Further experimental efforts for target identification are required.

## 3. Materials and Methods

### 3.1. Instrumentation

^1^H and ^13^C NMR spectra were recorded on a «Bruker Avance III HD 400» (400 MHz for ^1^H and 100 MHz for ^13^C NMR) at 40 °C. The chemical shifts (δ) were measured in ppm with respect to the solvent ([D_6_] DMSO, ^1^H: δ = 2.50 ppm, ^13^C: δ = 39.52 ppm). The coupling constants (*J*) are given in Hertz (Hz). The splitting patterns of apparent multiplets associated with the averaged coupling constants were designated as s (singlet), d (doublet), t (triplet), q (quartet), m (multiplet), dd (doublet of doublets), and br. (broadened). High-resolution mass measurements (HRMS) were carried out using a BrukermicroTOF-QTM ESI-TOF mass spectrometer. GC/MS analysis was performed on an «Agilent 7890B» interfaced to an «Agilent 5977A» mass-selective detector. The melting points were determined with a «Stuart SMP 40». Data sets for X-ray diffraction were collected with a «New Xcalibur, Ruby» diffractometer. Column chromatography was performed on silica gel Macherey Nagel (40–63 μm). Flash column chromatography was performed over silica gel (0.04–0.063 mm), using a mixture of EtOAc and petroleum ether as an eluent. TLC plates were visualized via exposure to ultraviolet light. All the reactions were carried out using freshly distilled and dry solvents from solvent stills.

### 3.2. Materials

Starting 1*H*-indole-3-carboxaldehyde (purity 97%), furfural (99%), 2-thiophenecarboxaldehyde (98%), 4-pyridinecarboxaldehyde (97%), cyclohexanecarbaldehyde (97%), 1,2-phenylenediamine (99.5%), 2-aminothiophenol (99%), benzoyl chloride (99%), iodomethane (99%), benzyl bromide (98%), and *n*-butyllithium solutions (2.5 M in hexanes) were purchased from Sigma-Aldrich (St. Louis, MO, USA). The reagents for the analysis of the antimicrobial activity were as follows: cefazolin—MP Biomedicals (Illkirch, France); cefotaxime—PROMED (Saransk, Russia); amikacin sulfate—Sigma-Aldrich (St. Louis, MO, USA); rifampicin—GERBU (Gaiberg, Germany); fluconazole—Sigma-Aldrich (St. Louis, MO, USA); isoniazid—Sigma (St. Louis, MO, USA); LB broth—VWR (Radnor, PA, USA); and LB agar—Sigma (St. Louis, MO, USA).

### 3.3. Synthesis

#### 3.3.1. Synthesis of the Starting Substrates

Starting 1-methyl-1*H*-indole-3-carbaldehyde (**1b**), 1-ethyl-1*H*-indole-3-carbaldehyde (**1c**), 1-isopropyl-1*H*-indole-3-carbaldehyde (**1d**), 1-benzyl-1*H*-indole-3-carbaldehyde (**1f**), 1-*n*-butyl-1*H*-indole-3-carbaldehyde (**1g**), 5-bromo-1-methyl-1*H*-indole-3-carbaldehyde (**1p**) [47], 2-phenyl-1*H*-indole-3-carbaldehyde (**1h**), 2-(4-nitrophenyl)-1*H*-indole-3-carbaldehyde (**1i**), 2-(4-fluorophenyl)-1*H*-indole-3-carbaldehyde (**1j**), 2-(thiophen-2-yl)-1*H*-indole-3-carbaldehyde (**1k**), 2-(4-hydroxyphenyl)-1*H*-indole-3-carbaldehyde (**1l**), 2-(4-methoxyphenyl)-1*H*-indole-3-carbaldehyde (**1m**) [48], 1-phenyl-1*H*-indole-3-carbaldehyde (**1e**), 5-iodo-1*H*-indole-3-carbaldehyde (**1n**), 5-bromo-1*H*-indole-3-carbaldehyde (**1o**), 1-methyl-1*H*-pyrrole-2-carbaldehyde (**1t**) [49], benzofuran-2-carbaldehyde (**1q**) [50], 3-(4-fluorophenyl)-1-phenyl-1*H*-pyrazole-4-carbaldehyde (**1v**) [51], and 2-*N*-phenylbenzene-1,2-diamine (**2v**) [52] were synthesized according to a known procedure. Starting 4-chlorobenzene-1,2-diamine (**2b**), 4-fluorobenzene-1,2-diamine (**2c**), 4,5-dimethylbenzene-1,2-diamine (**2e**), 4-(trifluoromethyl)benzene-1,2-diamine (**2f**), 4-methoxybenzene-1,2-diamine (**2g**), 4-methylbenzene-1,2-diamine (**2h**), 4,5-dichlorobenzene-1,2-diamine (**2i**), 4-nitrobenzene-1,2-diamine (**2j**), 3-methylbenzene-1,2-diamine (**2k**), 3,4-dimethylbenzene-1,2-diamine (**2p**), 3,4-difluorobenzene-1,2-diamine (**2q**), 4-[(3,4-diaminophenyl)methyl]benzene-1,2-diamine (**2w**), and phenazine-2,3-diamine (**2x**) were synthesized according to a known procedure [53]. *N*-Substituted phenylenediamines 2-*N*-methylbenzene-1,2-diamine (**2r**), 2-*N*-(propan-2-yl)benzene-1,2-diamine (**2s**), *N*-allyl-*o*-phenylenediamine (**2t**), and 2-*N*-benzylbenzene-1,2-diamine (**2u**) were synthesized according to a known procedure [54]. Starting 4-bromobenzene-1,2-diamine (**2d**), 3-bromo-5-chlorobenzene-1,2-diamine (**2l**), 3-bromo-5-methylbenzene-1,2-diamine (**2m**), 3-bromo-5-methoxybenzene-1,2-diamine (**2n**), and 3,5-dibromobenzene-1,2-diamine (**2o**) were synthesized according to a known procedure [55].

#### 3.3.2. General Procedure for the Synthesis of 1*H*-Benzo[*d*]imidazoles **3a**-**bb**

To a suspension of aldehyde **1** (1.3 mmol) and Na_2_S_2_O_5_ (4.5 mmol, 0.855 g) in *N*,*N*-dimethylacetamide (2.5 mL), benzene-1,2-diamine or 2-aminothiophenol **2** (1.3 mmol) and H_2_O (4.5 mmol, 81 μL) were added. The reaction mixture was heated at 150 °C and vigorously stirred for 2–16 h (TLC control). Then, the reaction mixture was poured into H_2_O (50 mL). The formed precipitate was filtered. The products **3e**, **3h**, **3u**-**w**, **3y**, **3ac**, **3ai**, **3an**, **3az, 3ba**-**bd**, and **3bf** were purified using column chromatography on silica gel using the mixture of petroleum ether/EtOAc (gradient chromatography from 20:1 to 1:1) as the eluent and recrystallized from a suitable solvent. The products **3a**-**d**, **3f**, **3g**, **3i**-**t**, **3x**, **3z**, **3aa**, **3ab**, **3ad**-**ah**, **3aj-am,** and **3ao**-**ay** were recrystallized from a suitable solvent without prior purification using column chromatography.

*2-(1H-Indol-3-yl)-1H-benzo[d]imidazole* (**3a**) [56]. A yield of 0.288 g (95%), 8 h, white solid. Mp. = 215–216 °C (DMAC/H_2_O, lit. [56] 226–228 °C). ^1^H NMR (400 MHz, DMSO–*d*_6_) δ = 12.04 (br.s, 1H), 8.39–8.33 (m, 2H), 7.69–7.67 (m, 2H), 7.60–7.58 (m, 1H), and 7.35–7.27 (m, 4H) ppm; ^13^C{^1^H} NMR (100 MHz, DMSO–*d*_6_) δ = 147.7, 136.5, 135.0 (br.s), 129.1, 124.1, 123.2, 122.8, 121.0, 120.3, 113.6, 112.5, and 102.5 ppm.

*2-(1-Methyl-1H-indol-3-yl)-1H-benzo[d]imidazole* (**3b**) [57]. A yield of 0.254 g (79%), 6 h, light-beige solid. Mp. = 214–216 °C (EtOH, lit. [57] 231–233 °C). ^1^H NMR (400 MHz, DMSO–*d*_6_) δ = 8.38 (br.s, 1H), 8.34–8.29 (m, 1H), 7.75–7.71 (m, 2H), 7.69–7.66 (m, 1H), 7.45–7.36 (m, 4H), and 3.98 (s, 3H) ppm; ^13^C{^1^H} NMR (100 MHz, DMSO–*d*_6_) δ = 146.5, 137.2, 133.8, 133.2 (br.s), 124.2, 124.0, 123.2, 121.7, 120.0, 113.4, 111.2, 99.7, and 33.4 ppm.

*2-(1-Ethyl-1H-indol-3-yl)-1H-benzo[d]imidazole* (**3c**) [58]. A yield of 0.299 g (88%), 7 h, light-beige solid. Mp. = 245–247 °C, decomposition (DMAC/H_2_O, lit. [58] 248–249 °C). ^1^H NMR (400 MHz, DMSO–*d*_6_) δ = 12.39 (br.s, 1H), 8.53–8.51 (m, 1H), 8.17 (s, 1H), 7.59–7.52 (m, 3H), 7.30–7.21 (m, 2H), 7.16–7.12 (m, 2H), 4.32 (q, *J* = 7.2 Hz, 2H), and 1.47 (t, *J* = 7.2 Hz, 3H) ppm; ^13^C{^1^H} NMR (100 MHz, DMSO–*d*_6_) δ = 149.0, 136.1, 128.2, 125.6, 122.1, 121.5, 121.0, 120.3, 110.1, 105.9, 40.6, and 15.1 ppm.

*2-(1-Isopropyl-1H-indol-3-yl)-1H-benzo[d]imidazole* (**3d**). A yield of 0.350 g (98%), 7 h, light-beige solid. Mp. = 179 °C, decomposition (DMAC/H_2_O). ^1^H NMR (400 MHz, DMSO–*d*_6_) δ = 8.49–8.47 (m, 1H), 8.37 (s, 1H), 7.66–7.64 (m, 1H), 7.62–7.58 (m, 2H), 7.32–7.26 (m, 2H), 7.25–7.20 (m, 2H), 4.92–4.82 (m, 1H), and 1.55 (d, *J* = 6.8 Hz, 6H) ppm; ^13^C{^1^H} NMR (100 MHz, DMSO–*d*_6_) δ = 148.5, 137.5 (br.s), 135.9, 126.4, 125.2, 122.3, 121.9, 121.2, 120.8, 113.8 (br.s), 110.5, 104.5, 47.1, and 22.3 ppm. HRMS (ESI) calcd for C_18_H_18_N_3_ [M+H]^+^ 276.1495; found 276.1496.

*2-(1-Phenyl-1H-indol-3-yl)-1H-benzo[d]imidazole* isolated as a solvate with DMAC (1:0.8DMAC) (**3e**). A yield of 0.418 g (84%), 7 h, light-beige solid. Mp. = 231–232 °C (EtOH). ^1^H NMR (400 MHz, DMSO–*d*_6_) δ = 8.68–8.64 (m, 1H), 8.44 (br.s, 1H), 7.72–7.59 (m, 7H), 7.53–7.48 (m, 1H), 7.37–7.32 (m, 2H), and 7.22–7.17 (m, 2H) ppm; ^13^C{^1^H} NMR (100 MHz, DMSO–*d*_6_) δ = 148.2, 138.3, 135.8, 130.0, 128.7, 127.2, 126.2, 124.0, 123.4, 122.0, 121.5, 114.2 (br.s), 110.7, and 108.1 ppm. HRMS (ESI) calcd for C_21_H_16_N_3_ [M+H]^+^ 310.1339; found 310.1346.

*2-(1-Benzyl-1H-indol-3-yl)-1H-benzo[d]imidazole* (**3f**) [59]. A yield of 0.399 g (95%), 6.5 h, light-beige solid. Mp. = 214–215 °C (DMAC/H_2_O, lit. [59] 218–220 °C). ^1^H NMR (400 MHz, DMSO–*d*_6_) δ = 8.52–8.49 (m, 1H), 8.28 (s, 1H), 7.64–7.57 (m, 3H), 7.38–7.24 (m, 7H), 7.22–7.18 (m, 2H), and 5.56 (s, 2H) ppm; ^13^C{^1^H} NMR (100 MHz, DMSO–*d*_6_) δ = 148.4, 137.9 (br.s), 137.3, 136.5, 129.9, 128.6, 127.6, 127.3, 125.4, 122.6, 121.7, 121.3, 120.8, 113.9 (br.s), 110.7, 105.3, and 49.5 ppm.

*2-(1-Butyl-1H-indol-3-yl)-1H-benzo[d]imidazole* (**3g**) [58]. A yield of 0.368 g (98%), 6 h, light-beige solid. Mp. = 190–192 °C, decomposition (DMAC/H_2_O, lit. [58] 207–208 °C). ^1^H NMR (400 MHz, DMSO–*d*_6_) δ = 8.47 (d, *J* = 7.6 Hz, 1H), 8.25 (s, 1H), 7.63–7.59 (m, 3H), 7.32–7.21 (m, 4H), 4.30 (t, *J* = 6.8 Hz, 2H), 1.87–1.79 (m, 2H), 1.38–1.28 (m, 2H), and 0.93 (t, *J* = 7.2 Hz, 3H) ppm; ^13^C{^1^H} NMR (100 MHz, DMSO–*d*_6_) δ = 147.3, 136.3 (br.s), 135.4, 129.1, 124.1, 121.4, 121.0, 120.1, 119.7, 112.8, 109.5, 103.0, 44.6, 30.5, 18.3, and 12.4 ppm.

*2-(2-Phenyl-1H-indol-3-yl)-1H-benzo[d]imidazole* isolated as a solvate with DMAC (1:0.3DMAC) (**3h**), for no solvate [60]. A yield of 0.421 g (96%), 14 h, white solid. Mp. = 132–133 °C (EtOH, lit. for no solvate [60] 235–236 °C). ^1^H NMR (400 MHz, DMSO–*d*_6_) δ = 11.96 (br.s, 1H), 7.85 (d, *J* = 8.0 Hz, 1H), 7.67–7.64 (m, 2H), 7.60–7.55 (m, 2H), 7.52 (d, *J* = 8.0 Hz, 1H), 7.48–7.39 (m, 3H), 7.27–7.21 (m, 3H), and 7.19–7.15 (m, 1H) ppm; ^13^C{^1^H} NMR (100 MHz, DMSO–*d*_6_) δ = 144.1, 134.6, 132.4, 127.7, 125.0, 124.8, 124.6, 124.1, 119.0, 118.4, 116.8, 116.1, 110.9, 110.7 and 108.0 ppm.

*2-[2-(4-Nitrophenyl)-1H-indol-3-yl]-1H-benzo[d]imidazole* isolated as a solvate with DMAC (1:0.3DMAC) (**3i**), for no solvate [60]. A yield of 0.229 g (46%), 7 h, brown solid. Mp. = 132–133 °C (EtOH, lit. for not solvate [60] 357–358 °C). ^1^H NMR (400 MHz, DMSO–*d*_6_) δ = 11.80 (br.s, 1H), 11.47 (br.s, 1H), 7.84 (d, *J* = 7.6 Hz, 1H), 7.55–7.46 (m, 2H), 7.44–7.41 (m, 1H), 7.36–7.34 (m, 2H), 7.16–7.06 (m, 4H), and 6.62–6.60 (m, 2H) ppm; ^13^C{^1^H} NMR (100 MHz, DMSO–*d*_6_) δ = 149.1, 148.8, 139.0, 135.6, 129.1, 128.3, 121.4, 121.0, 120.9, 119.7, 119.5, 118.8, 113.6, 111.0, and 101.3 ppm.

*2-[2-(4-Fluorophenyl)-1H-indol-3-yl]-1H-benzo[d]imidazole* (**3j**). A yield of 0.332 g (78%), 6 h, light-beige solid. Mp. = 283–284 °C (DMAC/H_2_O). ^1^H NMR (400 MHz, DMSO–*d*_6_) δ = 12.03 (br.s, 1H), 11.84 (s, 1H), 7.89 (d, *J* = 8.0 Hz, 1H), 7.75–7.70 (m, 2H), 7.54–7.43 (m, 3H), 7.33–7.27 (m, 2H), 7.25–7.21 (m, 1H), and 7.18–7.13 (m, 3H) ppm; ^13^C{^1^H} NMR (100 MHz, DMSO–*d*_6_) δ = 162.0 (d, *J*_CF_ = 244.0 Hz), 148.0, 136.5, 135.9, 130.5 (d, *J*_CF_ = 8.0 Hz), 128.2 (d, *J*_CF_ = 3.0 Hz), 127.8, 122.4, 121.2, 121.1, 120.2, 120.0, 115.4 (d, *J*_CF_ = 22.0 Hz), 111.4, and 103.6 ppm. HRMS (ESI) calcd for C_21_H_15_FN_3_ [M+H]^+^ 328.1245; found 328.1253.

*2-[2-(Thiophen-2-yl)-1H-indol-3-yl]-1H-benzo[d]imidazole* (**3k**) [60]. A yield of 0.229 g (56%), 6 h, yellow needles. Mp. = 241–242 °C, decomposition (EtOH, lit. [60] 201–203 °C). ^1^H NMR (400 MHz, DMSO–*d*_6_) δ = 12.61 (br.s, 1H), 9.10 (s, 1H), 8.45–8.44 (m, 1H), 7.92 (d, *J* = 8.0 Hz, 1H), 7.65–7.62 (m, 2H), 7.60–7.57 (m, 2H), 7.43–7.40 (m, 1H), 7.26–7.22 (m, 2H), and 7.16–7.14 (m, 2H) ppm; ^13^C{^1^H} NMR (100 MHz, DMSO–*d*_6_) δ = 145.3, 145.2, 138.7, 134.5, 130.3, 129.7, 128.3, 127.5, 127.0, 126.5, 121.9, 118.5, and 112.1 ppm.

*4-[3-(1H-Benzo[d]imidazol-2-yl)-1H-indol-2-yl]phenol* (**3l**) [61]. A yield of 0.410 g (97%), 6 h, light-yellow solid. Mp. = 280 °C, decomposition (EtOH, lit. [61] 200 °C). ^1^H NMR (400 MHz, DMSO–*d*_6_) δ = 11.87 (s, 1H), 11.62 (s, 1H), 9.67 (s, 1H), 7.86 (d, *J* = 7.6 Hz, 1H), 7.65–7.63 (m, 1H), 7.51–7.49 (m, 2H), 7.46–7.41 (m, 2H), 7.20–7.09 (m, 4H), and 6.85–6.82 (m, 2H) ppm; ^13^C{^1^H} NMR (100 MHz, DMSO–*d*_6_) δ = 157.7, 148.5, 144.0, 138.1, 135.7, 134.5, 129.7, 128.1, 122.5, 121.8, 121.3, 120.8, 119.9, 119.7, 118.1, 115.4, 111.2, 110.9, and 102.3 ppm.

*2-[2-(4-Methoxyphenyl)-1H-indol-3-yl]-1H-benzo[d]imidazole* isolated as a solvate with DMAC (1:0.2DMAC) (**3m**), for no solvate [60]. A yield of 0.456 g (98%), 6 h, light beige solid. Mp. = 133–135 °C (DMAC/H_2_O, lit. for not solvate [60] 223–224 °C). ^1^H NMR (400 MHz, DMSO–*d*_6_) δ = 11.93 (s, 1H), 7.84 (d, *J* = 7.6 Hz, 1H), 7.62–7.58 (m, 4H), 7.51 (d, *J* = 8.0 Hz, 1H), 7.30–7.26 (m, 2H), 7.23 (d, *J* = 8.0 Hz, 1H), 7.19–7.15 (m, 1H), 7.05–7.02 (m, 2H), and 3.81 (s, 3H) ppm; ^13^C{^1^H} NMR (100 MHz, DMSO–*d*_6_) δ = 159.7, 147.6, 138.9, 136.9, 136.8, 135.9, 129.7, 127.6, 123.4, 122.4, 120.5, 119.4, 114.2, 111.5, 100.1, 100.0, and 55.2 ppm.

*2-(5-Iodo-1H-indol-3-yl)-1H-benzo[d]imidazole* (**3n**). A yield of 0.439 g (94%), 14 h, light beige solid. Mp. = 262–263 °C (EtOH). ^1^H NMR (400 MHz, DMSO–*d*_6_) δ = 12.45 (br.s, 1H), 11.74 (s, 1H), 8.90–8.89 (m, 1H), 8.13 (d, *J* = 2.8 Hz, 1H), 7.58–7.56 (m, 2H), 7.49 (dd, *J* = 8.4, 1.6 Hz, 1H), 7.37 (d, *J* = 8.4 Hz, 1H), and 7.18–7.13 (m, 2H) ppm; ^13^C{^1^H} NMR (100 MHz, DMSO–*d*_6_) δ = 148.8, 135.5, 130.1, 129.5, 127.6, 126.8, 121.2, 114.3, 105.9, and 84.2 ppm. HRMS (ESI) calcd for C_15_H_11_IN_3_ [M+H]^+^ 359.9992; found 359.9982.

*2-(5-Bromo-1H-indol-3-yl)-1H-benzo[d]imidazole* (**3o**) [62]. A yield of 0.393 g (97%), 9 h, beige solid. Mp. = 261 °C, decomposition (petroleum ether/EtOAc). ^1^H NMR (400 MHz, DMSO–*d*_6_) δ = 12.50 (br.s, 1H), 11.77 (s, 1H), 8.70 (d, *J* = 2.0 Hz, 1H), 8.18 (d, *J* = 2.8 Hz, 1H), 7.59–7.55 (m, 2H), 7.49 (d, *J* = 8.4 Hz, 1H), 7.34 (dd, *J* = 8.4, 2.0 Hz, 1H), and 7.18–7.14 (m, 2H) ppm; ^13^C{^1^H} NMR (100 MHz, DMSO–*d*_6_) δ = 148.7, 135.2, 127.3, 126.8, 124.7, 123.4, 121.2, 113.9, 112.9, and 106.1 ppm.

*2-(5-Bromo-1-methyl-1H-indol-3-yl)-1H-benzo[d]imidazole* (**3p**). A yield of 0.389 g (92%), 6 h, light-beige solid. Mp. = 246–247 °C, decomposition (DMAC/H_2_O). ^1^H NMR (400 MHz, DMSO–*d*_6_) δ = 8.65–8.64 (m, 1H), 8.22 (s, 1H), 7.64–7.60 (m, 2H), 7.57 (d, *J* = 8.8 Hz, 1H), 7.43 (dd, *J* = 8.8, 2.0 Hz, 1H), 7.26–7.21 (m, 2H), and 3.92 (s, 3H) ppm; ^13^C{^1^H} NMR (100 MHz, DMSO–*d*_6_) δ = 147.6, 137.3 (br.s), 135.8, 132.3, 126.6, 125.0, 123.1, 122.1, 113.9 (br.s), 113.7, 112.6, 103.6, and 33.3 ppm. HRMS (ESI) calcd for C_16_H_13_BrN_3_ [M+H]^+^ 326.0287; found 326.0282.

*5-Chloro-2-(1H-indol-3-yl)-1H-benzo[d]imidazole* isolated as a solvate with DMAC (1:2DMAC) (**3q**), for no solvate [63]. A yield of 0.552 g (94%), 6.5 h, beige needles. Mp. = 123–124 °C (petroleum ether/EtOAc, lit. for not solvate [63] 213–215 °C). ^1^H NMR (400 MHz, DMSO–*d*_6_) δ = 12.53 (br.s, 1H), 11.63 (br.s, 1H), 8.50–8.48 (m, 1H), 8.16 (d, *J* = 2.8 Hz, 1H), 7.58–7.50 (m, 3H), and 7.25–7.14 (m, 3H) ppm; ^13^C{^1^H} NMR (100 MHz, DMSO–*d*_6_) δ = 150.9, 136.5, 126.5, 125.3, 125.0, 122.2, 121.2, 121.1, 120.3, 111.8, and 106.1 ppm.

*5-Fluoro-2-(1H-indol-3-yl)-1H-benzo[d]imidazole* isolated as a solvate with DMAC (1:1.3DMAC) (**3r**). A yield of 0.435 g (90%), 6 h, light-brown needles. Mp. = 121–122 °C (petroleum ether/EtOAc). ^1^H NMR (400 MHz, DMSO–*d*_6_) δ = 12.49 (br.s, 1H), 11.60 (br.s, 1H), 8.51–8.46 (m, 1H), 8.13 (d, *J* = 2.8 Hz, 1H), 7.53–7.48 (m, 2H), 7.35–7.32 (m, 1H), 7.24–7.17 (m, 2H), and 7.00–6.95 (m, 1H) ppm; ^13^C{^1^H} NMR (100 MHz, DMSO–*d*_6_) δ = 158.2 (d, *J*_CF_ = 232.0 Hz), 150.9 (br.s), 136.4, 126.1, 125.0, 122.1, 121.2, 120.2, 111.8, 108.6 (d, *J*_CF_ = 25.0 Hz), and 106.3 ppm. HRMS (ESI) calcd for C_15_H_11_FN_3_ [M+H]^+^ 252.0932; found 252.0938.

*5-Bromo-2-(1H-indol-3-yl)-1H-benzo[d]imidazole* (**3s**) [63]. A yield of 0.353 g (87%), 6.5 h, yellow solid. Mp. = 253–254 °C (DMAC/H_2_O, lit. [63] 248–250 °C). ^1^H NMR (400 MHz, DMSO–*d*_6_) δ = 12.54 (br.s, 1H), 11.62 (s, 1H), 8.50–8.45 (m, 1H), 8.15 (d, *J* = 2.8 Hz, 1H), 7.70 (br.s, 1H), 7.53–7.48 (m, 2H), and 7.28–7.18 (m, 3H) ppm; ^13^C{^1^H} NMR (100 MHz, DMSO–*d*_6_) δ = 150.7, 136.4, 126.5, 125.0, 123.7, 122.2, 121.2, 120.3, 113.1 (br.s), 111.8, and 106.0 ppm.

*2-(1H-Indol-3-yl)-5,6-dimethyl-1H-benzo[d]imidazole* (**3t**). A yield of 0.309 g (91%), 7 h, light-beige solid. Mp. = 212–213 °C, decomposition (DMAC/H_2_O). ^1^H NMR (400 MHz, DMSO–*d*_6_) δ = 12.33 (br.s, 1H), 8.42 (d, *J* = 3.2 Hz, 1H), 8.24–8.19 (m, 1H), 7.67–7.63 (m, 1H), 7.52 (s, 2H), 7.38–7.34 (m, 2H), and 2.40 (s, 6H) ppm; ^13^C{^1^H} NMR (100 MHz, DMSO–*d*_6_) δ = 145.3, 136.6, 134.1, 131.1, 130.0, 123.4, 123.3, 121.6, 119.4, 113.0, 112.9, 99.3, and 19.8 ppm. HRMS (ESI) calcd for C_17_H_16_N_3_ [M+H]^+^ 262.1339; found 262.1338.

*Mixture of 2-(1H-indol-3-yl)-5-trifluoromethyl-1H-benzo[d]imidazole and 2-(1H-indol-3-yl)-6-(trifluoromethyl)-1H-benzo[d]imidazole* (**3u**) (ratio of isomers 1:1) isolated as a solvate with DMAC (1:1DMAC). A yield of 0.203 g (52%), 7 h, white solid. Mp. = 52–60 °C (petroleum ether/EtOAc). ^1^H NMR (400 MHz, DMSO–d_6_) δ = 12.79–12.78 (m, 2H), 11.70–11.68 (m, 2H), 8.52–8.50 (m, 2H), 8.22–8.20 (m, 2H), 7.95 (br.s, 1H), 7.80–7.75 (m, 2H), 7.65 (d, *J* = 8.0 Hz, 1H), 7.54–7.51 (m, 2H), 7.47–7.45 (m, 2H), and 7.26–7.20 (m, 4H) ppm; ^13^C{^1^H} NMR (100 MHz, DMSO–d_6_) δ = 152.4, 151.9, 146.8 (q, *J*_CF_ = 1.0 Hz), 143.8, 136.7 (br.s), 136.5, 133.7, 127.0, 126.7 (q, *J*_CF =_ 32.0 Hz), 126.8, 126.7 (q, *J*_CF =_ 30.4 Hz), 122.5 (q, *J*_CF_ = 270.0 Hz), 126.3, 125.1, 122.3, 121.2, 120.4, 118.4, 118.0 (q, *J*_CF =_ 4.6 Hz), 117.6 (q, *J*_CF =_ 4.2 Hz), 114.7 (q, *J*_CF =_ 4.2 Hz), 111.9, 110.8, 107.4 (q, *J*_CF_ = 4.8 Hz), and 105.9 ppm. HRMS (ESI) calcd for C_16_H_11_F_3_N_3_ [M+H]^+^ 302.0900; found 302.0908.

*2-(1H-Indol-3-yl)-5-methoxy-1H-benzo[d]imidazole* isolated as a solvate with DMAC (1:0.3DMAC) (**3v**). A yield of 0.192 g (51%), 6 h, yellow solid. Mp. = 63–65 °C (DMAC/H_2_O). ^1^H NMR (400 MHz, DMSO–*d*_6_) δ = 12.21 (br.s, 1H), 11.52 (s, 1H), 8.51–8.49 (m, 1H), 8.08 (d, *J* = 2.4 Hz, 1H), 7.50–7.43 (m, 2H), 7.23–7.16 (m, 2H), 7.07 (br.s, 1H), 6.78 (dd, *J* = 8.8, 2.0 Hz, 1H), and 3.81 (s, 3H) ppm; ^13^C{^1^H} NMR (100 MHz, DMSO–*d*_6_) δ = 155.2, 136.4, 125.5, 125.1, 122.0, 121.3, 120.0, 111.7, 109.9 (br.s), 106.8, and 55.4 ppm. HRMS (ESI) calcd for C_16_H_14_N_3_O [M+H]^+^ 264.1131; found 264.1128.

*2-(1H-Indol-3-yl)-5-methyl-1H-benzo[d]imidazole* (**3w**) [64]. A yield of 0.292 g (91%), 8.5 h, light beige solid. Mp. = 253–255 °C (EtOH, lit. [64] 252–254 °C). ^1^H NMR (400 MHz, DMSO–*d*_6_) δ = 12.03 (br.s, 1H), 8.37–8.34 (m, 1H), 8.32 (d, *J* = 2.8 Hz, 1H), 7.61–7.55 (m, 2H), 7.47 (s, 1H), 7.33–7.27 (m, 2H), 7.17 (d, *J* = 8.0 Hz, 1H), and 2.48 (s, 3H) ppm; ^13^C{^1^H} NMR (100 MHz, DMSO–*d*_6_) δ = 147.3, 136.5, 134.9 (br.s), 133.2 (br.s), 132.7, 128.9, 124.5, 124.1, 122.8, 121.0, 120.2, 113.4, 113.1, 112.4, 102.5, and 21.1 ppm.

*5,6-Dichloro-2-(1H-indol-3-yl)-1H-benzo[d]imidazole* (**3x**). A yield of 0.365 g (93%), 6 h, gray solid. Mp. = 282 °C, decomposition (DMAC/H_2_O). ^1^H NMR (400 MHz, DMSO–*d*_6_) δ = 12.70 (br.s, 1H), 11.69 (s, 1H), 8.49–8.44 (m, 1H), 8.19 (d, *J* = 2.8 Hz, 1H), 7.76 (s, 2H), 7.54–7.49 (m, 1H), and 7.26–7.19 (m, 2H) ppm; ^13^C{^1^H} NMR (100 MHz, DMSO–*d*_6_) δ = 152.1, 136.5, 127.0, 125.0, 123.2, 122.3, 121.1, 120.4, 111.9, and 105.7 ppm. HRMS (ESI) calcd for C_15_H_10_Cl_2_N_3_ [M+H]^+^ 302.0246; found 302.0244.

*Mixture of 2-(1H-indol-3-yl)-6-nitro-1H-benzo[d]imidazole and 2-(1H-indol-3-yl)-5-nitro-1H-benzo[d]imidazole* (**3y**) (ratio of isomers 1:1) [65]. A yield of 0.228 g (63%), 6.5 h, light yellow oil. ^1^H NMR (400 MHz, DMSO–*d*_6_) δ = 12.22 (br.s, 1H), 11.55 (s, 1H), 11.43 (s, 1H), 9.85 (s, 1H), 8.52–8.46 (m, 2H), 8.10–8.08 (m, 1H), 8.01–7.94 (m, 2H), 7.52–7.41 (m, 3H), 7.26–7.11 (m, 6H), 6.72–6.67 (m, 1H), and 6.52–6.48 (m, 1H) ppm; ^13^C{^1^H} NMR (100 MHz, DMSO–*d*_6_) δ = 149.5, 149.4, 147.2 (br.s), 147.1, 143.8, 136.4, 136.3, 133.6, 125.7, 125.6, 125.1, 125.0, 124.9, 122.1, 121.9, 121.4, 121.3, 120.1, 120.0, 119.8, 113.8, 113.7, 111.7, 111.6, 110.5, 110.4, 107.2, 106.8, and 106.7 ppm.

*2-(1H-Indol-3-yl)-7-methyl-1H-benzo[d]imidazole* (**3z**). A yield of 0.311 g (97%), 6 h, light-brown solid. Mp. = 156–157 °C (petroleum ether/EtOAc). ^1^H NMR (400 MHz, DMSO–*d*_6_) δ = 11.86 (s, 1H), 8.44–8.40 (m, 1H), 8.30 (d, *J* = 2.8 Hz, 1H), 7.58–7.54 (m, 1H), 7.45 (d, *J* = 8.0 Hz, 1H), 7.30–7.23 (m, 2H), 7.19–7.15 (m, 1H), 7.08–7.07 (m, 1H), and 2.62 (s, 3H) ppm; ^13^C{^1^H} NMR (100 MHz, DMSO–*d*_6_) δ = 147.9, 136.5, 135.9, 135.8, 128.1, 124.6, 123.6, 123.5, 123.1, 122.5, 122.5, 120.8, 120.6, 112.2, 111.2, 111.1, 103.9 (br.s), and 16.7 ppm. HRMS (ESI) calcd for C_16_H_14_N_3_ [M+H]^+^ 248.1182; found 248.1181.

*7-Bromo-5-chloro-2-(1H-indol-3-yl)-1H-benzo[d]imidazole* (**3aa**). A yield of 0.391 g (87%), 6 h, white solid. Mp. = 115–116 °C (DMAC/H_2_O). ^1^H NMR (400 MHz, DMSO–*d*_6_) δ = 12.84 (s, 1H), 11.70 (s, 1H), 8.52–8.51 (m, 1H), 8.19–8.18 (m, 1H), 7.53–7.50 (m, 2H), 7.41–7.40 (m, 1H), and 7.26–7.20 (m, 2H) ppm; ^13^C{^1^H} NMR (100 MHz, DMSO–*d*_6_) δ = 151.3, 141.8, 136.5, 135.0, 126.9, 125.7, 125.0, 123.2, 122.3, 121.2, 120.5, 111.9, 110.9, 109.7, and 105.6 ppm. HRMS (ESI) calcd for C_15_H_10_BrClN_3_ [M+H]^+^ 345.9741; found 345.9753.

*7-Bromo-2-(1H-indol-3-yl)-5-methyl-1H-benzo[d]imidazole* (**3ab**). A yield of 0.343 g (81%), 6 h, light-beige solid. Mp. = 243 °C, decomposition (EtOH). ^1^H NMR (400 MHz, DMSO–*d*_6_) δ = 12.34 (s, 1H), 8.58 (d, *J* = 2.8 Hz, 1H), 8.23–8.19 (m, 1H), 7.68–7.64 (m, 1H), 7.54 (s, 2H), 7.38–7.34 (m, 2H), and 2.50 (s, 3H) ppm; ^13^C{^1^H} NMR (100 MHz, DMSO–*d*_6_) δ = 147.6, 136.5, 136.2, 133.2, 132.2, 130.2, 130.1, 128.6, 123.7, 123.3, 121.6, 119.7, 112.9, 112.4, 104.5, 99.3, and 20.7 ppm. HRMS (ESI) calcd for C_16_H_13_BrN_3_ [M+H]^+^ 326.0287; found 326.0278.

*7-Bromo-2-(1H-indol-3-yl)-5-methoxy-1H-benzo[d]imidazole* (**3ac**). A yield of 0.253 g (57%), 8 h, gray solid. Mp. = 118 °C, decomposition (petroleum ether/EtOAc). ^1^H NMR (400 MHz, DMSO–*d*_6_) δ = 12.50 (br.s, 1H), 11.59 (s, 1H), 8.53–8.51 (m, 1H), 8.14 (br.s, 1H), 7.51–7.49 (m, 1H), 7.25–7.19 (m, 2H), 7.01–6.98 (m, 2H), and 3.83 (s, 3H) ppm; ^13^C{^1^H} NMR (100 MHz, DMSO–*d*_6_) δ = 155.5, 149.5 (br.s), 136.4, 126.2 (br.s), 125.1, 122.2, 121.3, 120.2, 112.2 (br.s), 111.8, 106.2, 94.4 (br.s), and 55.9 ppm. HRMS (ESI) calcd for C_16_H_13_BrN_3_O [M+H]^+^ 342.0237; found 342.0236.

*5,7-Dibromo-2-(1H-indol-3-yl)-1H-benzo[d]imidazole* (**3ad**). A yield of 0.437 g (86%), 6 h, light-beige solid. Mp. = 148–150 °C (DMAC/H_2_O). ^1^H NMR (400 MHz, DMSO–*d*_6_) δ = 12.82 (br.s, 1H), 11.70 (s, 1H), 8.53–8.50 (m, 1H), 8.25 (br.s, 1H), 7.68 (br.s, 1H), 7.54–7.50 (m, 2H), and 7.26–7.20 (m, 2H) ppm; ^13^C{^1^H} NMR (100 MHz, DMSO–*d*_6_) δ = 151.3 (br.s), 136.4, 127.3, 127.2, 125.7, 125.1, 122.3, 121.3, 120.5, 113.1, 111.9, and 105.5 ppm. HRMS (ESI) calcd for C_15_H_10_Br_2_N_3_ [M+H]^+^ 389.9236; found 389.9226.

*2-(1H-Indol-3-yl)-6,7-dimethyl-1H-benzo[d]imidazole* (**3ae**). A yield of 0.329 g (97%), 6.5 h, light-beige solid. Mp. = 154–155 °C (EtOAc). ^1^H NMR (400 MHz, DMSO–*d*_6_) δ = 12.07 (br.s, 1H), 11.53 (s, 1H), 8.58–8.56 (m, 1H), 8.18–8.17 (m, 1H), 7.51–7.49 (m, 1H), 7.27–7.24 (m, 1H), 7.23–7.18 (m, 2H), 6.96 (d, *J* = 8.0 Hz, 1H), 2.54 (s, 3H), and 2.35 (s, 3H) ppm; ^13^C{^1^H} NMR (100 MHz, DMSO–*d*_6_) δ = 148.8, 136.4, 127.9 (br.s), 125.7, 125.3, 123.1, 122.0, 121.5, 120.0, 111.7, 106.9, 18.9, and 13.5 ppm. HRMS (ESI) calcd for C_17_H_16_N_3_ [M+H]^+^ 262.1339; found 262.1335. (CCDC 2293846 contains the supplementary crystallographic data for this paper. These data can be obtained free of charge from the Cambridge Crystallographic Data Centre via. Available online: www.ccdc.cam.ac.uk/structures; accessed on 8 September 2023; for details, see Supplementary Materials.)

*6,7-Difluoro-2-(1H-indol-3-yl)-1H-benzo[d]imidazole* (**3af**). A yield of 0.252 g (72%), 6.5 h, gray solid. Mp. = 221–222 °C (DMAC/H_2_O). ^1^H NMR (400 MHz, DMSO–*d*_6_) δ = 12.72 (br.s, 1H), 11.66 (s, 1H), 8.51–8.47 (m, 1H), 8.18 (s, 1H), 7.54–7.50 (m, 1H), and 7.26–7.11 (m, 4H) ppm; ^13^C{^1^H} NMR (100 MHz, DMSO–*d*_6_) δ = 151.8–151.6 (m), 146.2 (d, *J*_CF_ = 9.0 Hz), 143.9 (d, *J*_CF_ = 10.0 Hz), 144.9 (d, *J*_CF_ = 258.0 Hz), 136.4, 133.7–132.8 (m), 126.8, 125.0, 122.3, 121.1, 120.4, 111.9, 110.1–109.8 (m), 105.8, and 105.6 (dd, *J*_CF_ = 12.0, 3.6 Hz) ppm. HRMS (ESI) calcd for C_15_H_10_F_2_N_3_ [M+H]^+^ 270.0837; found 270.0833.

*2-(1H-Indol-3-yl)-1-methyl-1H-benzo[d]imidazole* (**3ag**) [66]. A yield of 0.267 g (83%), 7 h, brown solid. Mp. = 230 °C, decomposition (EtOH, lit. [66] 302–303 °C). ^1^H NMR (400 MHz, DMSO–*d*_6_) δ = 11.73 (br.s, 1H), 8.38 (d, *J* = 8.0 Hz, 1H), 8.06 (d, *J* = 2.8 Hz, 1H), 7.69–7.64 (m, 1H), 7.58–7.51 (m, 2H), 7.26–7.16 (m, 4H), and 3.97 (s, 3H) ppm; ^13^C{^1^H} NMR (100 MHz, DMSO–*d*_6_) δ = 149.8, 143.1, 136.0, 135.9, 126.6, 126.3, 122.2, 121.4, 121.2, 120.0, 118.1, 111.6, 109.5, 105.1, and 31.3 ppm.

*2-(1H-Indol-3-yl)-1-isopropyl-1H-benzo[d]imidazole* (**3ah**). A yield of 0.311 g (87%), 6 h, white solid. Mp. = 257–258 °C, decomposition (DMAC/H_2_O). ^1^H NMR (400 MHz, DMSO–*d*_6_) δ = 11.68 (br.s, 1H), 7.94 (d, *J* = 8.0 Hz, 1H), 7.78–7.74 (m, 2H), 7.69–7.65 (m, 1H), 7.53 (d, *J* = 8.0 Hz, 1H), 7.24–7.13 (m, 4H), 5.07–4.96 (m, 1H), 1.63 (s, 3H), and 1.62 (d, *J* = 6.8 Hz, 3H) ppm; ^13^C{^1^H} NMR (100 MHz, DMSO–*d*_6_) δ = 148.9, 144.0, 136.0, 133.2, 126.6, 126.4, 122.0, 121.1, 121.0, 120.2, 120.0, 118.8, 112.1, 111.8, 105.1, 48.0, and 20.9 ppm. HRMS (ESI) calcd for C_18_H_18_N_3_ [M+H]^+^ 276.1495; found 276.1505.

*1-Allyl-2-(1H-indol-3-yl)-1H-benzo[d]imidazole* (**3ai**). A yield of 0.188 g (53%), 14 h, light-beige solid. Mp. = 213 °C, decomposition (petroleum ether/EtOAc). ^1^H NMR (400 MHz, DMSO–*d*_6_) δ = 11.68 (br.s, 1H), 8.36 (d, *J* = 7.6 Hz, 1H), 7.84 (br.s, 1H), 7.72–7.70 (m, 1H), 7.53–7.47 (m, 2H), 7.25–7.16 (m, 4H), 6.18–6.09 (m, 1H), 5.21 (d, *J* = 10.4 Hz, 1H), 5.07–5.05 (m, 2H), and 4.92 (d, *J* = 17.2 Hz, 1H) ppm; ^13^C{^1^H} NMR (100 MHz, DMSO–*d*_6_) δ = 149.4, 143.1, 136.0, 135.3, 133.4, 126.4, 125.9, 122.2, 121.4, 121.3, 120.1, 118.3, 116.2, 111.6, 109.8, 104.6, and 46.1 ppm. HRMS (ESI) calcd for C_18_H_16_N_3_ [M+H]^+^ 274.1339; found 274.1327.

*1-Benzyl-2-(1H-indol-3-yl)-1H-benzo[d]imidazole* (**3aj**) [67]. A yield of 0.361 g (86%), 6.5 h, white solid. Mp. = 230–231 °C (DMAC/H_2_O, lit. [67] 232–234 °C). ^1^H NMR (400 MHz, DMSO–*d*_6_) δ = 11.59 (br.s, 1H), 8.39 (d, *J* = 7.6 Hz, 1H), 7.73 (d, *J* = 8.0 Hz, 1H), 7.67 (d, *J* = 2.0 Hz, 1H), 7.49 (d, *J* = 7.6 Hz, 1H), 7.45 (d, *J* = 8.0 Hz, 1H), 7.32–7.28 (m, 2H), 7.25–7.16 (m, 5H), 7.08 (d, *J* = 7.6 Hz, 2H), and 5.73 (s, 2H) ppm; ^13^C{^1^H} NMR (100 MHz, DMSO–*d*_6_) δ = 149.7, 143.2, 137.1, 135.9, 135.6, 128.7, 127.2, 126.4, 125.9, 125.8, 122.3, 121.5, 121.6, 121.4, 120.2, 118.4, 111.6, 109.9, 104.7, and 47.0 ppm.

*2-(1H-Indol-3-yl)-1-phenyl-1H-benzo[d]imidazole* (**3ak**) [68]. A yield of 0.386 g (96%), 14 h, purple solid. Mp. = 224–225 °C (EtOH, lit. [68] 223–224 °C). ^1^H NMR (400 MHz, DMSO–*d*_6_) δ = 11.40 (br.s, 1H), 8.47–8.44 (m, 1H), 7.77 (d, *J* = 8.0 Hz, 1H), 7.70–7.63 (m, 3H), 7.56–7.52 (m, 2H), 7.45–7.41 (m, 1H), 7.30–7.26 (m, 1H), 7.22–7.16 (m, 3H), 7.05 (d, *J* = 8.0 Hz, 1H), and 6.68 (d, *J* = 2.8 Hz, 1H) ppm; ^13^C{^1^H} NMR (100 MHz, DMSO–*d*_6_) δ = 149.1, 142.5, 136.6, 136.5, 135.6, 130.2, 129.3, 128.0, 126.2, 126.0, 122.3, 122.2, 122.1, 121.6, 120.3, 118.0, 111.6, 109.5, and 104.5 ppm.

*Bis[2-(1H-indol-3-yl)-1H-benzo[d]imidazol-6-yl]methane* (**3al**). A yield of 0.590 g (95%), 6 h, gray solid. Mp. = 280 °C, decomposition (DMAC/H_2_O). ^1^H NMR (400 MHz, DMSO–*d*_6_) δ = 12.12 (s, 2H), 8.38 (s, 1H), 8.37 (s, 1H) 8.32–8.30 (m, 2H), 7.64 (d, *J* = 8.4 Hz, 2H), 7.61–7.59 (m, 4H), 7.33–7.27 (m, 6H), and 4.29 (s, 2H) ppm; ^13^C{^1^H} NMR (100 MHz, DMSO–*d*_6_) δ = 147.3, 137.5, 136.6, 134.3, 132.7, 129.8, 124.8, 123.9, 123.0, 121.2, 120.0, 113.5, 113.2, 112.7, 101.6, and 41.0 ppm. HRMS (ESI) calcd for C_31_H_24_N_6_ [M+2H]^+^ 240.1026; found 240.0974.

*2-(1H-Indol-3-yl)-1H-imidazo[4,5-b]phenazine* (**3am**). A yield of 0.261 g (60%), 9.5 h, dark-brown solid. Mp. = 330 °C, decomposition (acetone). ^1^H NMR (400 MHz, DMSO–*d*_6_) δ = 12.88 (br.s, 1H), 11.99 (s, 1H), 8.64–8.63 (m, 1H), 8.45 (s, 1H), 8.22–8.20 (m, 4H), 7.85–7.83 (m, 2H), 7.59–7.57 (m, 1H), and 7.31–7.29 (m, 2H) ppm; ^13^C{^1^H} NMR (100 MHz, DMSO–*d*_6_) δ = 157.7, 141.3, 140.4, 136.7, 129.6, 129.0, 128.7, 125.3, 122.7, 121.5, 121.1, 112.2, and 105.5 ppm. HRMS (ESI) calcd for C_21_H_14_N_5_ [M+H]^+^ 336.1244; found 336.1231.

*7-Bromo-5-methoxy-2-(2-phenyl-1H-indol-3-yl)-1H-benzo[d]imidazole* (**3an**). A yield of 0.348 g (64%), 5 h, beige solid. Mp. = 139–140 °C (petroleum ether/EtOAc). ^1^H NMR (400 MHz, DMSO–*d*_6_) δ = 12.11 (br.s, 1H), 11.84 (br.s, 1H), 7.90–7.89 (m, 1H), 7.71 -7.69 (m, 2H), 7.52–7.38 (m, 4H), 7.26–7.22 (m, 1H), 7.17–7.13 (m, 1H), 7.05 (d, *J* = 2.0 Hz, 1H), 6.95 (br.s, 1H), and 3.81 (s, 3H) ppm; ^13^C{^1^H} NMR (100 MHz, DMSO–*d*_6_) δ = 155.7, 148.0 (br.s), 137.5, 137.1 (br.s), 135.9, 135.4 (br.s), 131.6, 128.5, 128.2, 127.9 (br.s), 122.4, 120.2, 120.0, 112.8 (br.s), 111.4, 111.2 (br.s), 103.2, 94.5 (br.s), and 55.8 ppm. HRMS (ESI) calcd for C_22_H_17_BrN_3_O [M+H]^+^ 418.0550; found 418.0550.

*5-Bromo-2-(5-bromo-1H-indol-3-yl)-1H-benzo[d]imidazole* isolated as a solvate with DMAC (1:0.5DMAC) (**3ao**). A yield of 0.369 g (65%), 6 h, white solid. Mp. = 178–179 °C (DMAC/H_2_O). ^1^H NMR (400 MHz, DMSO–*d*_6_) δ = 11.94 (s, 1H), 8.63–8.62 (m, 1H), 8.25 (d, *J* = 2.4 Hz, 1H), 7.78–7.77 (m, 1H), 7.55 (d, *J* = 8.8 Hz, 1H), 7.51 (d, *J* = 8.8 Hz, 1H), and 7.38–7.32 (m, 2H) ppm; ^13^C{^1^H} NMR (100 MHz, DMSO–*d*_6_) δ = 149.5, 139.7, 139.6, 136.7, 136.6, 135.2, 128.7, 126.5, 125.0, 124.6, 123.0, 116.8 (br.s), 115.3 (br.s), 114.1, 113.9, 113.3, and 104.6 ppm. HRMS (ESI) calcd for C_15_H_10_Br_2_N_3_ [M+H]^+^ 389.9236; found 389.9225.

*2-(1-Benzyl-1H-indol-3-yl)-5-methyl-1H-benzo[d]imidazole* (**3ap**). A yield of 0.421 g (96%), 6 h, white solid. Mp. = 196–197 °C (DMAC/H_2_O). ^1^H NMR (400 MHz, DMSO–*d*_6_) δ = 8.47–8.45 (m, 1H), 8.27 (s, 1H), 7.64–7.62 (m, 1H), 7.48 (d, *J* = 8.0 Hz, 1H), 7.38–7.34 (m, 3H), 7.32–7.25 (m, 5H), 7.06 (d, *J* = 8.0 Hz, 1H), 5.56 (s, 2H), and 2.45 (s, 3H) ppm; ^13^C{^1^H} NMR (100 MHz, DMSO–*d*_6_) δ = 147.8, 137.2, 137.1, 137.0, 136.5, 135.8, 135.7, 131.3, 130.1, 128.6, 127.6, 127.3, 125.2, 123.4, 122.6, 121.2, 120.8, 113.8, 113.3, 110.8, 104.8, 49.5, and 21.2 ppm. HRMS (ESI) calcd for C_23_H_20_N_3_ [M+H]^+^ 338.1652; found 338.1643.

*2-(5-Bromo-1H-indol-3-yl)-6,7-dimethyl-1H-benzo[d]imidazole* (**3aq**). A yield of 0.415 g (94%), 5 h, light-beige solid. Mp. = 153–155 °C (DMAC/H_2_O). ^1^H NMR (400 MHz, DMSO–*d*_6_) δ = 12.10 (br.s, 1H), 11.73 (s, 1H), 8.74–8.73 (m, 1H), 8.22 (s, 1H), 7.48 (d, *J* = 8.8 Hz, 1H), 7.34 (dd, *J* = 8.8, 2.0 Hz, 1H), 7.28 (br.s, 1H), 6.96 (d, *J* = 8.0 Hz, 1H), 2.52 (s, 3H), and 2.35 (s, 3H) ppm; ^13^C{^1^H} NMR (100 MHz, DMSO–*d*_6_) δ = 148.1, 135.1, 127.0, 126.9, 124.6, 123.6, 123.3, 113.8, 112.7, 106.5, 18.9, and 13.5 ppm. HRMS (ESI) calcd for C_17_H_15_BrN_3_ [M+H]^+^ 340.0444; found 340.0450.

*7-Bromo-2-(5-bromo-1-methyl-1H-indol-3-yl)-5-methyl-1H-benzo[d]imidazole* isolated as a solvate with DMAC (1:0.3DMAC) (**3ar**). A yield of 0.529 g (91%), 6 h, light-beige solid. Mp. = 114–116 °C (DMAC/H_2_O). ^1^H NMR (400 MHz, DMSO–*d*_6_) δ = 8.69 (d, *J* = 2.0 Hz, 1H), 8.20 (s, 1H), 7.55 (d, *J* = 8.8 Hz, 1H), 7.41 (dd, *J* = 8.8, 2.0 Hz, 1H), 7.32 (s, 1H), 7.21 (s, 1H), 3.91 (s, 3H), and 2.42 (s, 3H) ppm; ^13^C{^1^H} NMR (100 MHz, DMSO–*d*_6_) δ = 148.8, 135.8, 132.4, 131.6, 127.0, 125.1, 124.8, 123.5, 113.4, 112.4, 104.8, 33.2, and 20.7 ppm. HRMS (ESI) calcd for C_17_H_14_Br_2_N_3_ [M+H]^+^ 417.9549; found 417.9534.

*2-(Benzofuran-2-yl)-1H-benzo[d]imidazole* (**3as**) [69]. A yield of 0.173 g (57%), 8 h, yellow solid. Mp. = 209–210 °C (petroleum ether/EtOAc, lit. [70] 201 °C). ^1^H NMR (400 MHz, DMSO–*d*_6_) δ = 13.22 (br.s, 1H), 7.79 (d, *J* = 7.6 Hz, 1H), 7.72–7.63 (m, 4H), 7.45–7.41 (m, 1H), 7.37–7.33 (m, 1H), and 7.28–7.24 (m, 2H) ppm; ^13^C{^1^H} NMR (100 MHz, DMSO–*d*_6_) δ = 154.4, 147.2, 143.1, 127.9, 125.7, 123.6, 122.6 (br.s), 121.9, 111.3, and 106.1 ppm.

*2-(Furan-2-yl)-1H-benzo[d]imidazole* (**3at**) [71]. A yield of 0.182 g (76%), 7 h, grey solid. Mp. = 235–237 °C (EtOAc, lit. [71] 230–232 °C). ^1^H NMR (400 MHz, DMSO–*d*_6_) δ = 12.88 (br.s, 1H), 7.93–7.92 (m, 1H), 7.58–7.54 (m, 2H), 7.22–7.18 (m, 3H), and 6.73–6.71 (m, 1H) ppm; ^13^C{^1^H} NMR (100 MHz, DMSO–*d*_6_) δ = 145.5, 144.5, 143.5, 138.9 (br.s), 122.1, 115.1 (br.s), 112.2, and 110.3 ppm.

*2-(Thiophen-2-yl)-1H-benzo[d]imidazole* (**3au**) [72]. A yield of 0.247 g (95%), 6 h, light-beige solid. Mp. = 308–309 °C (DMAC/H_2_O, lit. [72] 312–313 °C). ^1^H NMR (400 MHz, DMSO–*d*_6_) δ = 12.91 (br.s, 1H), 7.84–7.83 (m, 1H), 7.72–7.71 (m, 1H), 7.58–7.54 (m, 2H), and 7.24–7.18 (m, 3H) ppm; ^13^C{^1^H} NMR (100 MHz, DMSO–*d*_6_) δ = 146.9, 138.9 (br.s), 133.6, 128.6, 128.1, 126.6, 122.1, and 114.6 (br.s) ppm.

*2-(1-Methyl-1H-pyrrol-2-yl)-1H-benzo[d]imidazole* (**3av**) [73]. A yield of 0.177 g (69%), 12 h, light-beige solid. Mp. = 231–232 °C, decomposition (EtOH, lit. [74] 231–232 °C). ^1^H NMR (400 MHz, DMSO–*d_6_*) δ = 12.38 (br.s, 1H), 7.53–7.51 (m, 2H), 7.17–7.13 (m, 2H), 6.99–6.98 (m, 1H), 6.88–6.87 (m, 1H), 6.17–6.15 (m, 1H), and 4.11 (s, 3H) ppm; ^13^C{^1^H} NMR (100 MHz, DMSO–*d_6_*) δ = 146.4, 126.9, 122.9, 121.4, 111.2, 107.7, and 36.4 ppm.

*2-(Pyridin-4-yl)-1H-benzo[d]imidazole* (**3aw**) [65]. A yield of 0.152 g (60%), 8 h, light-beige solid. Mp. = 216–217 °C (DMAC/H_2_O, lit. [65] 218–219 °C). ^1^H NMR (400 MHz, DMSO–*d*_6_) δ = 13.19 (br.s, 1H), 8.77–8.75 (m, 2H), 8.10–8.09 (m, 2H), 7.66 (br.s, 2H), and 7.28–7.26 (m, 2H) ppm; ^13^C{^1^H} NMR (100 MHz, DMSO–*d*_6_) δ = 150.4, 148.7, 143.6 (br.s), 137.1, 123.3 (br.s), 122.1 (br.s), 120.2, and 111.8 (br.s) ppm.

*2-[3-(4-Fluorophenyl)-1-phenyl-1H-pyrazol-4-yl]-1H-benzo[d]imidazole* (**3ax**) [75]. A yield of 0.437 g (95%), 6 h, light-yellow solid. Mp. = 240 °C, decomposition (DMAC/H_2_O, lit. [75] 260–262 °C). ^1^H NMR (400 MHz, DMSO–*d*_6_) δ = 12.50 (br.s, 1H), 9.07 (s, 1H), 8.05–8.00 (m, 2H), 7.98–7.95 (m, 2H), 7.61–7.56 (m, 4H), 7.43–7.39 (m, 1H), 7.30–7.25 (m, 2H), and 7.23–7.19 (m, 2H) ppm; ^13^C{^1^H} NMR (100 MHz, DMSO–*d*_6_) δ = 162.2 (d, *J*_CF_ = 243.0 Hz), 149.4, 145.4, 138.9, 130.3 (d, *J*_CF_ = 10.0 Hz), 130.29, 129.6, 128.8 (d, *J*_CF_ = 3.0 Hz), 126.9, 121.8, 118.5, 115.0 (d, *J*_CF_ = 21.0 Hz), and 112.6 ppm.

*2-Cyclohexyl-1H-benzo[d]imidazole* (**3ay**) [76]. A yield of 0.169 g (65%), 6 h, beige solid. Mp. = 218–219 °C, decomposition (acetone/EtOAc, lit. [76] 222–224 °C). ^1^H NMR (400 MHz, DMSO–*d*_6_) δ = 7.48–7.44 (m, 2H), 7.13–7.08 (m, 2H), 2.88–2.81 (m, 1H), 2.04–2.00 (m, 2H), 1.83–1.57 (m, 5H), and 1.45–1.21 (m, 3H) ppm; ^13^C{^1^H} NMR (100 MHz, DMSO–*d*_6_) δ = 158.7, 138.4 (br.s), 120.9, 114.3 (br.s), 37.6, 31.1, 25.5, and 25.4 ppm.

*2-(1H-Indol-3-yl)benzo[d]thiazole* (**3az**) [77]. A yield of 0.284 g (87%), 16 h, beige solid. Mp. = 170–172 °C (EtOH, lit. [77] 171–172 °C). ^1^H NMR (400 MHz, DMSO–*d*_6_) δ = 11.90 (br.s, 1H), 8.42–8.38 (m, 1H), 8.25 (d, *J* = 2.8 Hz, 1H), 8.03 (d, *J* = 8.0 Hz, 1H), 7.98 (d, *J* = 8.0 Hz, 1H), 7.56–7.53 (m, 1H), 7.50–7.46 (m, 1H), 7.37–7.33 (m, 1H), 7.29–7.25 (m, 2H) ppm; ^13^C{^1^H} NMR (100 MHz, DMSO–*d*_6_) δ = 162.7, 153.7, 136.7, 133.0, 128.7, 126.0, 124.5, 124.1, 122.6, 121.6, 121.5, 121.0, 120.6, 112.2, and 110.4 ppm.

*2-(Thiophen-2-yl)benzo[d]thiazole* (**3ba**) [78]. A yield of 0.272 g (96%), 2 h, beige needles. Mp. = 97–99 °C (EtOH, lit. [78] 99–100 °C). ^1^H NMR (400 MHz, DMSO–*d*_6_) δ = 8.10–8.08 (m, 1H), 8.01–7.98 (m, 1H), 7.86–7.82 (m, 2H), 7.54–7.50 (m, 1H), 7.46–7.42 (m, 1H), and 7.25–7.23 (m, 1H) ppm; ^13^C{^1^H} NMR (100 MHz, DMSO–*d*_6_) δ = 160.7, 153.0, 136.3, 134.1, 130.6, 129.4, 128.6, 126.6, 125.4, 122.3, and 122.1 ppm.

*2-(Furan-2-yl)benzo[d]thiazole* (**3bb**) [78]. A yield of 0.249 g (95%), 2 h, beige solid. Mp. = 105–107 °C (EtOH, lit. [78] 106–107 °C). ^1^H NMR (400 MHz, DMSO–*d*_6_) δ = 8.14–8.09 (m, 1H), 8.03–7.97 (m, 2H), 7.56–7.52 (m, 1H), 7.47–7.42 (m, 1H), 7.35–7.31 (m, 1H), and 6.79–6.76 (m, 1H) ppm; ^13^C{^1^H} NMR (100 MHz, DMSO–*d*_6_) δ = 156.7, 153.2, 147.9, 146.0, 133.6, 126.6, 125.3, 122.6, 122.2, 112.9, and 111.8 ppm.

#### 3.3.3. Synthesis of 1-Methyl-2-(1-methyl-1*H*-indol-3-yl)-1*H*-benzo[*d*]imidazole (**3bc**)

To a stirred solution of 2-(1*H*-indol-3-yl)-1*H*-benzo[*d*]imidazole (**3a**) (0.7 mmol, 0.163 g) in dry DMF (4.0 mL), NaH (2.1 mmol, 60% dispersion in mineral oil, 0.084 g) was added. The reaction mixture was stirred at room temperature for 10 min, followed by the addition of MeI (2.1 mmol, 130 µL). The reaction mixture was stirred at room temperature for 5 min (TLC control). Then, the reaction mixture was poured into H_2_O (50 mL). The formed precipitate was filtered and washed with H_2_O [25]. There was a yield of 0.122 g (67%) and it was a white solid. Mp = 175–177 °C (acetone). ^1^H NMR (400 MHz, DMSO–*d*_6_) δ = 8.44–8.41 (m, 1H), 8.10 (s, 1H), 7.68–7.65 (m, 1H), 7.57–7.55 (m, 2H), 7.32–7.28 (m, 1H), 7.25–7.20 (m, 3H), 3.97 (s, 3H), and 3.93 (s, 3H) ppm; ^13^C{^1^H} NMR (100 MHz, DMSO–*d*_6_) δ = 149.5, 143.1, 136.5, 135.9, 130.6, 126.7, 122.3, 121.7, 121.2, 120.3, 118.1, 109.9, 109.5, 104.2, 32.8, and 31.3 ppm. HRMS (ESI) calcd for C_17_H_16_N_3_ [M+H]^+^ 262.1339; found 262.1350.

#### 3.3.4. Synthesis of [3-(1*H*-Benzo[*d*]imidazol-2-yl)-1*H*-indol-1-yl]phenylmethanone (**3bd**)

To a solution of 2-(1*H*-indol-3-yl)-1*H*-benzo[*d*]imidazole (**3a**) (1.0 mmol, 0.233 g) and Et_3_N (4.5 mmol, 630 µL) in *N*,*N*-dimethylacetamide (4.0 mL), benzoyl chloride (4.0 mmol, 465 µL) was added. The reaction mixture was heated at 150 °C for 4 h (TLC control). Then, the reaction mixture was poured into H_2_O (50 mL) and extracted with EtOAc (3 × 20 mL). The combined organic layers were washed with H_2_O (3 × 10 mL) and brine (10 mL), dried with Na_2_SO_4_, and concentrated to dryness. The product was purified via column chromatography on silica gel using the mixture of petroleum ether/EtOAc (gradient chromatography from 30:1 to 1:1) as the eluent. There was a yield of 0.118 g (36%) and it was a brown oil. ^1^H NMR (400 MHz, DMSO–*d*_6_) δ = 12.78 (br.s, 1H) 8.79–8.75 (m, 1H), 8.39–8.36 (m, 1H), 8.34 (s, 1H), 7.90–7.88 (m, 2H), 7.70–7.67 (m, 2H), 7.64–7.60 (m, 2H), 7.53–7.48 (m, 3H), and 7.23–7.18 (m, 2H) ppm; ^13^C{^1^H} NMR (100 MHz, DMSO–*d*_6_) δ = 168.2, 146.8, 135.9, 133.6, 132.2, 129.2, 129.1, 128.8, 128.4, 128.2, 127.9, 127.5, 125.9, 125.4, 124.4, 122.4, 121.9, 115.7, and 112.2 ppm. Calcd for C_22_H_16_N_3_O [M+H]^+^ 338.1288; found 338.1291.

#### 3.3.5. Synthesis of 3-(1*H*-Benzo[*d*]imidazol-2-yl)-1-ethyl-1*H*-indole-2-carbaldehyde (**3bf**)

To a solution of 2-(1-ethyl-1*H*-indol-3-yl)-1*H*-benzo[*d*]imidazole (**3b**) (0.4 mmol, 0.104 g) in dry THF (40 mL) cooled at -78 °C, *n*-butyllithium (0.6 mmol, 240 µL, 2.5 M in hexanes) was added dropwise. After 1 h of stirring at -78 °C, DMF (1.6 mmol, 124 µL) was added dropwise. After 4h at -78 °C, the reaction mixture was quenched by addition of saturated aqueous NH_4_Cl solution (20 mL). The aqueous phase was extracted with EtOAc (3 × 10 mL) and the organic phase was washed with H_2_O (3 × 5 mL) and brine (5 mL), dried with Na_2_SO_4_, and concentrated. The product was purified via column chromatography on silica gel using the mixture of petroleum ether/EtOAc (gradient chromatography from 30:1 to 10:1) as the eluent. There was a yield of 0.018 g (16%) and it was a yellow oil. ^1^H NMR (400 MHz, DMSO–*d*_6_) δ = 10.49 (s, 1H), 8.25–8.23 (m, 1H), 7.78–7.76 (m, 1H), 7.71–7.67 (m, 2H), 7.56–7.52 (m, 1H), 7.37–7.33 (m, 1H), 7.29–7.25 (m, 2H), 4.71 (q, *J* = 7.2 Hz, 2H), and 1.36 (t, *J* = 7.2 Hz, 3H) ppm; ^13^C{^1^H} NMR (100 MHz, DMSO–*d*_6_) δ = 185.2, 146.2, 138.5, 132.3, 127.8, 124.9, 123.3, 122.7, 122.5, 117.7, 115.7 (br.s), 111.7, 40.1, and 16.0 ppm. HRMS (ESI) calcd for C_18_H_16_N_3_O [M+H]^+^ 290.1288; found 290.1287.

### 3.4. Antimicrobial Activity

The synthesized compounds were tested for their in vitro growth inhibitory and bactericidal (fungicidal) activity against *Staphylococcus aureus* (ATCC 25923), *Staphylococcus aureus* (ATCC 43300, MRSA), *Escherichia coli* (ATCC 8739), *Escherichia coli* (ATCC 25922), *Mycobacterium smegmatis* (mc(2)155/ATCC 700084), and *Candida albicans* (ATCC 10231). Cefazolin, cefotaxime, amikacin, rifampicin, isoniazid, and fluconazole were used as control antimicrobial (antifungal) agents. The cells were grown overnight at 37 °C in a glass tube with 5 mL of LB broth containing 1% of glucose for *C. albicans* and 0.05% Tween 80 for *M. smegmatis*. The grown cultures were then diluted 1:100 with fresh medium and cultivated for 5 h. The *M. smegmatis* culture was grown for 20 h with agitation on a shaker GFL1092 (GFL, Germany) (200 rpm, 37 °C). Then, the cultures were adjusted to OD 0.1 (A625) and diluted to 1:10 for *M. smegmatis* and 1:100 for the other microorganisms. The resulting suspension was used to determine the MIC using the serial dilutions method in 96-well plates with modifications. The synthesized compounds were dissolved in DMSO at a concentration of 20 mg/mL and a series of two-fold dilutions was prepared in the same solvent. Then, 10 μL of the solutions was added to the wells of the plate containing 190 μL of the cell suspension. To the control wells, 10 µL of DMSO was added. The plates were incubated at 37 °C under static conditions for 24 h (72 h for *M. smegmatis*) for the determination of minimum inhibitory concentration. The minimum bactericidal concentration and minimum fungicidal concentration were determined as the lowest concentration of an antimicrobial agent required to achieve a 99.9% reduction in the colony-forming units (CFU) number in the initial inoculum. In total, 10 µL from each well of a plate for the MIC determination was inoculated on Petri dish with LB agar (containing 1% of glucose for *C. albicans*), and colonies were checked after incubation (24 h, 37 °C) to determine the MBC or MFC.

### 3.5. Biofilm Assay

The MBEC—the lowest concentration of substance required to kill all the bacteria in a biofilm—was determined using the Calgary system with a 96-well plate MBEC™ Biofilm Inoculator (Innovotech, Canada) [79] according to the instructions from the manufacturer. Biofilm formation was measured using the classical crystal violet test in microtiter plates [80]. The cells were grown overnight at 37 °C in a glass tube with 5 mL of LB broth. The grown culture was then adjusted to OD 0.1 (A625) and added to the wells of the plates at 190 μL. Then, 10 μL of the investigated substances solutions was added to the wells to the concentrations indicated in the figures. Plates were incubated for 24 h at 37 °C under static conditions. After that, the wells were washed twice with distilled water, stained for 30 min with 0.1% crystal violet, and washed three times. The dye was extracted with 95% ethanol over 30 min and the optical density (A570) was measured with a Tecan Infinite M200Pro (Tecan, Austria) microplate reader.

### 3.6. Molecular Docking

We obtained the predicted 3D structures of the target proteins from the AlphaFold database [81] and crystal structures from the PDB database [82]. The Protein Structure Alignment tool from Schrödinger Maestro software [83] was used for structural alignment of proteins. We conducted molecular docking using the Schrödinger Maestro software. Protein structures were preprocessed, optimized, and minimized using the Protein Preparation Wizard and OPLS3 force field. During the preparation process, we added hydrogen atoms and accounted for disulfide bonds while removing water molecules. For the ligands, we employed LigPrep to prepare them. This involved generating ionization states within a pH range of 7±2, generating tautomers of the molecules, and determining the chirality based on their three-dimensional structure. The docking process utilized the standard precision (SP) method with default parameters.

## 4. Conclusions

In summary, the cyclocondensation reaction of various phenylenediamines with aldehydes on heating in *N*,*N*-dimethylacetamide in the presence of sodium metabisulfite provided a series of 2-(1*H*-indol-3-yl)-1*H*-benzo[*d*]imidazole derivatives **3** in good to high yields. The synthesized substances were shown to have high activity against *S. aureus* including MRSA, *C. albicans*, and *M. smegmatis*. Indolylbenzo[*d*]imidazoles **3aa**, **3ad**, **3ao**, and **3aq** showed the highest antibacterial activity against staphylococci and compounds **3i** and **3ag** against *M. smegmatis*. Target compounds **3ad**, **3ag**, and **3aq** demonstrated the highest activity against *C. albicans*. 2-(5-Bromo-1*H*-indol-3-yl)-6,7-dimethyl-1*H*-benzo[*d*]imidazole (**3aq**) demonstrated the highest bactericidal activity against staphylococci cells in the biofilm. High activity against *S. aureus* and especially MRSA, including biofilm cells, as well as against *M. smegmatis* and *C. albicans* suggested a prospect for the use of these lead compounds as components of antimicrobial drugs. The molecular docking-based target identification efforts yielded three plausible interaction models for the investigated 2-(1*H*-indol-3-yl)-1*H*-benzo[*d*]imidazole derivatives **3**. These models suggest that (p)ppGpp synthetases/hydrolases, FtsZ proteins, or pyruvate kinases could potentially be involved in their mechanism of antibacterial action.

## Figures and Tables

**Table 1:** Selected antimicrobial data (MIC and MBC/MFC, µg/mL) for the indolylbenzimidazole derivatives **3** and their analogs ^a^.

Compounds	*C.a.* 10231 ^b^		*M. s.* 70084 ^c^		*E. c.* 25922 ^d^		*E. c.* 8739 ^e^		*S. a.* 25923 ^f^		MRSA ^g^	
	MIC	MFC	MIC	MBC	MIC	MBC	MIC	MBC	MIC	MBC	MIC	MBC
**3a**	15.6	15.6	125	250	- ^h^	-	500	1000	125	125	125	125
**3b**	125	125	62.5	125	>500	>500	>500	>500	31.3	31.3	62.5	62.5
**3i**	31.3	62.5	7.8	62.5	-	-	-	-	62.5	62.5	125	125
**3j**	-	-	1000	1000	-	-	-	-	15.6	62.5	125	125
**3n**	31.3	31.3	125	125	-	-	-	-	15.6	15.6	15.6	31.3
**3o**	65.5	62.5	125	125	-	-	-	-	31.3	31.3	31.3	31.3
**3q**	62.5	62.5	250	250	125	125	125	125	62.5	62.5	31.3	62.5
**3r**	125	125	250	250	125	125	125	125	125	125	125	125
**3v**	250	250	125	125	250	250	125	250	31.3	62.5	62.5	125
**3w**	62.5	62.5	62.5	62.5	125	125	125	125	62.5	62.5	31.3	62.5
**3y**	-	-	125	250	1000	-	1000	-	125	125	125	125
**3z**	62.5	62.5	125	125	250	250	250	250	62.5	62.5	62.5	125
**3aa**	15.6	31.2	125	125	-	-	-	-	3.9	15.6	7.8	15.6
**3ab**	31.3	62.5	125	125	-	-	-	-	15.6	31.2	15.6	31.2
**3ac**	62.5	62.5	250	250	-	-	-	-	31.3	31.3	31.3	62.5
**3ad**	7.8	15.6	62.5	125	-	-	-	-	7.8	7.8	3.9	7.8
**3ae**	31.3	62.5	31.3	62.5	-	-	-	-	31.3	31.3	31.3	31.3
**3af**	125	125	125	125	125	125	125	125	62.5	125	62.5	250
**3ag**	3.9	7.8	3.9	125	-	-	-	-	15.6	125	15.6	250
**3al**	-	-	1000	1000	-	-	-	-	62.5	62.5	62.5	125
**3ao**	15.6	15.6	125	125	-	-	-	-	0.98	7.8	1.98	7.8
**3aq**	3.9	7.8	125	1000	-	-	-	-	0.98	3.9	1.95	3.9
**3at**	250	250	-	-	250	-	250	-	-	-	-	-
**3au**	250	250	-	-	-	-	-	-	-	-	-	-
**3az**	15.6	15.6	62.5	250	-	-	-	-	15.6	250	31.2	125
Cefotaxime	n.d. ^i^	n.d.	n.d.	n.d.	0.038	0.038	0.038	0.038	0.31	0.61	19.53	39.06
Cefazolin	n.d.	n.d.	n.d.	n.d.	2.44	2.44	2.44	2.44	0.15	0.61	9.77	39.06
Amikacin	n.d.	n.d.	n.d.	n.d.	19.53	19.53	19.53	19.53	4.88	9.77	9.77	9.77
Fluconazole	1.94	7.8	n.d.	n.d.	n.d.	n.d.	n.d.	n.d.	n.d.	n.d.	n.d.	n.d.
Isoniazid	n.d.	n.d.	4.58	9.16	n.d.	n.d.	n.d.	n.d.	n.d.	n.d.	n.d.	n.d.
Rifampicin	n.d.	n.d.	1.22	19.53	n.d.	n.d.	n.d.	n.d.	n.d.	n.d.	n.d.	n.d.

^a^ In the table, the Mode values from 3–6 independent experiments are presented. MIC—minimum inhibitory concentration; MBC—minimum bactericidal concentration; MFC—minimum fungicidal concentration; ^b^ *Candida albicans* ATCC 10231; ^c^ *Mycobacterium smegmatis* ATCC 70084; ^d^ *Escherichia coli* ATCC 25922; ^e^ *Escherichia coli* ATCC 8739; ^f^ *Staphylococcus aureus* ATCC 25923; ^g^ *Staphylococcus aureus* ATCC 43300 (MRSA); ^h^ (-) >1000 µg/mL; ^i^ n.d.—not determined.

**Table 2:** Antibiofilm activity data (MBEC ^a^, µg/mL) for the selected indolylbenzo[*d*]imidazoles ^b^.

Compounds	*C. albicans* ATCC 10231	*S. aureus* ATCC 25923	MRSA
**3aa**	125	250	250
**3ad**	62.5	125	125
**3ao**	125	125	125
**3aq**	62.5	62.5	125

^a^ Minimal biofilm eradication concentration; ^b^ the table presents the mode values from 3–4 independent experiments.

**Table 3:** The docking results for interaction of compounds **3** with (p)ppGpp synthetases/hydrolases from different bacterial species presented as docking scores (kcal/mol).

Compounds	*E. coli* RelA (AF)	*S. aureus* Rel (AF)	*M. smegmatis* Rel (AF)	Compounds	*E. coli* RelA (AF)	*S. aureus* Rel (AF)	*M. smegmatis* Rel (AF)
**3a**	−5.044	−4.350	−5.306	**3y**	−3.650	−3.593	−3.528
**3b**	−4.941	−4.279	−5.615	**3z**	−4.573	−3.907	−3.992
**3c**	−4.937	−4.304	−4.402	**3aa**	−4.337	−4.085	−4.953
**3d**	−5.058	−4.277	−4.722	**3ab**	−4.328	−3.940	−3.956
**3e**	−4.673	−3.836	−5.021	**3ac**	−4.395	−3.854	−4.141
**3f**	−5.532	−4.298	−4.259	**3ad**	−4.143	−4.052	−4.525
**3g**	−4.547	−4.486	−4.914	**3ae**	−4.079	−3.984	−4.321
**3h**	−4.394	−3.833	−4.629	**3af**	−5.081	−4.601	−5.327
**3i**	−5.181	−4.308	−4.485	**3ag**	−4.694	−4.200	−3.808
**3j**	−4.485	−4.281	−4.313	**3ah**	−4.698	−3.483	−4.638
**3k**	−3.864	−4.241	−4.405	**3ai**	−3.982	−3.554	−4.201
**3l**	−4.631	−3.946	−5.017	**3aj**	−4.823	−3.835	−4.589
**3m**	−4.506	−4.149	−4.362	**3ak**	−4.348	−3.797	−4.209
**3n**	−4.422	−4.116	−4.893	**3al**	−4.559	−2.974	−3.484
**3o**	−4.729	−4.433	−5.199	**3am**	−4.854	−4.112	−5.176
**3p**	−4.497	−4.171	−4.094	**3an**	−4.154	−3.660	−3.548
**3q**	−4.904	−4.175	−4.724	**3ao**	−3.837	−3.847	−4.405
**3r**	−4.288	−4.294	−4.948	**3ap**	−3.569	−3.846	−3.898
**3s**	−4.204	−3.921	−4.595	**3aq**	−4.566	−3.816	−4.095
**3t**	−4.778	−4.328	−4.591	**3ar**	−3.409	−2.410	−2.760
**3u**	−4.563	−4.719	−5.054	**3az**	−5.005	−3.927	−5.166
**3v**	−4.696	−3.701	−4.011	**3bd**	−6.101	−4.656	−4.254
**3w**	−4.346	−3.993	−4.244	**3bf**	−5.454	−4.274	−4.784
**3x**	−4.619	−4.234	−5.299	relacin ^a^	−6.757	−6.880	−7.457
DMNP ^a,b^ (COO—)	−3.898	−4.612	−3.309	indole-5-carboxylic acid ^a^ (I2) (COO—)	−5.461	−5.883	−5.283

^a^ Reference ligand; ^b^ DMNP−4-(4,7-dimethyl-1,2,3,4-tetrahydronaphthalen-1-yl)pentanoic acid.

**Table 4:** The docking results for interaction of compounds with pyruvate kinases and FtsZ proteins from different bacterial species presented as docking scores (kcal/mol).

	Pyruvate Kinase				FtsZ		
Compounds	*C. albicans* (AF)	*E. coli* (PDB 1PKY)	*S. aureus* (PDB 3T0T)	*M. smegmatis* (AF)	*E. coli* (AF)	*S. aureus* FtsZ (AF)	*M. smegmatis* (AF)
**3a**	−5.803	−6.356	−8.309	−3.881	−6.178	−5.650	−5.944
**3n**	−5.095	−6.303	−7.491	−4.317	−5.996	−4.969	−5.614
**3o**	−5.591	−6.255	−7.751	−4.843	−6.089	−5.197	−5.765
**3b**	−5.376	−7.073	−8.194	−2.248	−5.488	−5.573	−5.538
**3c**	−5.479	−3.345	−8.277	−4.776	−4.593	−5.008	−5.579
**3d**	−5.516	−6.797	−8.508	−5.982	−4.982	−5.198	−5.488
**3g**	−5.287	−6.462	−8.841	−3.059	−5.407	−4.639	−5.677
**3f**	−6.058	−3.848	−7.638	−3.480	−5.186	−4.240	−4.881
**3q**	−5.452	−5.591	−8.257	−2.944	−5.971	−4.877	−5.694
**3r**	−5.177	−5.529	−7.835	−3.084	−5.703	−4.939	−5.492
**3s**	−5.057	−5.598	−7.974	−3.998	−5.685	−4.922	−5.448
**3y**	−4.361	−5.915	−6.917	−3.147	−5.361	−4.471	−4.852
**3u**	−4.524	−7.168	−8.628	−4.140	−6.546	−5.452	−5.694
**3v**	−4.624	−5.391	−7.663	−2.867	−5.561	−4.858	−5.098
**3ae**	−5.288	−5.856	−7.707	−3.257	−5.602	−5.155	−5.691
**3t**	−5.443	−6.320	−8.256	−3.528	−6.209	−5.556	−5.704
**3w**	−5.358	−5.929	−8.052	−3.051	−5.665	−4.918	−5.534
**3h**	−6.084	−4.775	−7.679	−2.959	−5.078	−4.497	−5.073
**3i**	−4.606	−3.271	−7.804	−3.009	−4.595	−4.703	−5.015
**3al**	−3.258	−2.866	−5.359	n.d. ^a^	n.d.	−2.465	−4.450
**3ag**	−5.493	−7.666	−8.086	−2.382	−5.552	−5.756	−6.139
**3x**	−6.043	−6.111	−8.326	−3.543	−6.297	−5.178	−6.116
**3ab**	−4.610	−6.001	−7.703	−3.223	−6.129	−5.334	−5.640
**3ac**	−5.317	−5.406	−7.721	−4.100	−6.052	−5.182	−5.551
**3aa**	−5.302	−5.352	−7.314	−4.012	−6.220	−5.405	−5.772
**3ao**	−4.632	−5.325	−7.520	−2.945	−6.060	−4.931	−5.243
**3z**	−5.180	−5.759	−7.497	−2.915	−5.708	−5.119	−5.256
**3ap**	−5.011	−8.114	−8.184	−2.191	−5.035	−4.565	−4.203
**3af**	−5.182	−5.211	−8.506	−4.393	−6.308	−5.146	−6.087
**3j**	−4.627	−5.168	−7.640	−2.895	−5.004	−4.315	−4.970
**3aq**	−5.470	−1.811	−7.425	−3.099	−5.686	−4.736	−5.279
**3ad**	−5.126	−5.177	−7.927	−1.929	−5.092	−4.522	−5.348
**3p**	−5.063	−6.641	−7.668	−2.930	−4.833	−5.478	−4.828
**3az**	−6.224	−6.985	−7.912	−3.907	−5.315	−5.263	−5.647
Reference ligands							
GTP ^b^	-	-	-	-	−7.548	−7.705	−7.385
IS-130 ^c^	−3.179	−2.543	−6.933	−3.488	-	-	-

^a^ n.d.—not determined; ^b^ GTP—guanosine triphosphate; ^c^ IS-130—*N*’-[(1*E*)-1-(1*H*-benzimidazol-2-yl)ethylidene]-5-bromo-2-hydroxybenzohydrazide.

## Data Availability

The supporting information includes NMR spectra and HRMS charts.

## Supplementary Materials

The following supporting information can be downloaded at https://www.mdpi.com/article/10.3390/molecules28207095/s1, Table S1: Antimicrobial data (MIC and MBC/MFC, µg/mL) for the indolylbenzimidazole derivatives and their analogs **3**; Figure S1: Influence of sublethal concentrations of amikacin on the *S. aureus* ATCC 25923 biofilm biomass (OD570) and the number of colony-forming units (CFU) in plankton. Diagrams show the mean values of three experiments; copies of ^1^H, ^13^C NMR spectra of target compounds; copies of HRMS of new compounds. Table S2: Experimental details for 2-(1*H*-indol-3-yl)-6,7-dimethyl-1*H*-benzo[*d*]imidazole (**3ae**, CCDC 2293846); Figure S2. Structure of 2-(1*H*-indol-3-yl)-6,7-dimethyl-1*H*-benzo[*d*]imidazole (**3ae**, CCDC 2293846) according to the X-ray diffraction data; non-hydrogen atoms are shown as thermal vibration ellipsoids with a probability of 50%.

## Sample Availability

Samples of synthesized compounds are available from the authors.
