# Supplementary material for: Synthesis, Antimicrobial and Antibiofilm Activities, and Molecular Docking Investigations of 2-(1H-Indol-3-yl)-1H-benzo[d]imidazole Derivatives
Source: Molecules. 2023 Oct 14;28(20):7095. doi: 10.3390/molecules28207095 (PMC10609029; doi:10.3390/molecules28207095)
Supplement: Supplementary file 1 [file molecules-28-07095-s001.zip › molecules-2657091-supplementary.pdf]

Synthesis, Antimicrobial and Antibiofilm Activities, and Molecular Docking Investigations of 2-(1*H*-Indol-3-yl)-1*H*-benzo[*d*]imidazole Derivatives

Elena Y. Mendogralo [1,*], Larisa Y. Nesterova [2,3], Ekaterina R. Nasibullina [1], Roman O. Shcherbakov [1], Danil A. Myasnikov [1], Alexander G. Tkachenko [2,3], Roman Y. Sidorov [1,3] and Maxim G. Uchuskin [1]

[1] Department of Chemistry, Perm State University, Bukireva St. 15, 614990 Perm, Russia; kat.nasibullina@yandex.ru (E.R.N.); romanshcherbakov00@gmail.com (R.O.S.); mda@psu.ru (D.A.M.); sidorov.r@iegm.ru (R.Y.S.); mu@psu.ru (M.G.U.)
[2] Department of Biology, Perm State University, Bukireva St. 15, 614990 Perm, Russia; larisa.nesterova@bk.ru (L.Y.N.); agtkachenko@iegm.ru (A.G.T.)
[3] Institute of Ecology and Genetics of Microorganisms, Perm Federal Research Center, the Ural Branch of Russian Academy of Sciences, Goleva St. 13, 614081 Perm, Russia
* Correspondence: zelina.e@psu.ru

Supporting information

Table of contents

1. Antimicrobial data (MIC and MBC/MFC, μg/mL) for the indolylbenzimidazole derivatives and their analogs **3**……………………..…………………………………………………….……….S2
2. Diagram of the effect of sublethal concentrations of amikacin on the biomass of *S. aureus* ATCC 25923 (OD570) biofilm and the number of colony forming units (CFU) in plankton…………......S4
3. Copies of ^{1}H, ^{13}C NMR spectra of target compounds…………………………………..…………...S5
4. Copies of HRMS of new compounds…………..…………………………………..……...….S62
5. X-ray crystallography data……………………………………………………………….......S76

Table S1: Antimicrobial data (MIC and MBC/MFC, μg/mL) for the indolylbenzimidazole derivatives and their analogs **3** [a]

**Compounds**	***C.a.* 10231** [b]		***M. s.* 70084** [c]		***E. c.* 25922** [d]		***E. c.* 8739** [e]		***S. a.* 25923** [f]		**MRSA** [g]	
	**MIC**	**MFC**	**MIC**	**MBC**	**MIC**	**MBC**	**MIC**	**MBC**	**MIC**	**MBC**	**MIC**	**MBC**
**3a**	15.6	15.6	125	250	- [h]	-	500	1000	125	125	125	125
**3b**	125	125	62.5	125	>500	>500	>500	>500	31.3	31.3	62.5	62.5
**3c**	125	125	-	-	-	-	-	-	-	-	-	-
**3d**	62.5	125	-	-	-	-	-	-	500	500	1000	1000
**3e**	-	-	-	-	-	-	-	-	-	-	-	-
**3f**	62.5	62.5	-	-	-	-	-	-	-	-	-	-
**3g**	250	250	-	-	-	-	-	-	-	-	-	-
**3h**	15.6	250	125	125	-	-	-	-	31.3	62.5	62.5	125
**3i**	31.3	62.5	7.8	62.5	-	-	-	-	62.5	62.5	125	125
**3j**	-	-	1000	1000	-	-	-	-	15.6	62.5	125	125
**3k**	-	-	-	-	-	-	-	-	-	-	-	-
**3l**	-	-	-	-	-	-	-	-	-	-	-	-
**3m**	-	-	1000	1000	-	-	-	-	-	-	-	-
**3n**	31.3	31.3	125	125	-	-	-	-	15.6	15.6	15.6	31.3
**3o**	65.5	62.5	125	125	-	-	-	-	31.3	31.3	31.3	31.3
**3p**	15.6	15.6	-	-	-	-	-	-	15.6	31.6	15.6	62.5
**3q**	62.5	62.5	250	250	125	125	125	125	62.5	62.5	31.3	62.5
**3r**	125	125	250	250	125	125	125	125	125	125	125	125
**3s**	65.5	62.5	250	250	-	-	-	-	31.3	62.5	31.3	31.3
**3t**	31.3	31.3	31.3	62.5	-	-	-	-	31.3	31.3	31.3	31.3
**3u**	31.3	62.5	500	500	-	-	-	-	31.3	31.3	31.3	31.3
**3v**	250	250	125	125	250	250	125	250	31.3	62.5	62.5	125
**3w**	62.5	62.5	62.5	62.5	125	125	125	125	62.5	62.5	31.3	62.5
**3x**	15.6	31.25	250	250	-	-	-	-	15.6	31.2	15.6	31.2
**3y**	-	-	125	250	1000	-	1000	-	125	125	125	125
**3z**	62.5	62.5	125	125	250	250	250	250	62.5	62.5	62.5	125
**3aa**	15.6	31.2	125	125	-	-	-	-	3.9	15.6	7.8	15.6
**3ab**	31.3	62.5	125	125	-	-	-	-	15.6	31.2	15.6	31.2
**3ac**	62.5	62.5	250	250	-	-	-	-	31.3	31.3	31.3	62.5
**3ad**	7.8	15.6	62.5	125	-	-	-	-	7.8	7.8	3.9	7.8
**3ae**	31.3	62.5	31.3	62.5	-	-	-	-	31.3	31.3	31.3	31.3
**3af**	125	125	125	125	125	125	125	125	62.5	125	62.5	250
**3ag**	3.9	7.8	3.9	125	-	-	-	-	15.6	125	15.6	250
**3ah**	-	-	-	-	-	-	-	-	-	-	-	-
**3ai**	-	-	-	-	-	-	-	-	-	-	-	-
**3aj**	-	-	-	-	-	-	-	-	-	-	-	-
**3ak**	-	-	-	-	-	-	-	-	-	-	-	-
**3al**	-	-	1000	1000	-	-	-	-	62.5	62.5	62.5	125
**3am**	-	-	-	-	-	-	-	-	-	-	-	-
**3an**	-	-	250	250	-	-	-	-	-	-	-	-
**3ao**	15.6	15.6	125	125	-	-	-	-	0.98	7.8	1.98	7.8
**3ap**	-	-	62.5	62.5	-	-	-	-	1000	1000	250	250
**3aq**	3.9	7.8	125	1000	-	-	-	-	0.98	3.9	1.95	3.9
**3ar**	-	-	-	-	-	-	-	-	-	-	-	-
**3as**	62.5	62.5	-	-	-	-	-	-	62.5	125	62.5	62.5
**3at**	250	250	-	-	250	-	250	-	-	-	-	-
**3au**	250	250	-	-	-	-	-	-	-	-	-	-
**3av**	-	-	-	-	-	-	-	-	-	-	-	-

**3aw**	125	125	500	1000	500	1000	500	1000	1000	1000	1000	1000
**3ax**	-	-	-	-	-	-	-	-	-	-	-	-
**3ay**	62.5	62.5	500	500	-	-	-	-	-	-	-	-
**3az**	15.6	15.6	62.5	250	-	-	-	-	15.6	250	31.2	125
**3ba**	-	-	-	-	-	-	-	-	-	-	-	-
**3bb**	-	-	-	-	-	-	-	-	-	-	-	-
Cefotaxime	n.d. [i]	n.d.	n.d.	n.d.	0.038	0.038	0.038	0.038	0.31	0.61	19.53	39.06
Cefazolin	n.d.	n.d.	n.d.	n.d.	2.44	2.44	2.44	2.44	0.15	0.61	9.77	39.06
Amikacin	n.d.	n.d.	n.d.	n.d.	19.53	19.53	19.53	19.53	4.88	9.77	9.77	9.77
Fluconazole	1.94	7.8	n.d.	n.d.	n.d.	n.d.	n.d.	n.d.	n.d.	n.d.	n.d.	n.d.
Isoniazid	n.d.	n.d.	4.58	9.16	n.d.	n.d.	n.d.	n.d.	n.d.	n.d.	n.d.	n.d.
Rifampicin	n.d.	n.d.	1.22	19.53	n.d.	n.d.	n.d.	n.d.	n.d.	n.d.	n.d.	n.d.

[a] In the table, the Mode values from 3-6 independent experiments are presented. MIC – minimum inhibitory concentration; MBC – minimum bactericidal concentration; MFC – minimum fungicidal concentration; [b] *Candida albicans* ATCC 10231; [c] *Mycobacterium smegmatis* ATCC 70084; [d] *Escherichia coli* ATCC 25922; [e] *Escherichia coli* ATCC 8739; [f] *Staphylococcus aureus* ATCC 25923; [g] *Staphylococcus aureus* ATCC 43300 (MRSA); [h] (-) >1000 μg/mL; [i] n.d. – not determined.

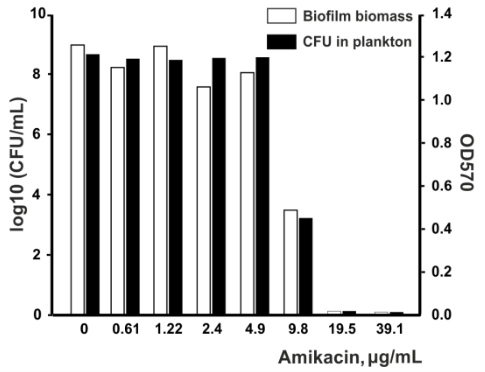

Figure S1: Influence of sublethal concentrations of amikacin on the *S. aureus* ATCC 25923 biofilm biomass (OD570) and the number of colony-forming units (CFU) in plankton. Diagrams show the mean values of three experiments.

Copies of ¹H, ¹³C NMR spectra of target compounds

¹H, DMSO-d₆, 400 MHz

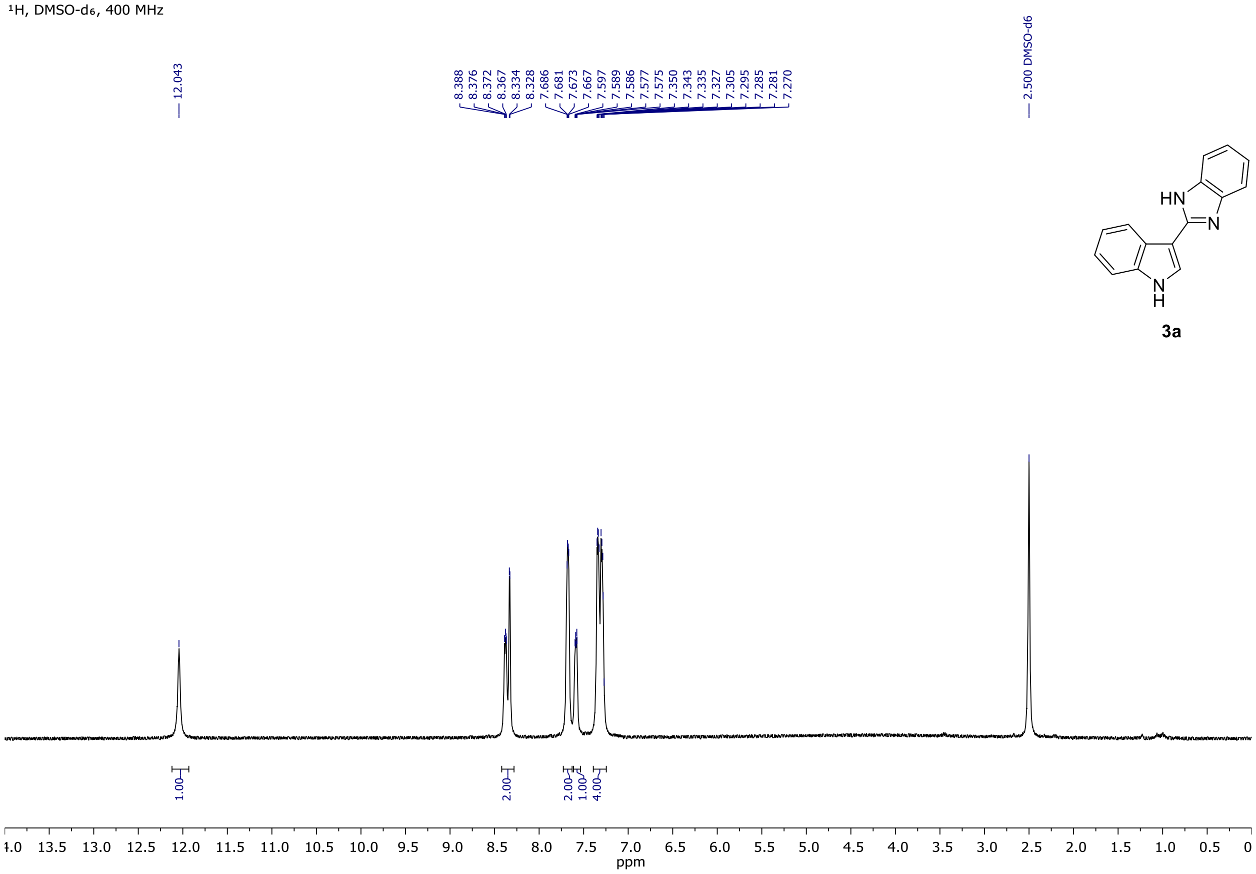

¹³C{¹H}, DMSO-d₆, 100 MHz

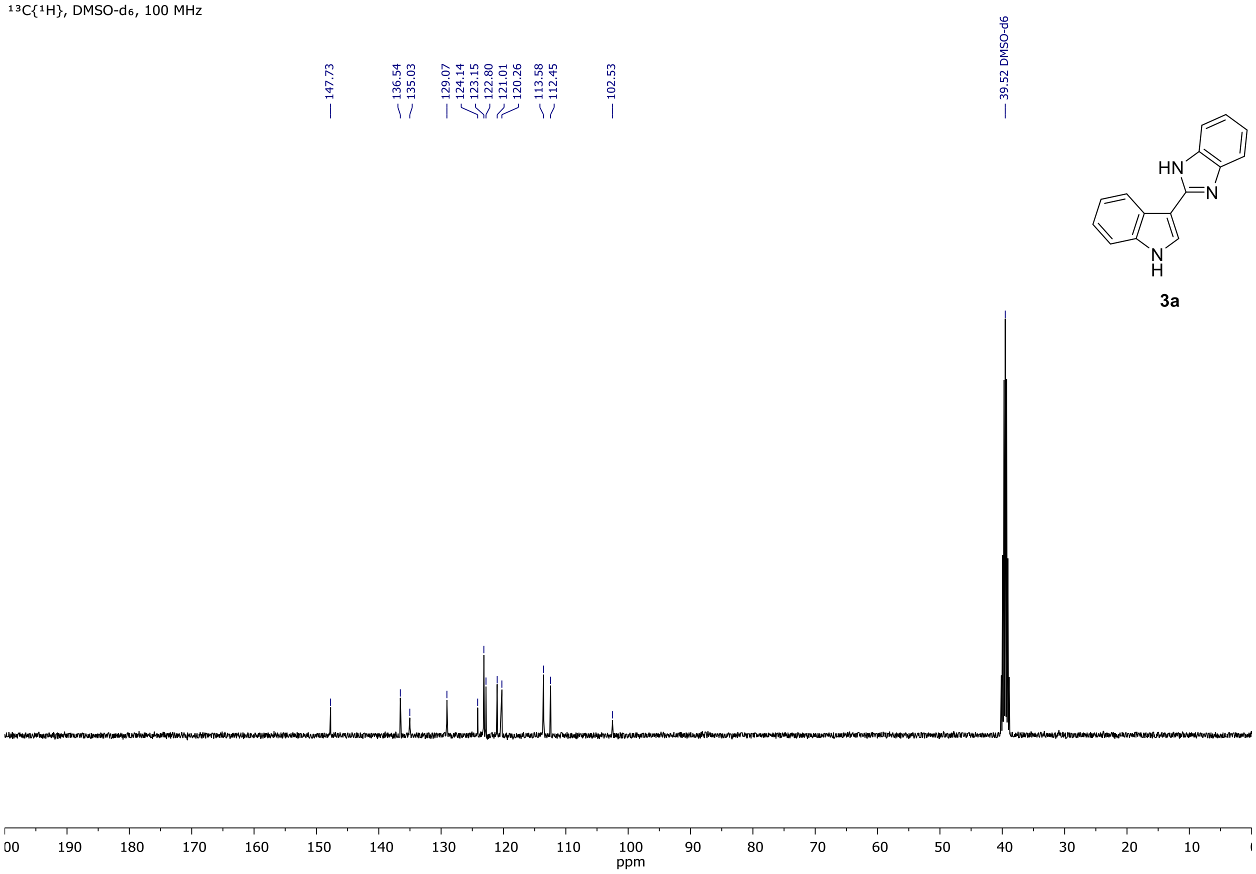

¹H, DMSO-d₆, 400 MHz

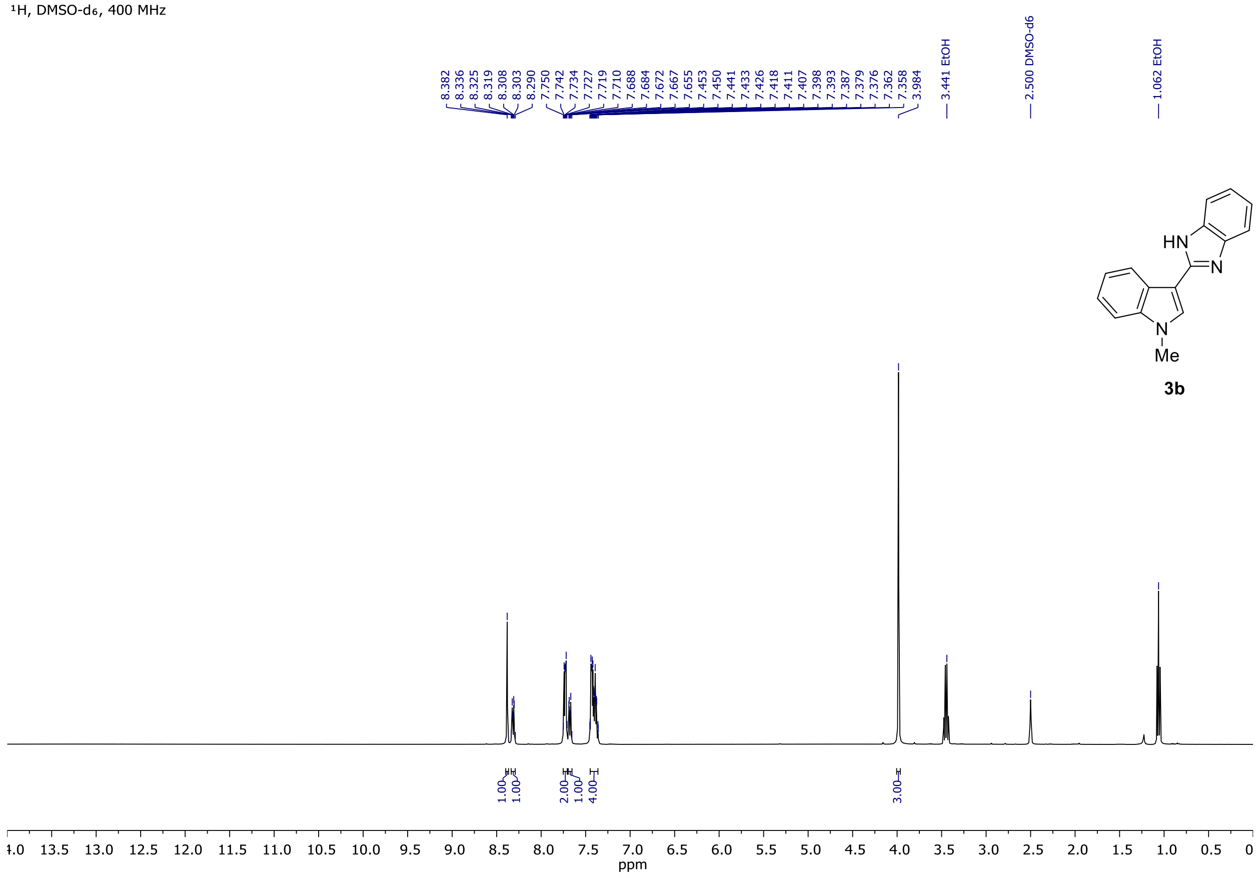

¹³C{¹H}, DMSO-d₆, 100 MHz

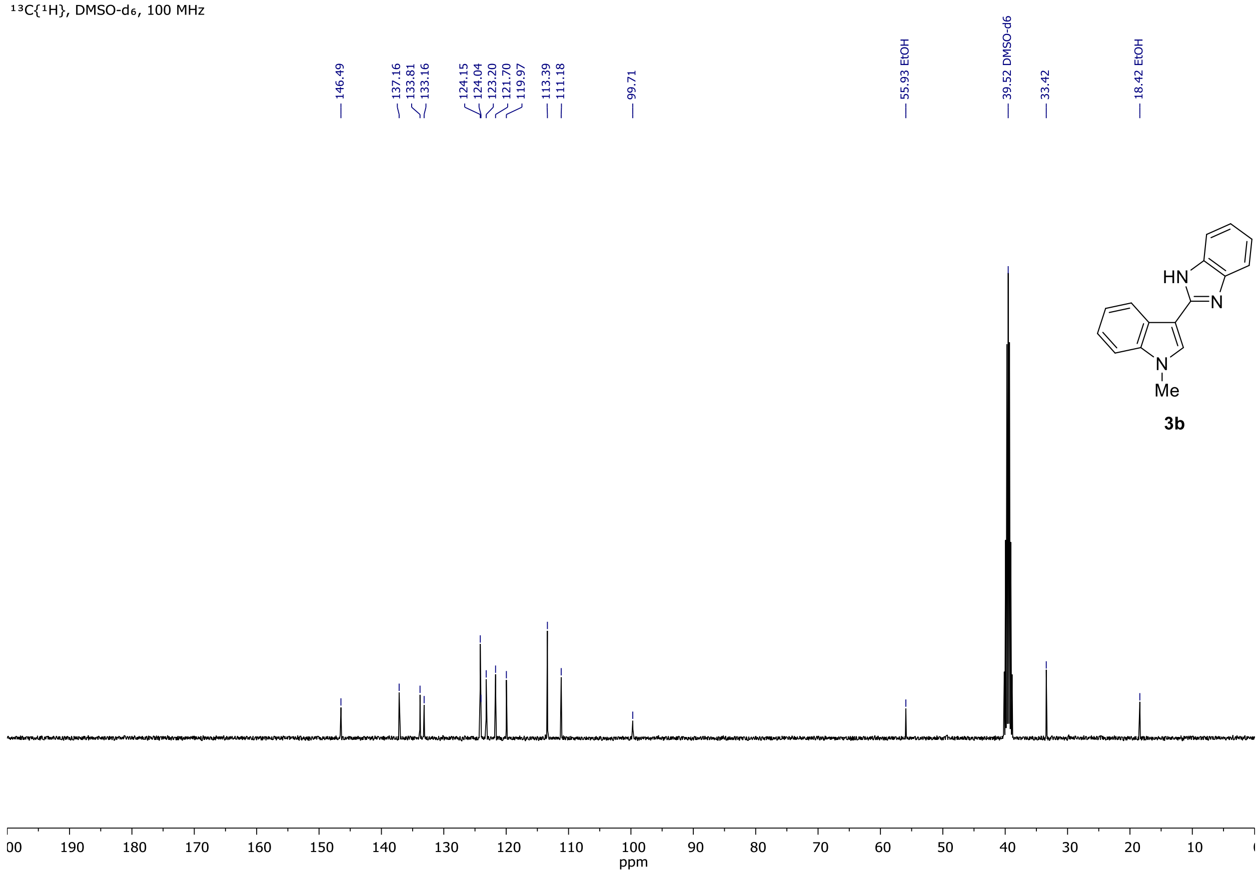

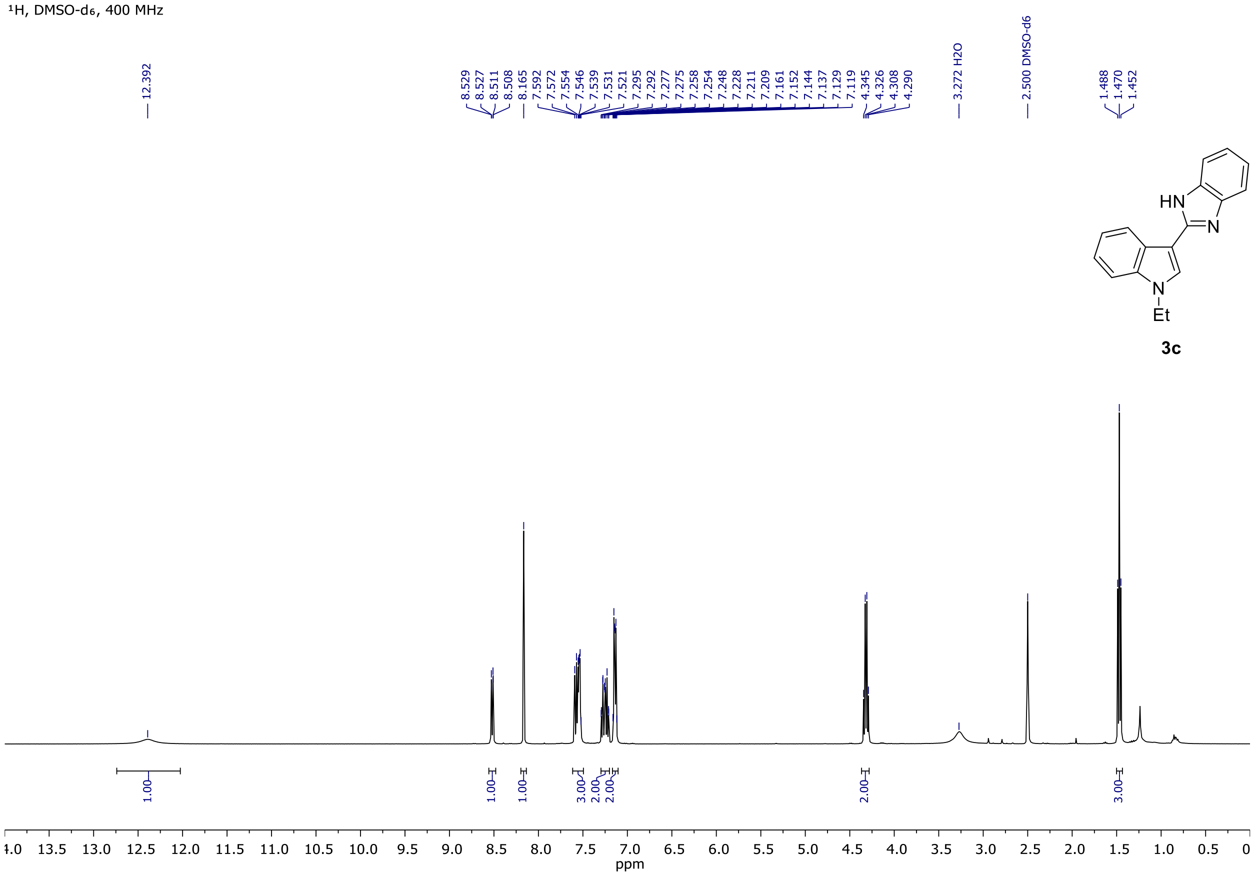
¹H, DMSO-d₆, 400 MHz
12.392
8.529
8.527
8.511
8.508
8.165
7.592
7.572
7.554
7.546
7.539
7.531
7.521
7.295
7.292
7.277
7.275
7.258
7.254
7.248
7.228
7.211
7.209
7.161
7.152
7.144
7.137
7.129
7.119
4.345
4.326
4.308
4.290
3.272 H2O
2.500 DMSO-d6
1.488
1.470
1.452
HN
N
N
Et
3c
1.00
1.00
1.00
3.00
2.00
2.00
2.00
3.00
ppm

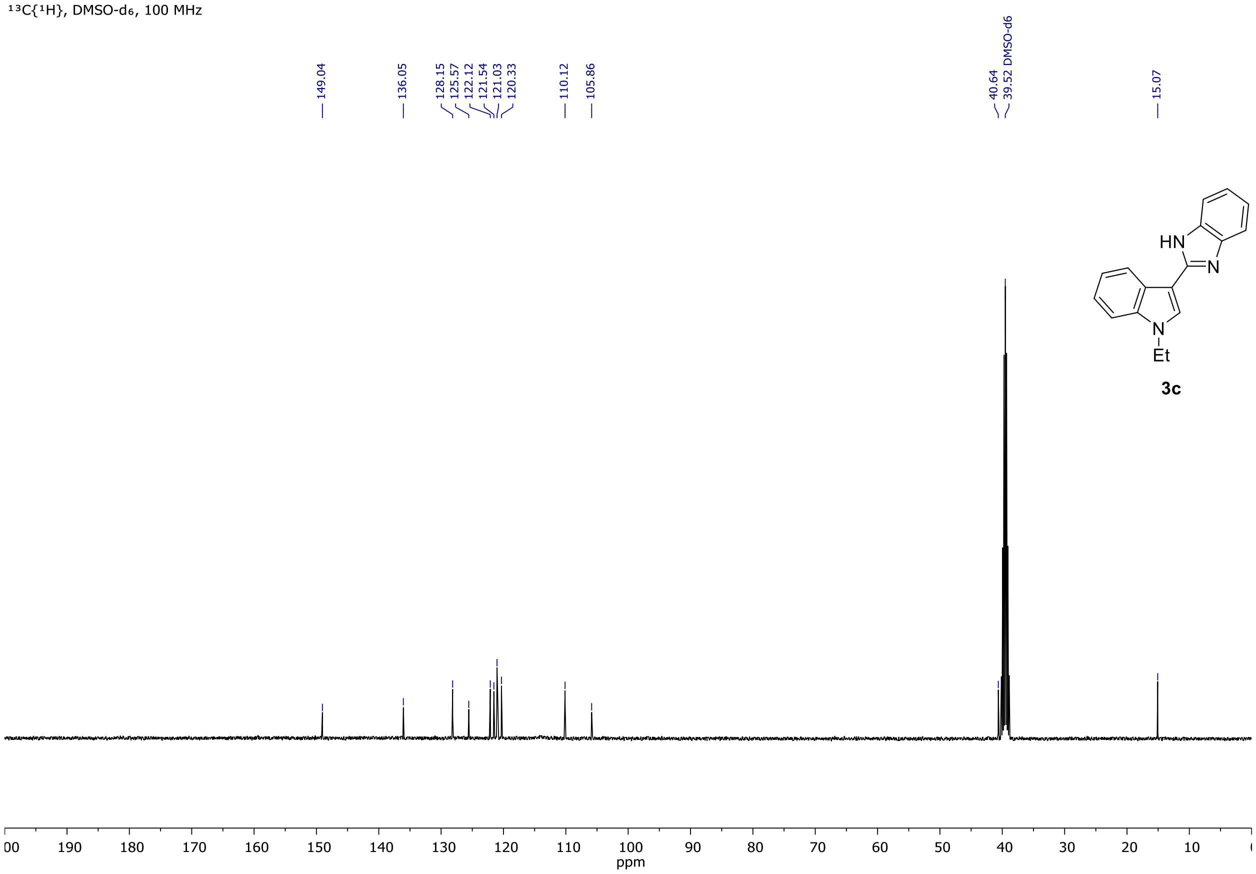
¹³C{¹H}, DMSO-d₆, 100 MHz
149.04
136.05
128.15
125.57
122.12
121.54
121.03
120.33
110.12
105.86
40.64
39.52 DMSO-d6
15.07
HN
N
N
Et
3c
ppm

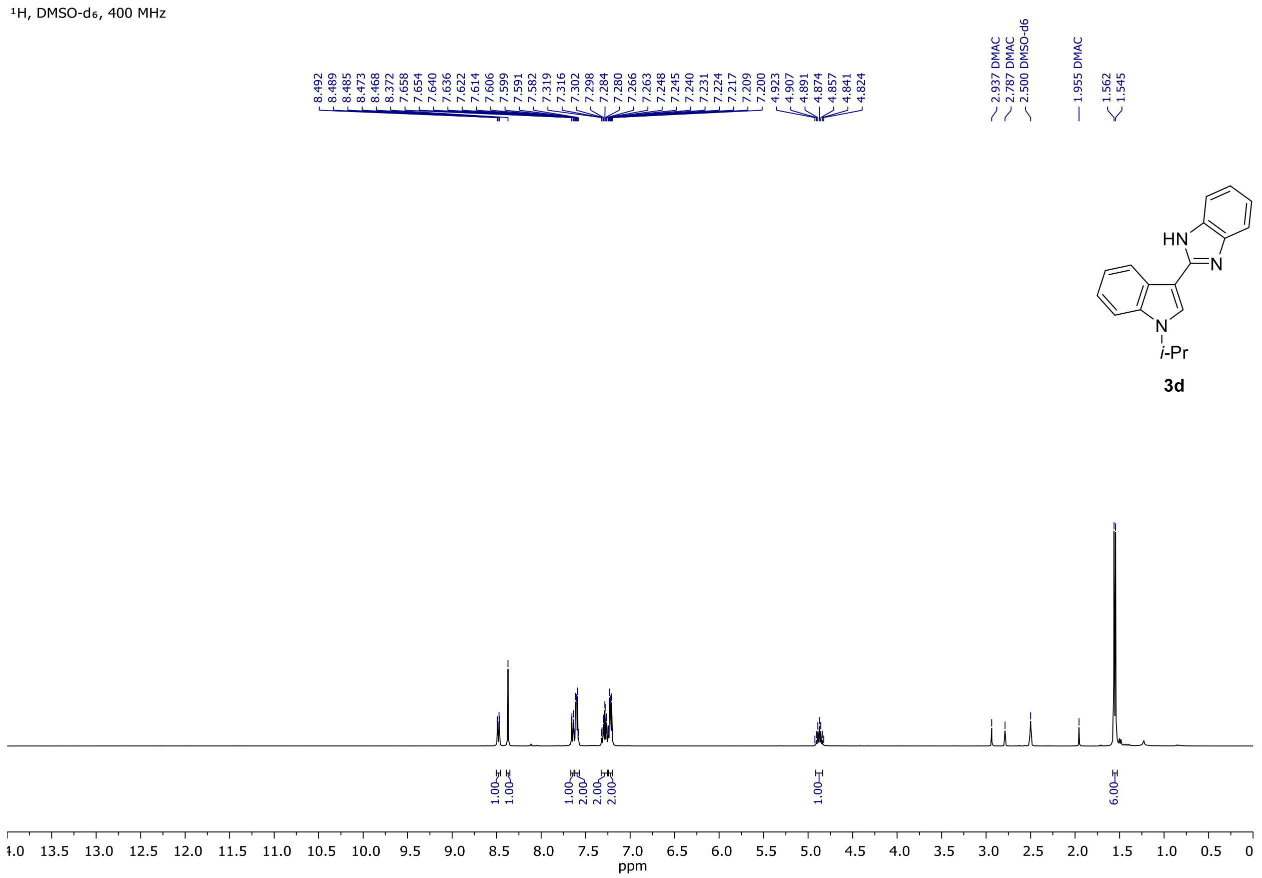
¹H, DMSO-d₆, 400 MHz
8.492 8.489 8.485 8.473 8.468 8.372 7.658 7.654 7.640 7.636 7.622 7.614 7.606 7.599 7.591 7.582 7.319 7.316 7.302 7.298 7.284 7.280 7.266 7.263 7.248 7.245 7.240 7.231 7.224 7.217 7.209 7.200 4.923 4.907 4.891 4.874 4.857 4.841 4.824
2.937 DMAC
2.787 DMAC
2.500 DMSO-d6
1.955 DMAC
1.562 1.545
HN
N
N
i-Pr
3d
1.00 1.00 1.00 2.00 2.00 2.00 1.00 6.00
ppm

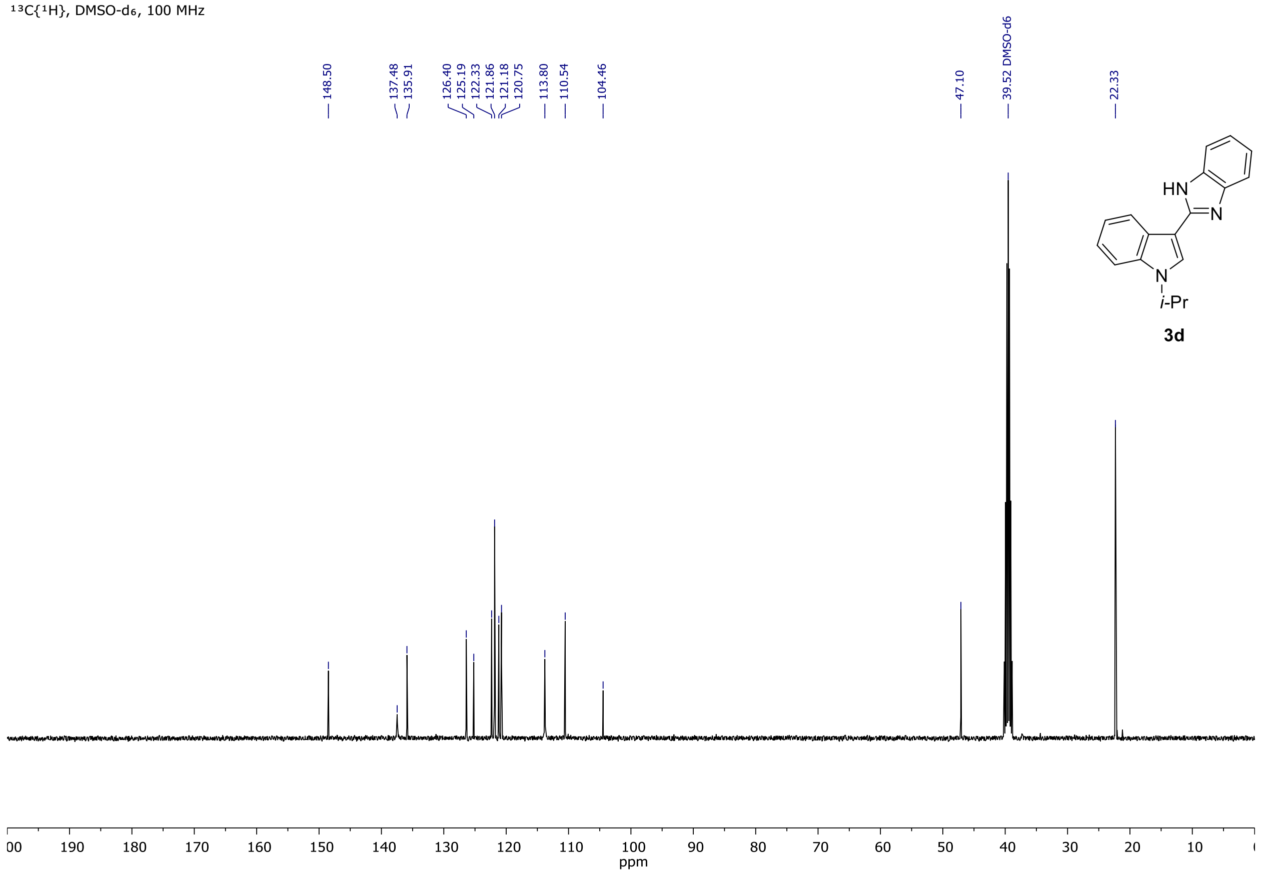
¹³C{¹H}, DMSO-d₆, 100 MHz
148.50 137.48 135.91 126.40 125.19 122.33 121.86 121.18 120.75 113.80 110.54 104.46 47.10
39.52 DMSO-d6
22.33
HN
N
N
i-Pr
3d
ppm

¹H, DMSO-d₆, 400 MHz

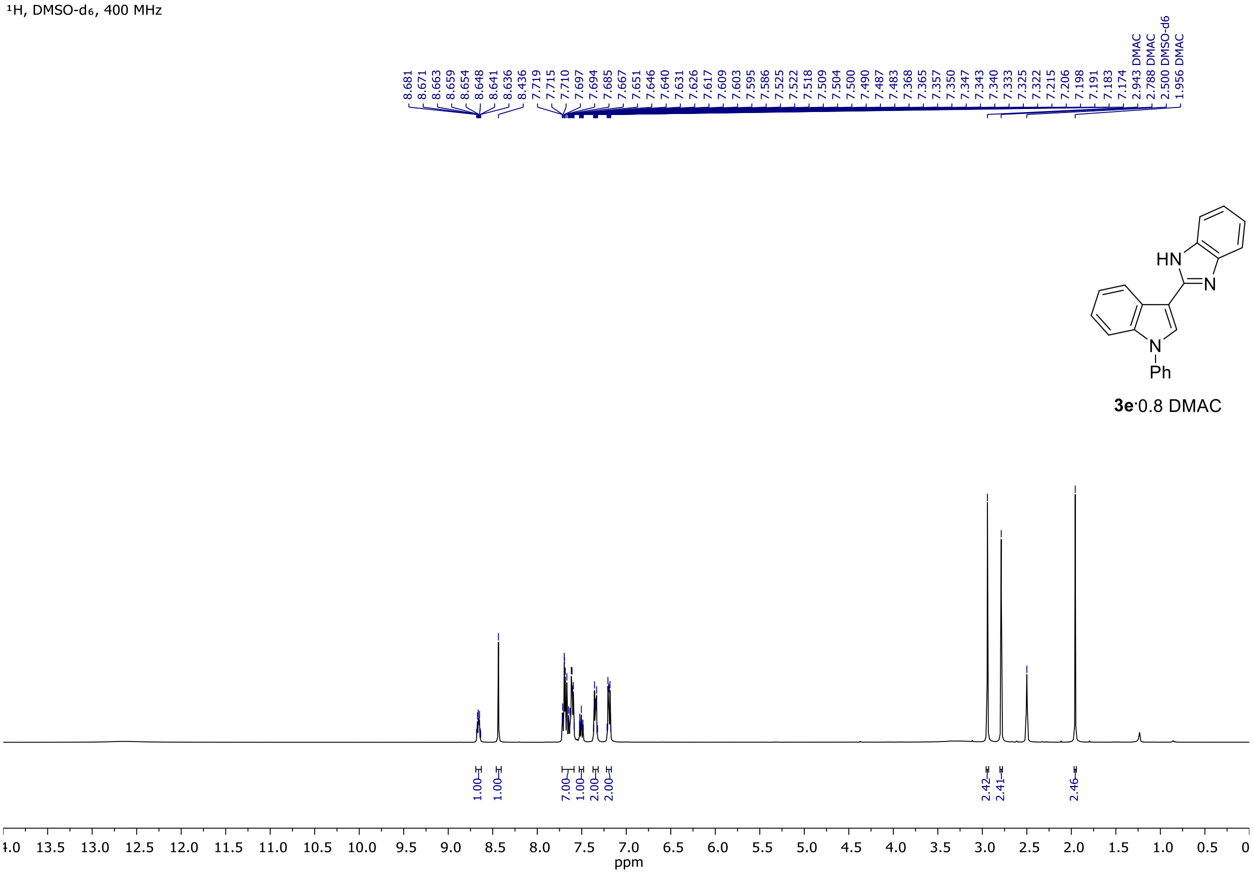

¹³C{¹H}, DMSO-d₆, 100 MHz

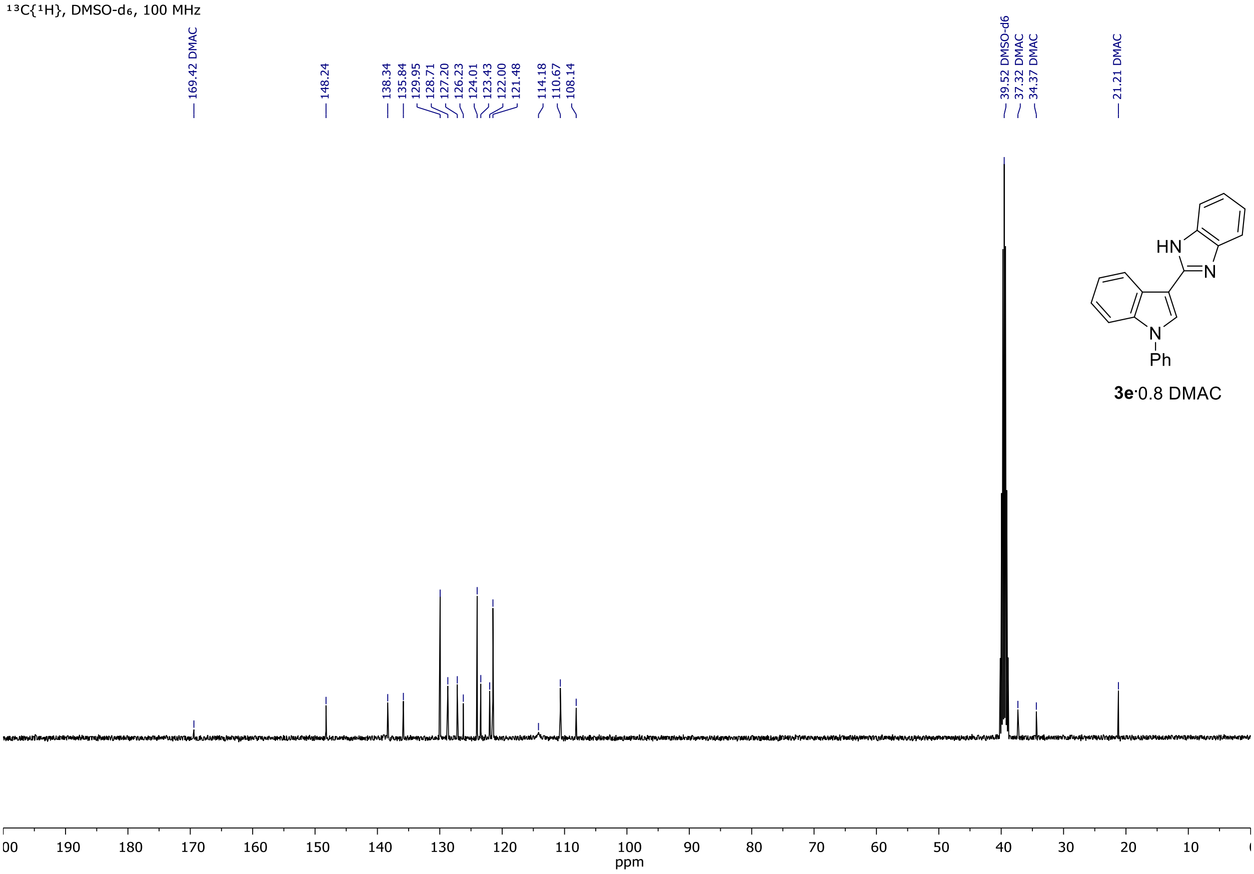

¹H, DMSO-d₆, 400 MHz

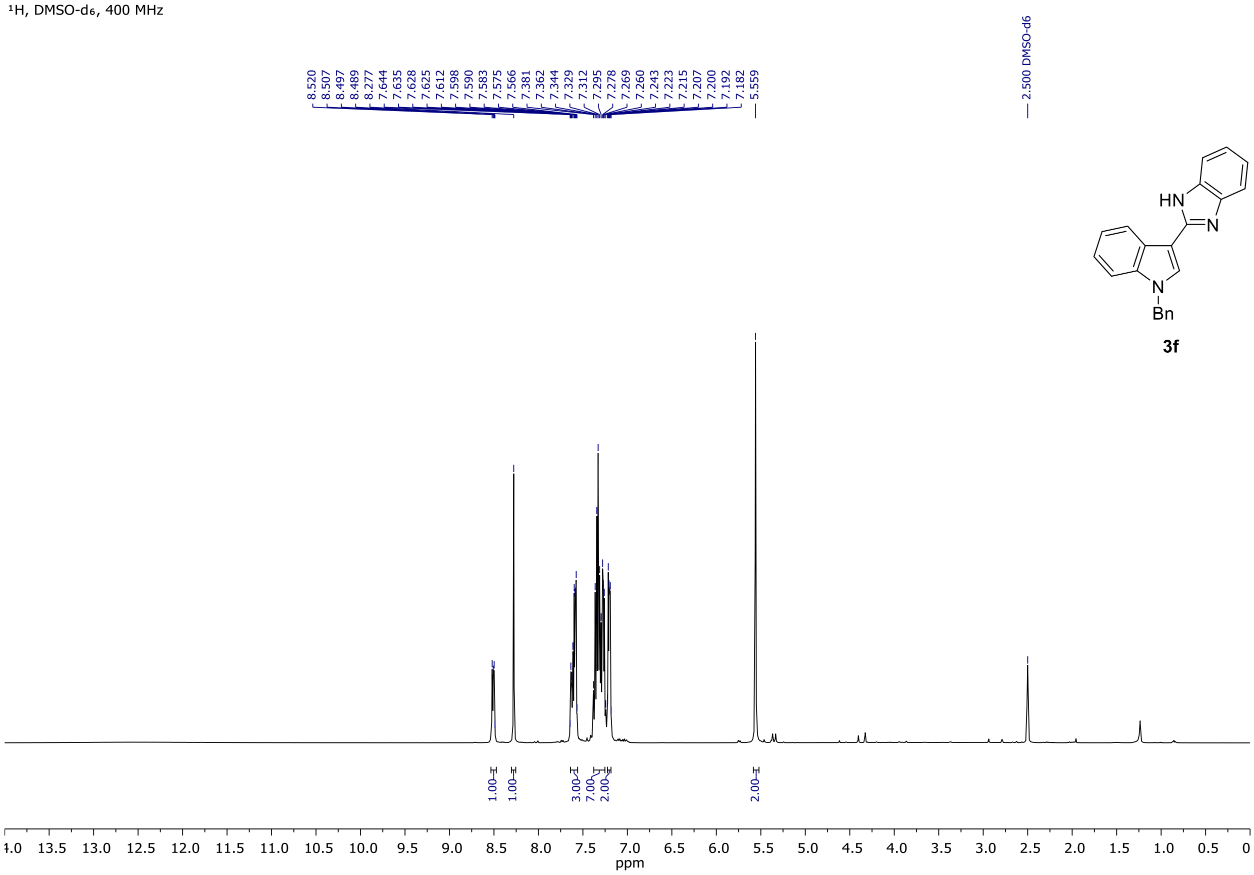

3f

¹³C{¹H}, DMSO-d₆, 100 MHz

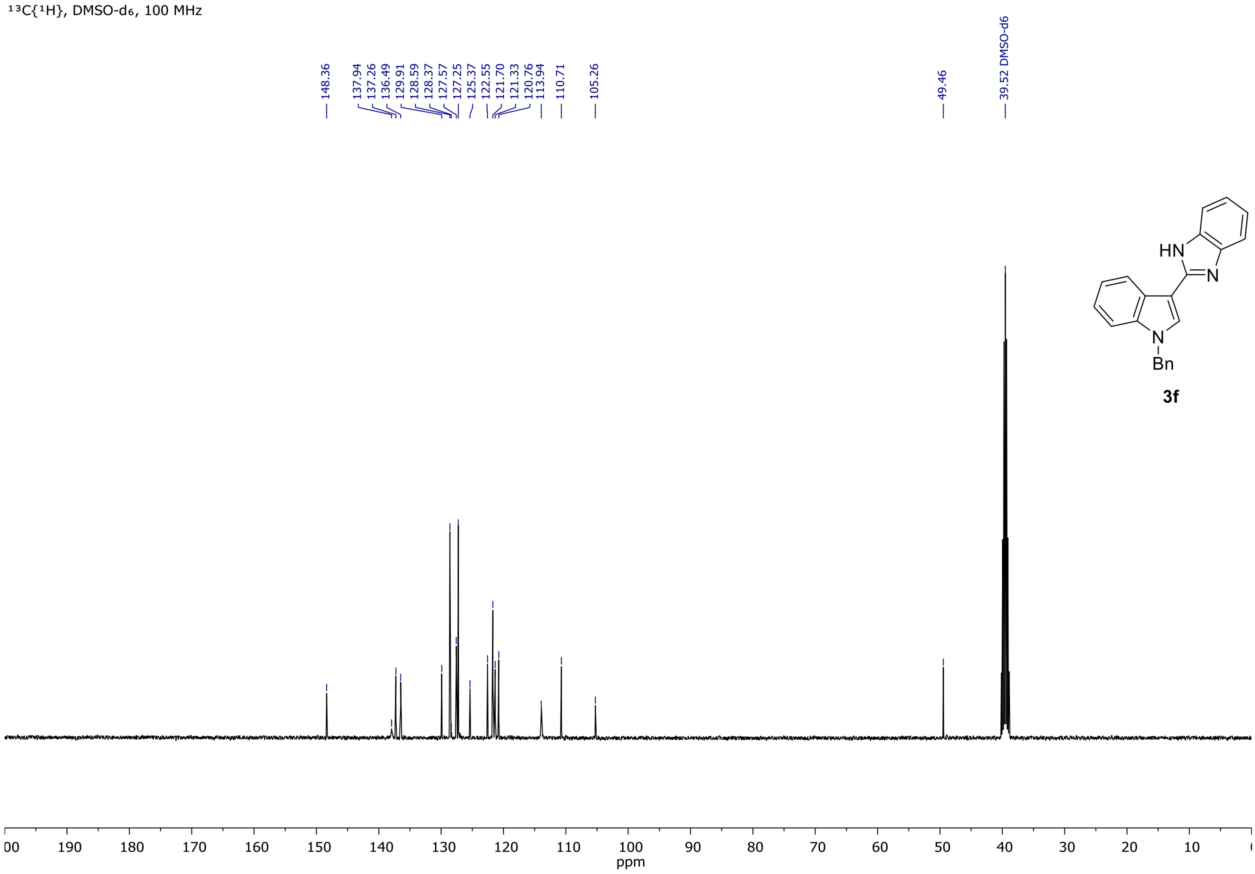

3f

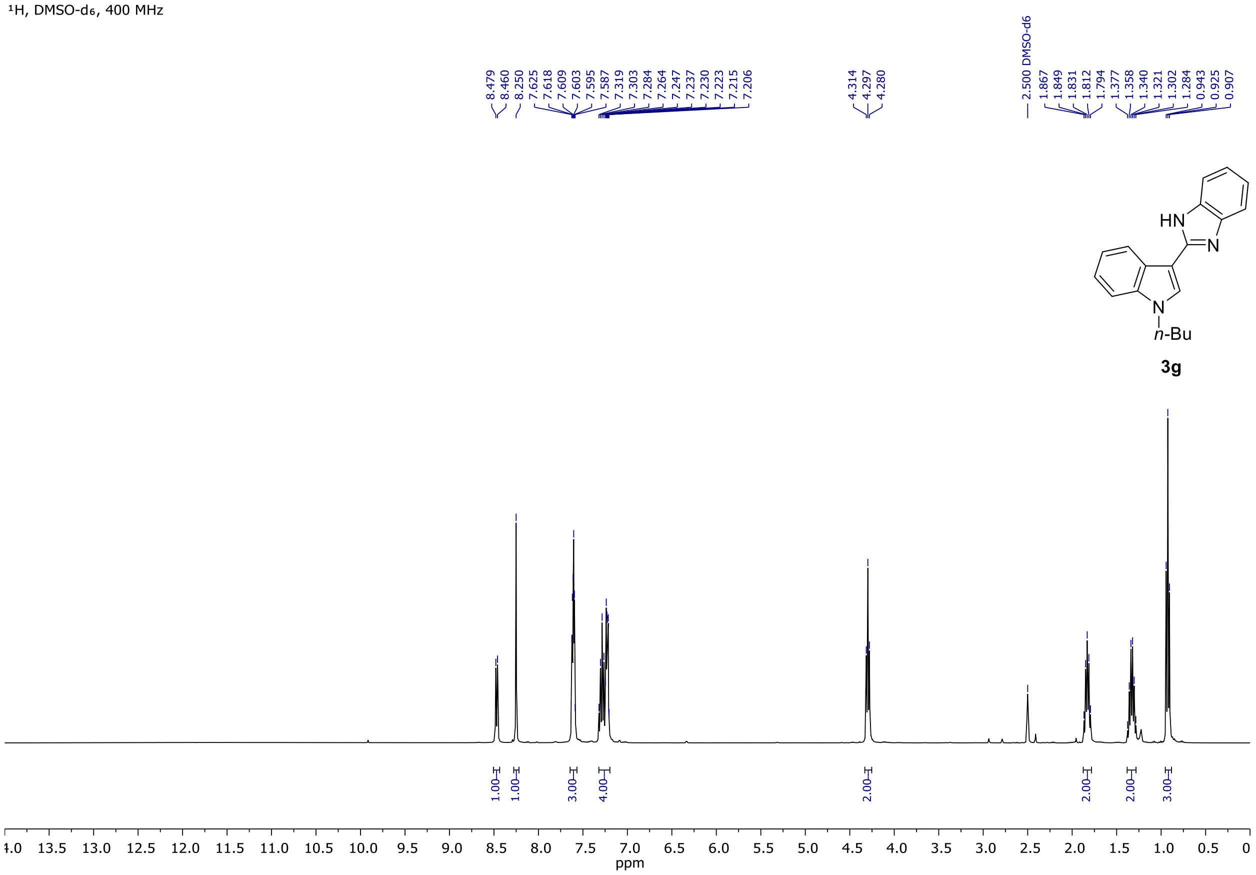
¹H, DMSO-d₆, 400 MHz
HN
N
N
n-Bu
3g
ppm

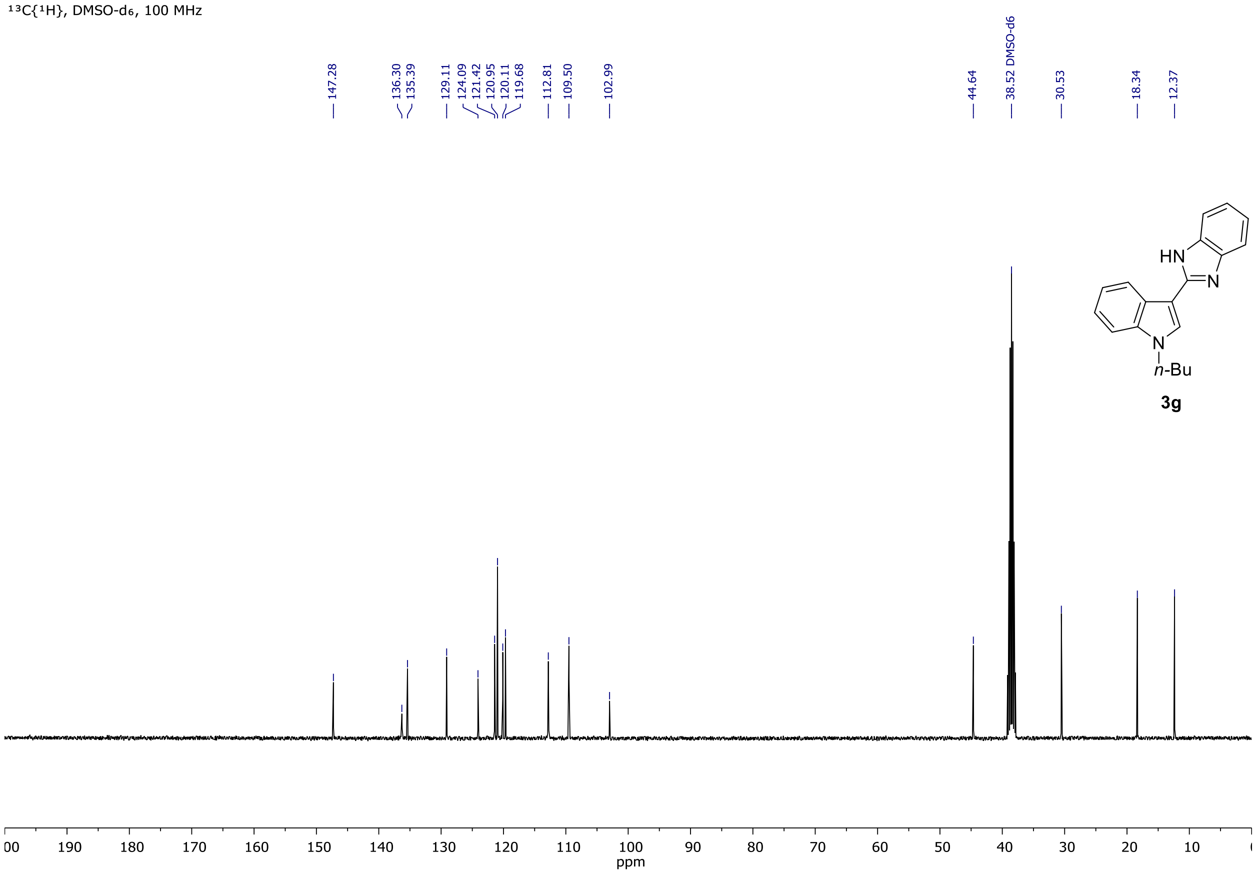
¹³C{¹H}, DMSO-d₆, 100 MHz
HN
N
N
n-Bu
3g
ppm

^{1}H, DMSO-d_6, 400 MHz

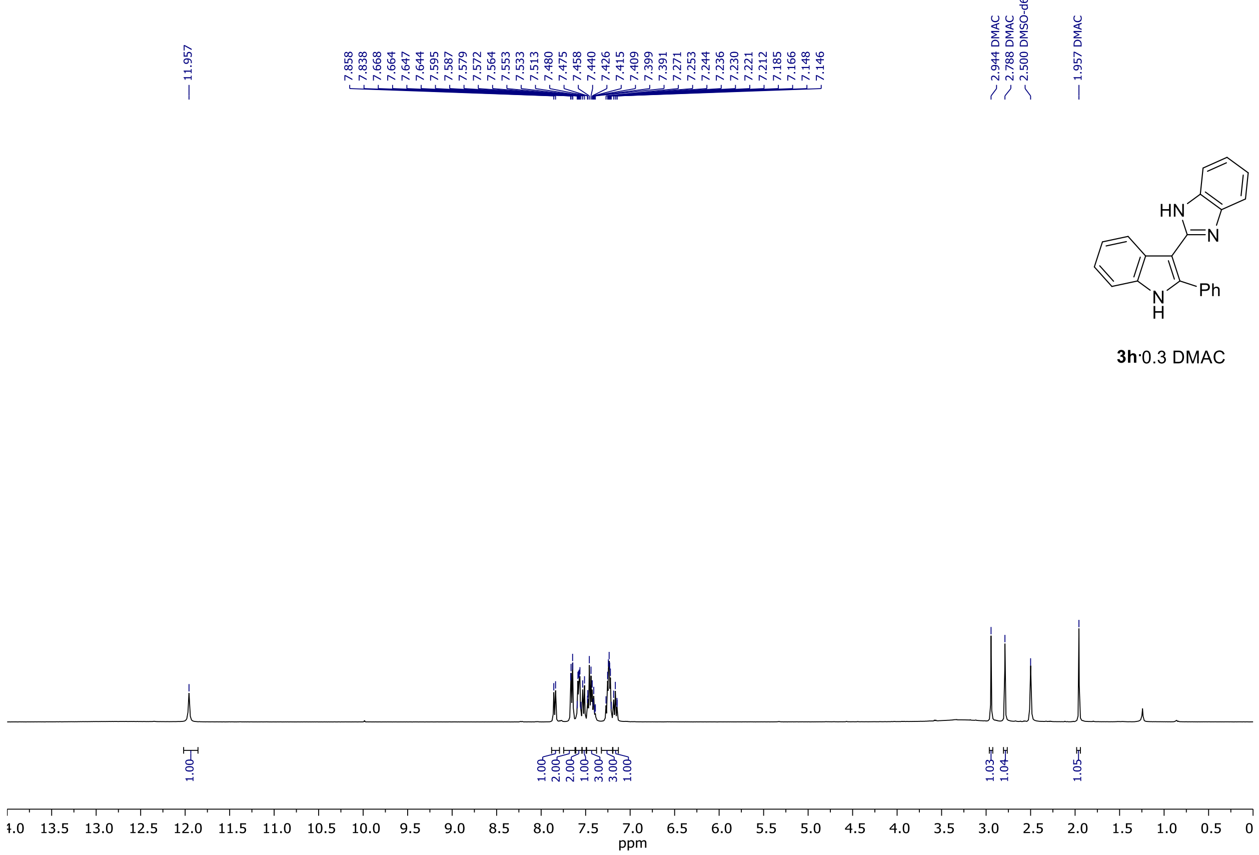

$^{13}C\{^{1}H\}$, DMSO-d_6, 100 MHz

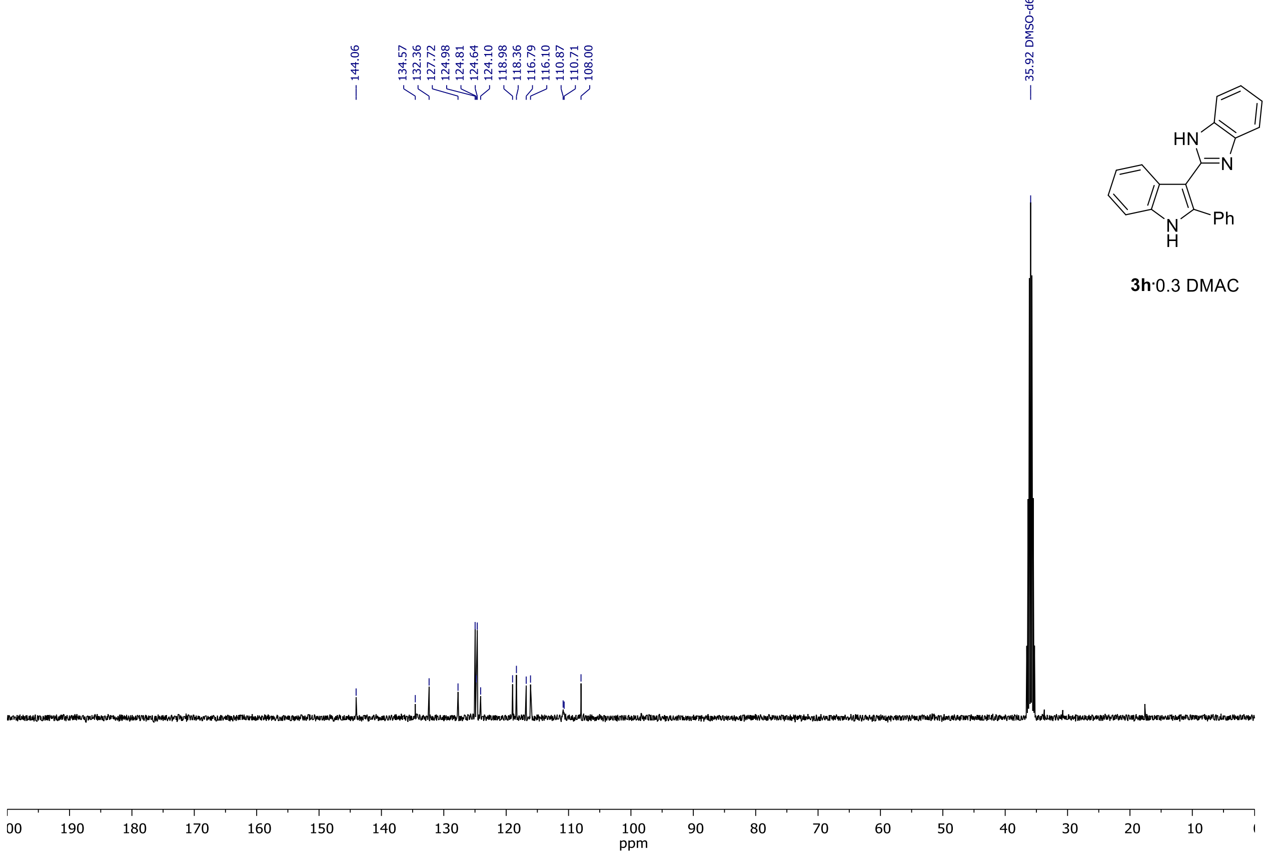

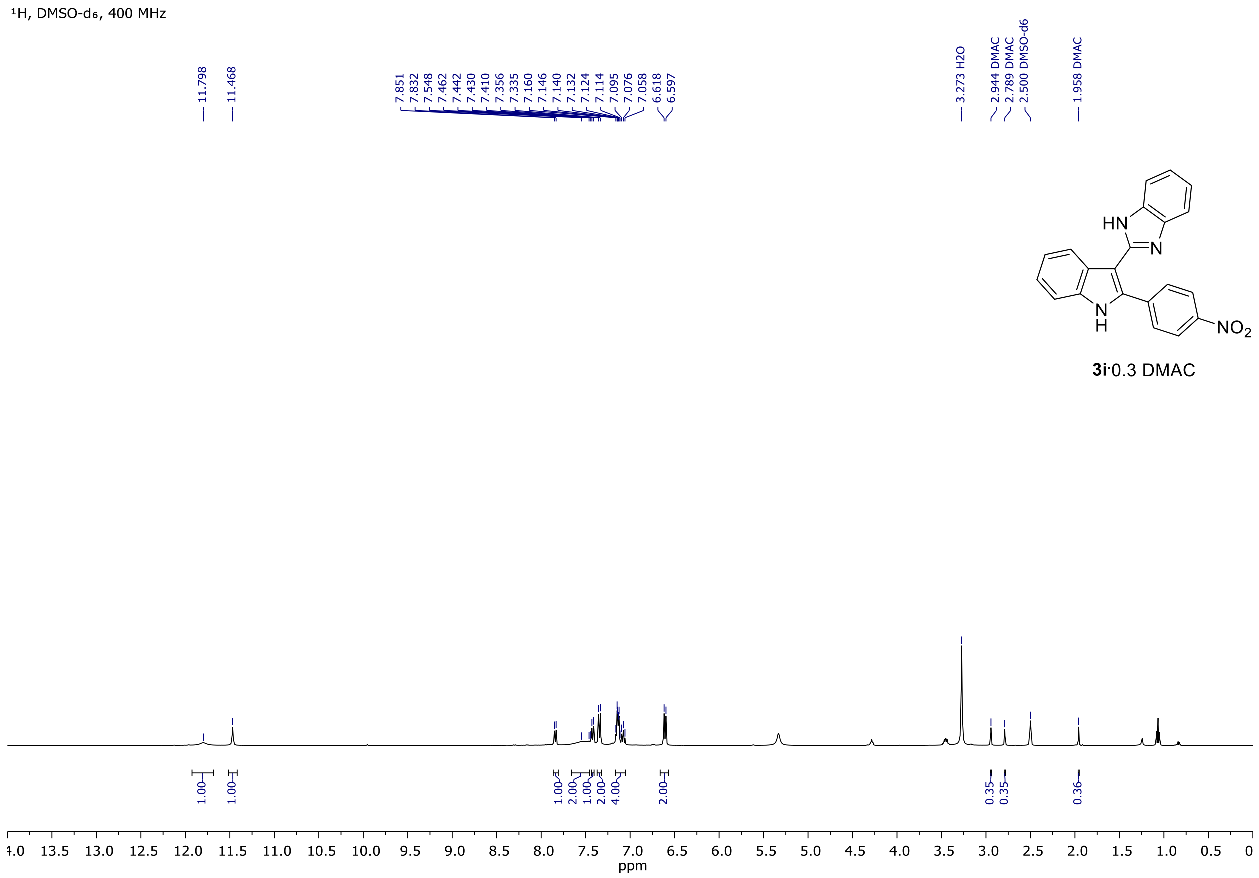
¹H, DMSO-d₆, 400 MHz
11.798
11.468
7.851 7.832 7.548 7.462 7.442 7.430 7.410 7.356 7.335 7.160 7.146 7.140 7.132 7.124 7.114 7.095 7.076 7.058 6.618 6.597
3.273 H2O
2.944 DMAC
2.789 DMAC
2.500 DMSO-d6
1.958 DMAC
HN
N
H
NO2
3i·0.3 DMAC
1.00 1.00 1.00 2.00 1.00 2.00 4.00 2.00 0.35 0.35 0.36
ppm

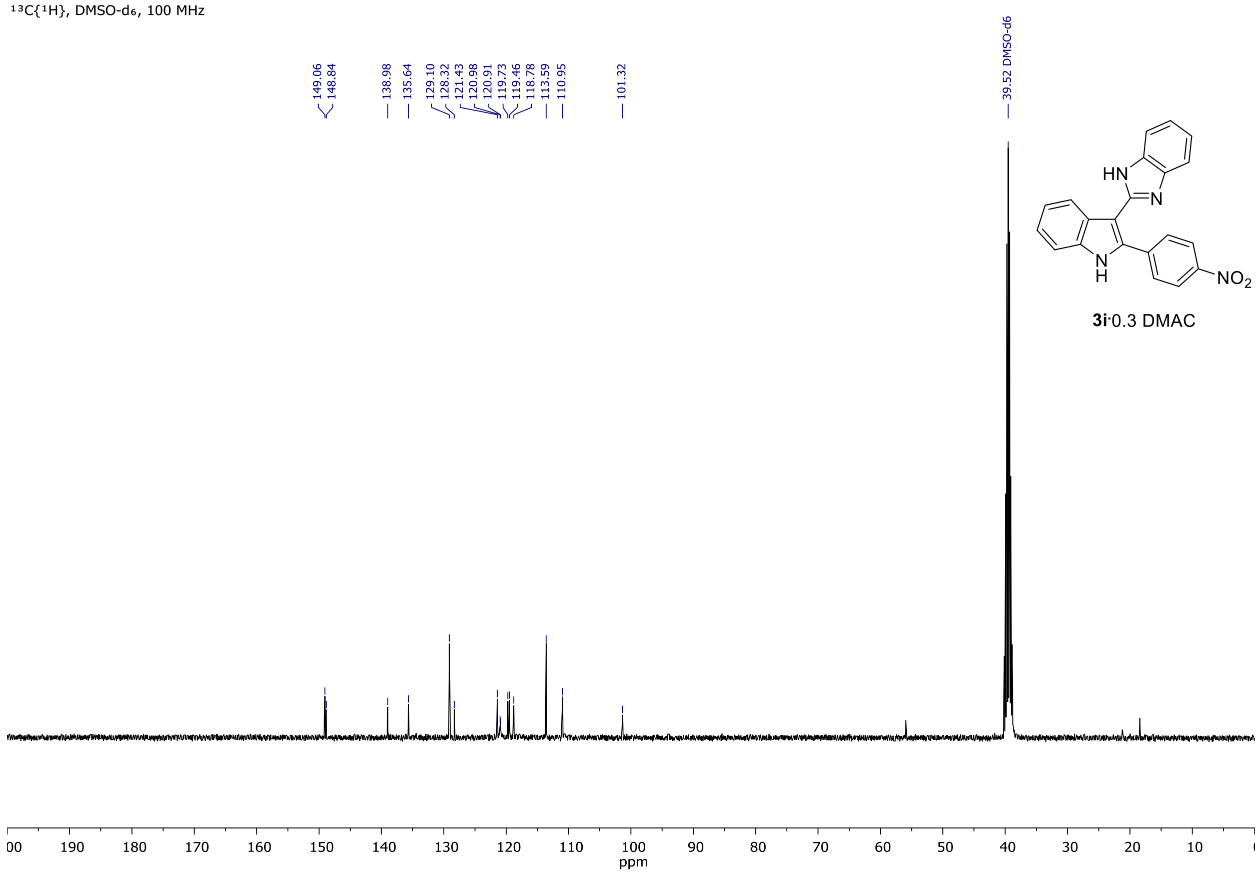
¹³C{¹H}, DMSO-d₆, 100 MHz
149.06 148.84
138.98
135.64
129.10 128.32 121.43 120.98 120.91 119.73 119.46 118.78
113.59
110.95
101.32
39.52 DMSO-d6
HN
N
H
NO2
3i·0.3 DMAC
ppm

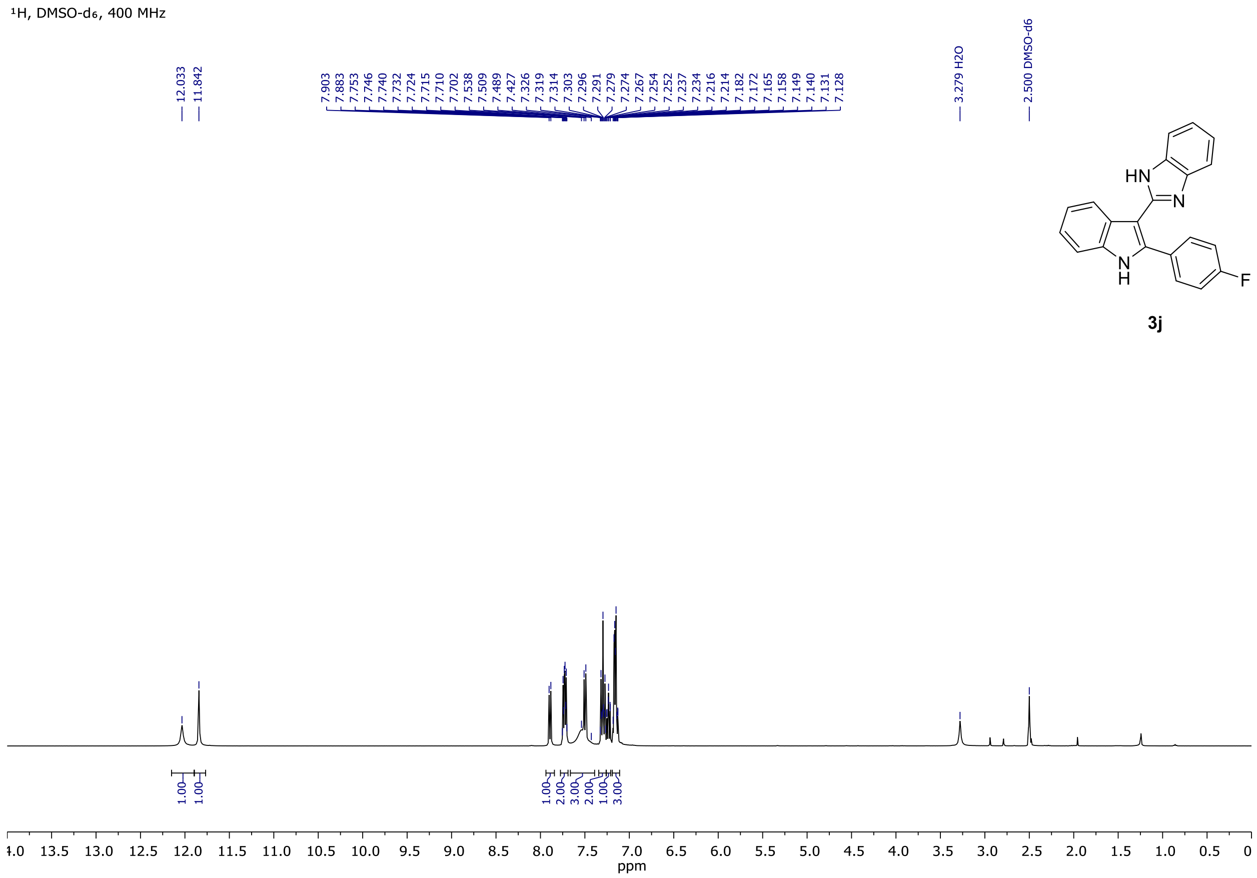

¹H, DMSO-d₆, 400 MHz
12.033
11.842
7.903 7.883 7.753 7.746 7.740 7.732 7.724 7.715 7.710 7.702 7.538 7.509 7.489 7.427 7.326 7.319 7.314 7.303 7.296 7.291 7.279 7.274 7.267 7.254 7.252 7.237 7.234 7.216 7.214 7.182 7.172 7.165 7.158 7.149 7.140 7.131 7.128
3.279 H2O
2.500 DMSO-d6
HN
N
N
H
F
3j
1.00 1.00
1.00 2.00 3.00 2.00 1.00 3.00
ppm

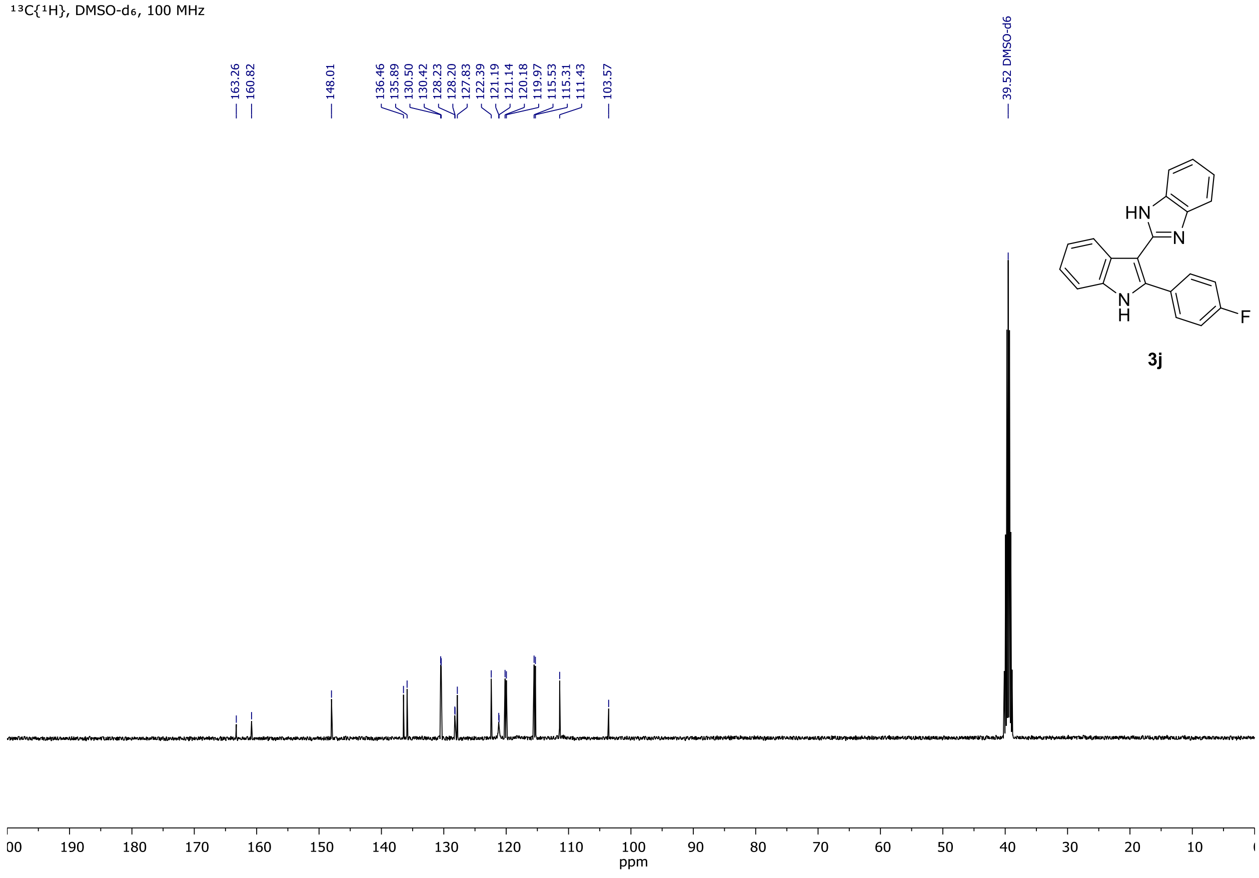

¹³C{¹H}, DMSO-d₆, 100 MHz
163.26
160.82
148.01
136.46 135.89 130.50 130.42 128.23 128.20 127.83 122.39 121.19 121.14 120.18 119.97 115.53 115.31 111.43
103.57
39.52 DMSO-d6
HN
N
N
H
F
3j
ppm

¹H, DMSO-d₆, 400 MHz

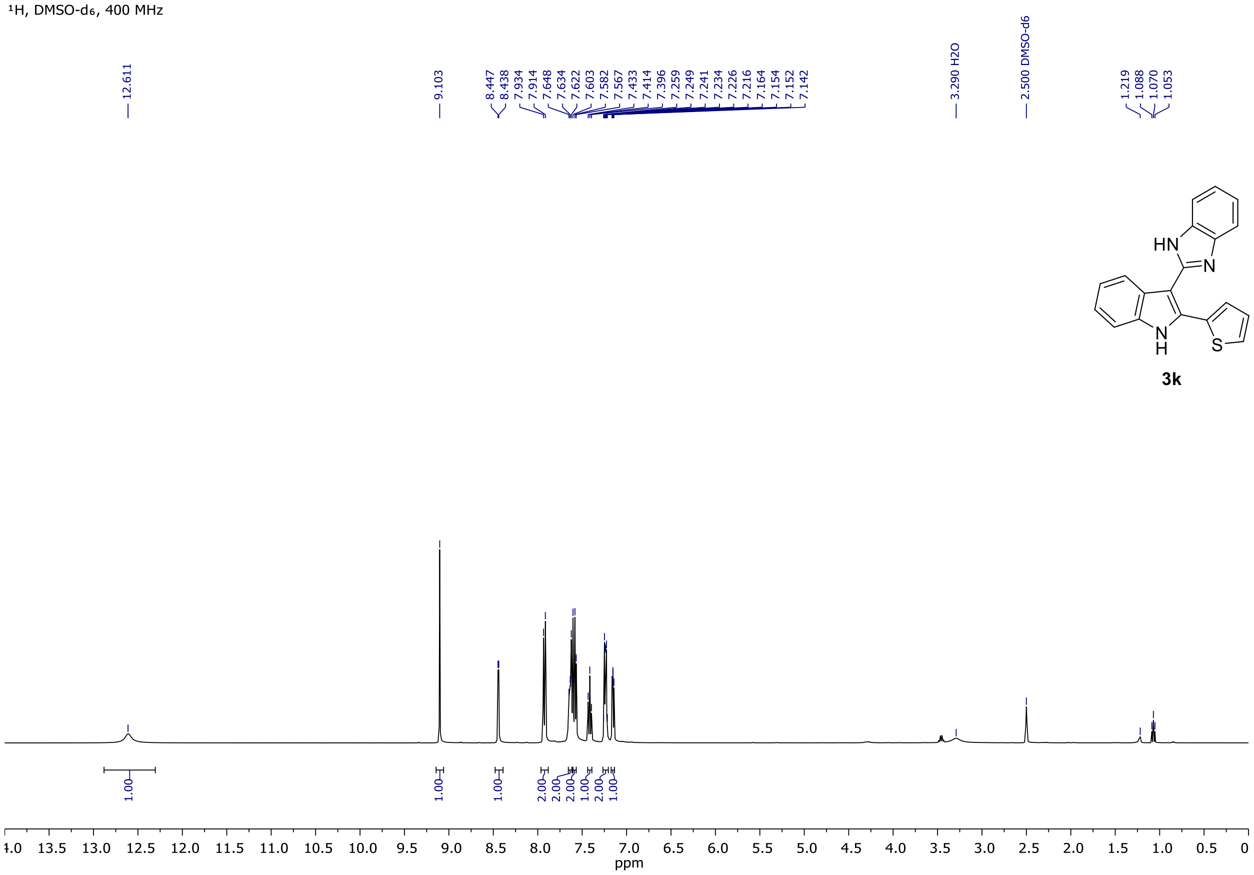

3k

¹³C{¹H}, DMSO-d₆, 100 MHz

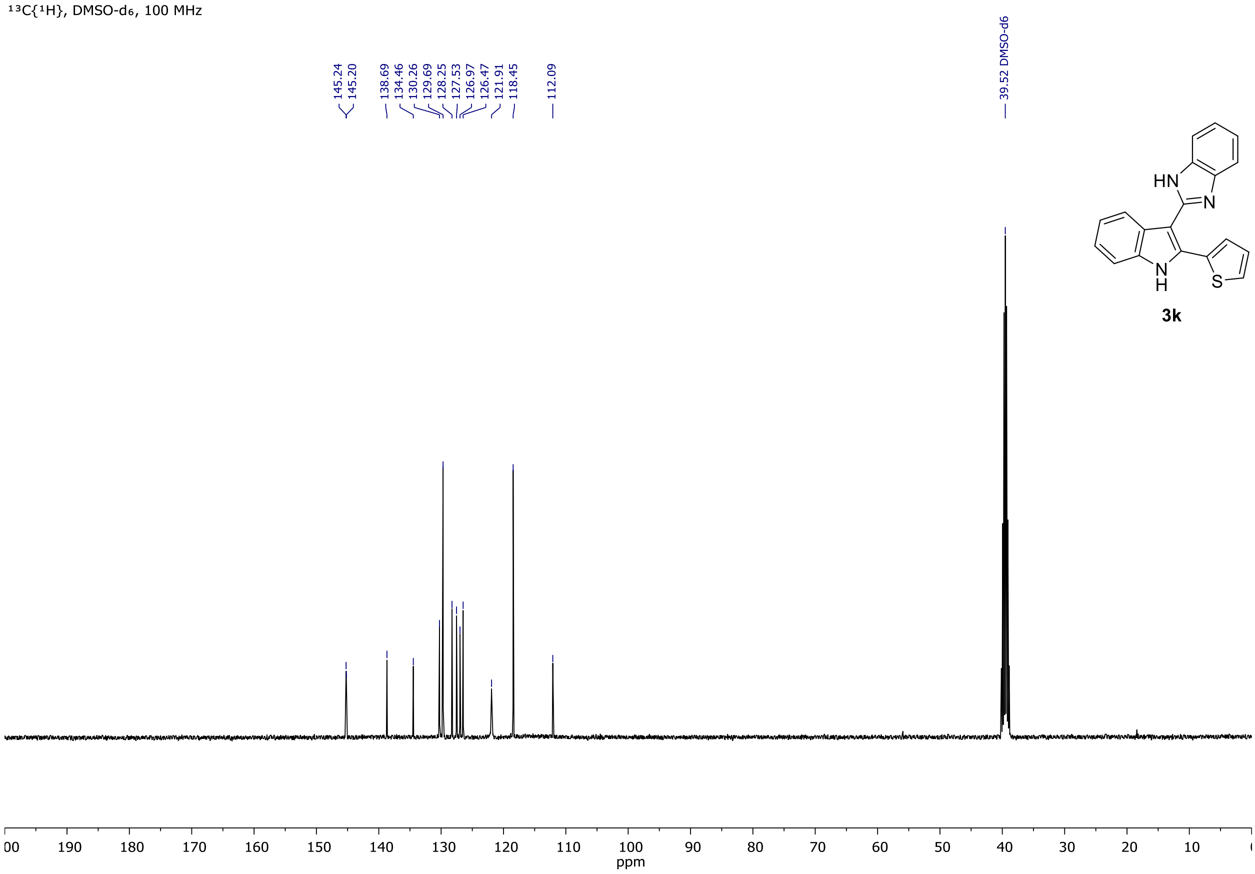

3k

^{1}H, DMSO-d_6, 400 MHz

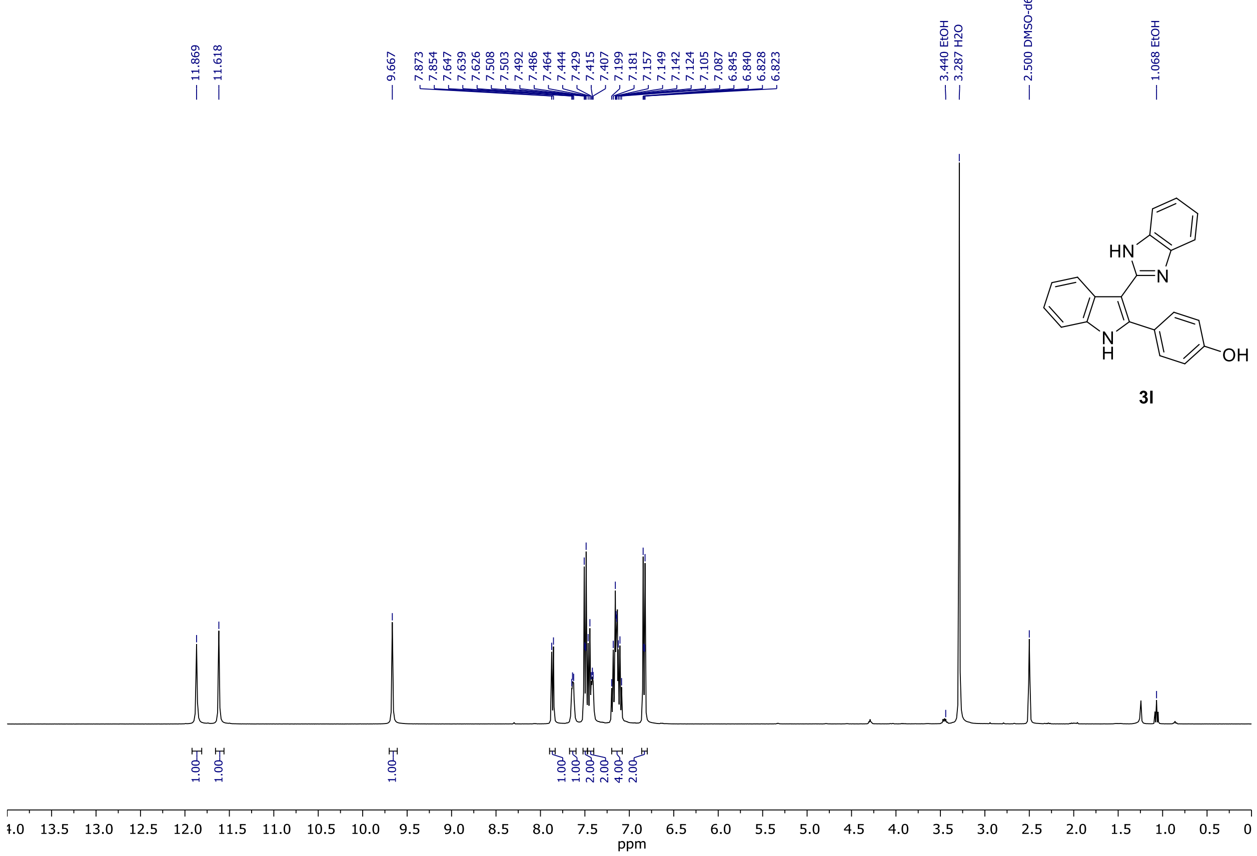

$^{13}C\{^{1}H\}$, DMSO-d_6, 100 MHz

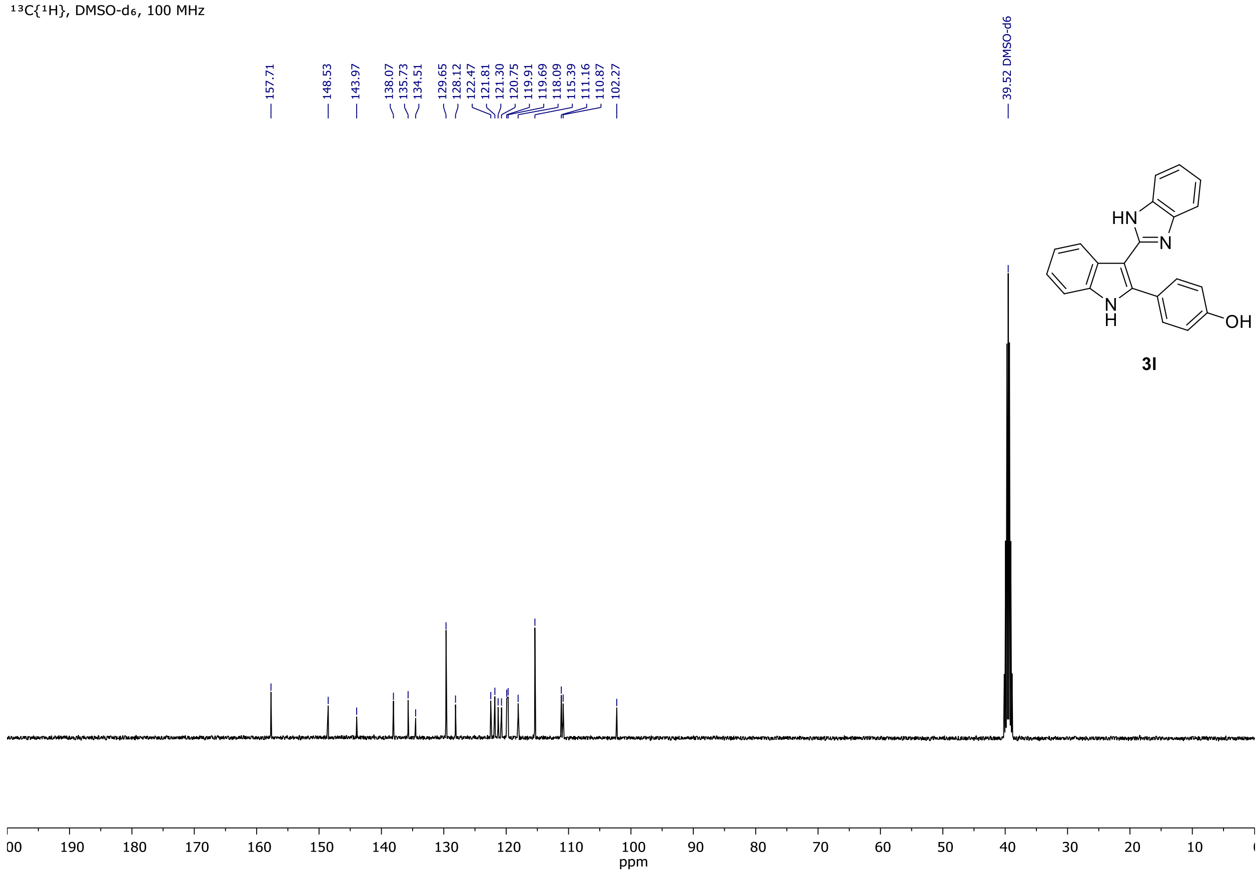

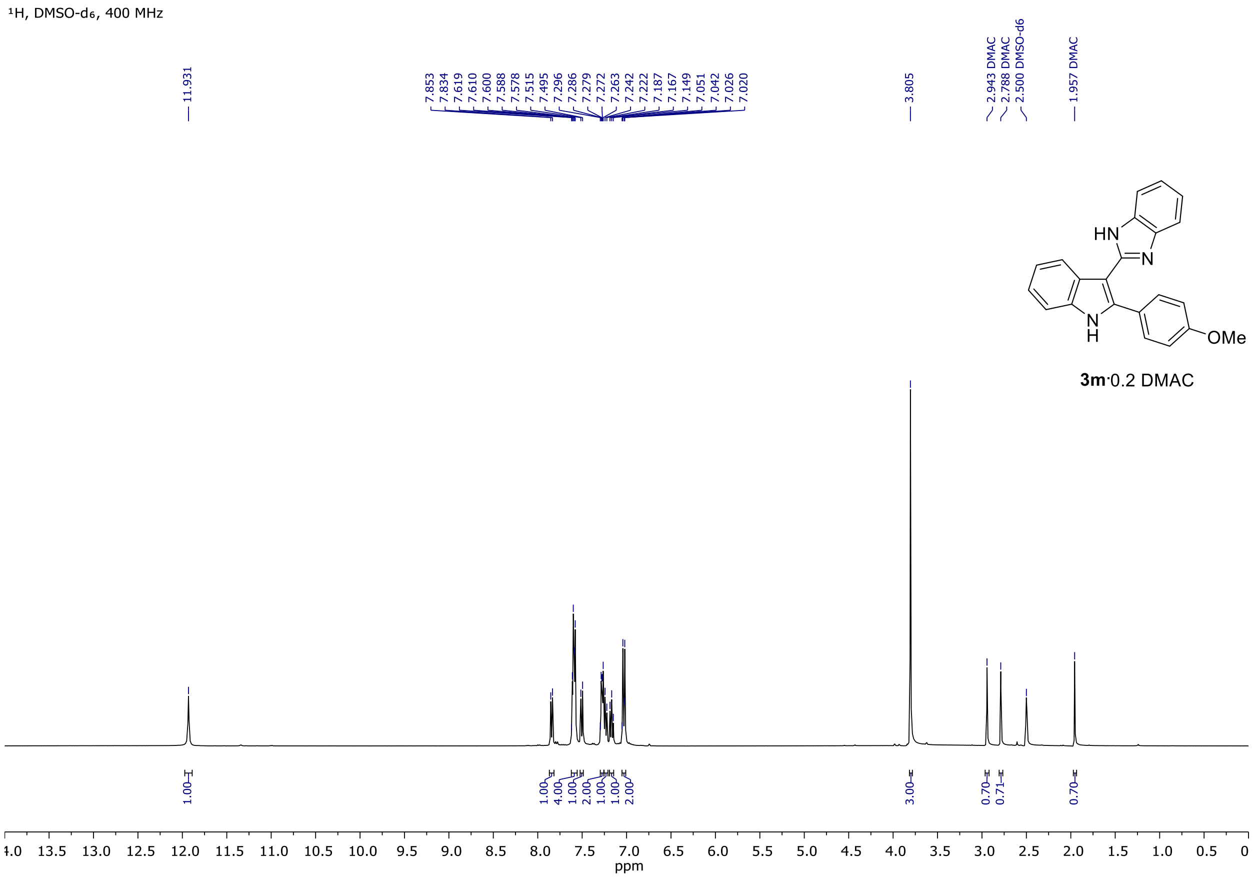
¹H, DMSO-d₆, 400 MHz
11.931
7.853 7.834 7.619 7.610 7.600 7.588 7.578 7.515 7.495 7.296 7.286 7.279 7.272 7.263 7.242 7.222 7.187 7.167 7.149 7.051 7.042 7.026 7.020
3.805
2.943 DMAC
2.788 DMAC
2.500 DMSO-d6
1.957 DMAC
HN
N
N
H
OMe
3m·0.2 DMAC
1.00
1.00 4.00 1.00 2.00 1.00 1.00 2.00
3.00
0.70 0.71
0.70
ppm

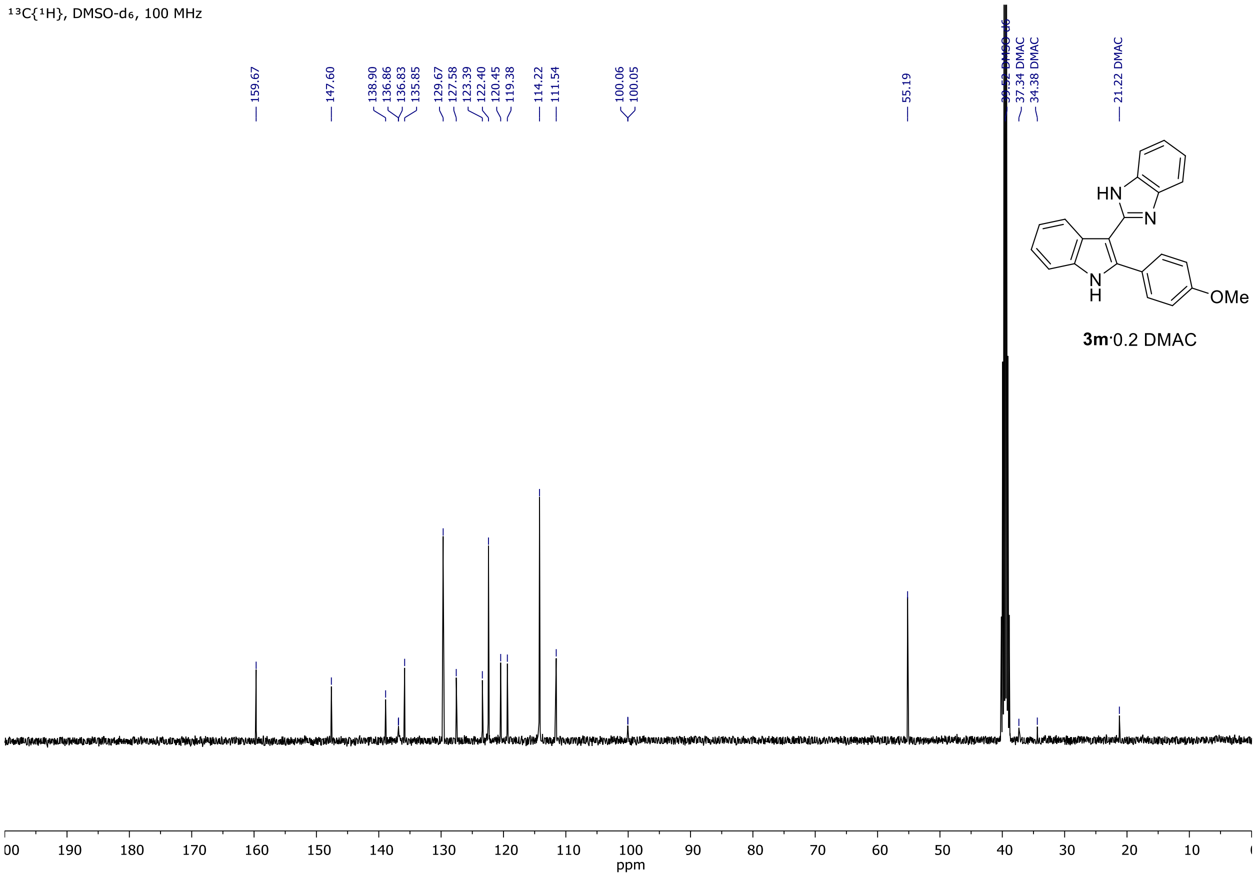
¹³C{¹H}, DMSO-d₆, 100 MHz
159.67
147.60
138.90 136.86 136.83 135.85
129.67 127.58 123.39 122.40 120.45 119.38
114.22 111.54
100.06 100.05
55.19
37.34 DMAC
34.38 DMAC
21.22 DMAC
HN
N
N
H
OMe
3m·0.2 DMAC
ppm

^1H, DMSO-d_6, 400 MHz

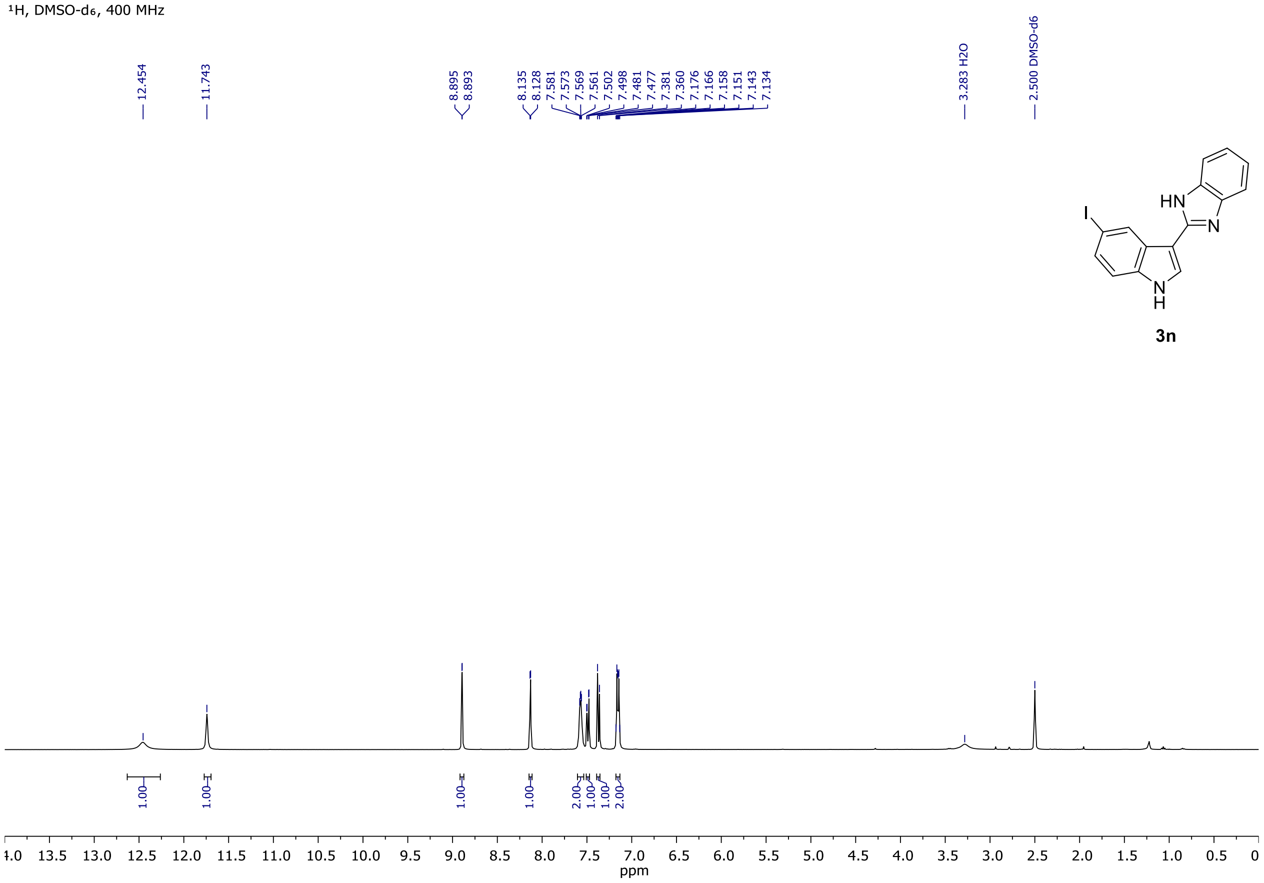

3n

$^{13}C\{^1H\}$, DMSO-d_6, 100 MHz

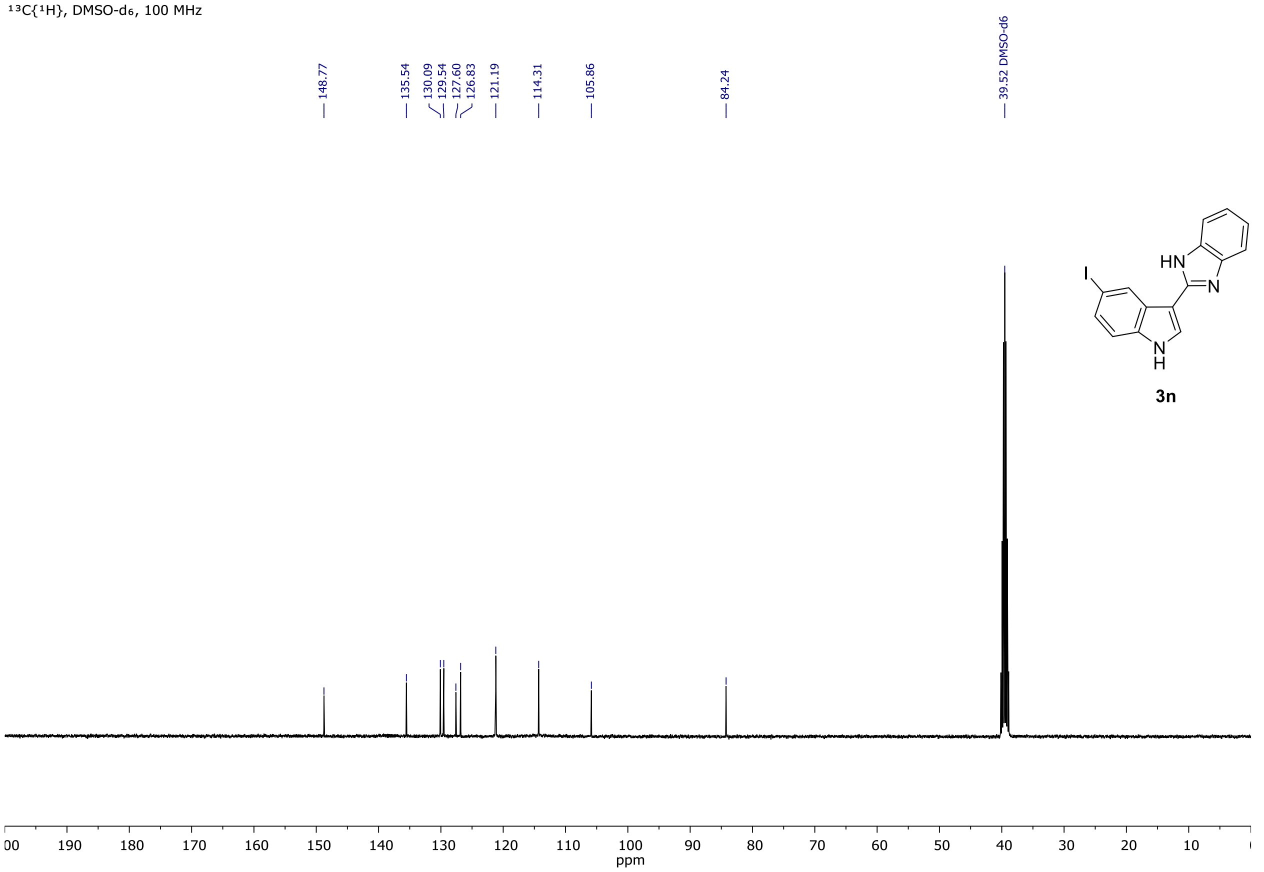

3n

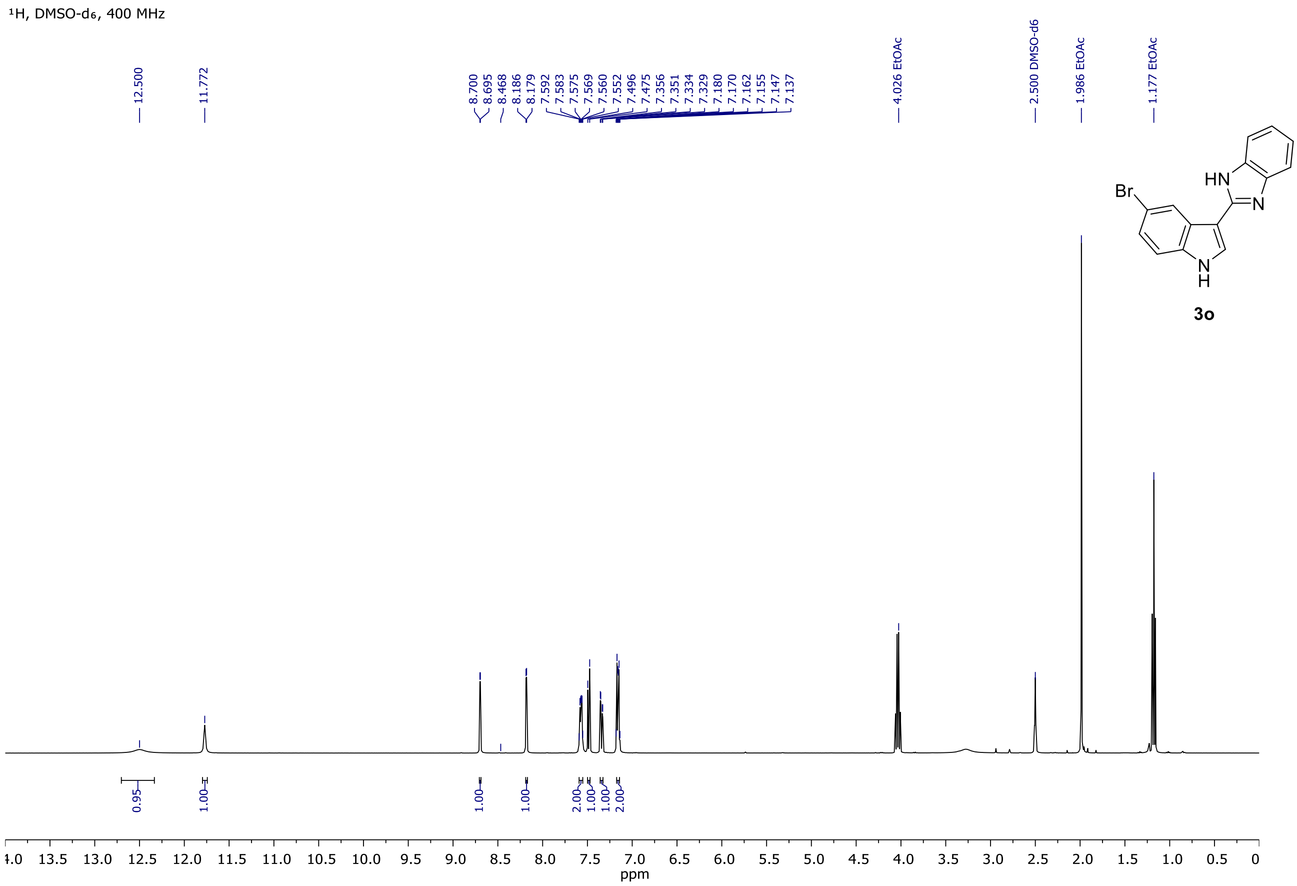
¹H, DMSO-d₆, 400 MHz
12.500
11.772
8.700
8.695
8.468
8.186
8.179
7.592
7.583
7.575
7.569
7.560
7.552
7.496
7.475
7.356
7.351
7.334
7.329
7.180
7.170
7.162
7.155
7.147
7.137
4.026 EtOAc
2.500 DMSO-d6
1.986 EtOAc
1.177 EtOAc
Br
HN
N
N
H
3o
0.95
1.00
1.00
1.00
2.00
1.00
1.00
2.00
ppm

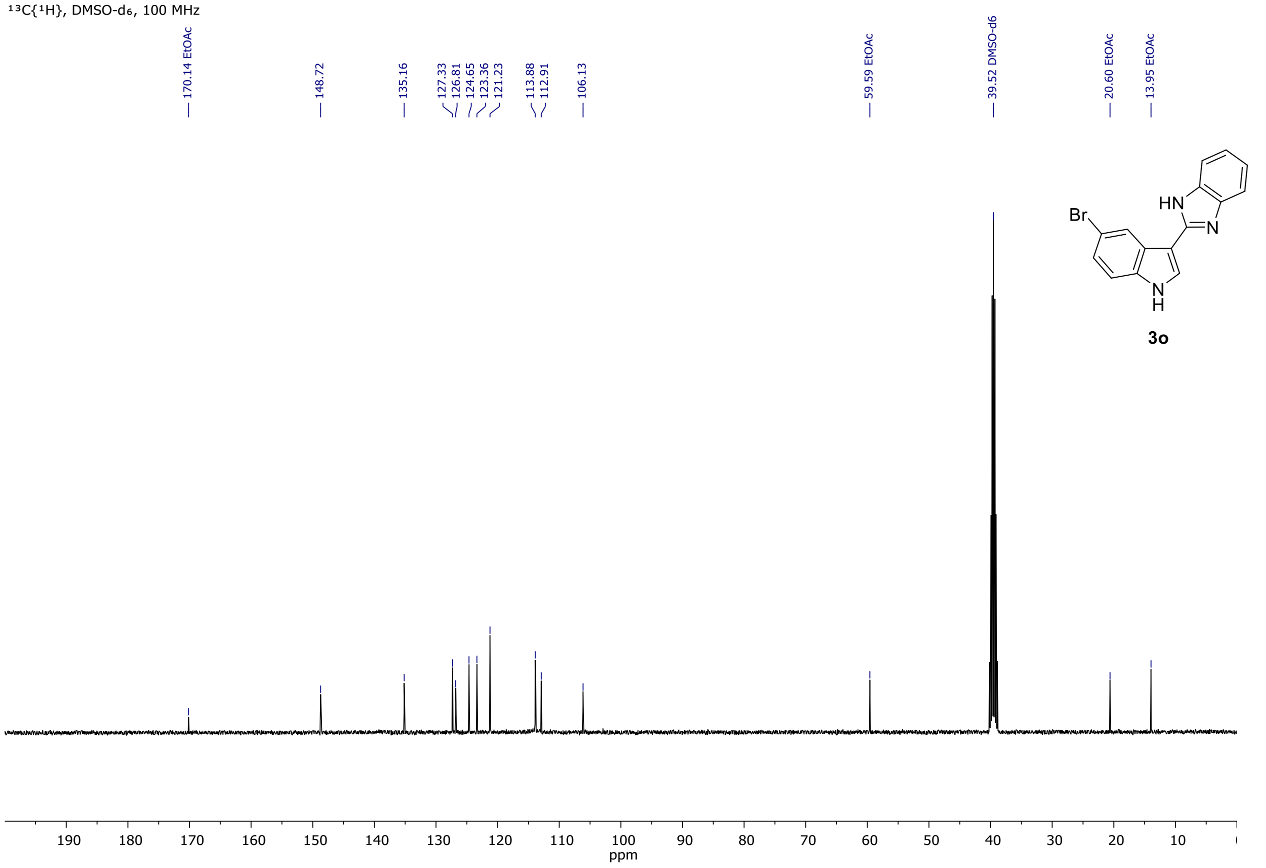
¹³C{¹H}, DMSO-d₆, 100 MHz
170.14 EtOAc
148.72
135.16
127.33
126.81
124.65
123.36
121.23
113.88
112.91
106.13
59.59 EtOAc
39.52 DMSO-d6
20.60 EtOAc
13.95 EtOAc
Br
HN
N
N
H
3o
ppm

¹H, DMSO-d₆, 400 MHz

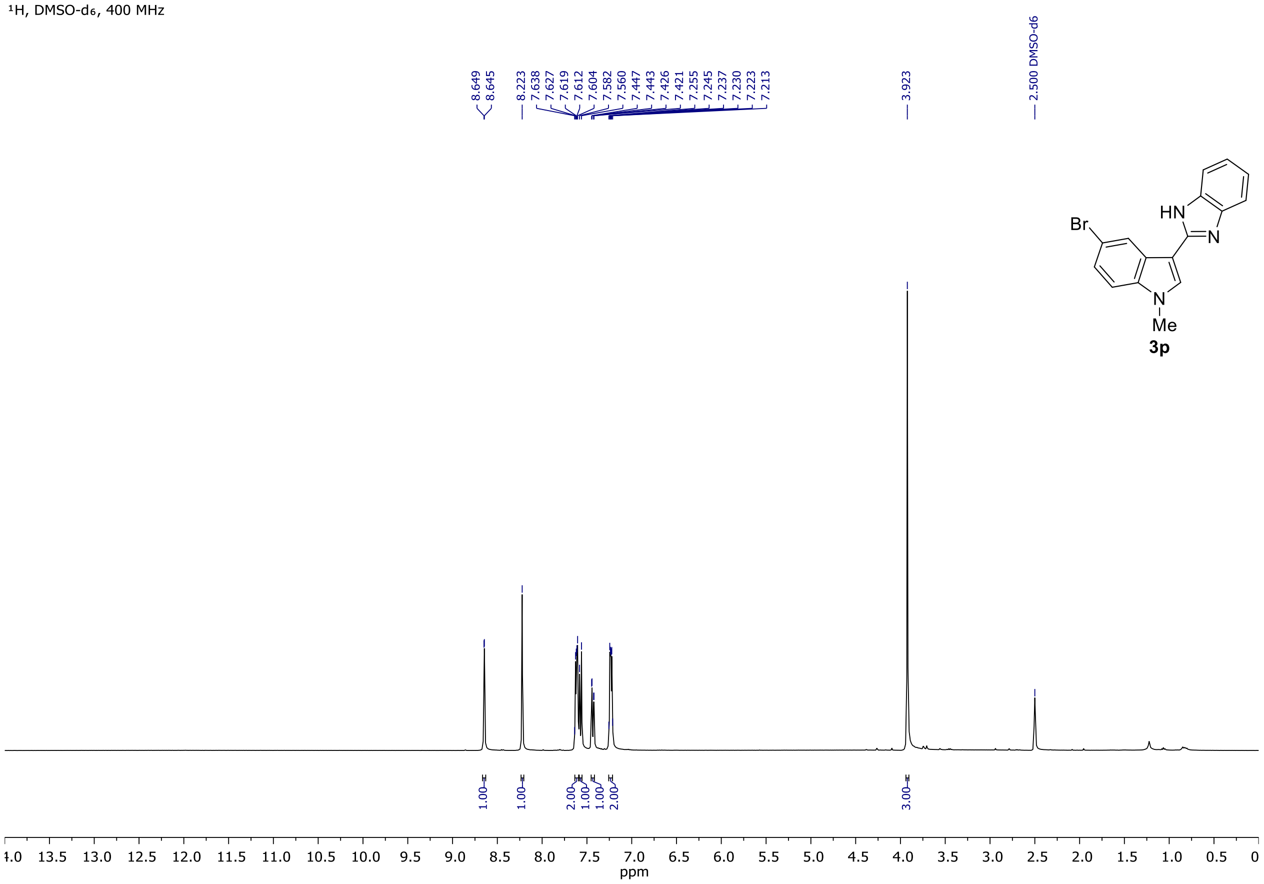

¹³C{¹H}, DMSO-d₆, 100 MHz

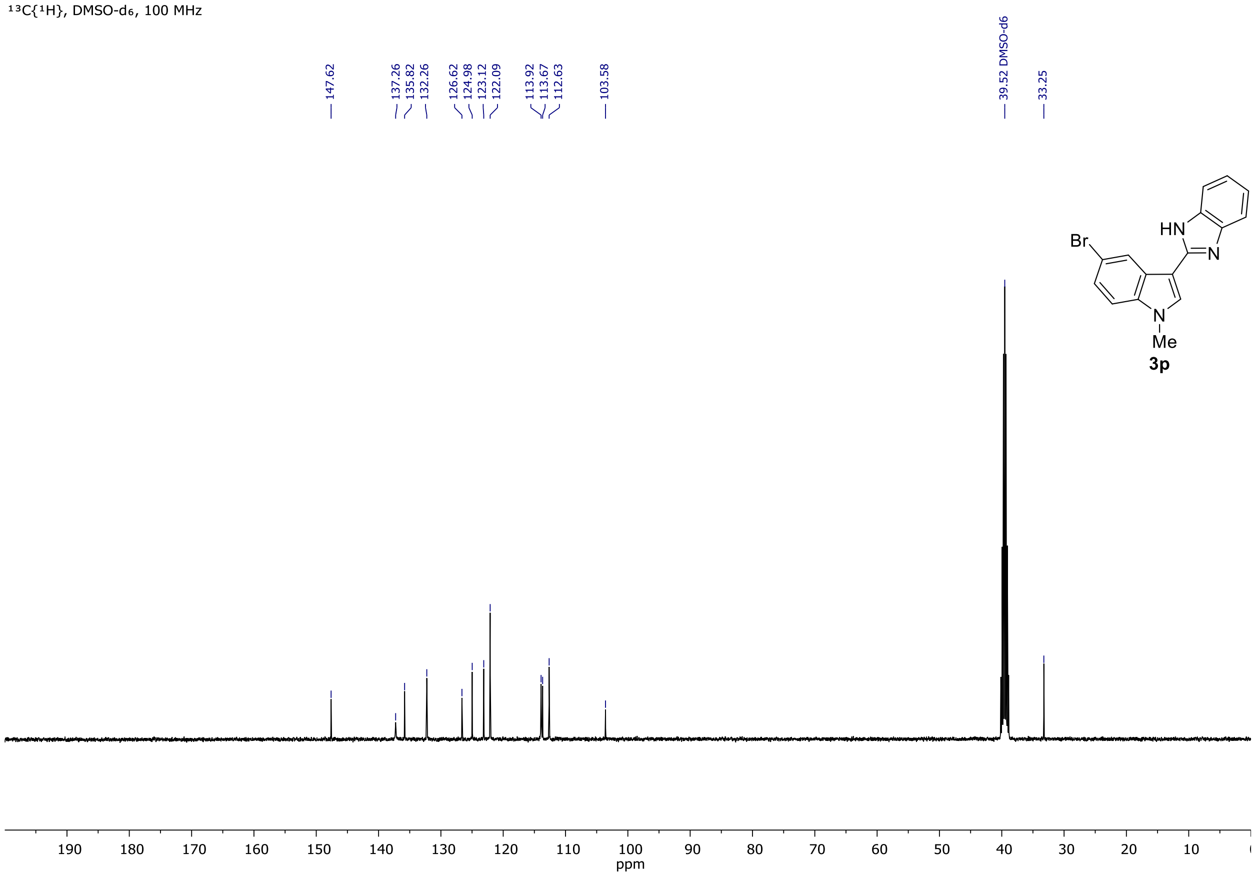

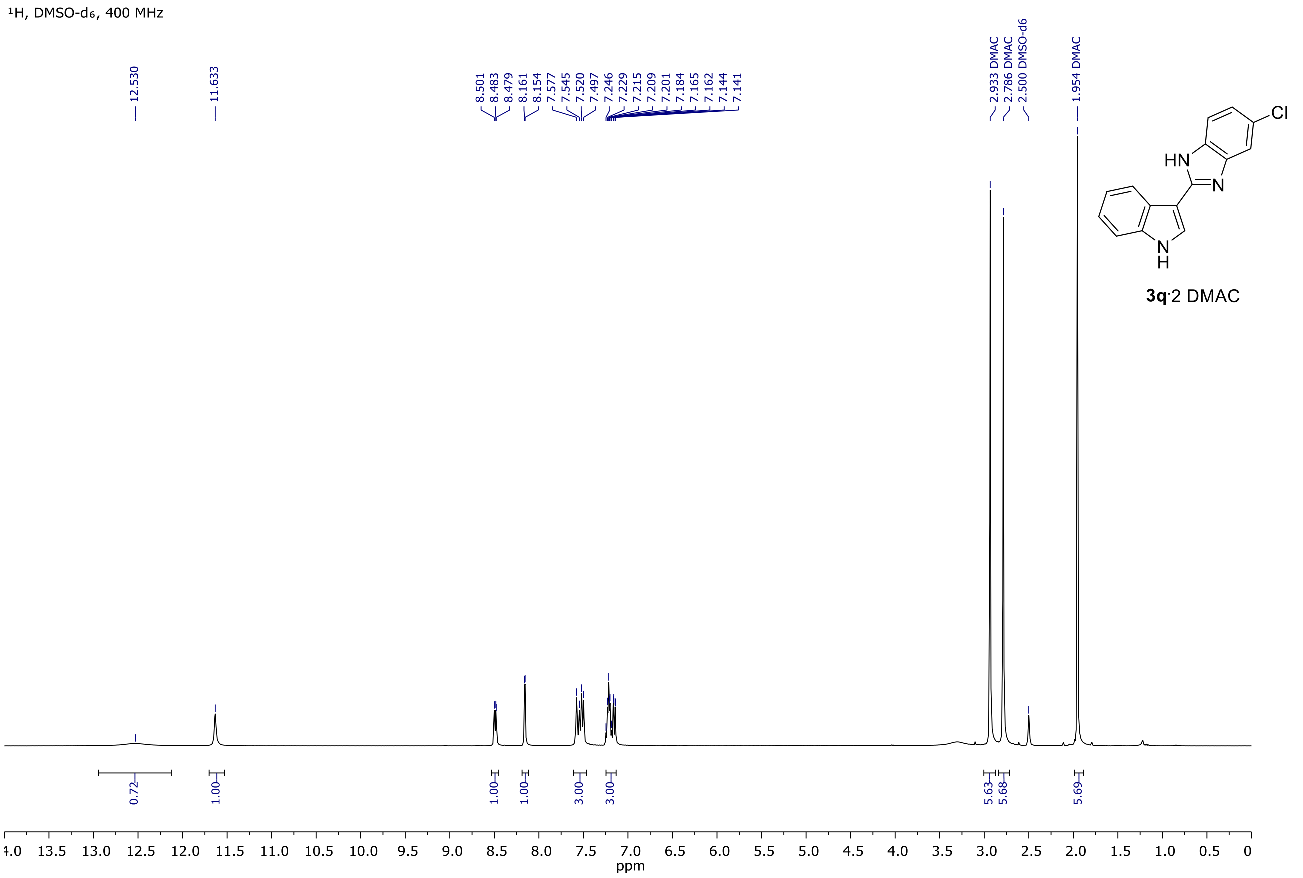
¹H, DMSO-d₆, 400 MHz
12.530
11.633
8.501
8.483
8.479
8.161
8.154
7.577
7.545
7.520
7.497
7.246
7.229
7.215
7.209
7.201
7.184
7.165
7.162
7.144
7.141
2.933 DMAC
2.786 DMAC
2.500 DMSO-d6
1.954 DMAC
0.72
1.00
1.00
1.00
3.00
3.00
5.63
5.68
5.69
ppm
HN
N
Cl
N
H
3q·2 DMAC

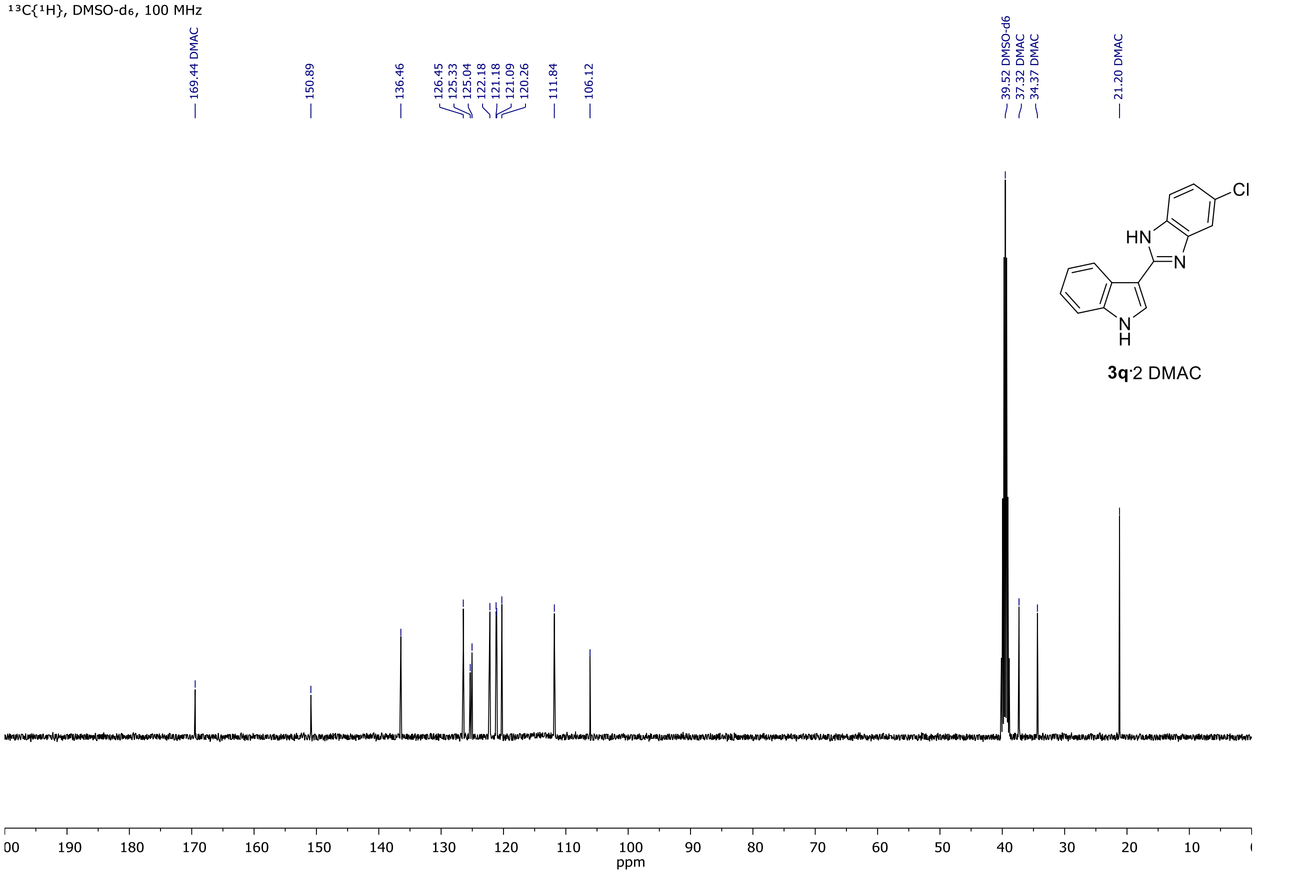
¹³C{¹H}, DMSO-d₆, 100 MHz
169.44 DMAC
150.89
136.46
126.45
125.33
125.04
122.18
121.18
121.09
120.26
111.84
106.12
39.52 DMSO-d6
37.32 DMAC
34.37 DMAC
21.20 DMAC
ppm
HN
N
Cl
N
H
3q·2 DMAC

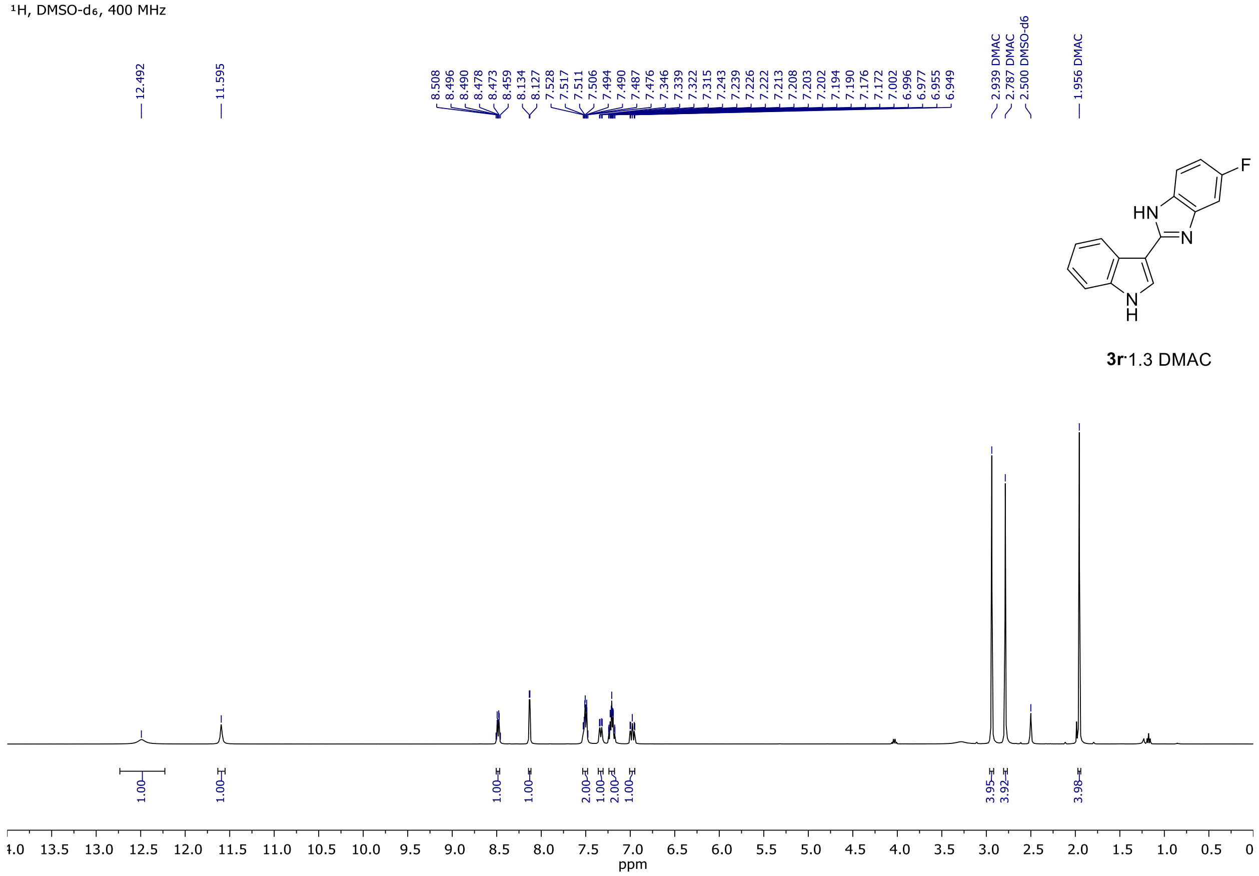
¹H, DMSO-d₆, 400 MHz
12.492
11.595
8.508
8.496
8.490
8.478
8.473
8.459
8.134
8.127
7.528
7.517
7.511
7.506
7.494
7.490
7.487
7.476
7.346
7.339
7.322
7.315
7.243
7.239
7.226
7.222
7.213
7.208
7.203
7.202
7.194
7.190
7.176
7.172
7.002
6.996
6.977
6.955
6.949
2.939 DMAC
2.787 DMAC
2.500 DMSO-d6
1.956 DMAC
F
HN
N
H
3r·1.3 DMAC
1.00
1.00
1.00
1.00
2.00
1.00
2.00
1.00
3.95
3.92
3.98
ppm

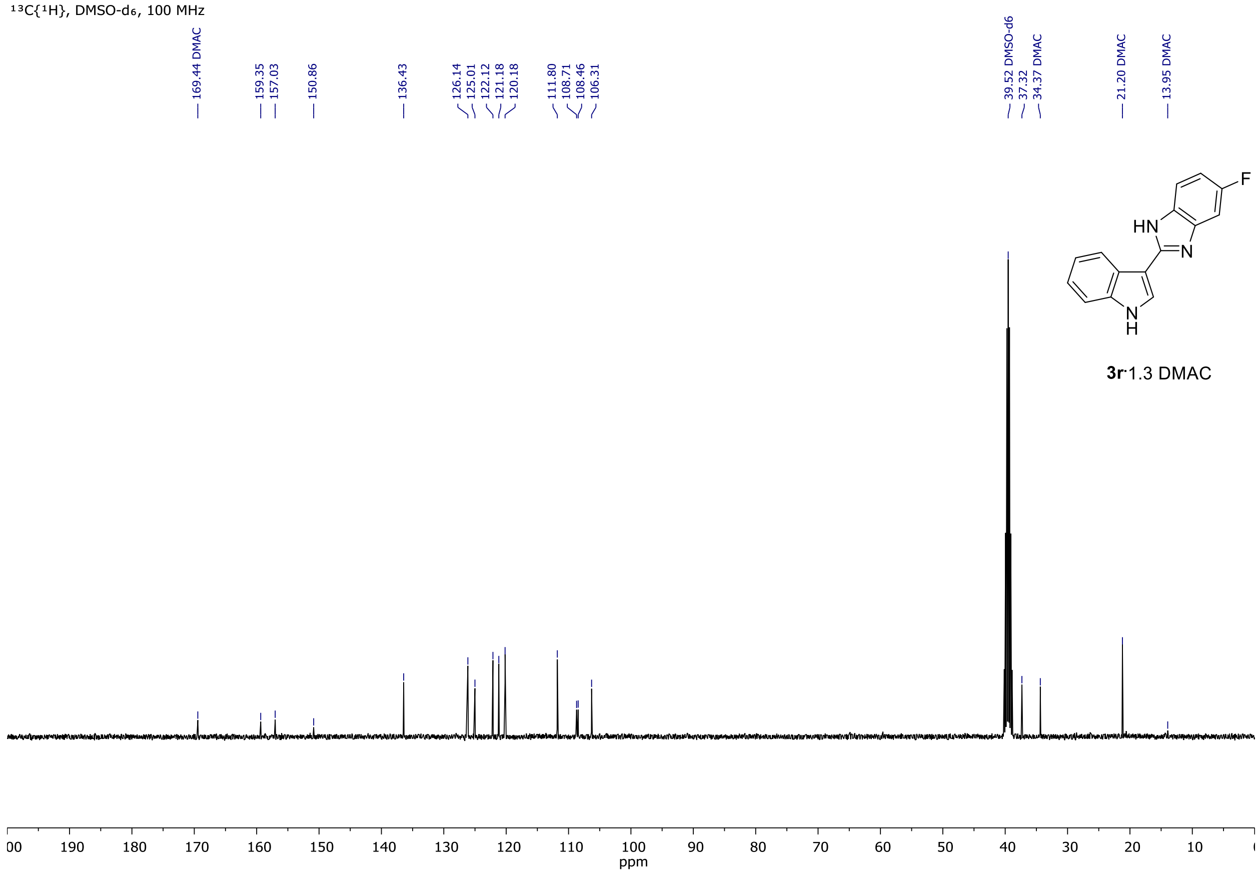
¹³C{¹H}, DMSO-d₆, 100 MHz
169.44 DMAC
159.35
157.03
150.86
136.43
126.14
125.01
122.12
121.18
120.18
111.80
108.71
108.46
106.31
39.52 DMSO-d6
37.32
34.37 DMAC
21.20 DMAC
13.95 DMAC
F
HN
N
H
3r·1.3 DMAC
ppm

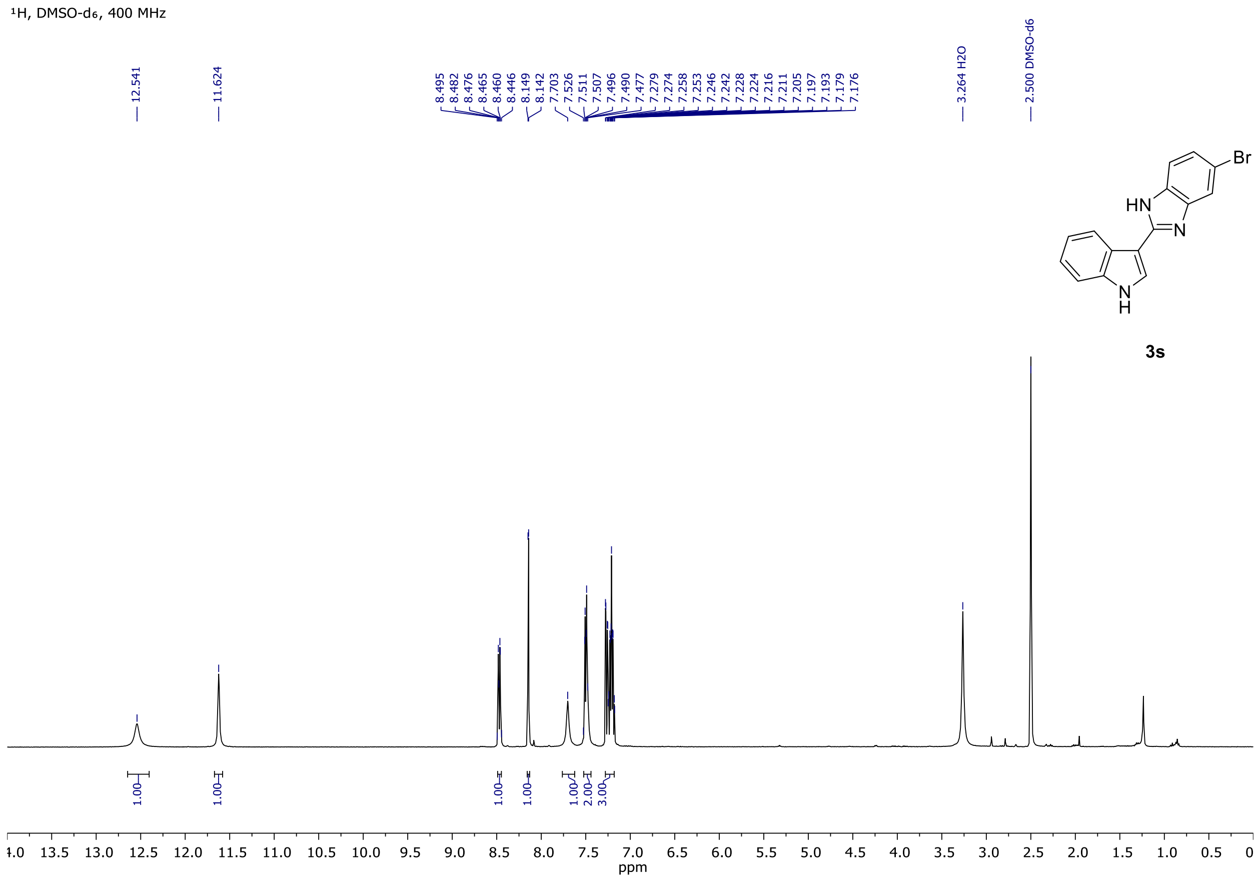
1H, DMSO-d6, 400 MHz
12.541
11.624
8.495
8.482
8.476
8.465
8.460
8.446
8.149
8.142
7.703
7.526
7.511
7.507
7.496
7.490
7.477
7.279
7.274
7.258
7.253
7.246
7.242
7.228
7.224
7.216
7.211
7.205
7.197
7.193
7.179
7.176
3.264 H2O
2.500 DMSO-d6
Br
HN
N
N
H
3s
1.00
1.00
1.00
1.00
1.00
2.00
3.00
ppm

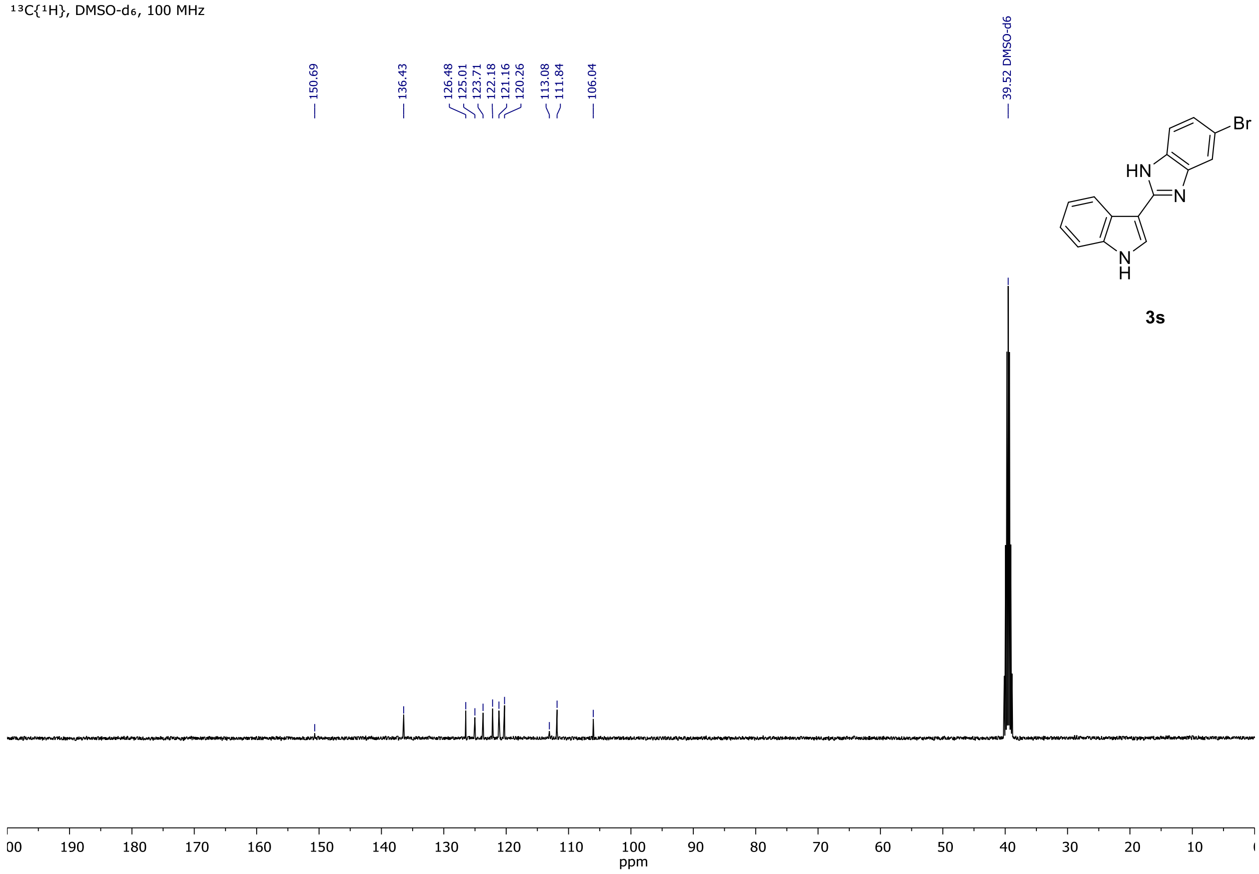
13C{1H}, DMSO-d6, 100 MHz
150.69
136.43
126.48
125.01
123.71
122.18
121.16
120.26
113.08
111.84
106.04
39.52 DMSO-d6
Br
HN
N
N
H
3s
ppm

¹H, DMSO-d₆, 400 MHz

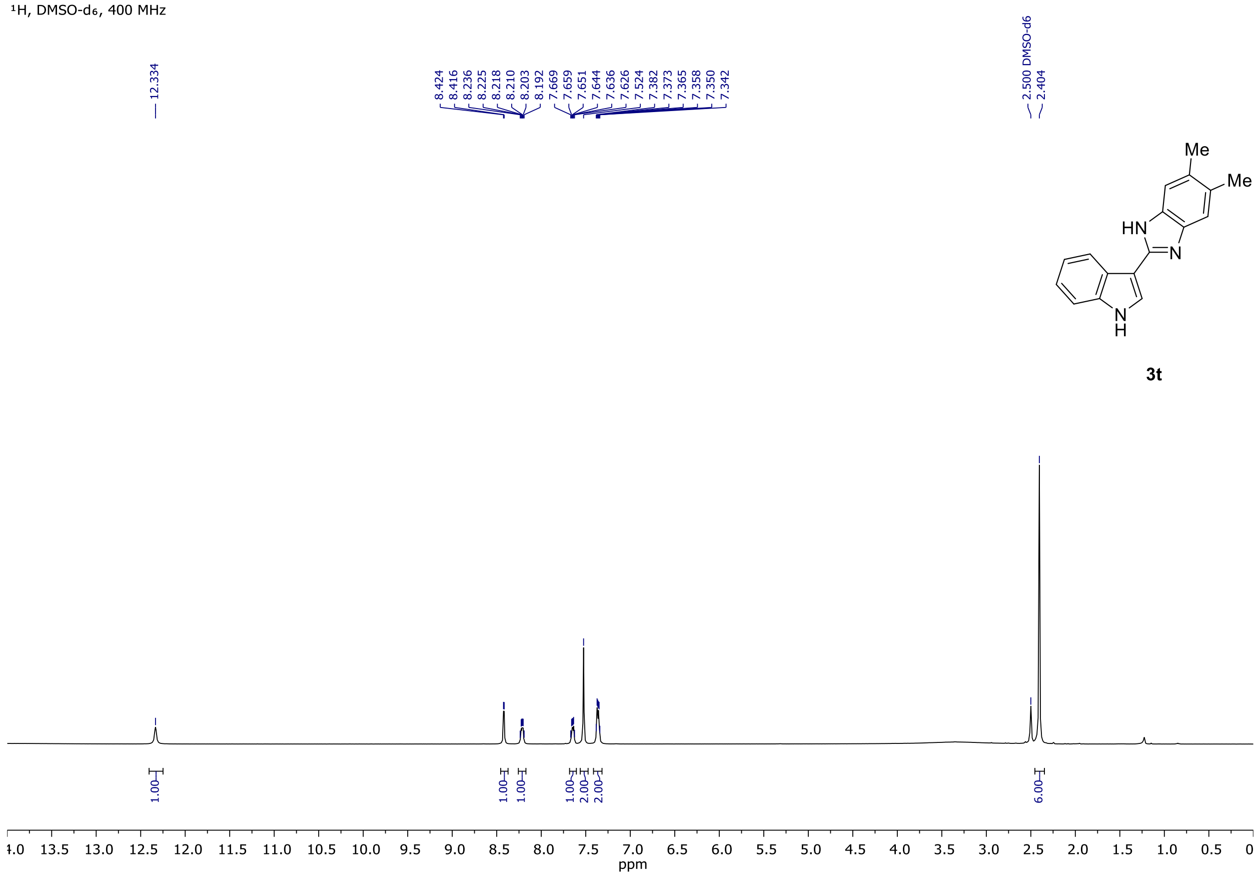

$^{13}C\{^1H\}$, DMSO-d₆, 100 MHz

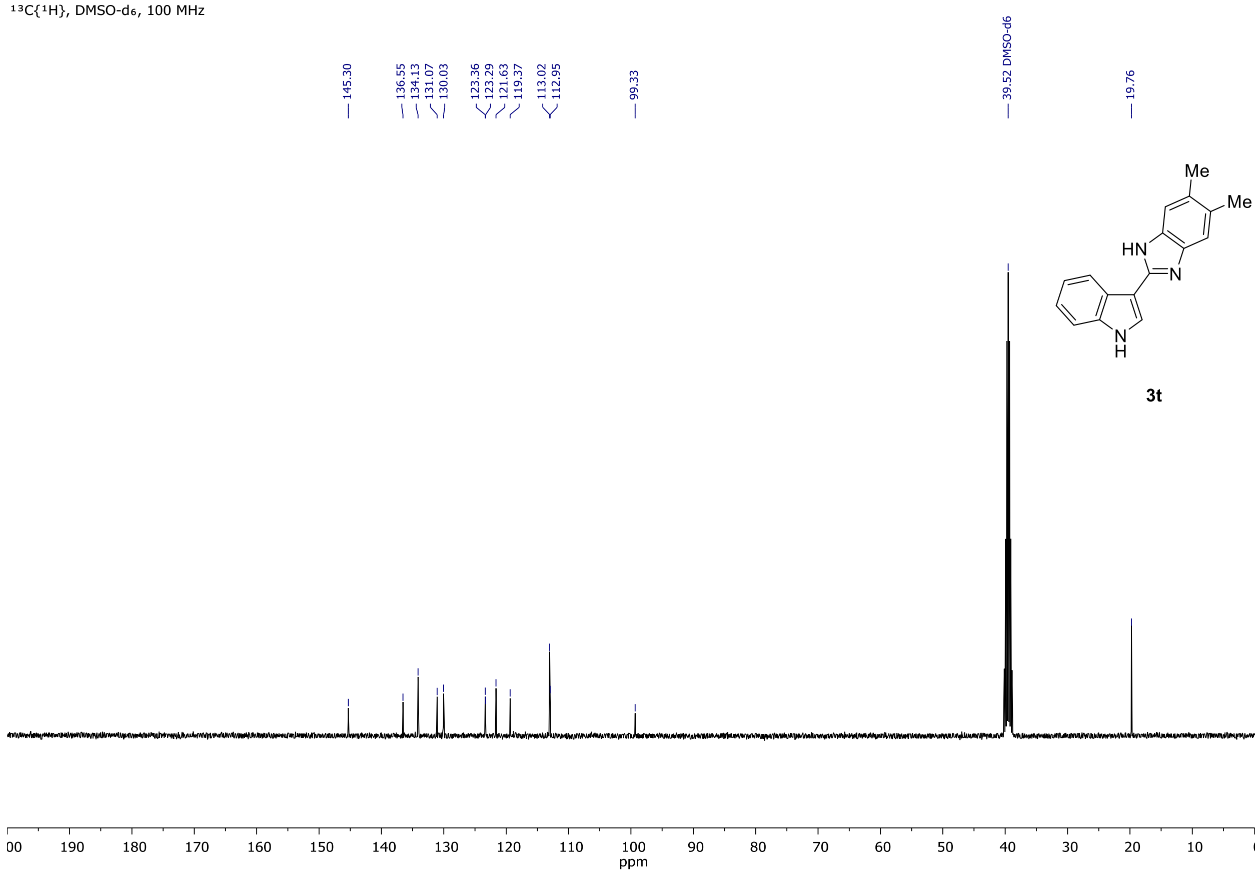

¹H, DMSO-d₆, 400 MHz

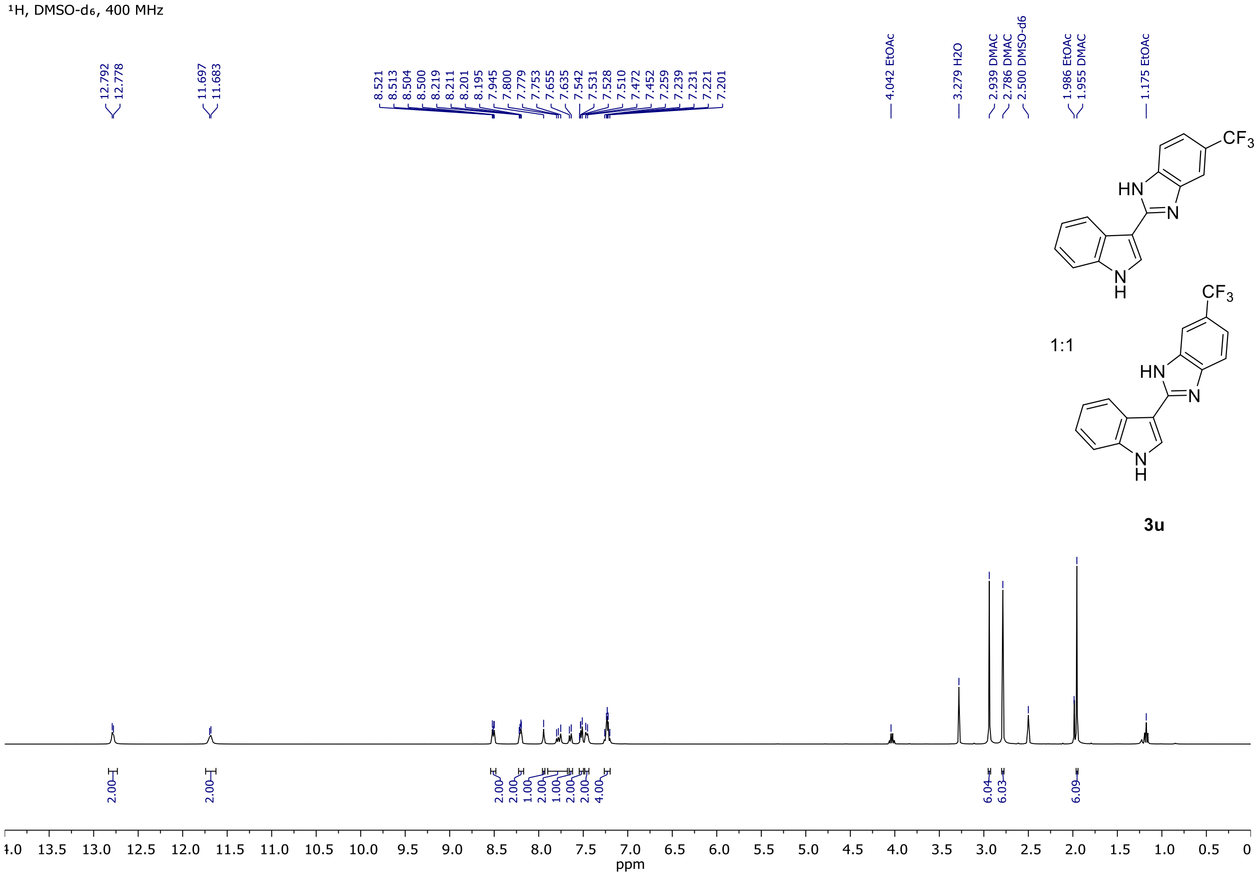

¹H, DMSO-d₆, 100 MHz

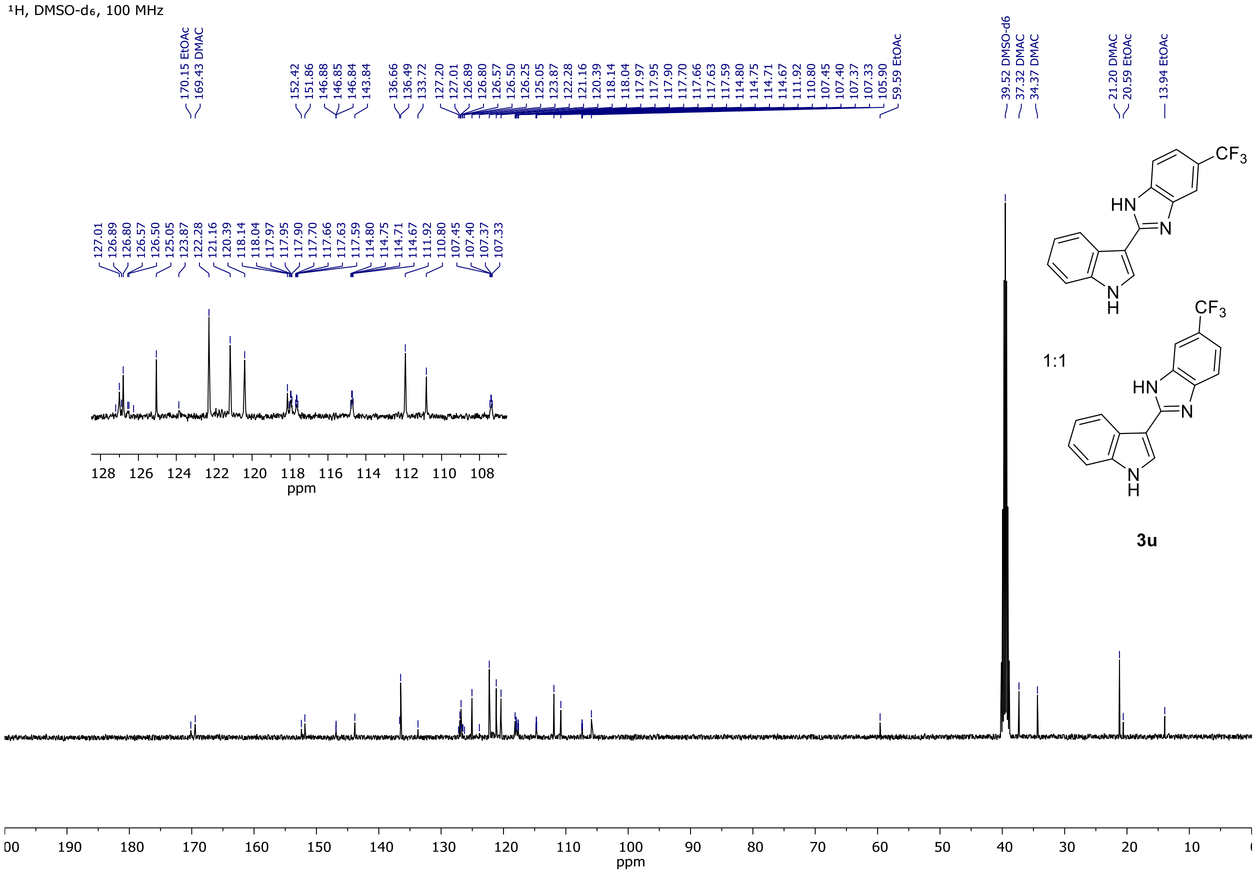

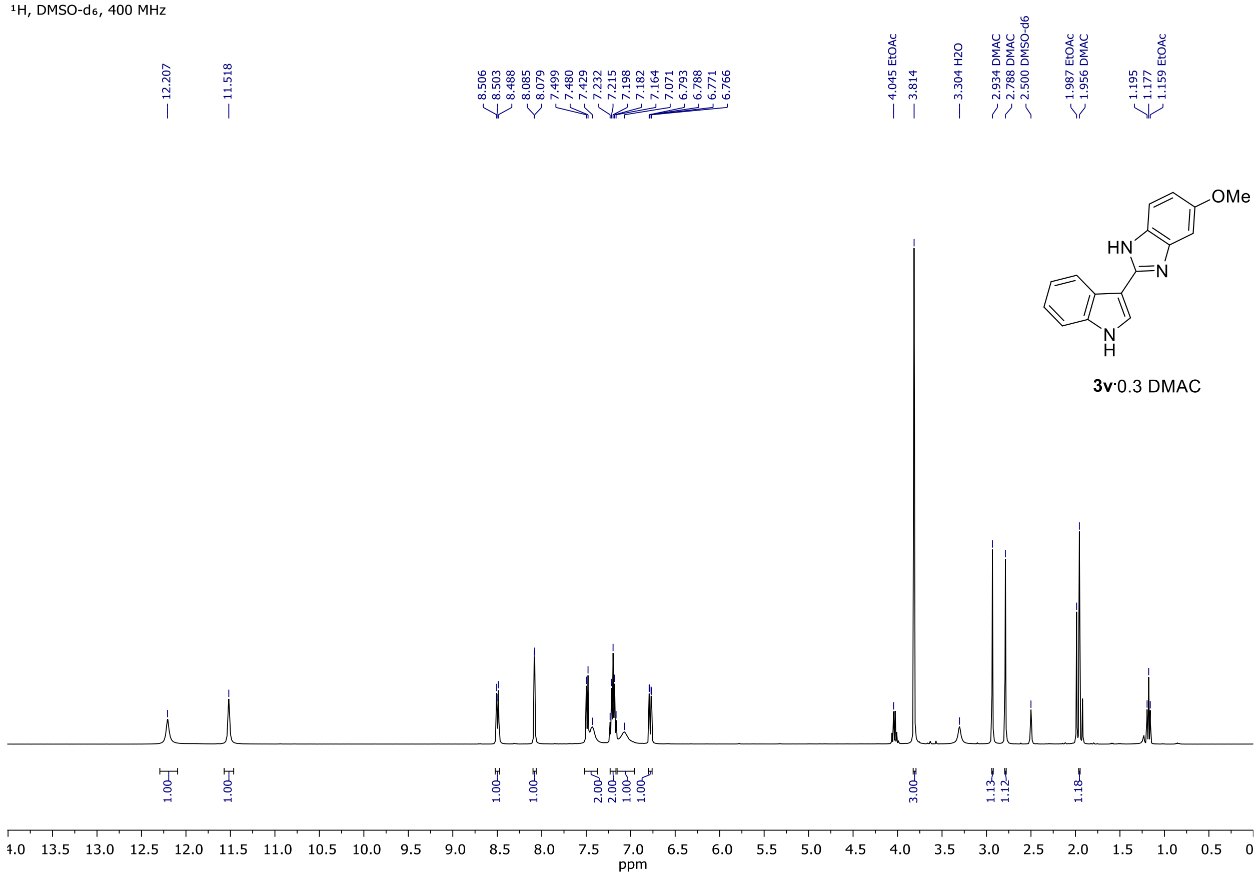
¹H, DMSO-d₆, 400 MHz
12.207
11.518
8.506
8.503
8.488
8.085
8.079
7.499
7.480
7.429
7.232
7.215
7.198
7.182
7.164
7.071
6.793
6.788
6.771
6.766
4.045 EtOAc
3.814
3.304 H2O
2.934 DMAC
2.788 DMAC
2.500 DMSO-d6
1.987 EtOAc
1.956 DMAC
1.195
1.177
1.159 EtOAc
OMe
HN
N
N
H
3v·0.3 DMAC
1.00
1.00
1.00
1.00
2.00
2.00
1.00
1.00
3.00
1.13
1.12
1.18
ppm

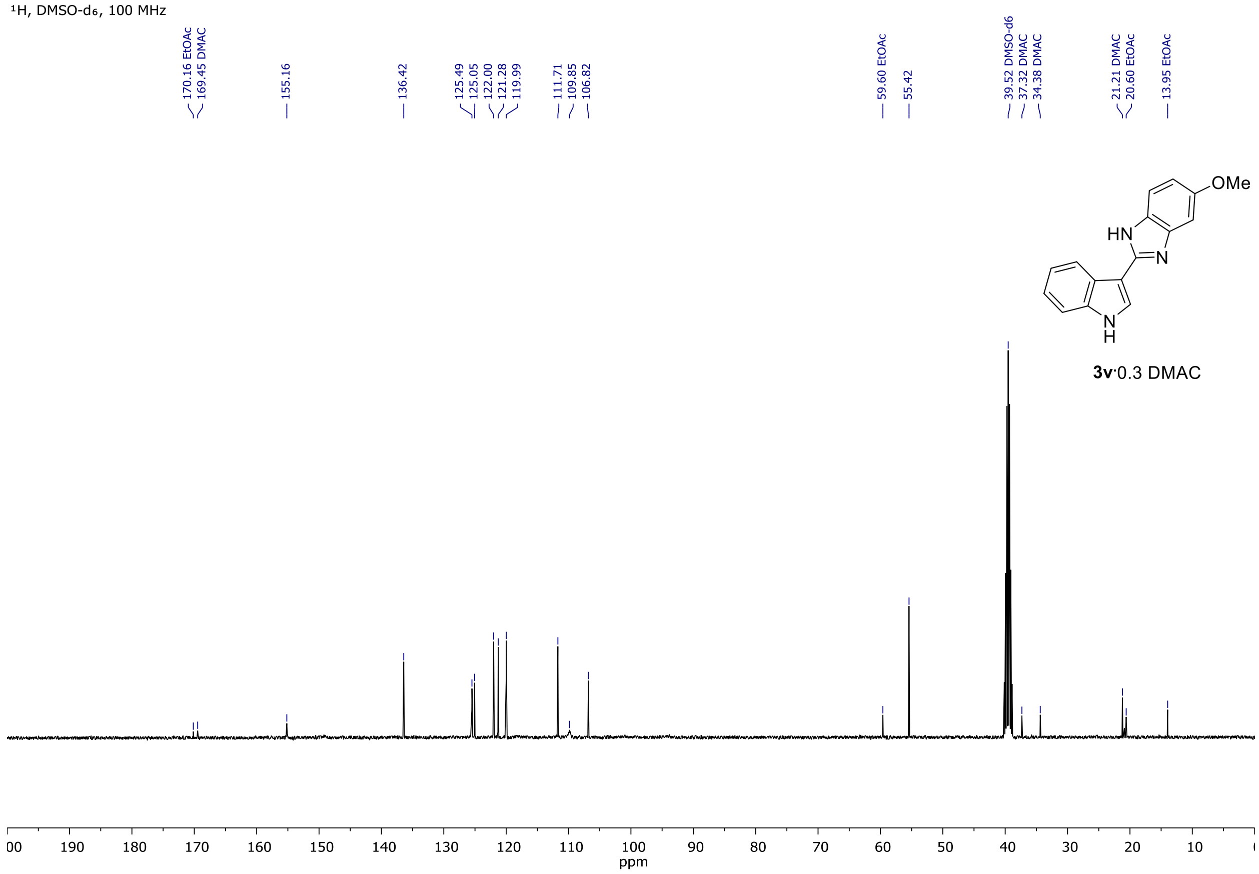
¹H, DMSO-d₆, 100 MHz
170.16 EtOAc
169.45 DMAC
155.16
136.42
125.49
125.05
122.00
121.28
119.99
111.71
109.85
106.82
59.60 EtOAc
55.42
39.52 DMSO-d6
37.32 DMAC
34.38 DMAC
21.21 DMAC
20.60 EtOAc
13.95 EtOAc
OMe
HN
N
N
H
3v·0.3 DMAC
ppm

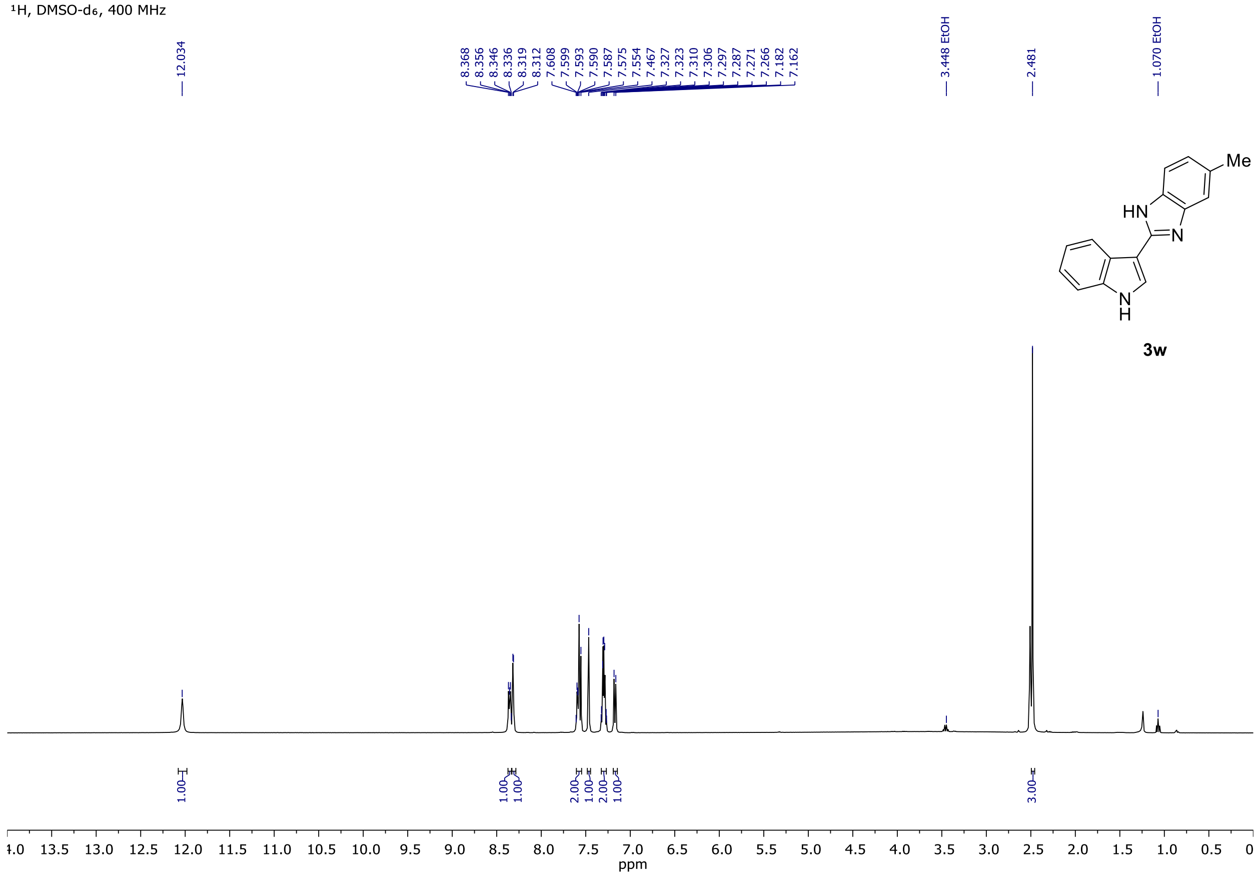
¹H, DMSO-d₆, 400 MHz
12.034
8.368
8.356
8.346
8.336
8.319
8.312
7.608
7.599
7.593
7.590
7.587
7.575
7.554
7.467
7.327
7.323
7.310
7.306
7.297
7.287
7.271
7.266
7.182
7.162
3.448 EtOH
2.481
1.070 EtOH
Me
HN
N
N
H
3w
1.00
1.00
1.00
2.00
1.00
2.00
1.00
3.00
ppm

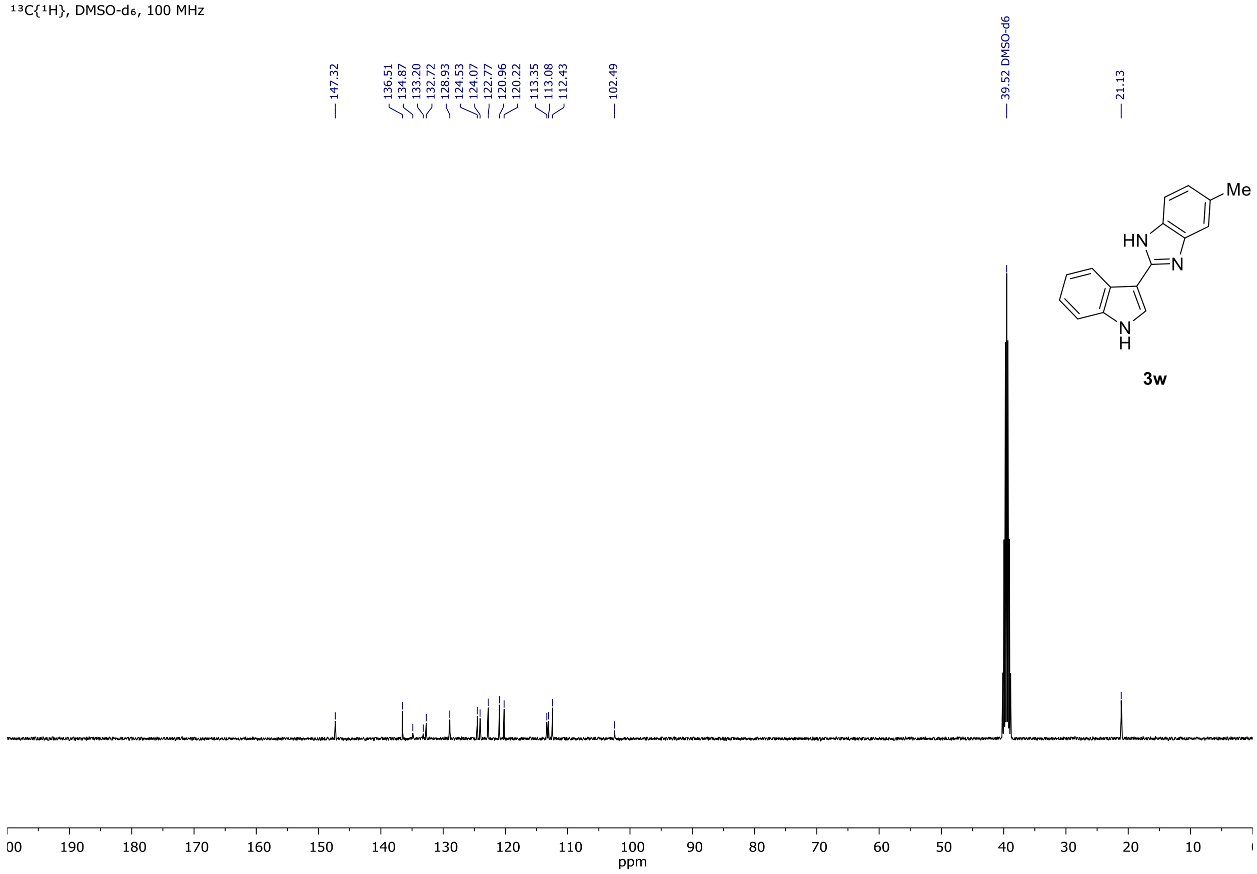
¹³C{¹H}, DMSO-d₆, 100 MHz
147.32
136.51
134.87
133.20
132.72
128.93
124.53
124.07
122.77
120.96
120.22
113.35
113.08
112.43
102.49
39.52 DMSO-d6
21.13
Me
HN
N
N
H
3w
ppm

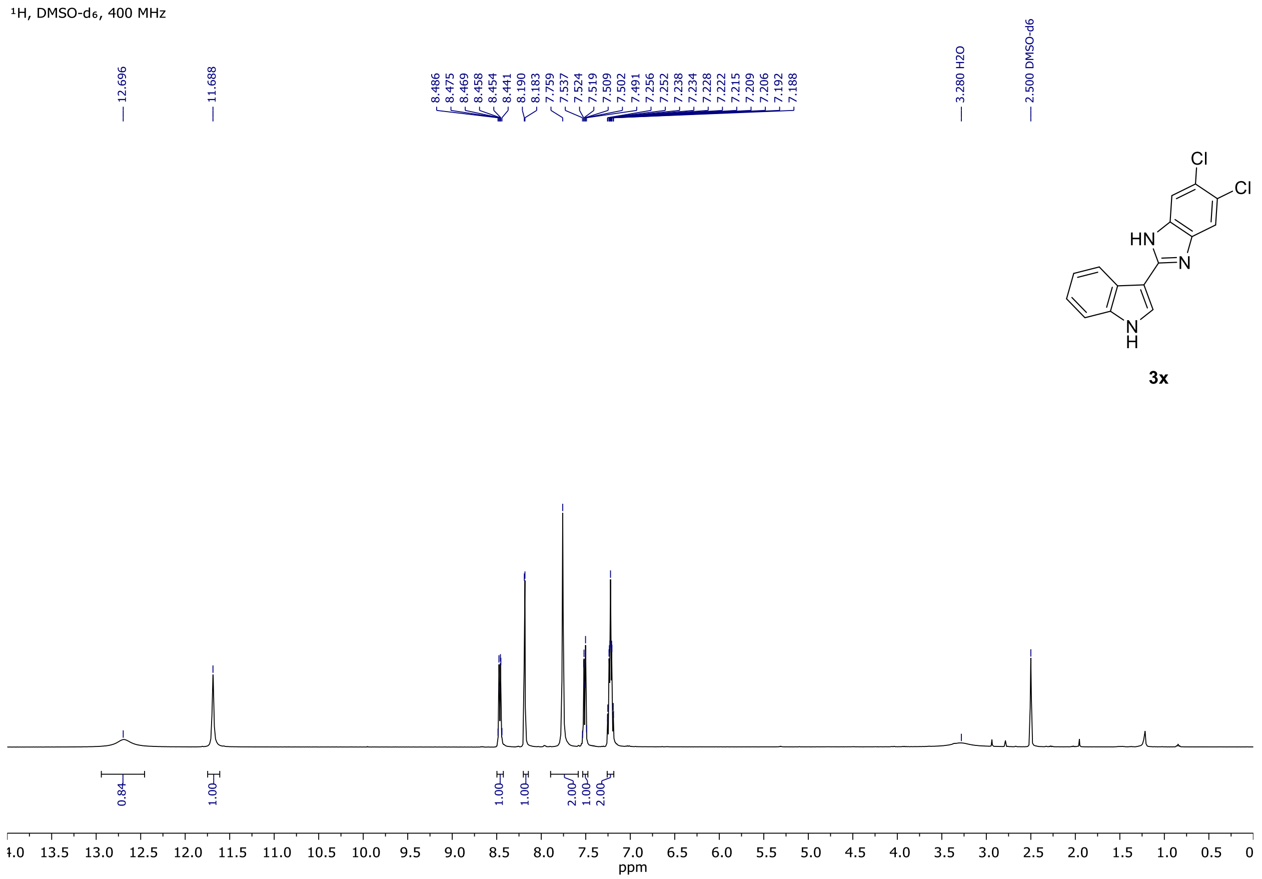
¹H, DMSO-d₆, 400 MHz
12.696
11.688
8.486 8.475 8.469 8.458 8.454 8.441 8.190 8.183 7.759 7.537 7.524 7.519 7.509 7.502 7.491 7.256 7.252 7.238 7.234 7.228 7.222 7.215 7.209 7.206 7.192 7.188
3.280 H2O
2.500 DMSO-d6
Cl
Cl
HN
N
N
H
3x
0.84
1.00
1.00
1.00
2.00
1.00
2.00
ppm

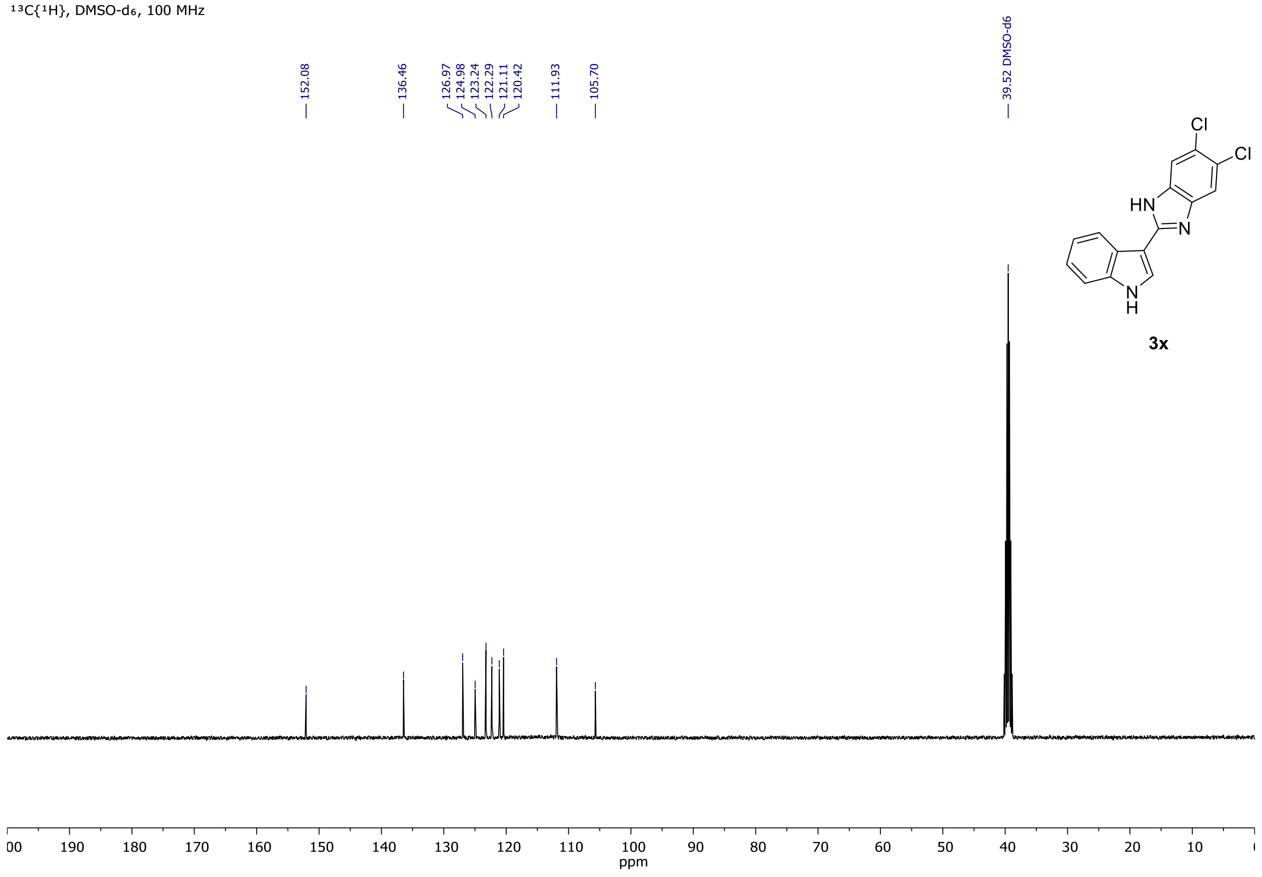
¹³C{¹H}, DMSO-d₆, 100 MHz
152.08
136.46
126.97 124.98 123.24 122.29 121.11 120.42
111.93
105.70
39.52 DMSO-d6
Cl
Cl
HN
N
N
H
3x
ppm

¹H, DMSO-d₆, 400 MHz

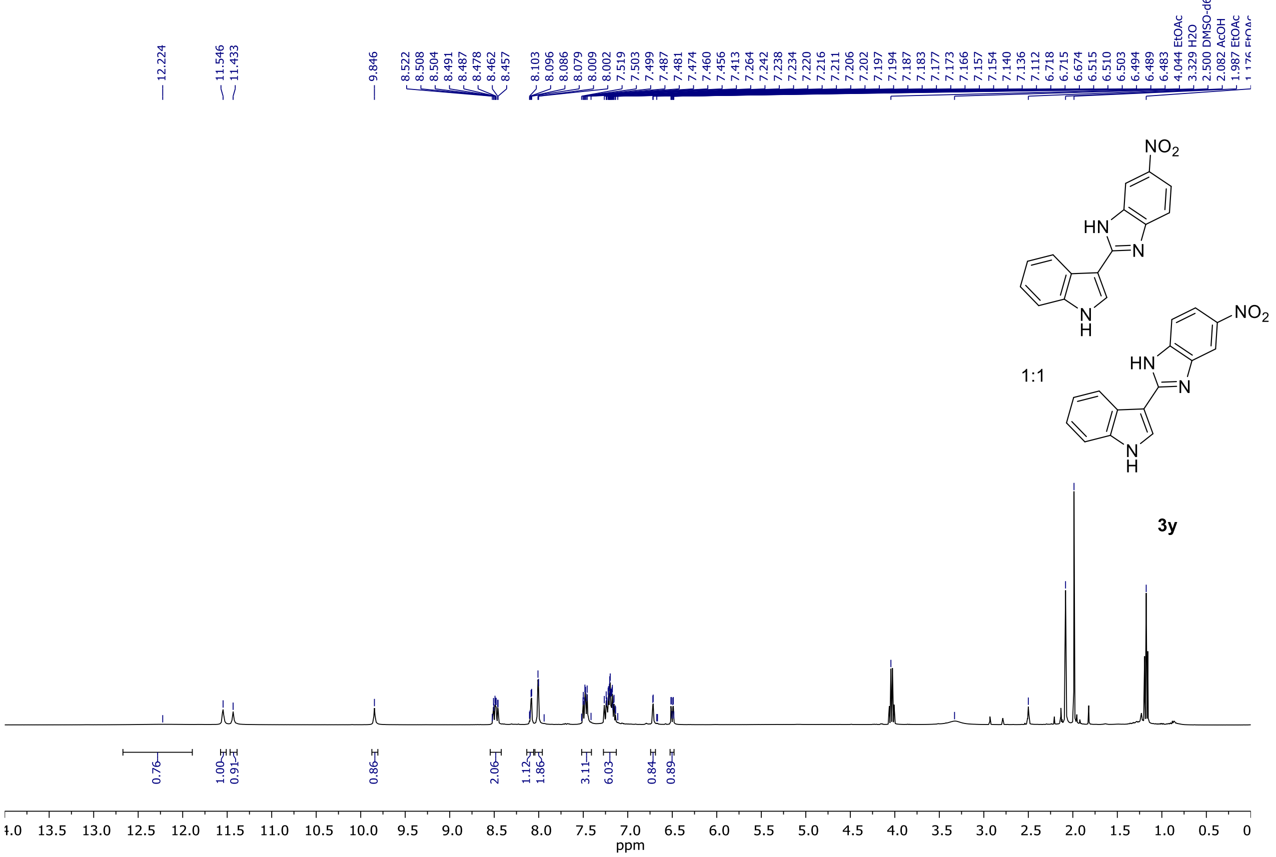

¹³C{¹H}, DMSO-d₆, 100 MHz

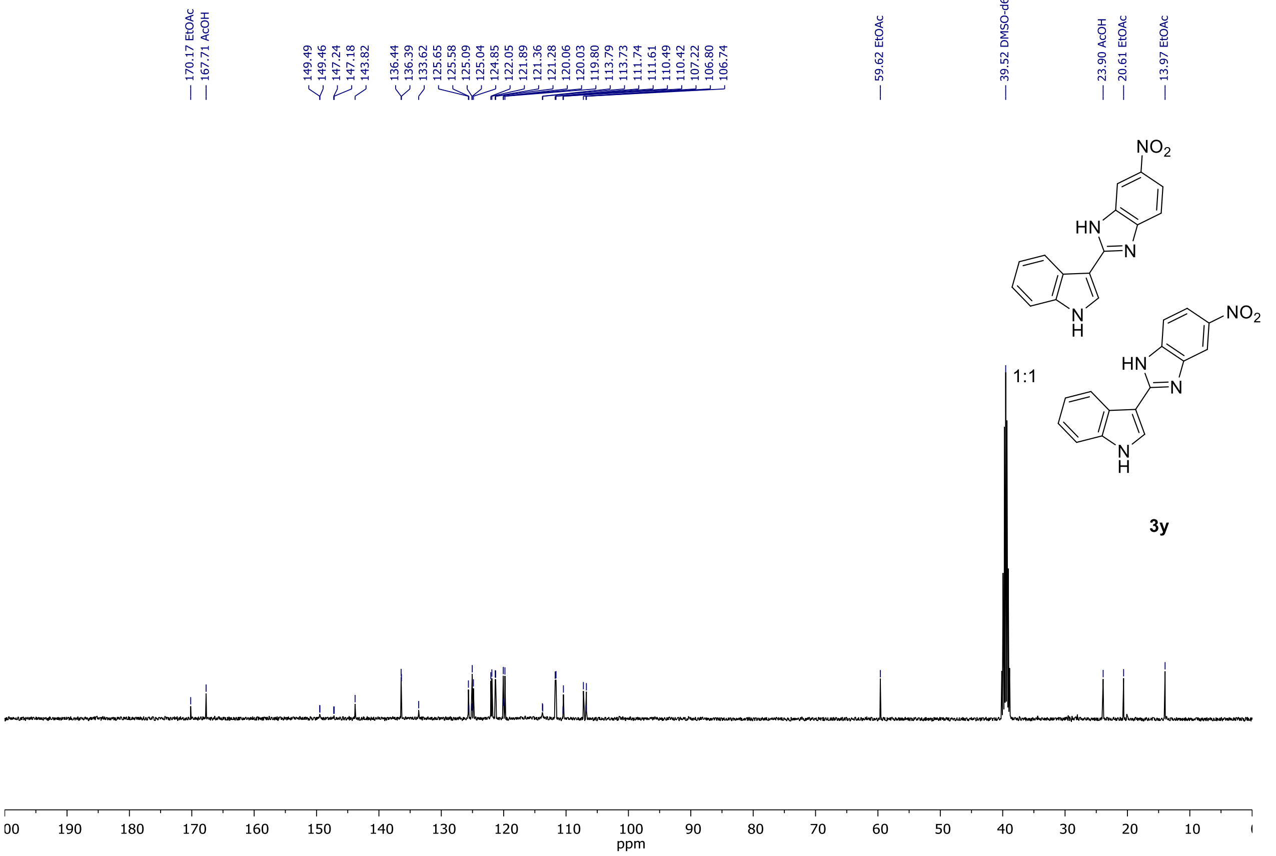

¹H, DMSO-d₆, 400 MHz

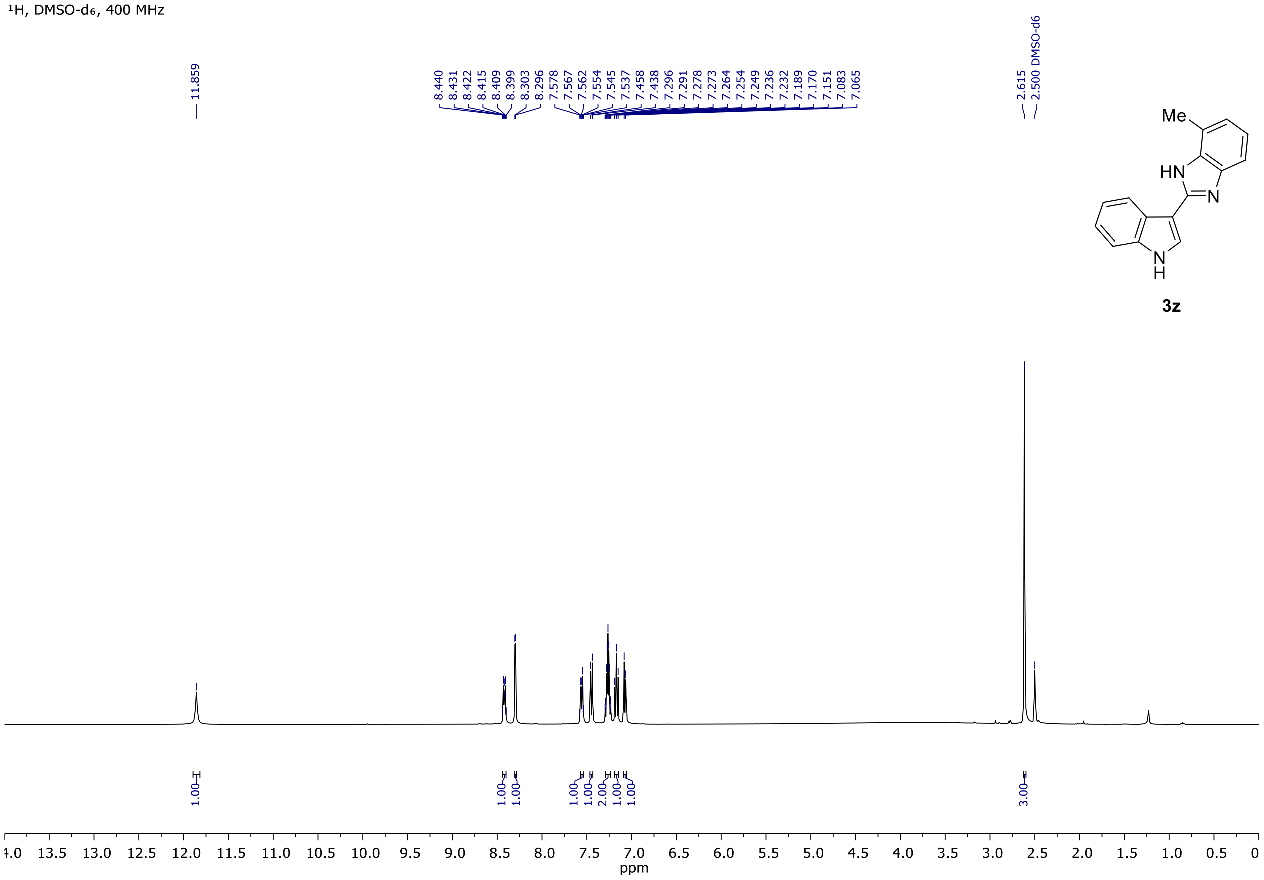

¹³C{¹H}, DMSO-d₆, 100 MHz

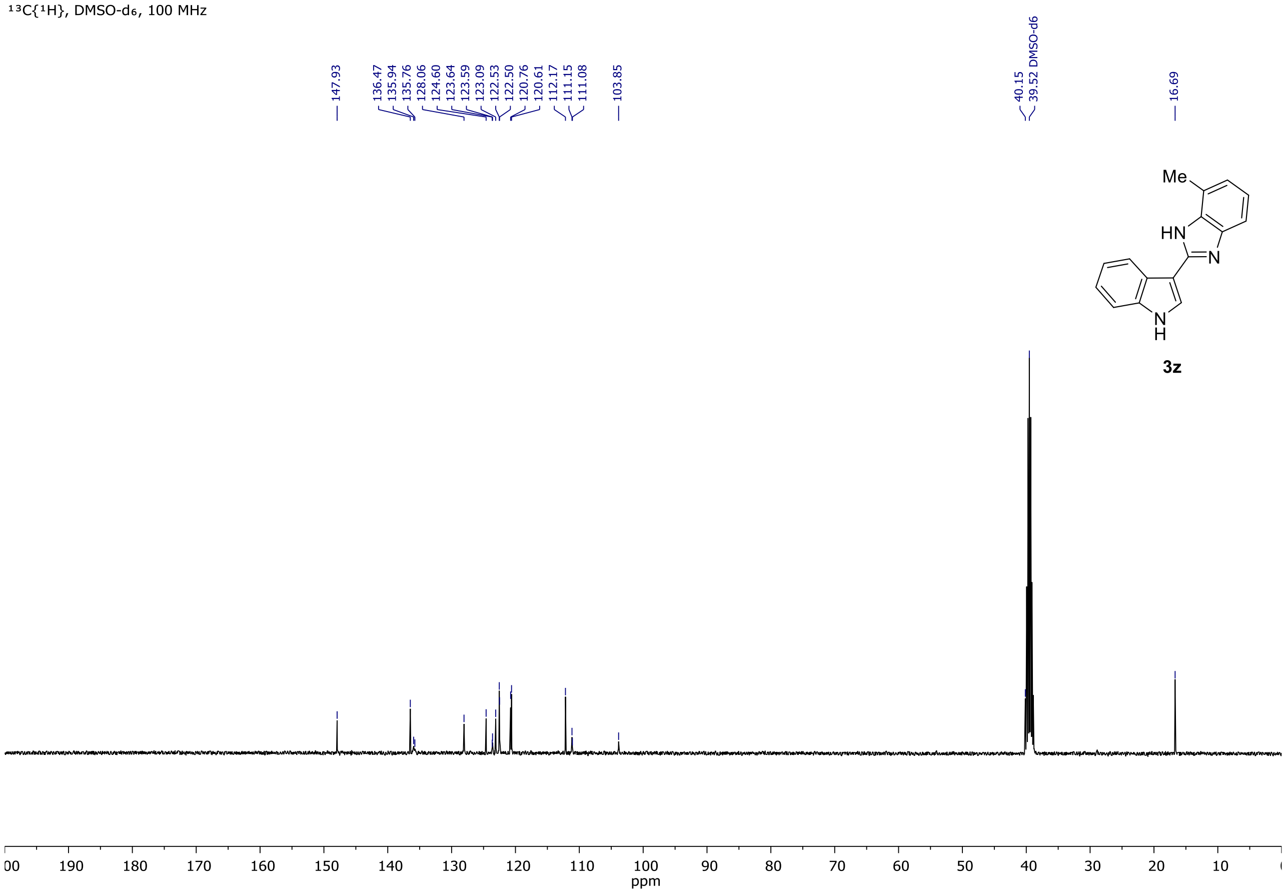

^1H, DMSO-d6, 400 MHz

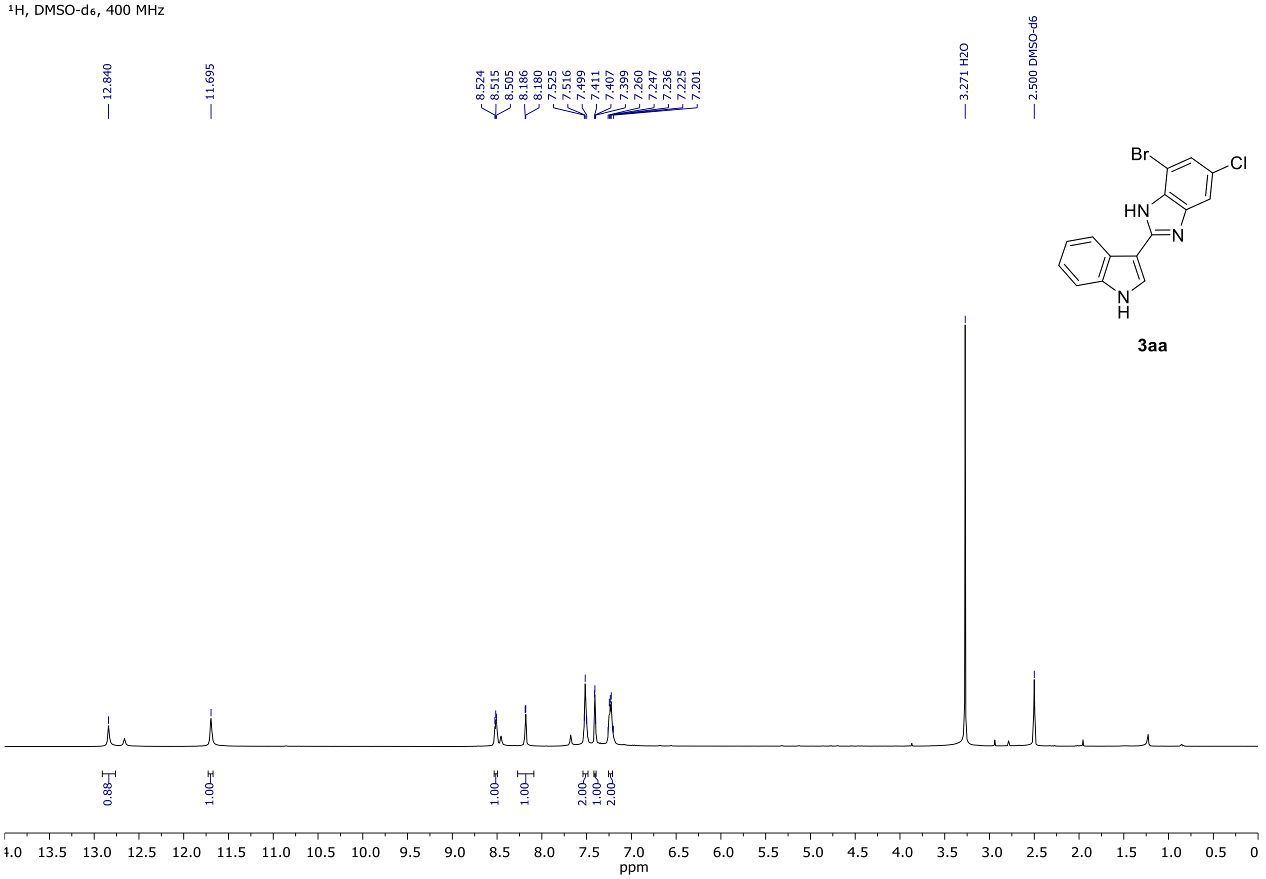

$^{13}C\{^1H\}$, DMSO-d6, 100 MHz

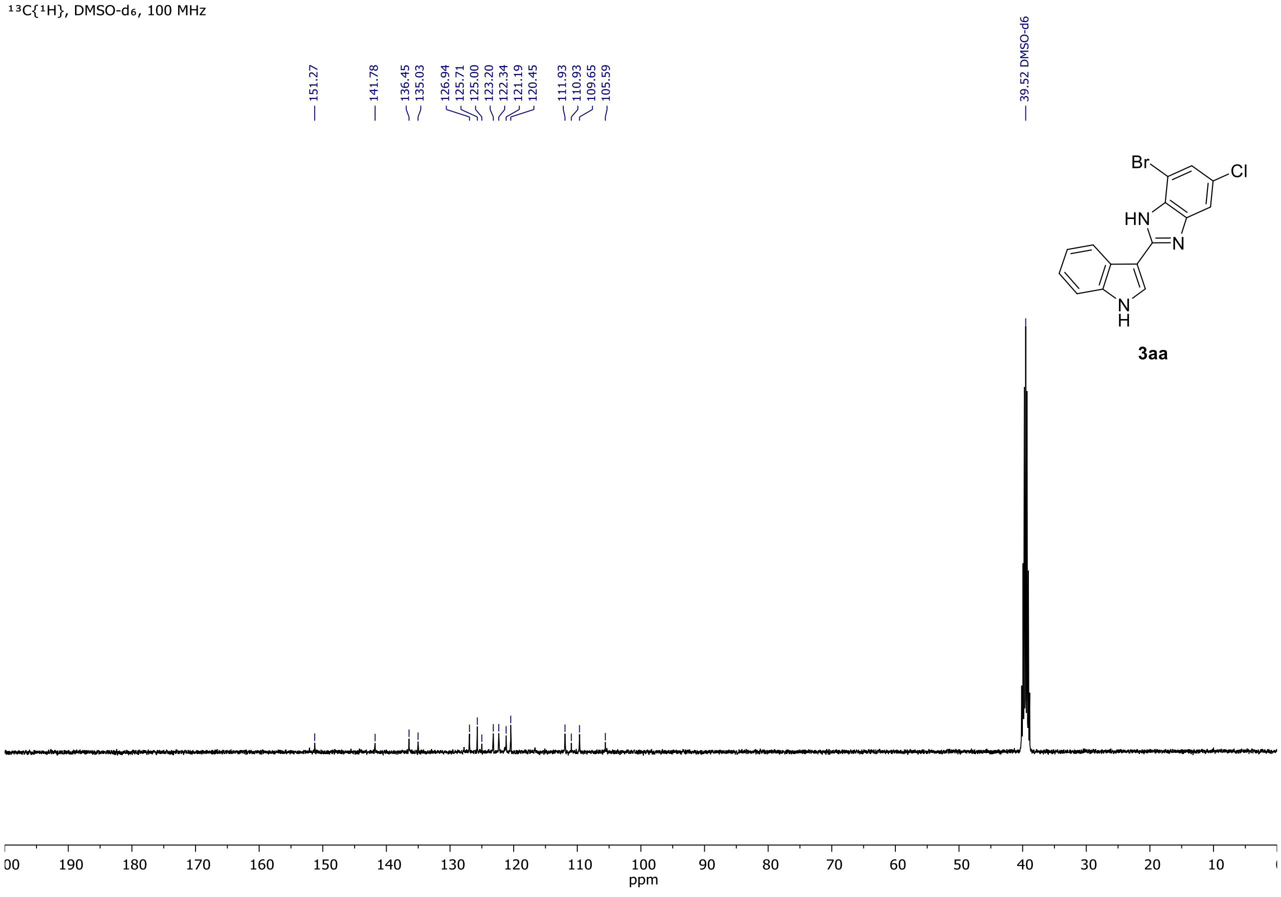

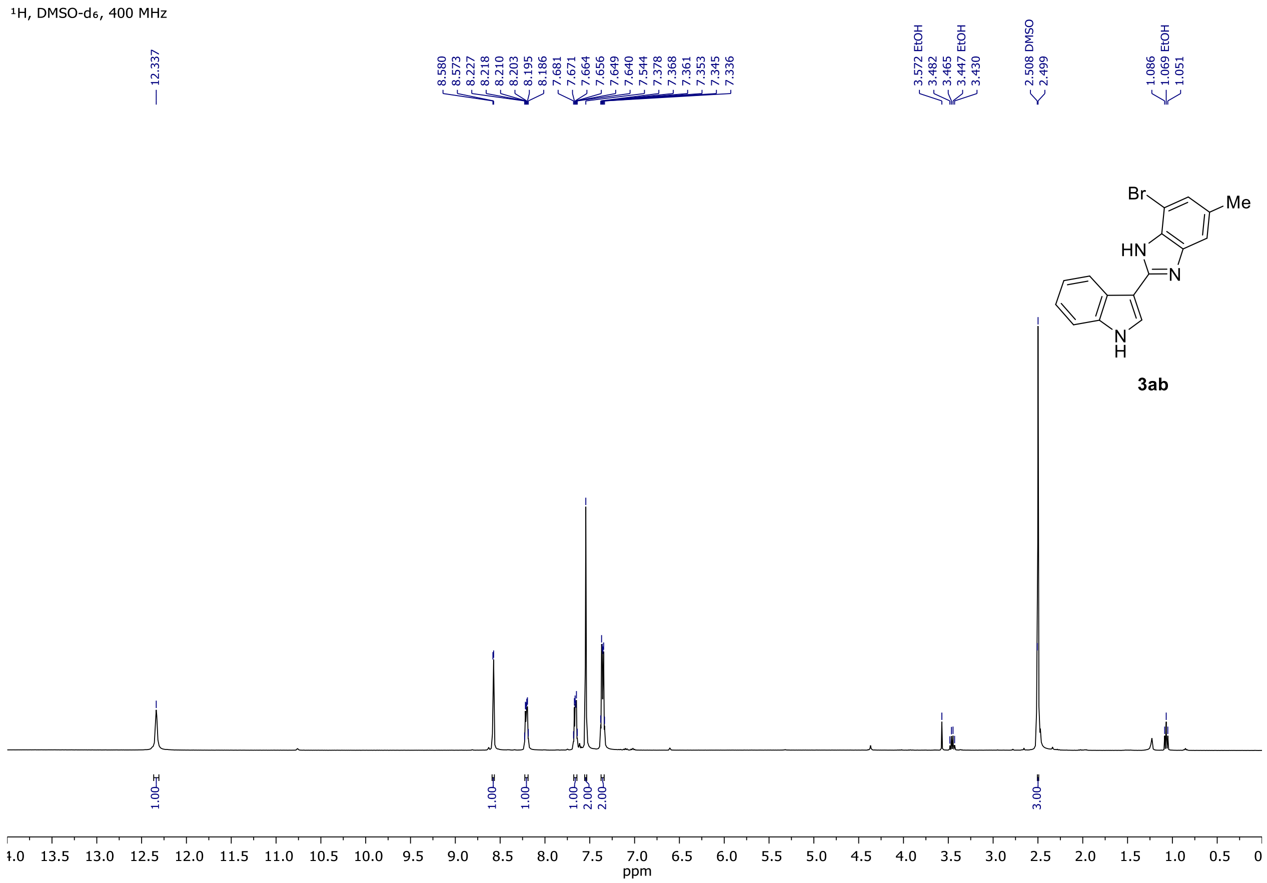
¹H, DMSO-d₆, 400 MHz
3ab
ppm

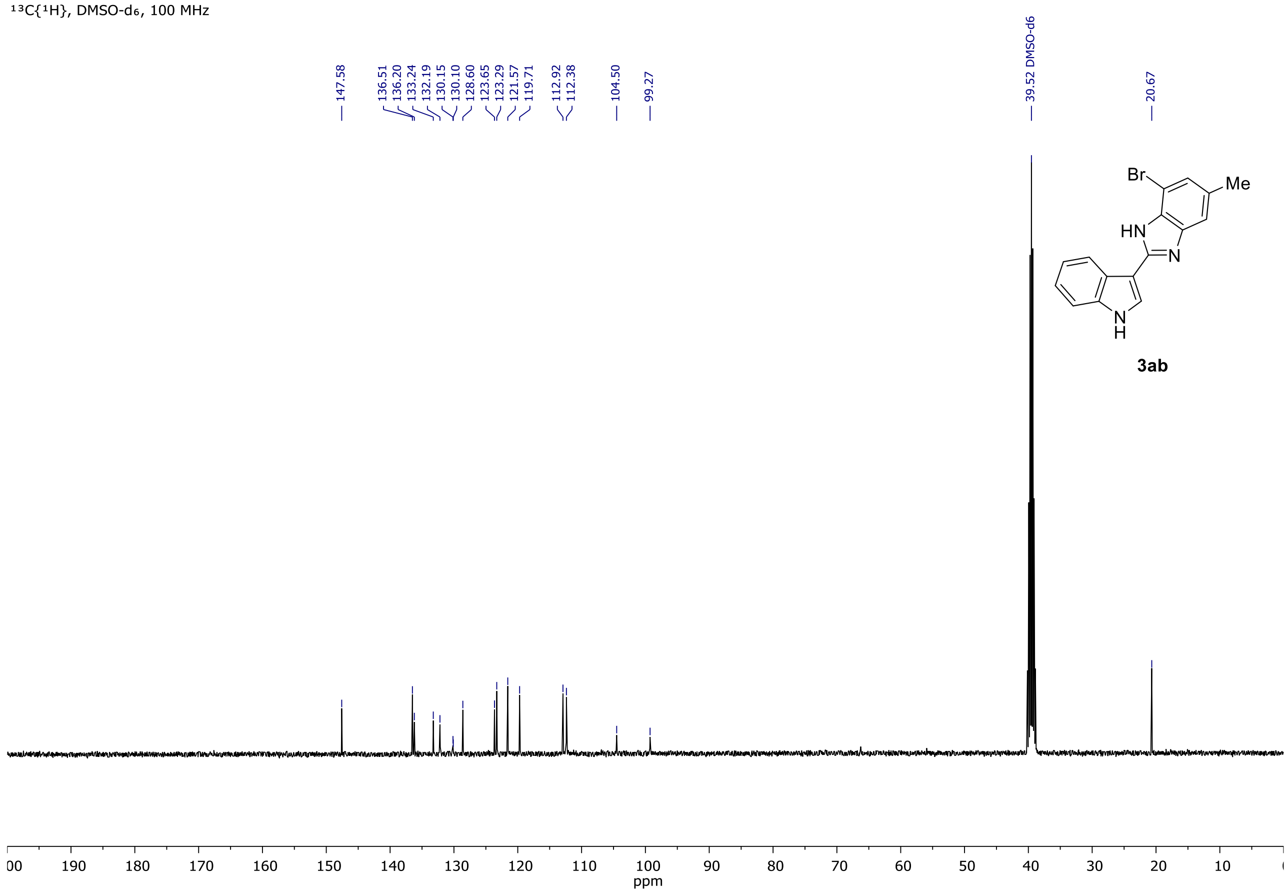
¹³C{¹H}, DMSO-d₆, 100 MHz
3ab
ppm

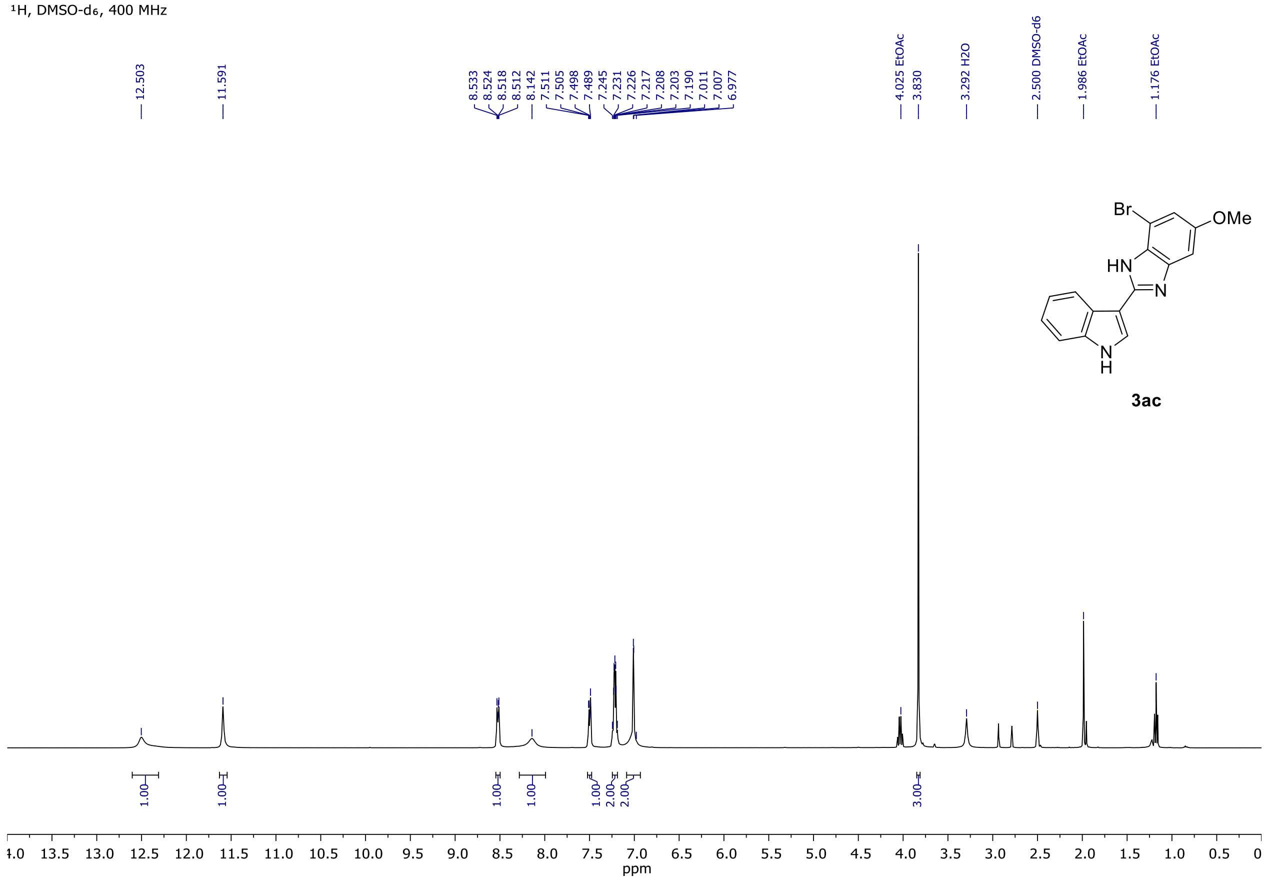
¹H, DMSO-d₆, 400 MHz
12.503
11.591
8.533
8.524
8.518
8.512
8.142
7.511
7.505
7.498
7.489
7.245
7.231
7.226
7.217
7.208
7.203
7.190
7.011
7.007
6.977
4.025 EtOAc
3.830
3.292 H2O
2.500 DMSO-d6
1.986 EtOAc
1.176 EtOAc
Br
OMe
HN
N
N
H
3ac
1.00
1.00
1.00
1.00
1.00
2.00
2.00
3.00
ppm

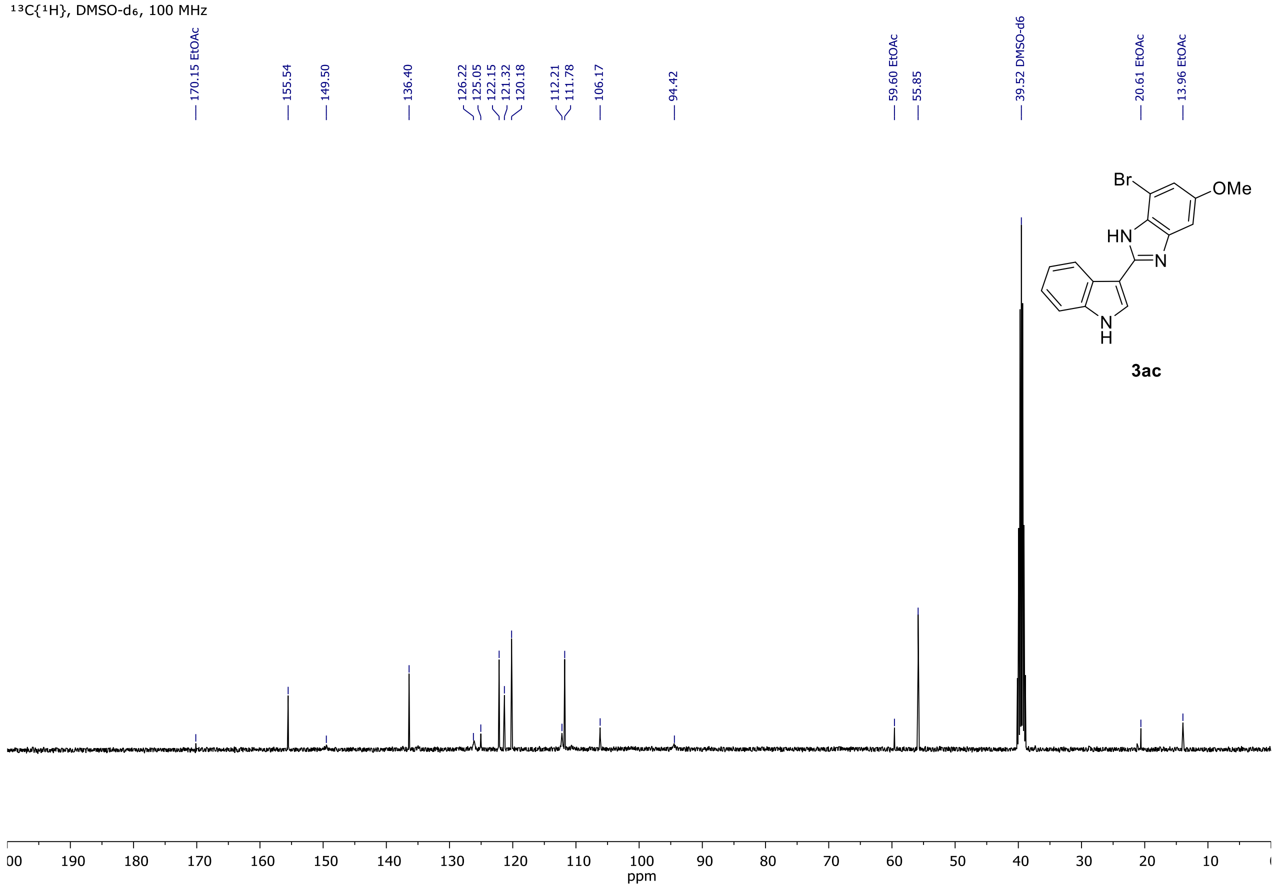
¹³C{¹H}, DMSO-d₆, 100 MHz
170.15 EtOAc
155.54
149.50
136.40
126.22
125.05
122.15
121.32
120.18
112.21
111.78
106.17
94.42
59.60 EtOAc
55.85
39.52 DMSO-d6
20.61 EtOAc
13.96 EtOAc
Br
OMe
HN
N
N
H
3ac
ppm

^{1}H, DMSO-d_6, 400 MHz

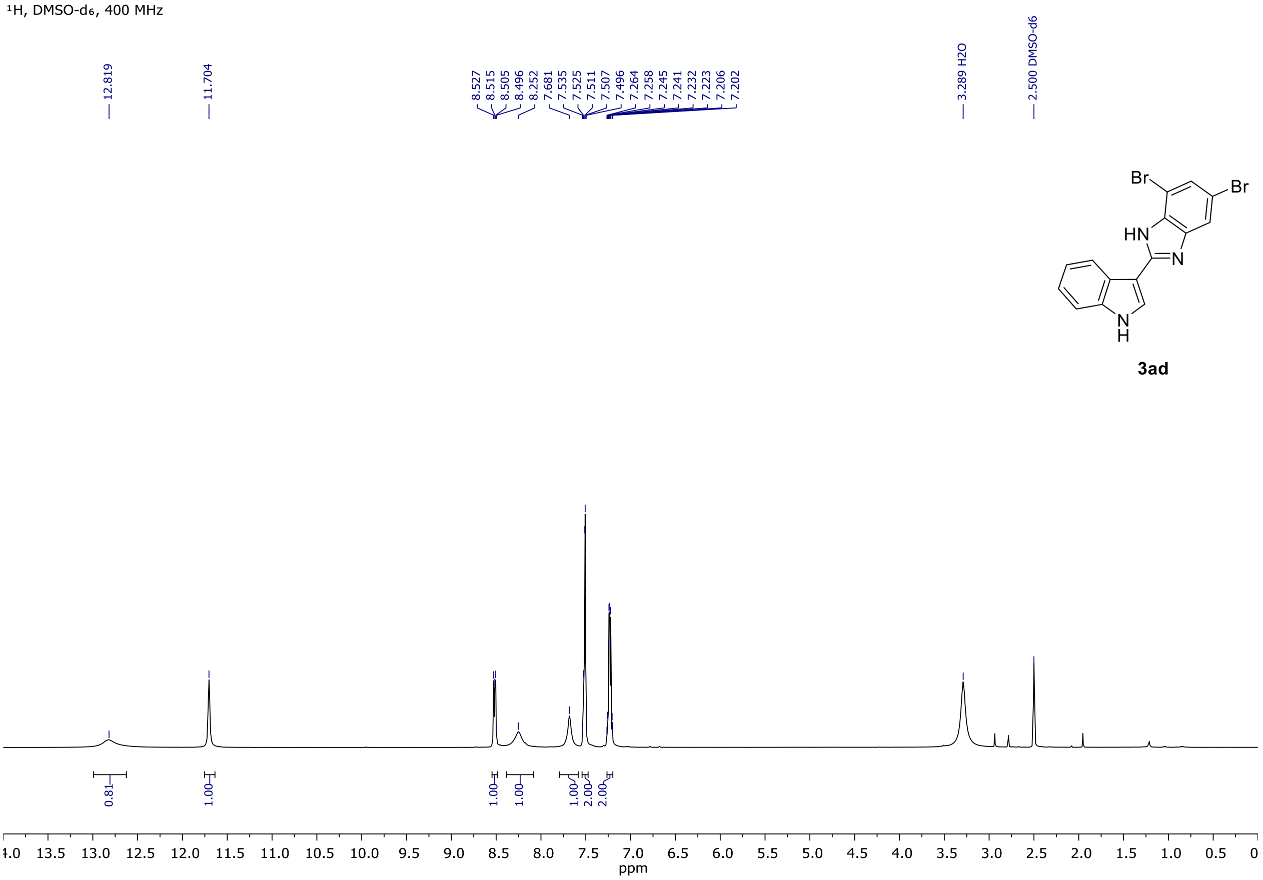

^{13}C{^{1}H}, DMSO-d_6, 100 MHz

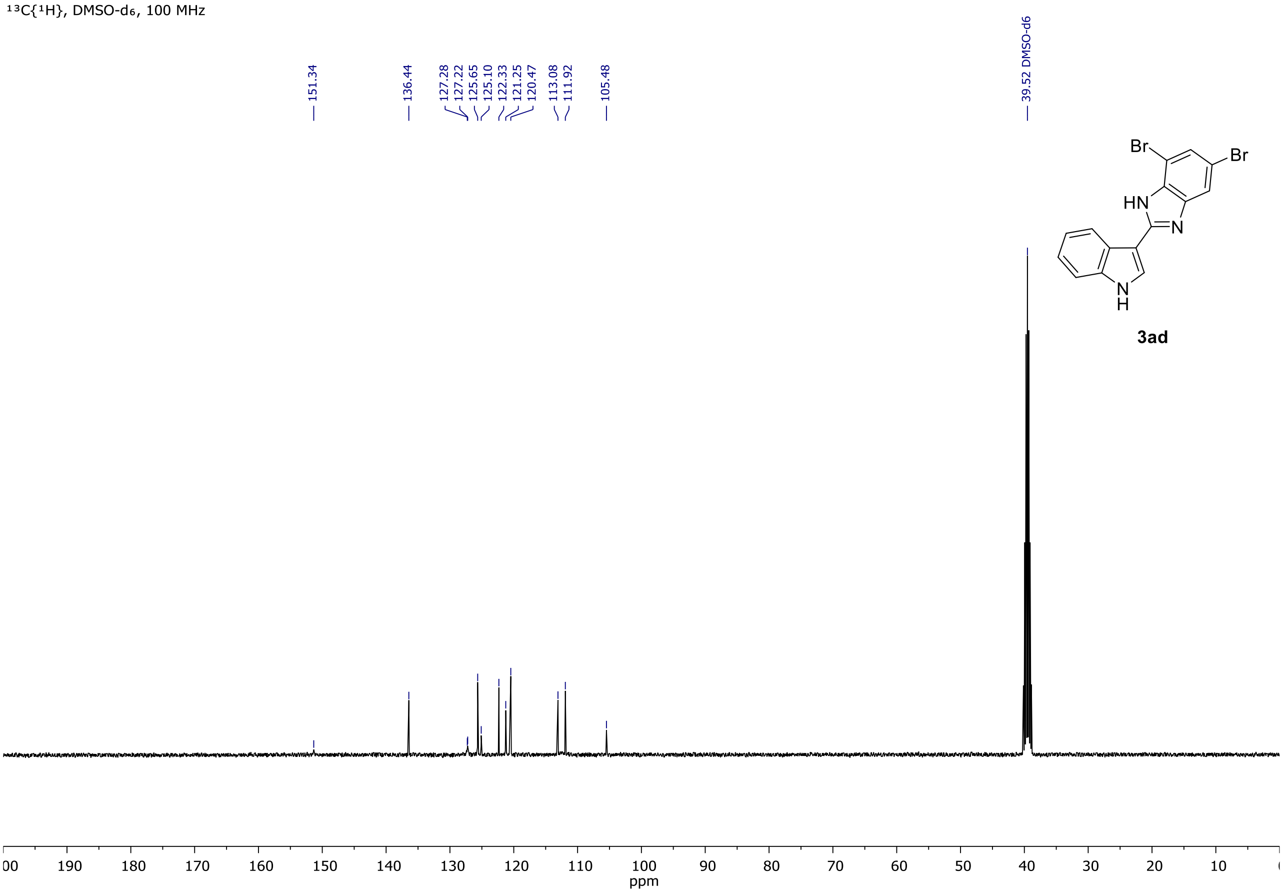

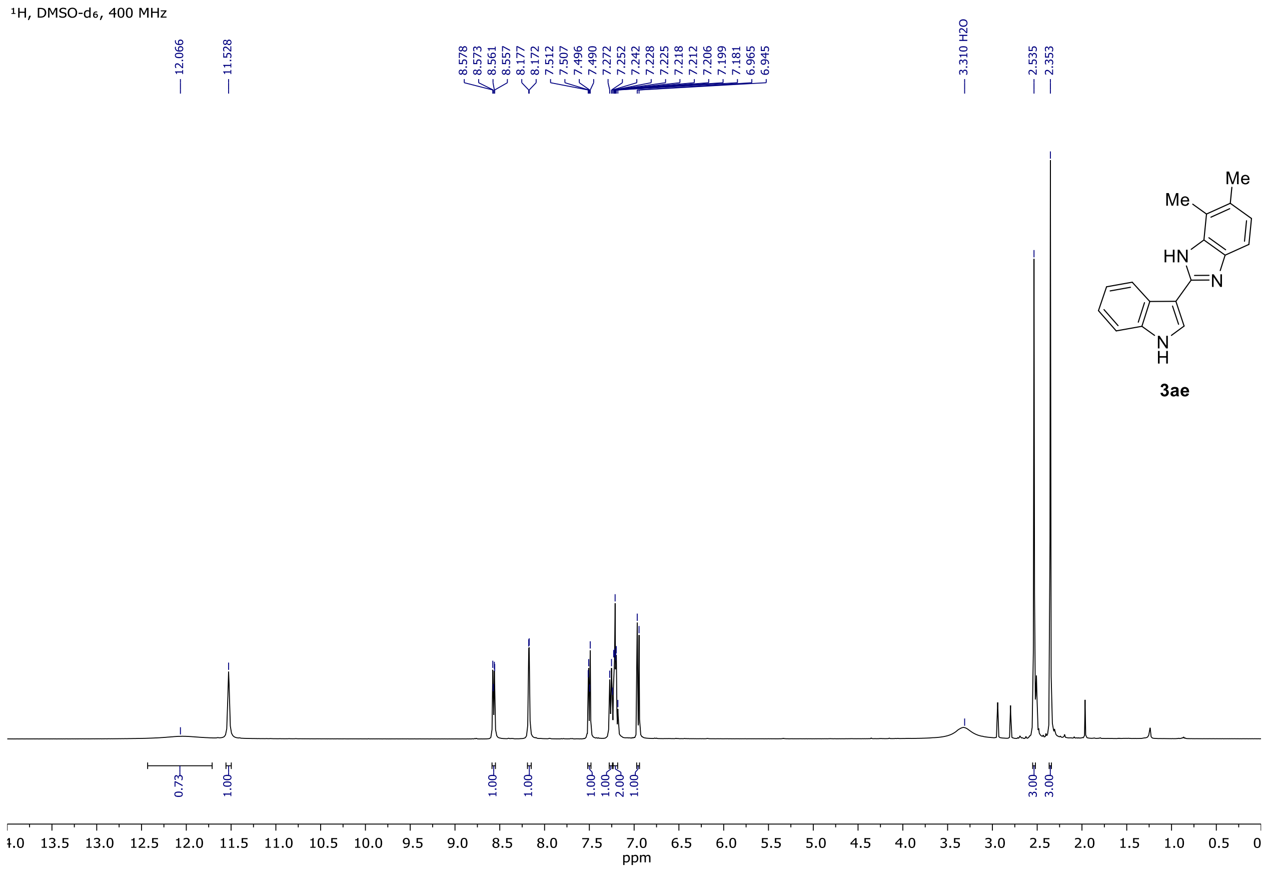
¹H, DMSO-d₆, 400 MHz
12.066
11.528
8.578
8.573
8.561
8.557
8.177
8.172
7.512
7.507
7.496
7.490
7.272
7.252
7.242
7.228
7.225
7.218
7.212
7.206
7.199
7.181
6.965
6.945
3.310 H2O
2.535
2.353
0.73
1.00
1.00
1.00
1.00
1.00
2.00
1.00
3.00
3.00
ppm
Me
Me
HN
N
N
H
3ae

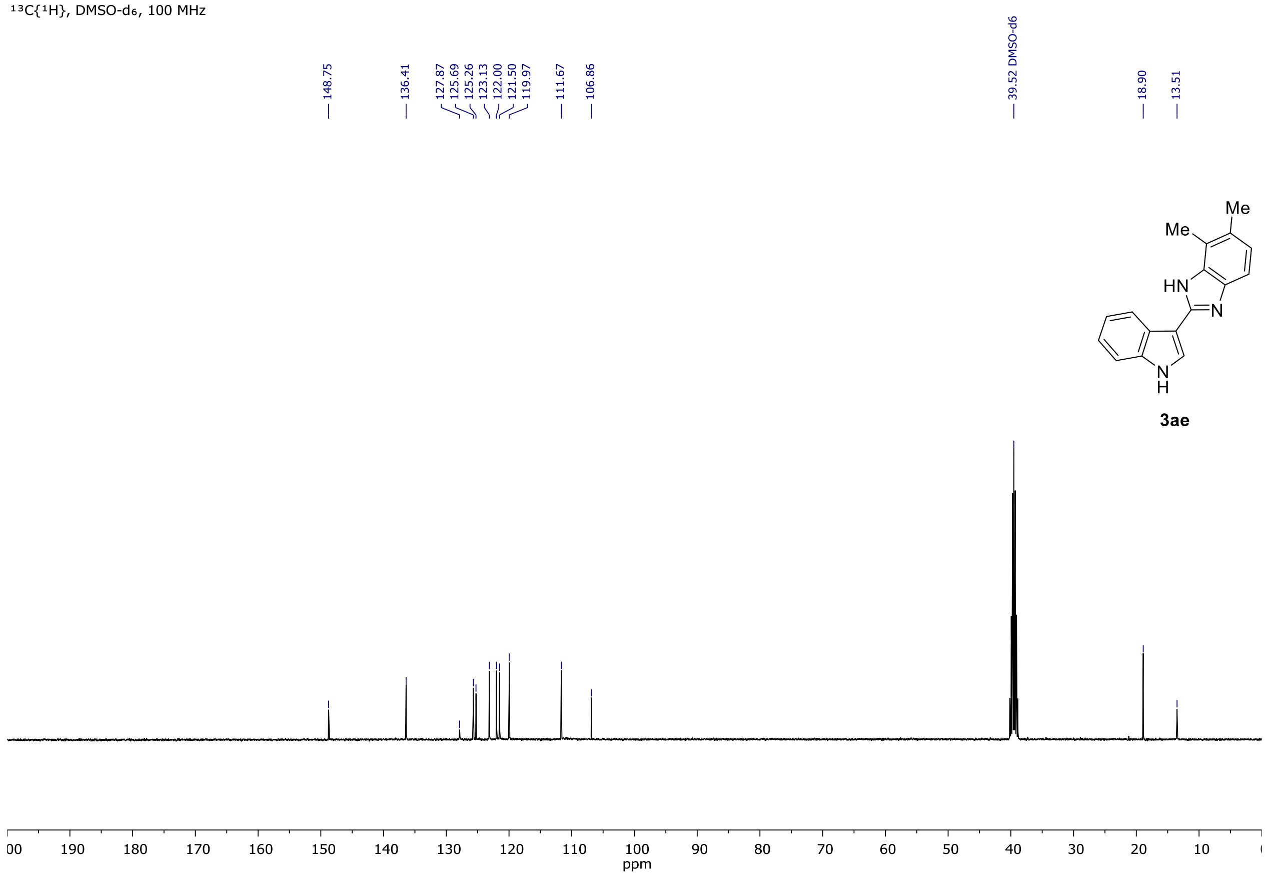
¹³C{¹H}, DMSO-d₆, 100 MHz
148.75
136.41
127.87
125.69
125.26
123.13
122.00
121.50
119.97
111.67
106.86
39.52 DMSO-d6
18.90
13.51
ppm
Me
Me
HN
N
N
H
3ae

¹H, DMSO-d₆, 400 MHz

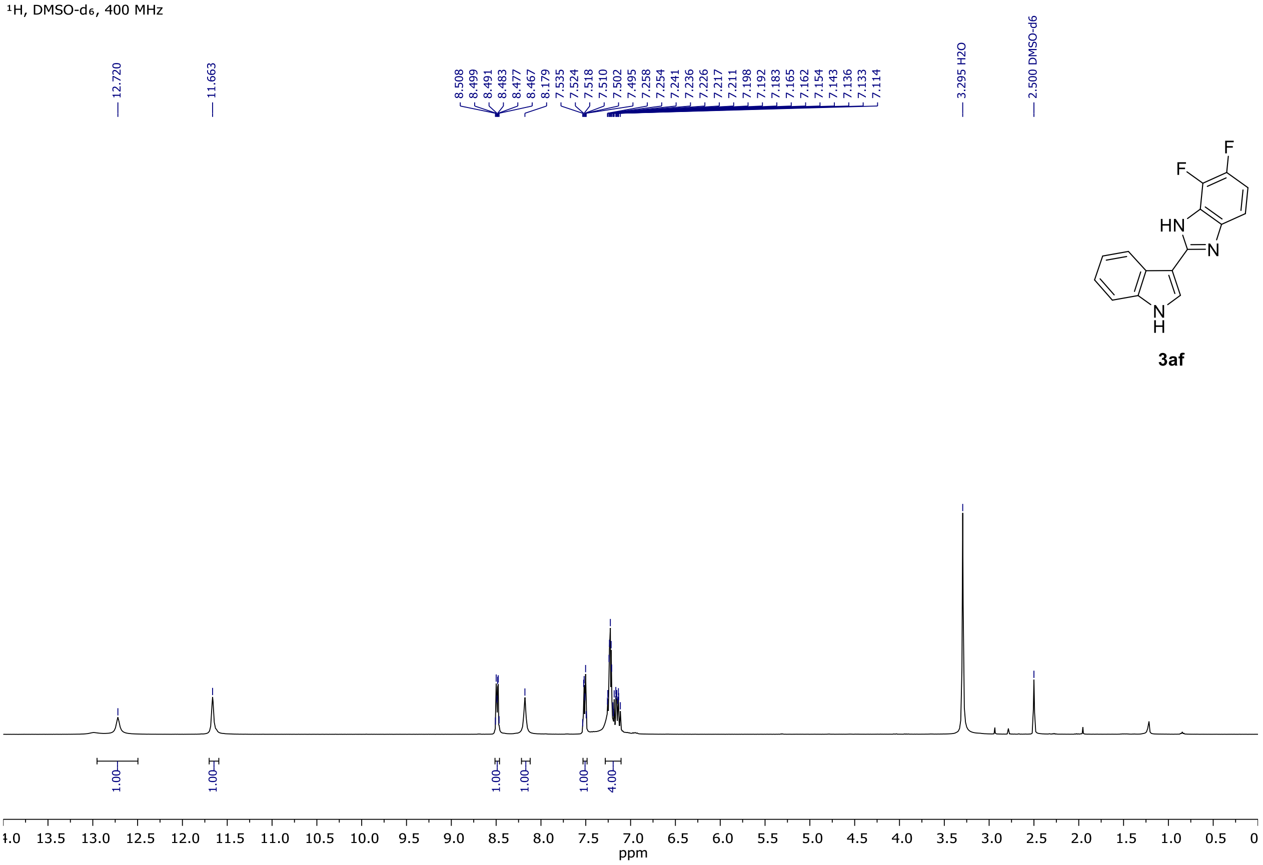

3af

¹³C{¹H}, DMSO-d₆, 100 MHz

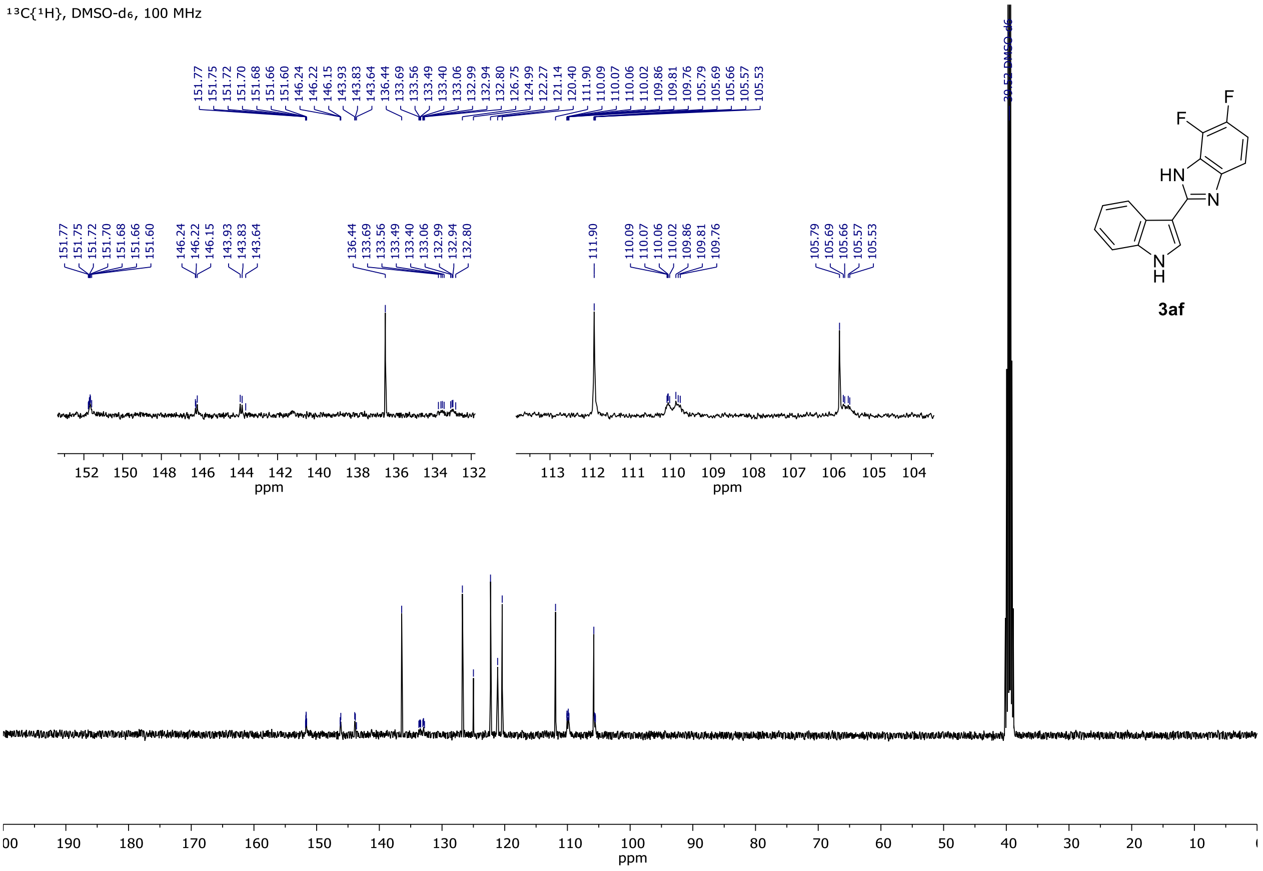

3af

¹H, DMSO-d₆, 400 MHz

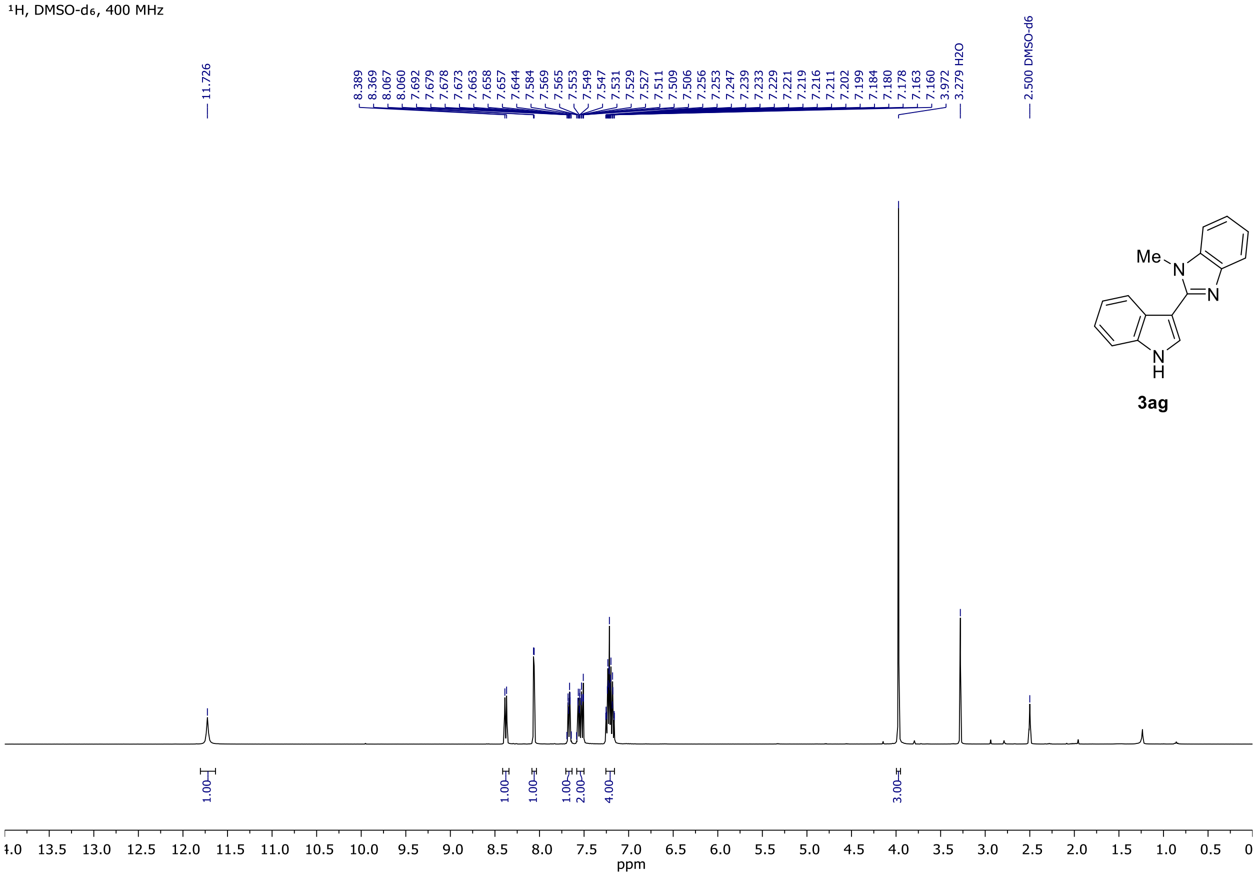

3ag

¹³C{¹H}, DMSO-d₆, 100 MHz

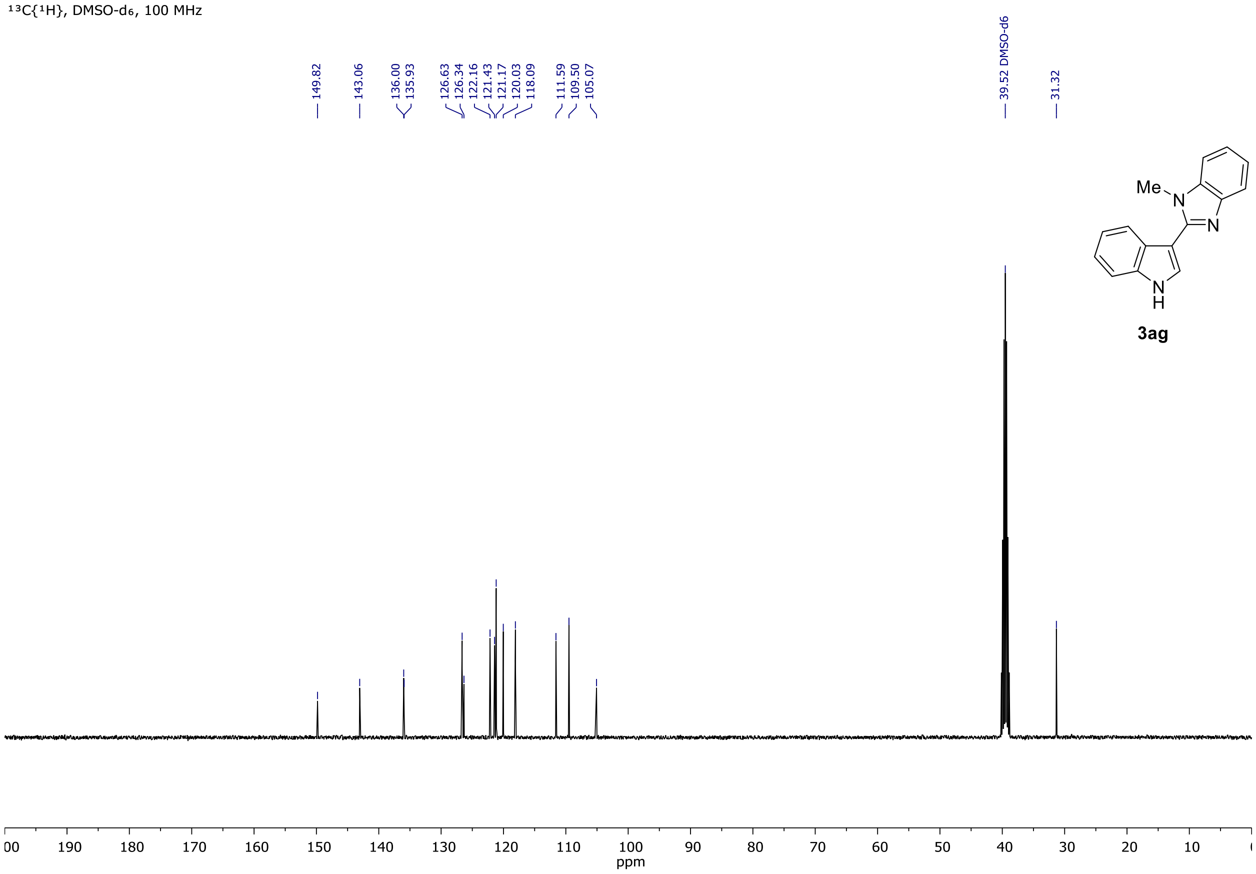

3ag

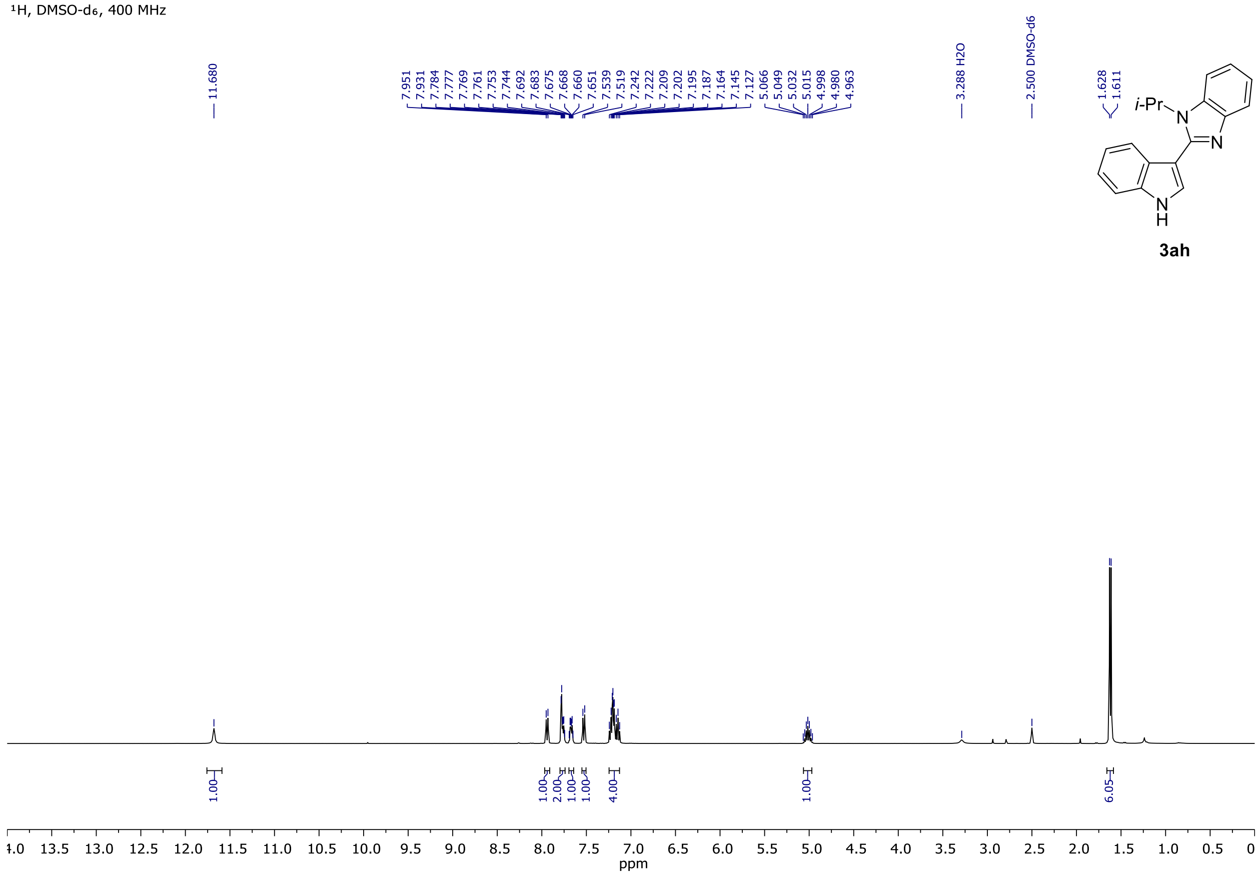
¹H, DMSO-d₆, 400 MHz
11.680
7.951
7.931
7.784
7.777
7.769
7.761
7.753
7.744
7.692
7.683
7.675
7.668
7.660
7.651
7.539
7.519
7.242
7.222
7.209
7.202
7.195
7.187
7.164
7.145
7.127
5.066
5.049
5.032
5.015
4.998
4.980
4.963
3.288 H2O
2.500 DMSO-d6
1.628
1.611
i-Pr
N
N
N
H
3ah
1.00
1.00
2.00
1.00
1.00
4.00
1.00
6.05
ppm

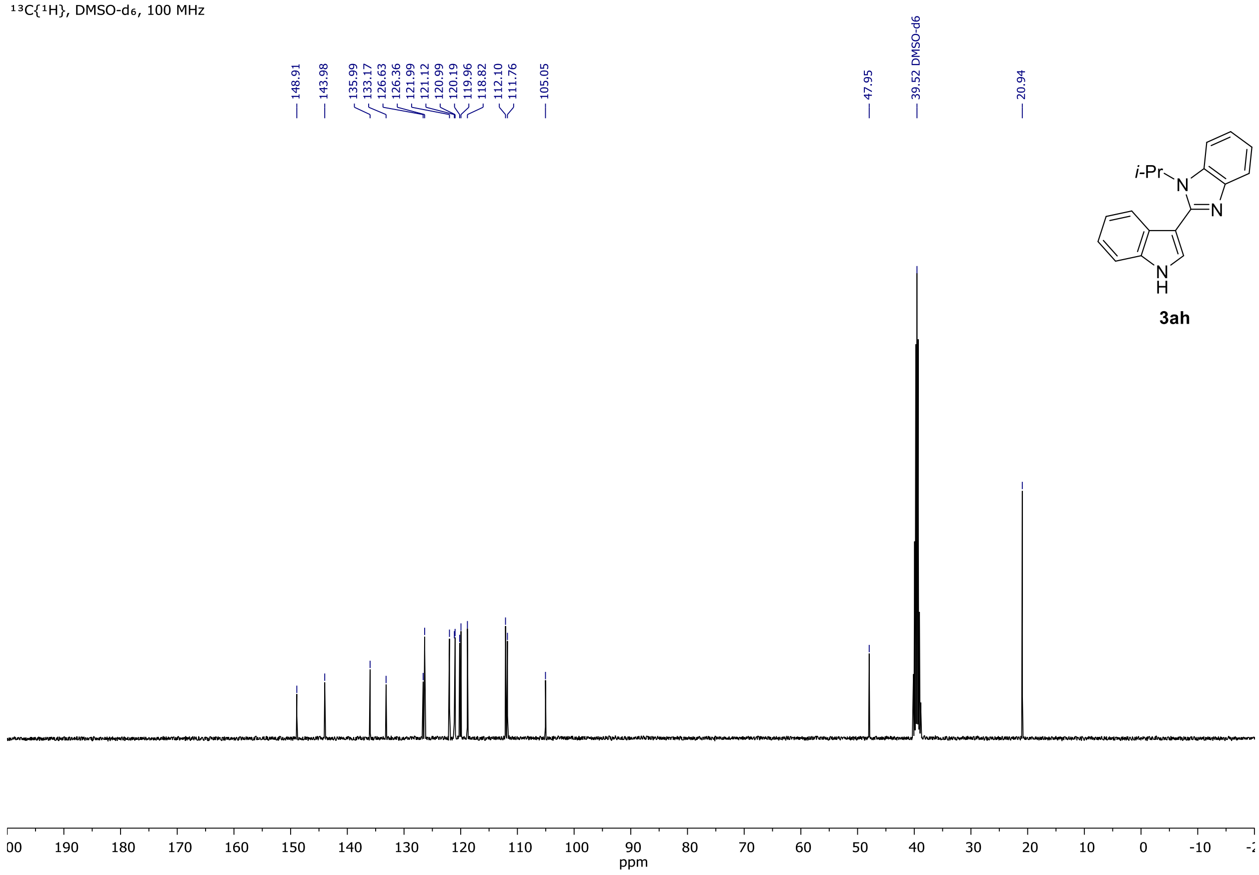
¹³C{¹H}, DMSO-d₆, 100 MHz
148.91
143.98
135.99
133.17
126.63
126.36
121.99
121.12
120.99
120.19
119.96
118.82
112.10
111.76
105.05
47.95
39.52 DMSO-d6
20.94
i-Pr
N
N
N
H
3ah
ppm

¹H, DMSO-d₆, 400 MHz

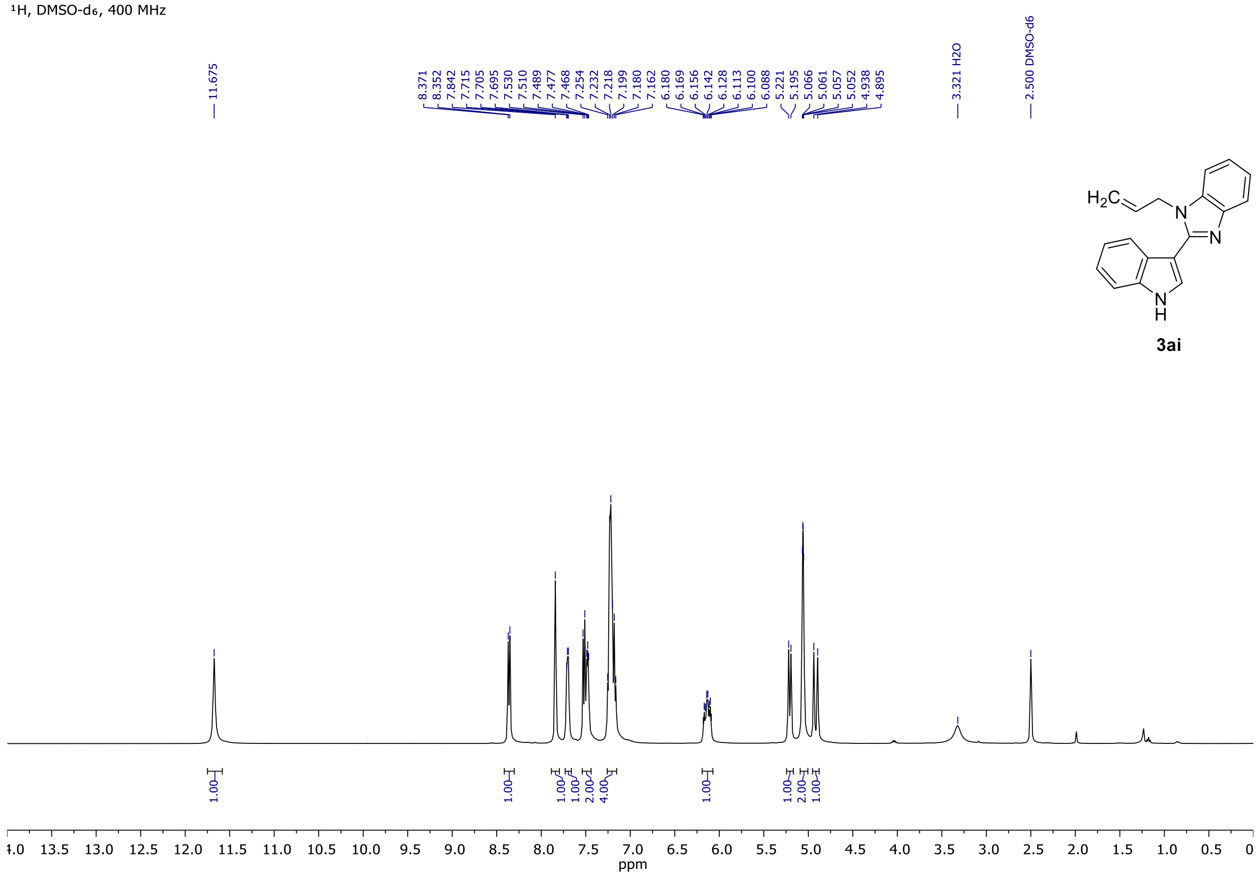

¹³C{¹H}, DMSO-d₆, 100 MHz

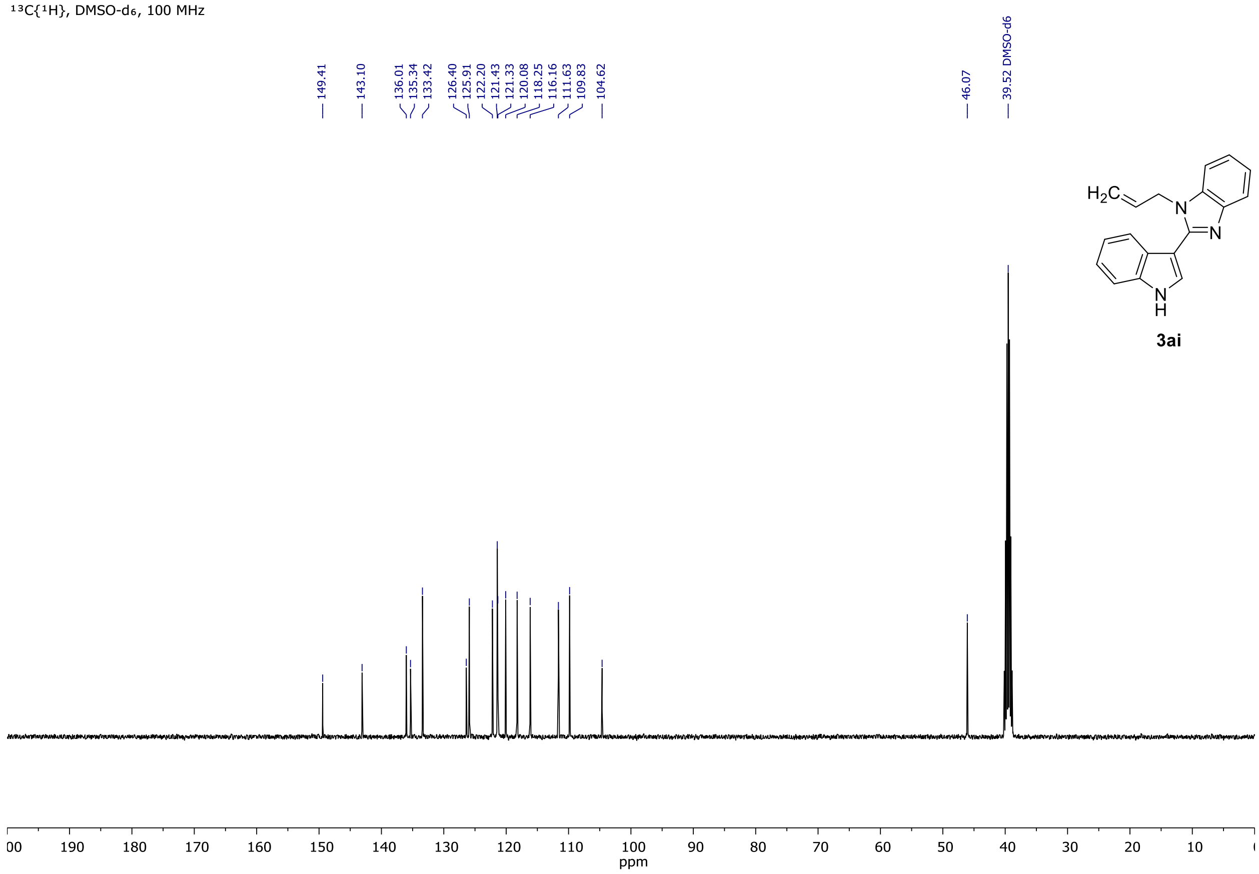

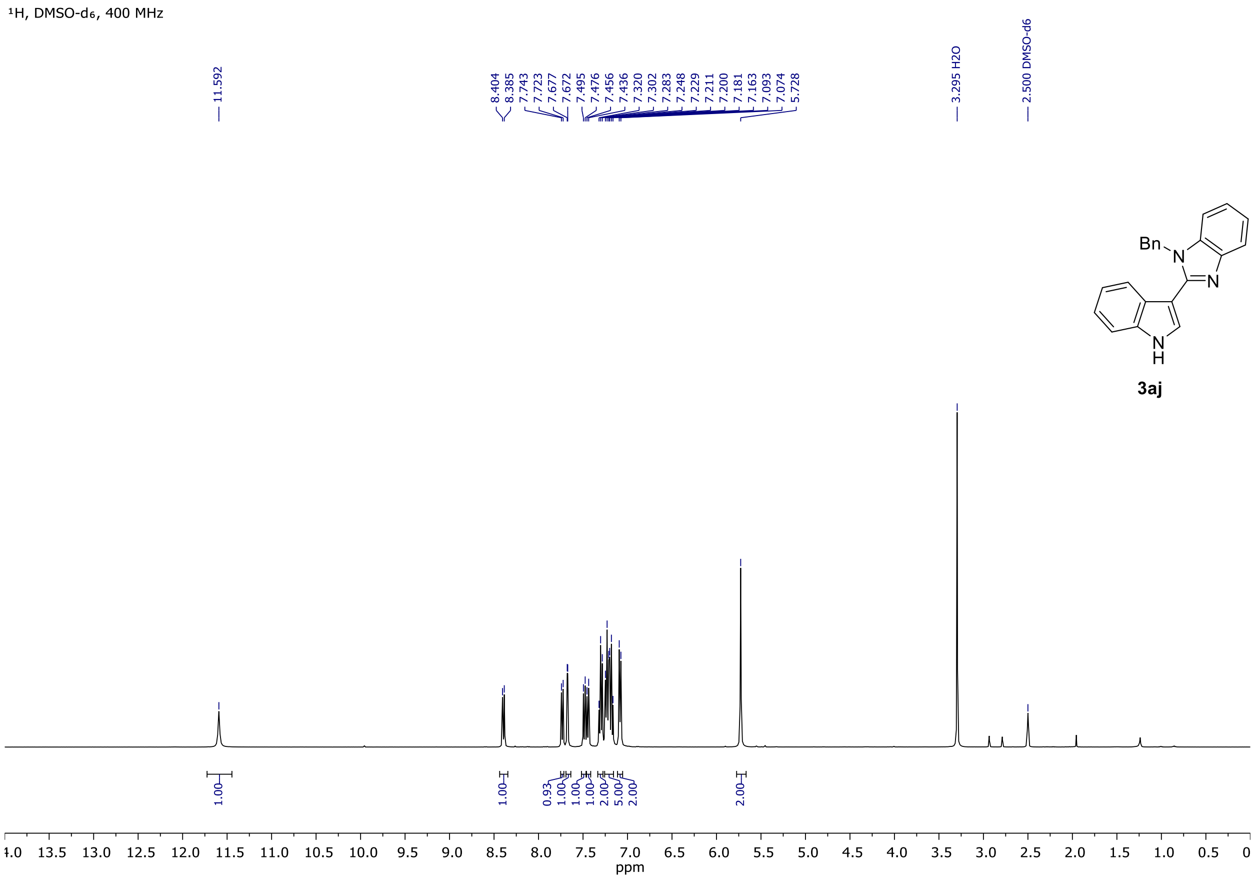
¹H, DMSO-d₆, 400 MHz
11.592
8.404
8.385
7.743
7.723
7.677
7.672
7.495
7.476
7.456
7.436
7.320
7.302
7.283
7.248
7.229
7.211
7.200
7.181
7.163
7.093
7.074
5.728
3.295 H2O
2.500 DMSO-d6
Bn
N
N
N
H
3aj
1.00
1.00
0.93
1.00
1.00
1.00
2.00
5.00
2.00
2.00
ppm

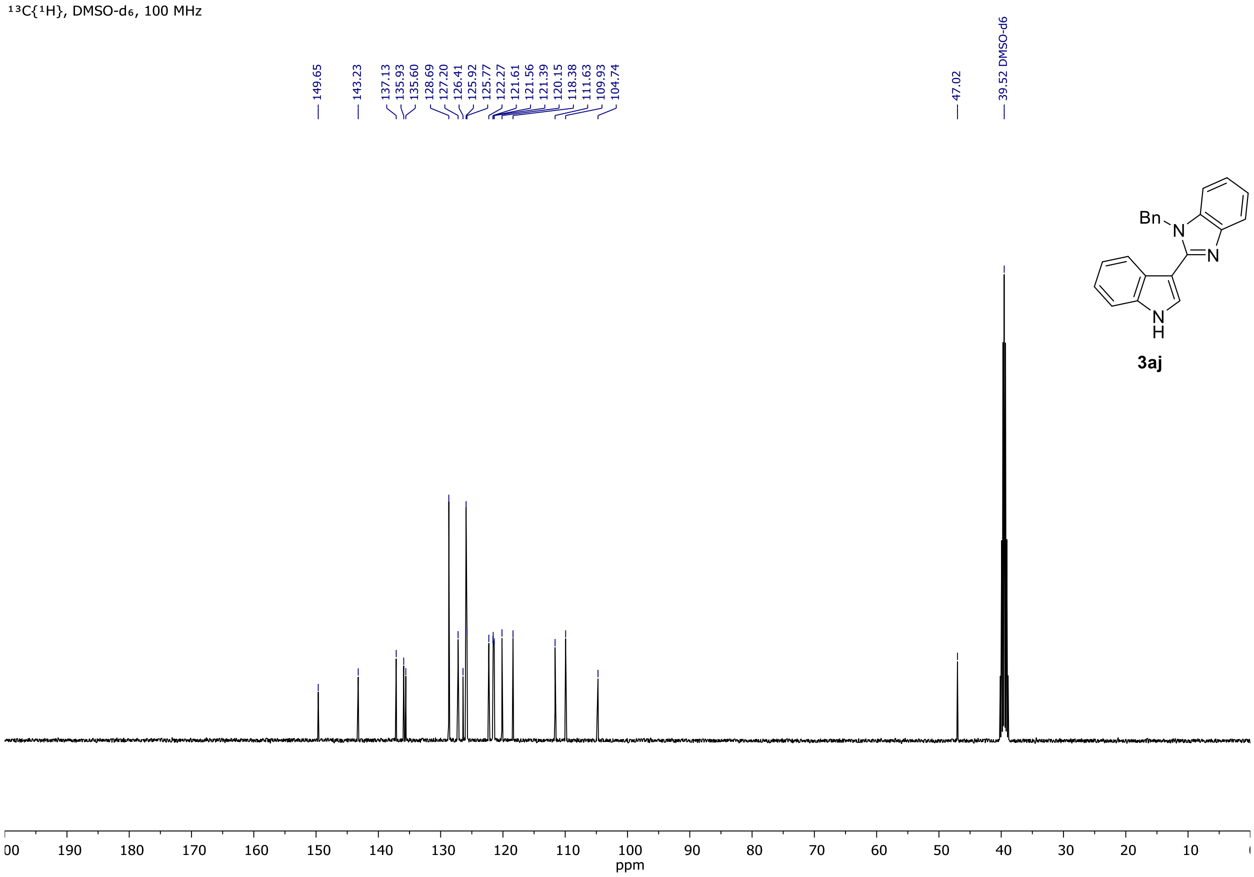
¹³C{¹H}, DMSO-d₆, 100 MHz
149.65
143.23
137.13
135.93
135.60
128.69
127.20
126.41
125.92
125.77
122.27
121.61
121.56
121.39
120.15
118.38
111.63
109.93
104.74
47.02
39.52 DMSO-d6
Bn
N
N
N
H
3aj
ppm

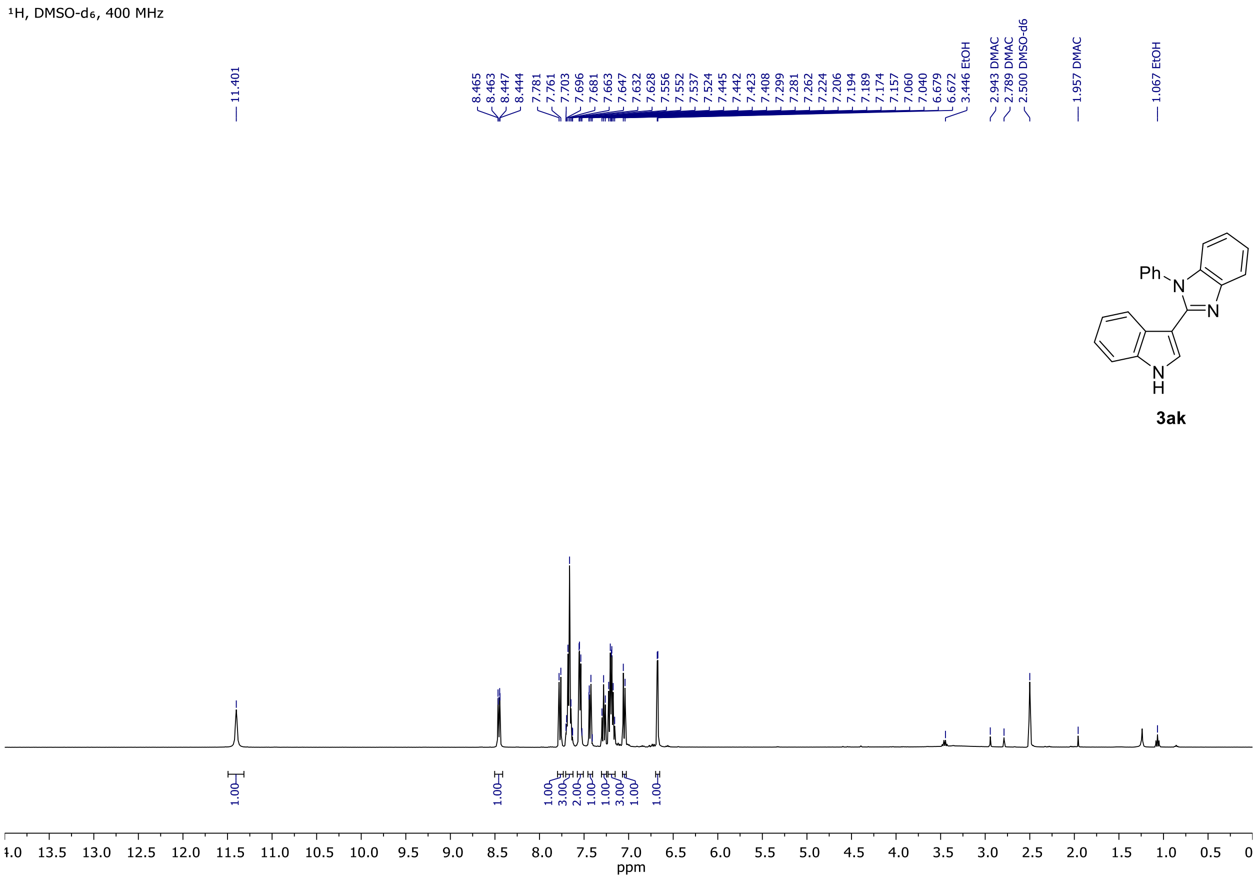
¹H, DMSO-d₆, 400 MHz
11.401
8.465
8.463
8.447
8.444
7.781
7.761
7.703
7.696
7.681
7.663
7.647
7.632
7.628
7.556
7.552
7.537
7.524
7.445
7.442
7.423
7.408
7.299
7.281
7.262
7.224
7.206
7.194
7.189
7.174
7.157
7.060
7.040
6.679
6.672
3.446 EtOH
2.943 DMAC
2.789 DMAC
2.500 DMSO-d6
1.957 DMAC
1.067 EtOH
Ph
N
N
N
H
3ak
1.00
1.00
1.00
3.00
2.00
1.00
1.00
3.00
1.00
1.00
ppm

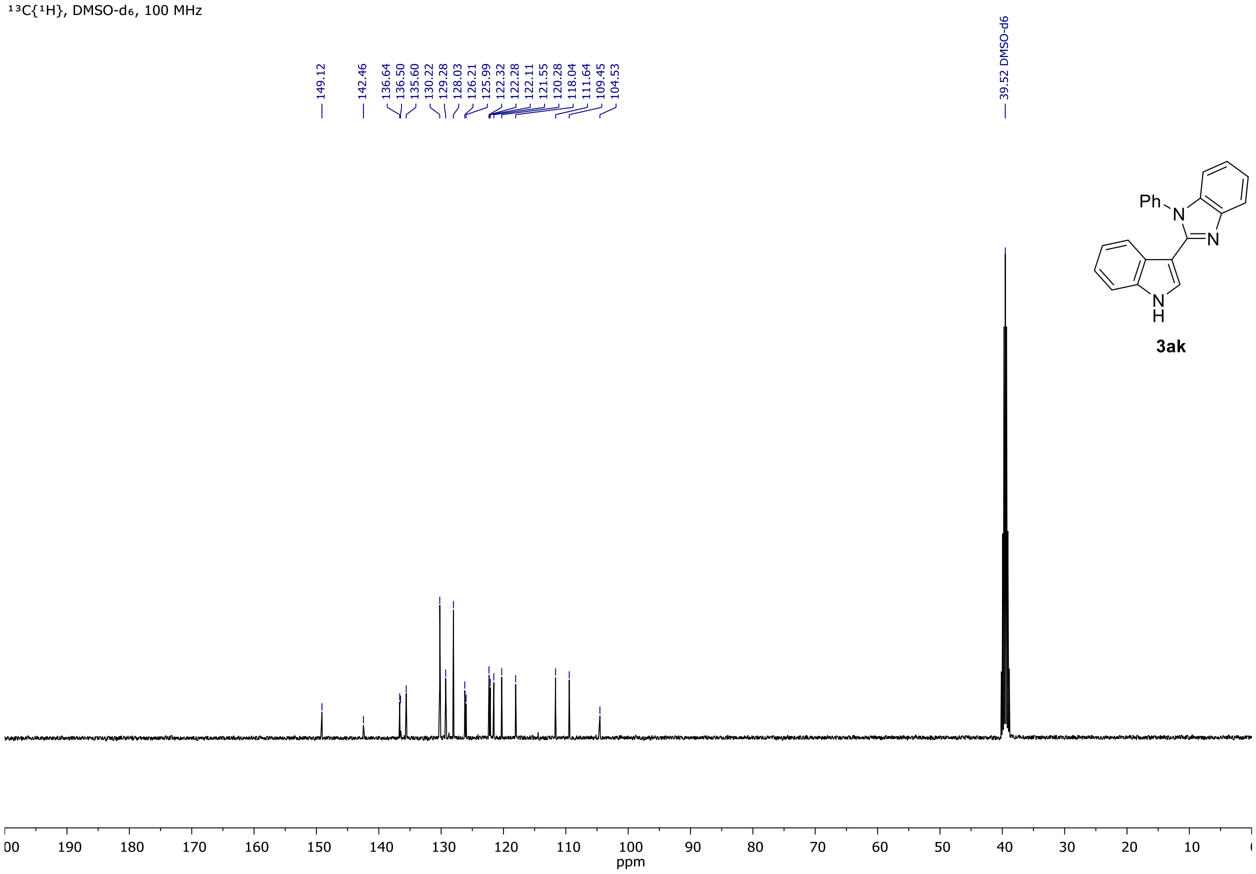
¹³C{¹H}, DMSO-d₆, 100 MHz
149.12
142.46
136.64
136.50
135.60
130.22
129.28
128.03
126.21
125.99
122.32
122.28
122.11
121.55
120.28
118.04
111.64
109.45
104.53
39.52 DMSO-d6
Ph
N
N
N
H
3ak
ppm

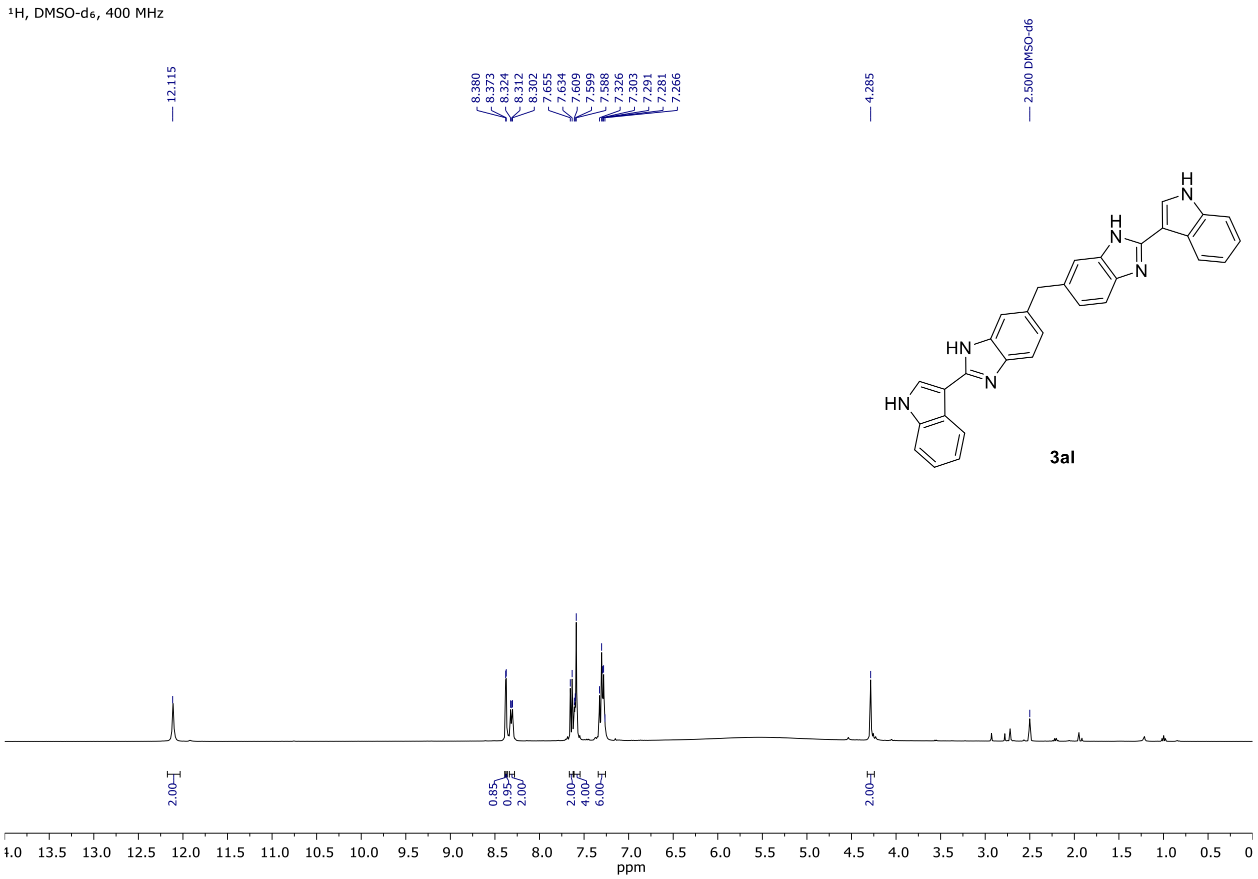
¹H, DMSO-d₆, 400 MHz
12.115
8.380
8.373
8.324
8.312
8.302
7.655
7.634
7.609
7.599
7.588
7.326
7.303
7.291
7.281
7.266
4.285
2.500 DMSO-d6
H
N
H
N
N
HN
N
HN
3al
2.00
0.85
0.95
2.00
2.00
4.00
6.00
2.00
ppm

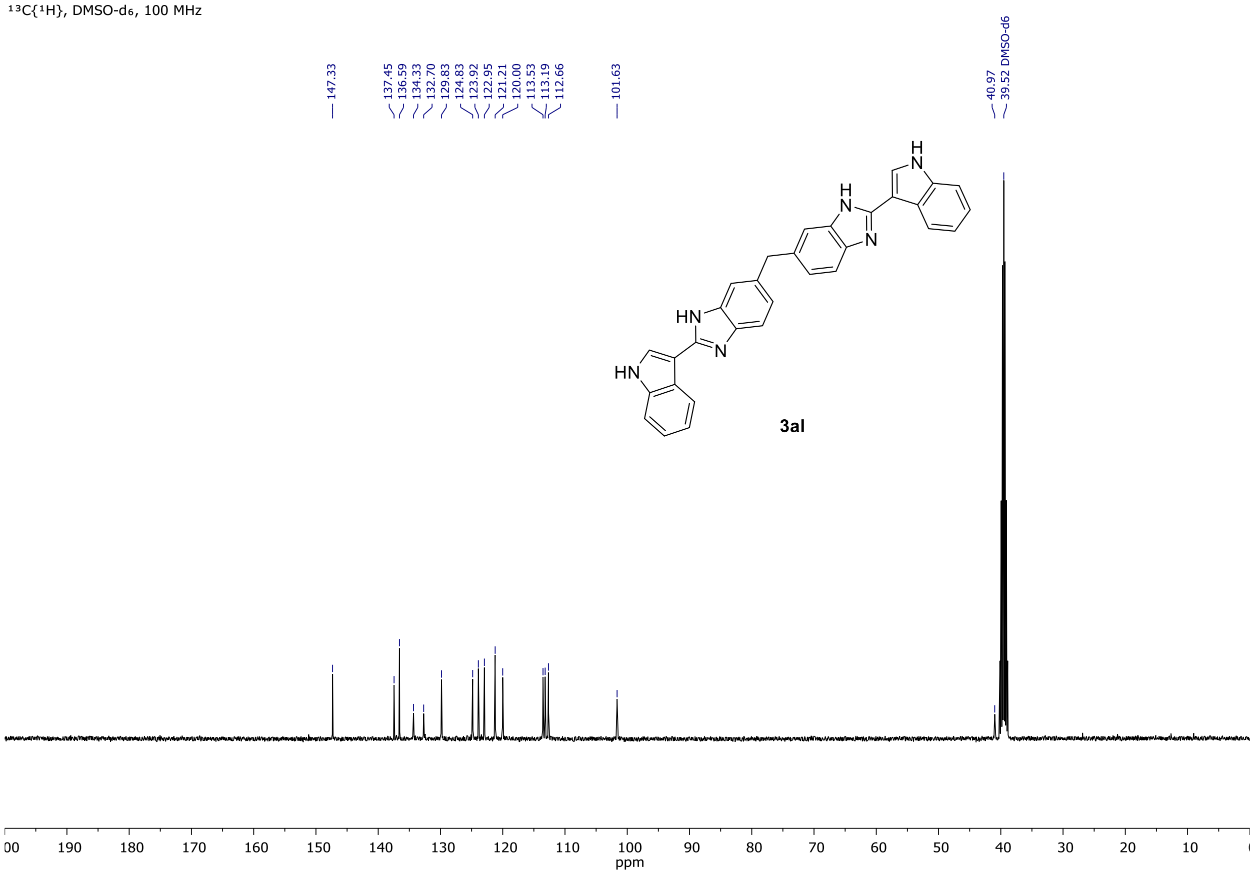
¹³C{¹H}, DMSO-d₆, 100 MHz
147.33
137.45
136.59
134.33
132.70
129.83
124.83
123.92
122.95
121.21
120.00
113.53
113.19
112.66
101.63
40.97
39.52 DMSO-d6
H
N
H
N
N
HN
N
HN
3al
ppm

^{1}H, DMSO-d_6, 400 MHz

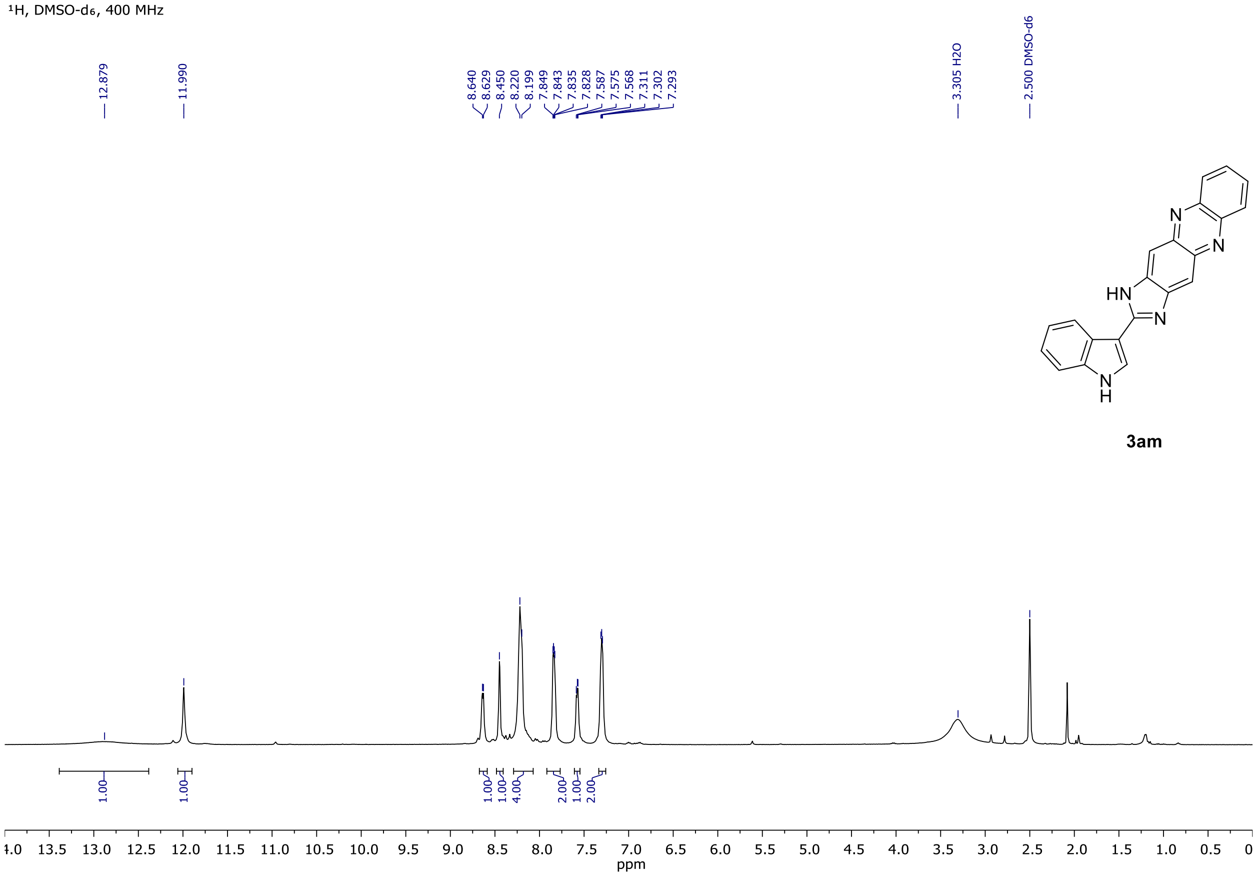

$^{13}C\{^{1}H\}$, DMSO-d_6, 100 MHz

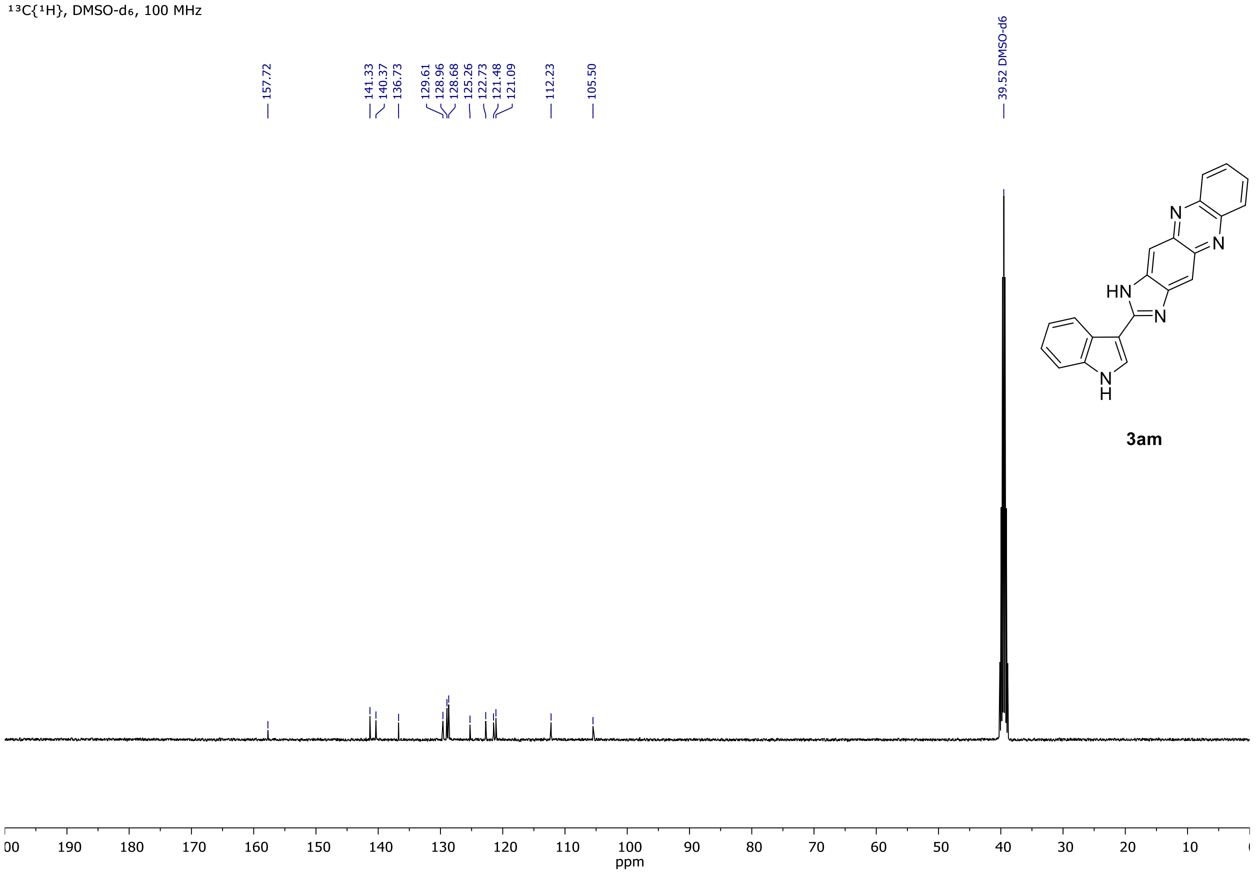

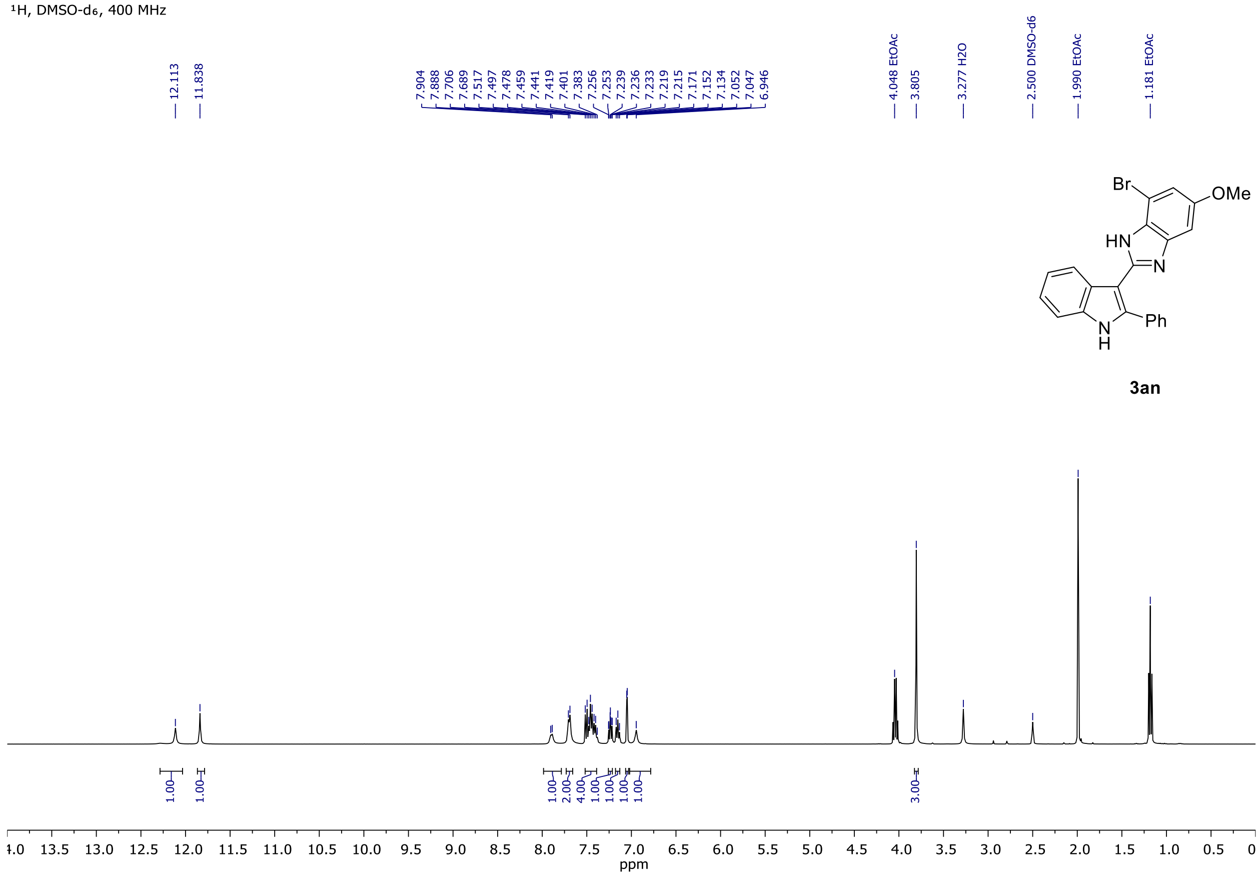
¹H, DMSO-d₆, 400 MHz
12.113
11.838
7.904 7.888 7.706 7.689 7.517 7.497 7.478 7.459 7.441 7.419 7.401 7.383 7.256 7.253 7.239 7.236 7.233 7.219 7.215 7.171 7.152 7.134 7.052 7.047 6.946
4.048 EtOAc
3.805
3.277 H2O
2.500 DMSO-d6
1.990 EtOAc
1.181 EtOAc
Br
OMe
HN
N
N
H
Ph
3an
1.00 1.00 1.00 2.00 4.00 1.00 1.00 1.00 1.00 3.00
ppm

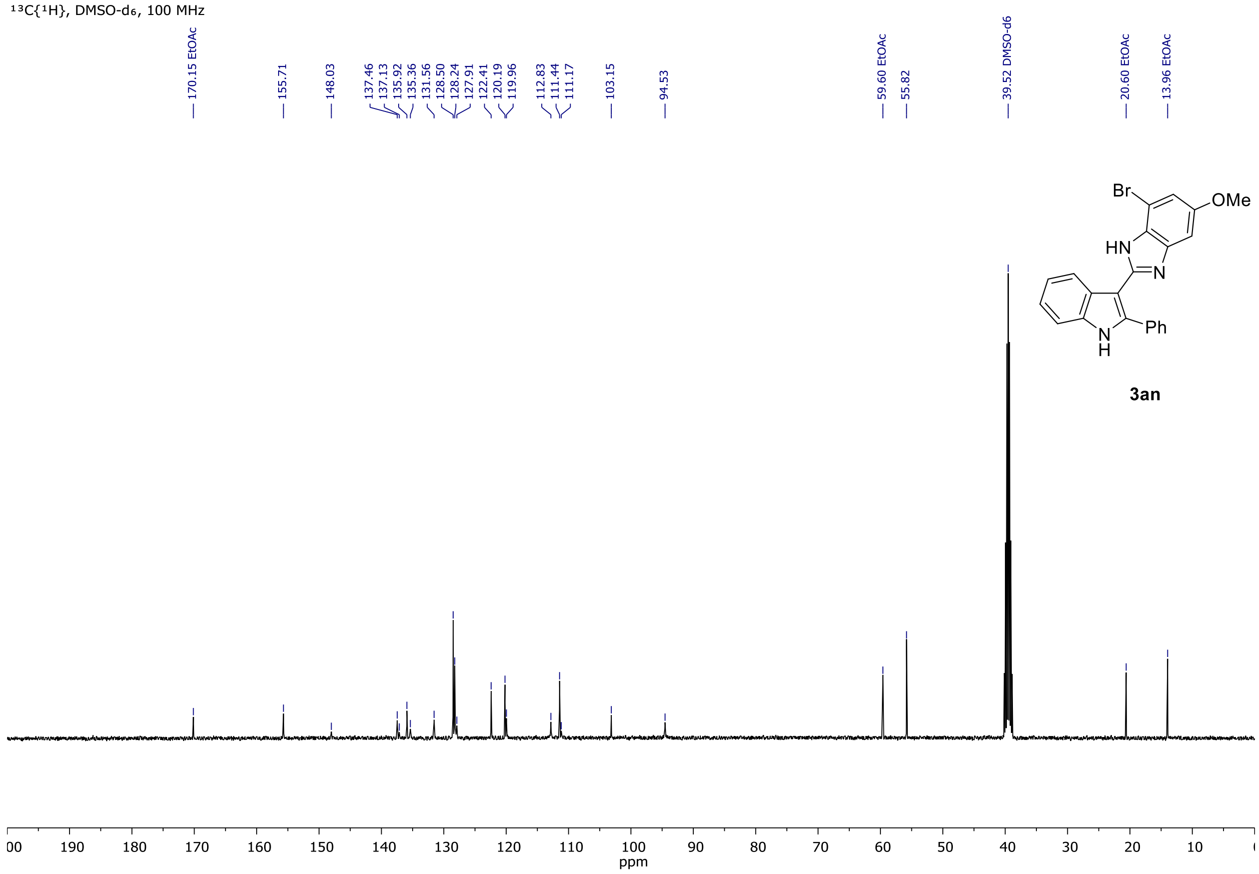
¹³C{¹H}, DMSO-d₆, 100 MHz
170.15 EtOAc
155.71
148.03
137.46 137.13 135.92 135.36 131.56 128.50 128.24 127.91 122.41 120.19 119.96
112.83 111.44 111.17
103.15
94.53
59.60 EtOAc
55.82
39.52 DMSO-d6
20.60 EtOAc
13.96 EtOAc
Br
OMe
HN
N
N
H
Ph
3an
ppm

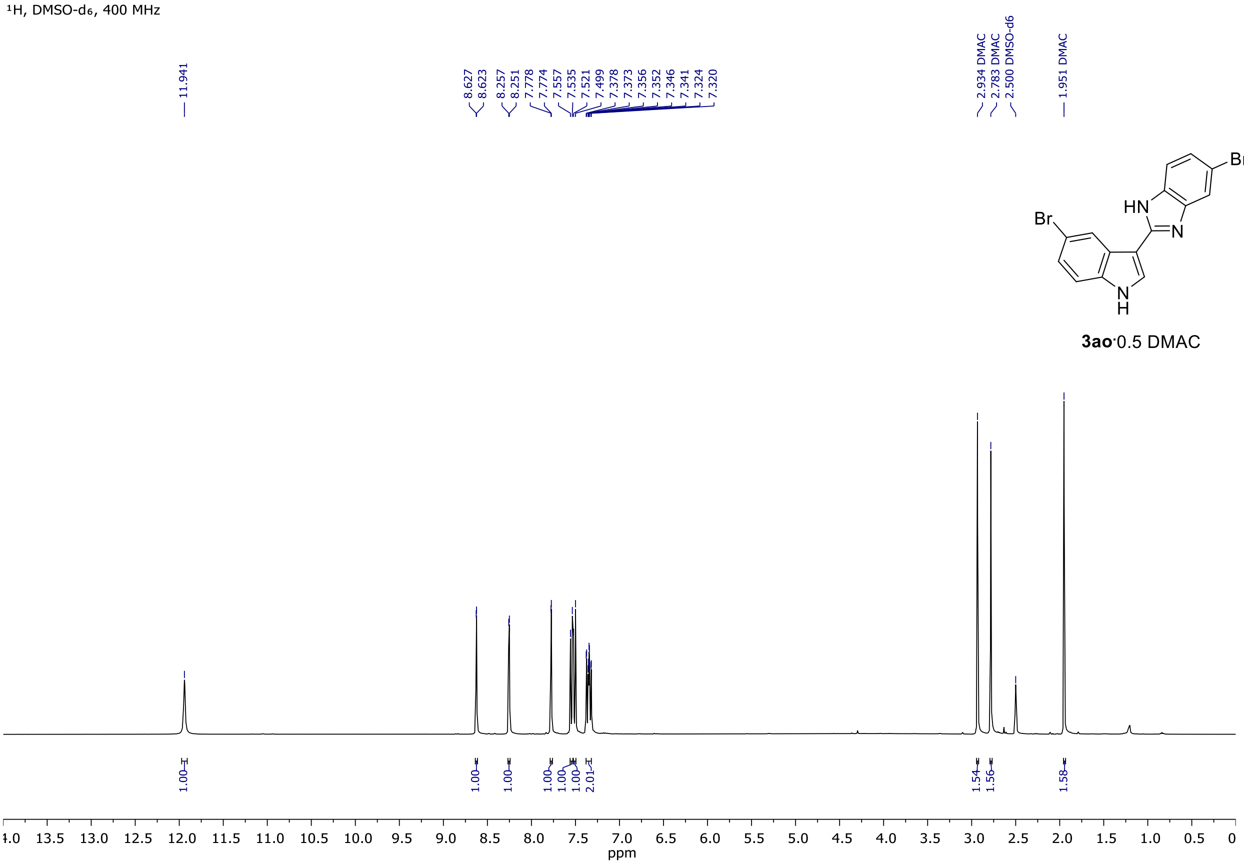
¹H, DMSO-d₆, 400 MHz
11.941
8.627
8.623
8.257
8.251
7.778
7.774
7.557
7.535
7.521
7.499
7.378
7.373
7.356
7.352
7.346
7.341
7.324
7.320
2.934 DMAC
2.783 DMAC
2.500 DMSO-d6
1.951 DMAC
Br
HN
N
Br
N
H
3ao·0.5 DMAC
1.00
1.00
1.00
1.00
1.00
1.00
2.01
1.54
1.56
1.58
ppm

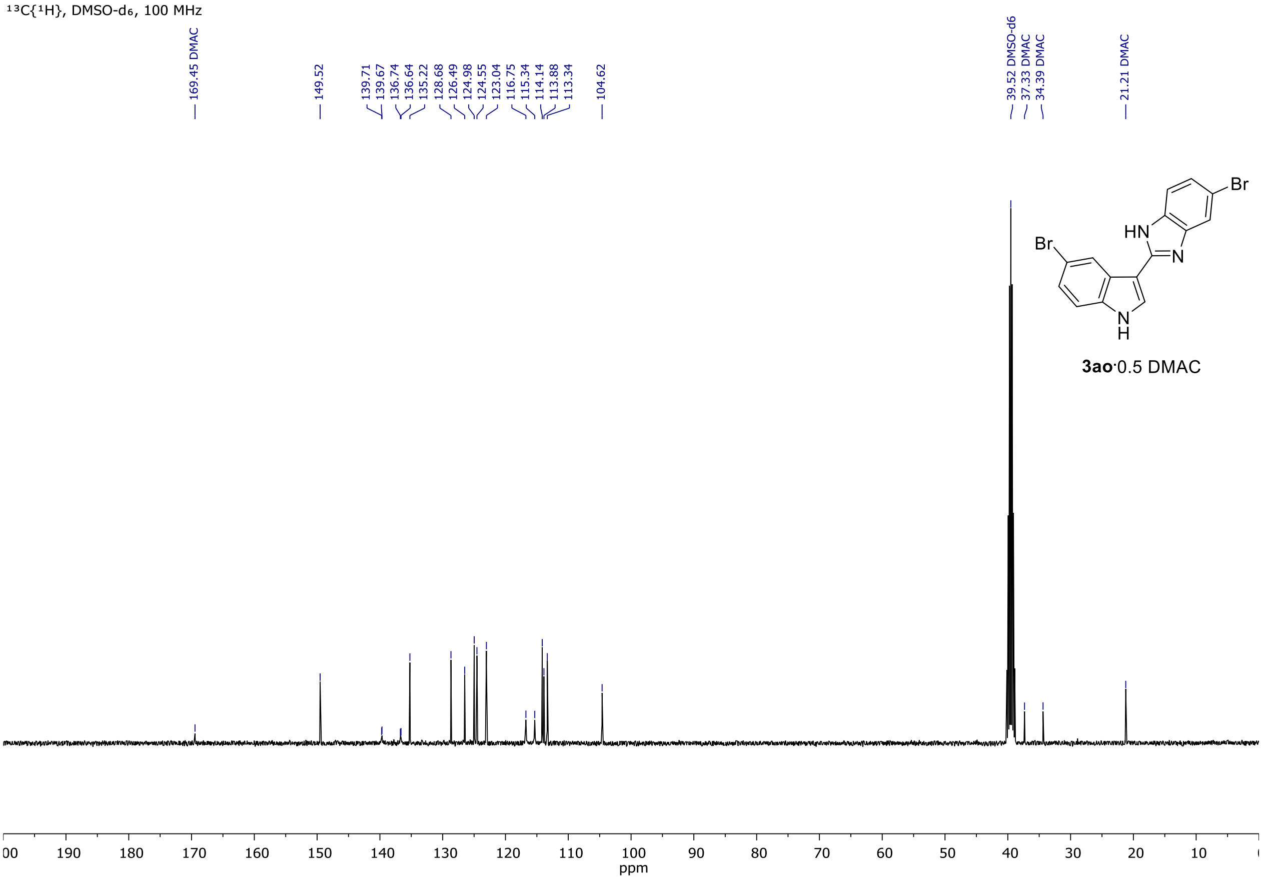
¹³C{¹H}, DMSO-d₆, 100 MHz
169.45 DMAC
149.52
139.71
139.67
136.74
136.64
135.22
128.68
126.49
124.98
124.55
123.04
116.75
115.34
114.14
113.88
113.34
104.62
39.52 DMSO-d6
37.33 DMAC
34.39 DMAC
21.21 DMAC
Br
HN
N
Br
N
H
3ao·0.5 DMAC
ppm

¹H, DMSO-d₆, 400 MHz

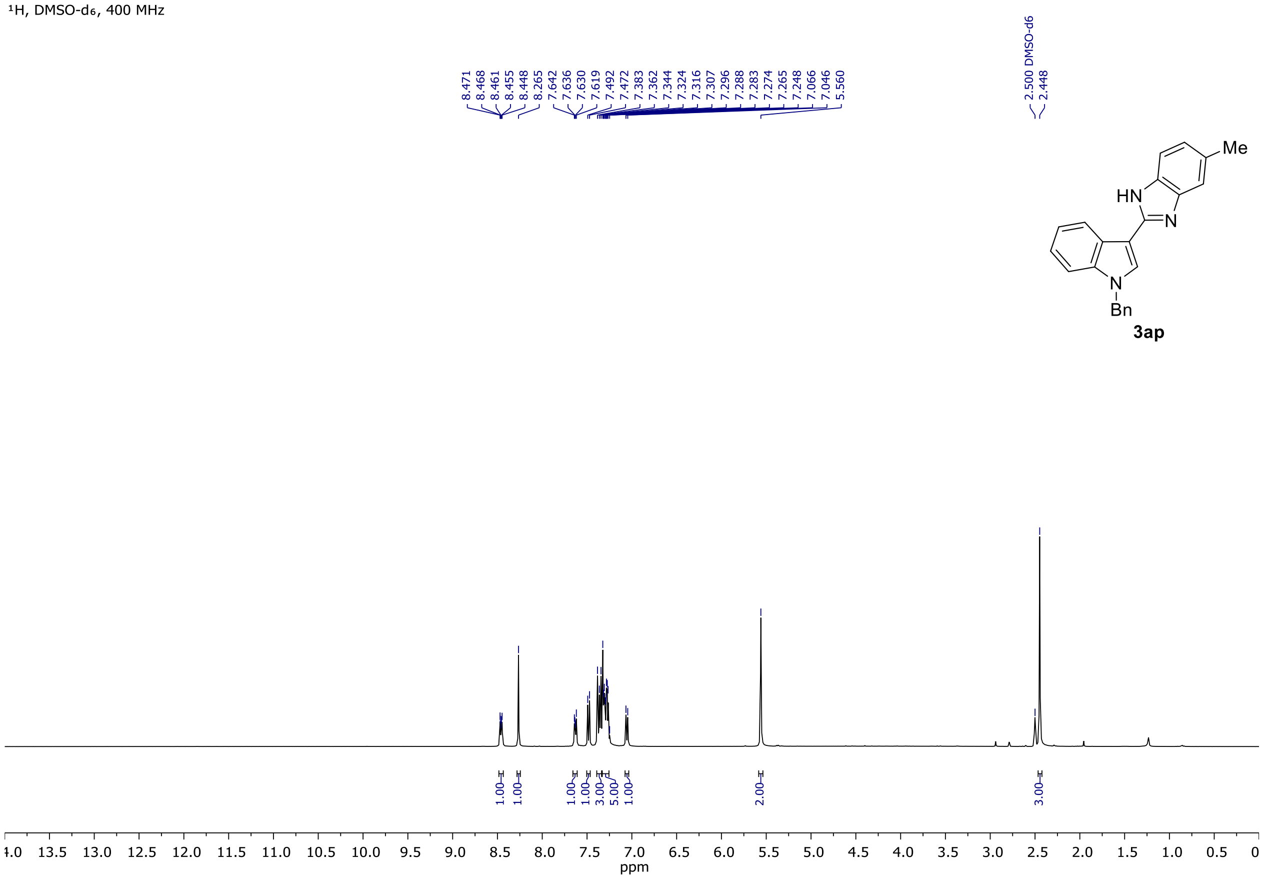

¹³C{¹H}, DMSO-d₆, 100 MHz

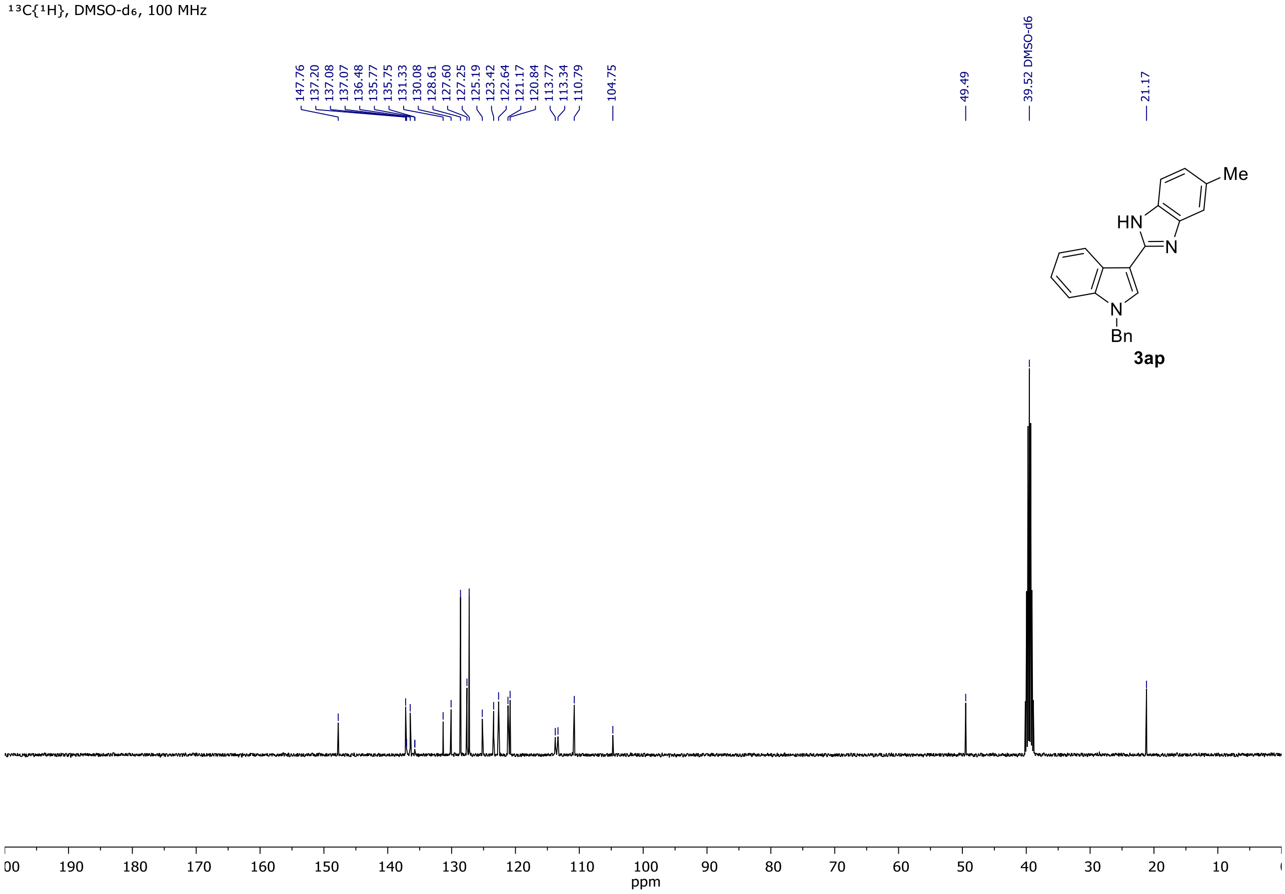

^{1}H, DMSO-d_6, 400 MHz

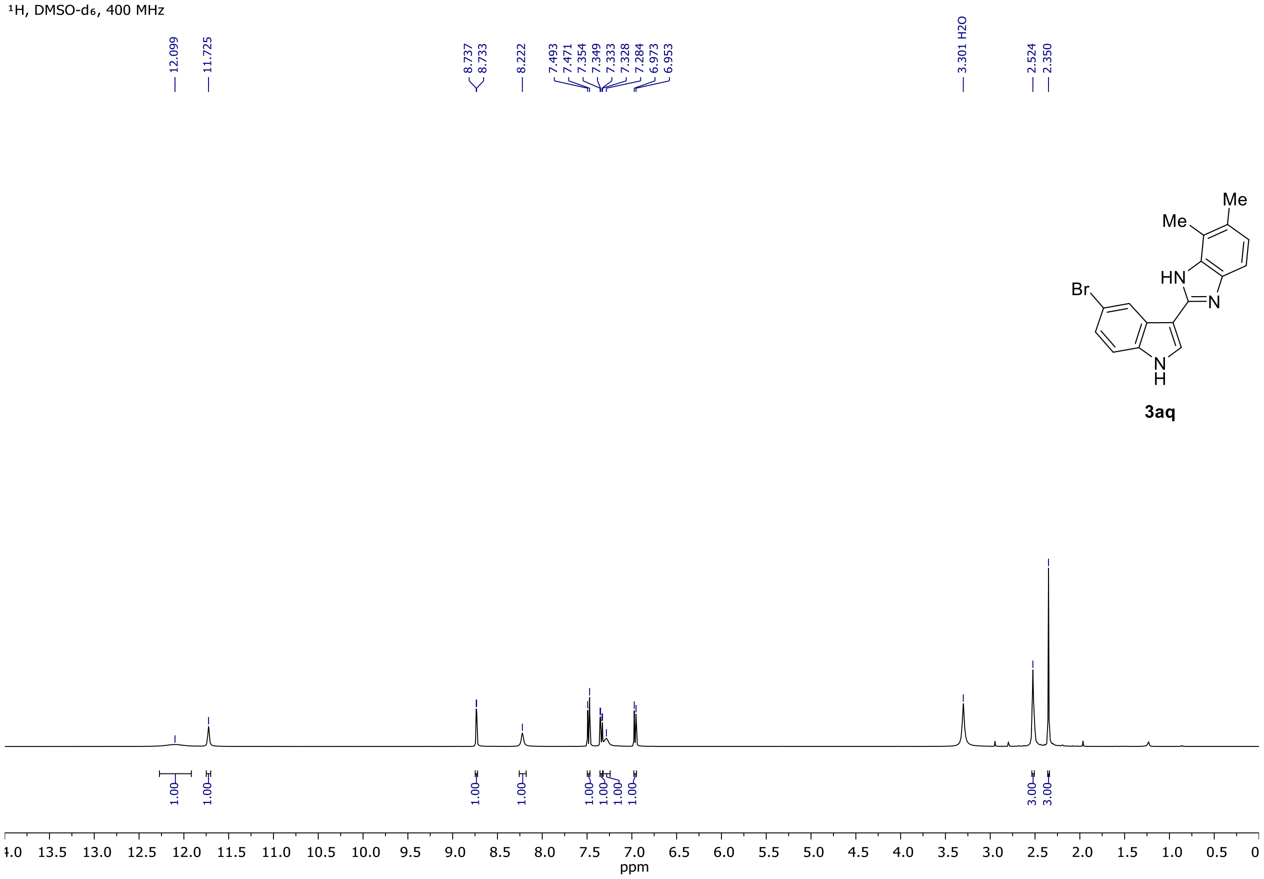

$^{13}C\{^{1}H\}$, DMSO-d_6, 100 MHz

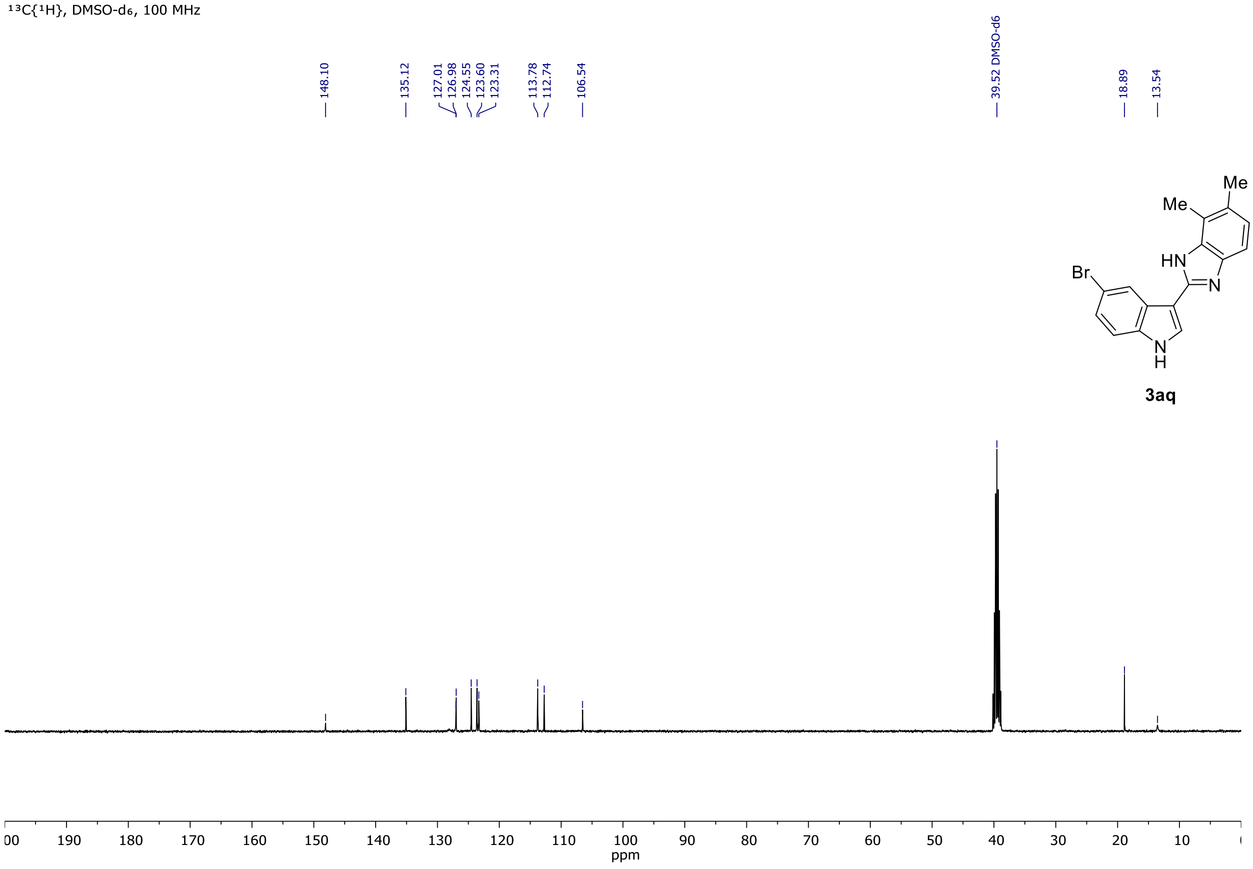

^1H, DMSO-d_6, 400 MHz

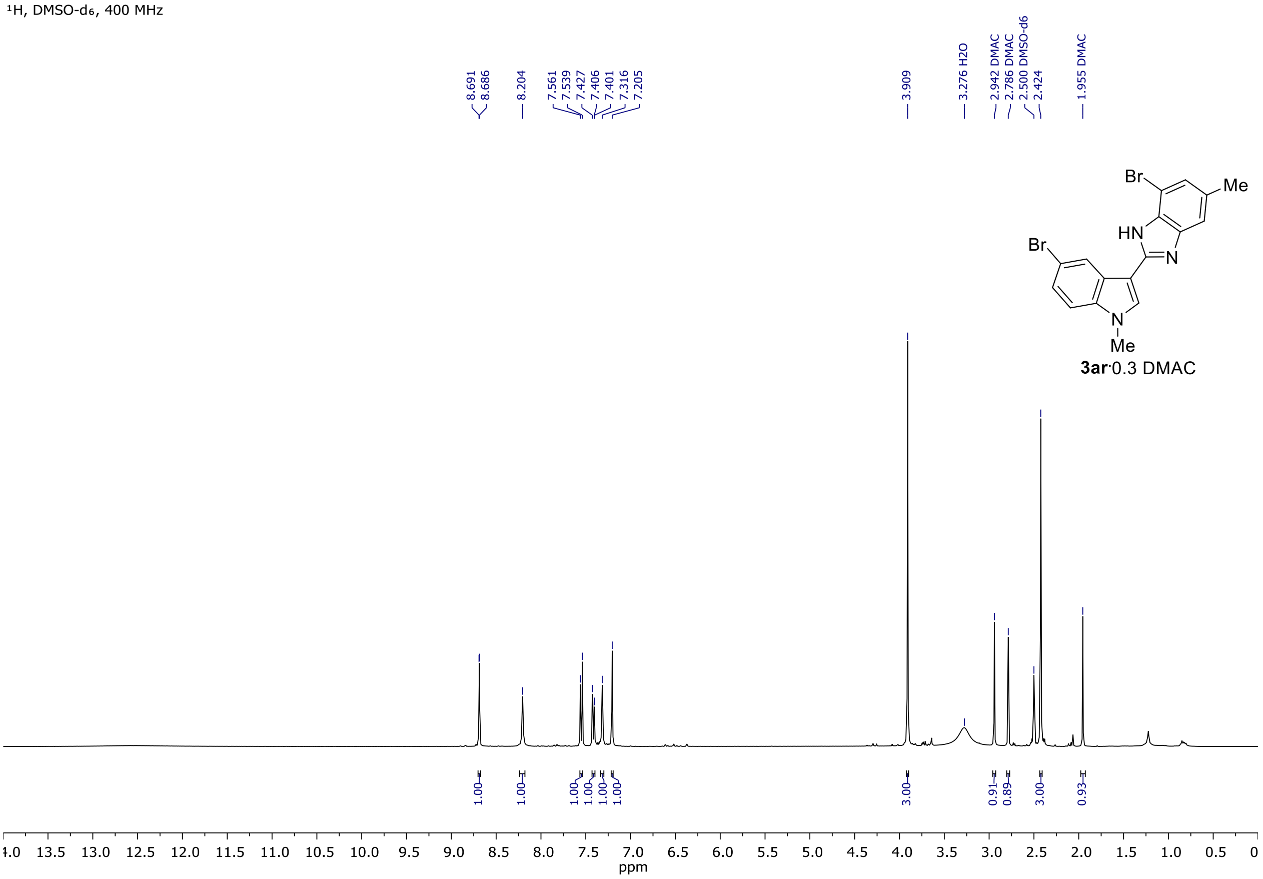

$^{13}C\{^1H\}$, DMSO-d_6, 100 MHz

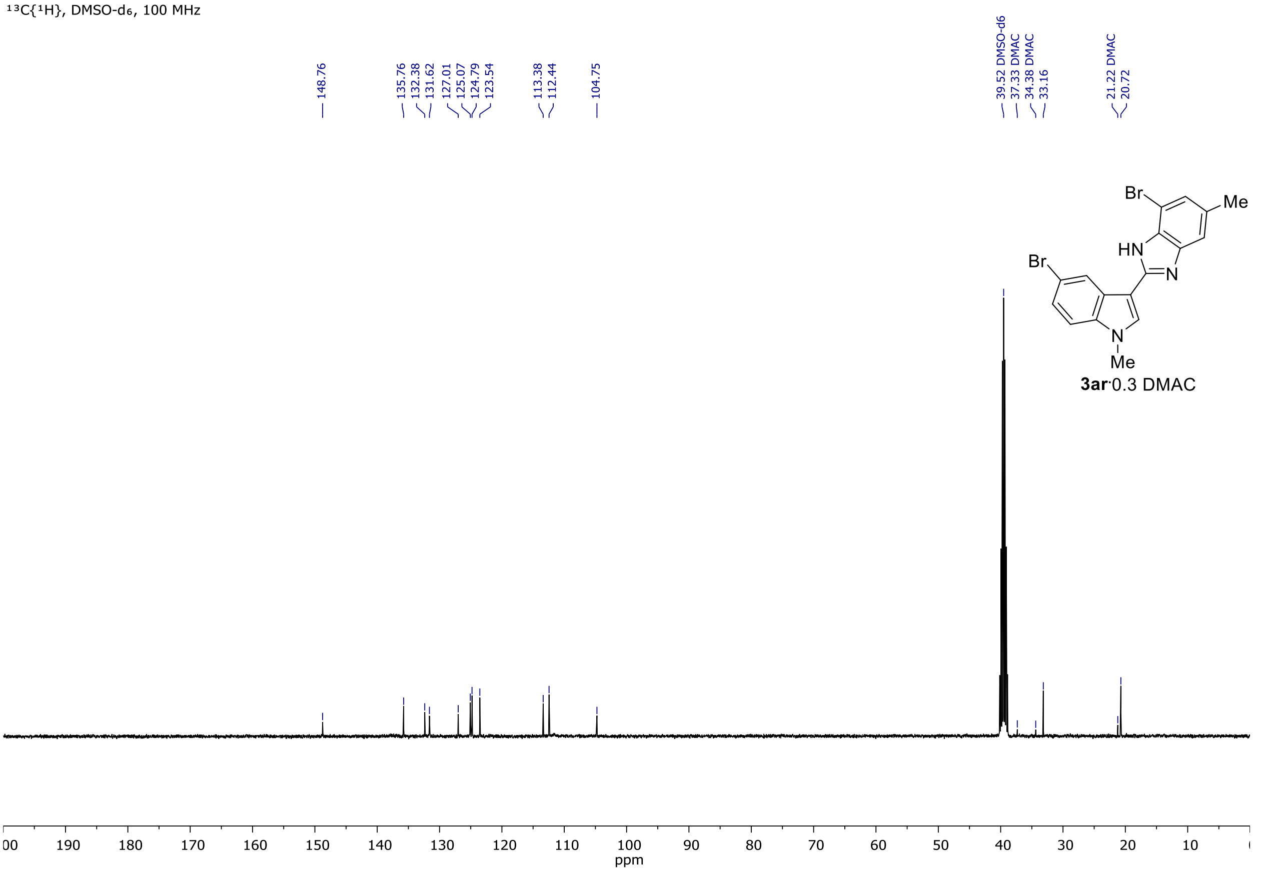

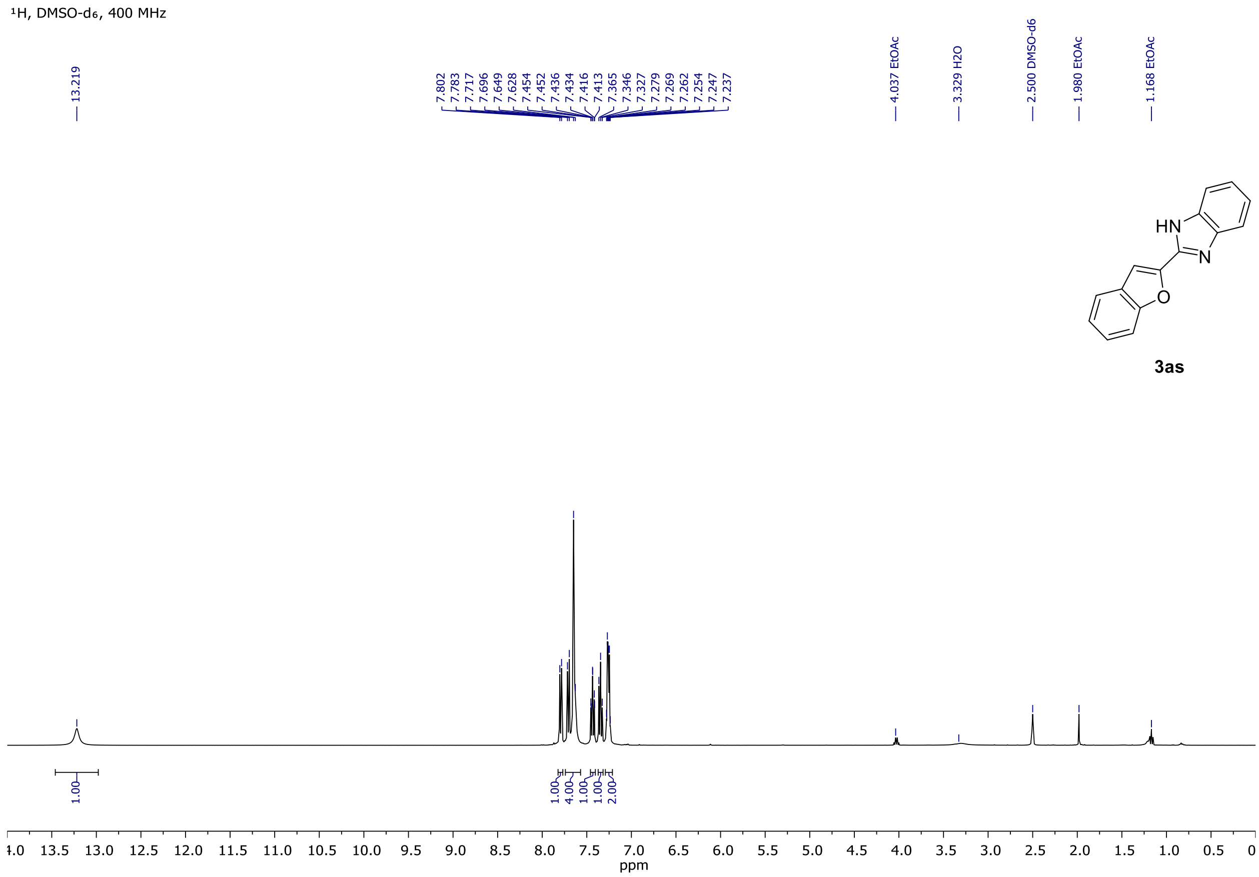
¹H, DMSO-d₆, 400 MHz
13.219
7.802 7.783 7.717 7.696 7.649 7.628 7.454 7.452 7.436 7.434 7.416 7.413 7.365 7.346 7.327 7.279 7.269 7.262 7.254 7.247 7.237
4.037 EtOAc
3.329 H2O
2.500 DMSO-d6
1.980 EtOAc
1.168 EtOAc
HN
N
O
3as
1.00
1.00
4.00
1.00
1.00
2.00
ppm

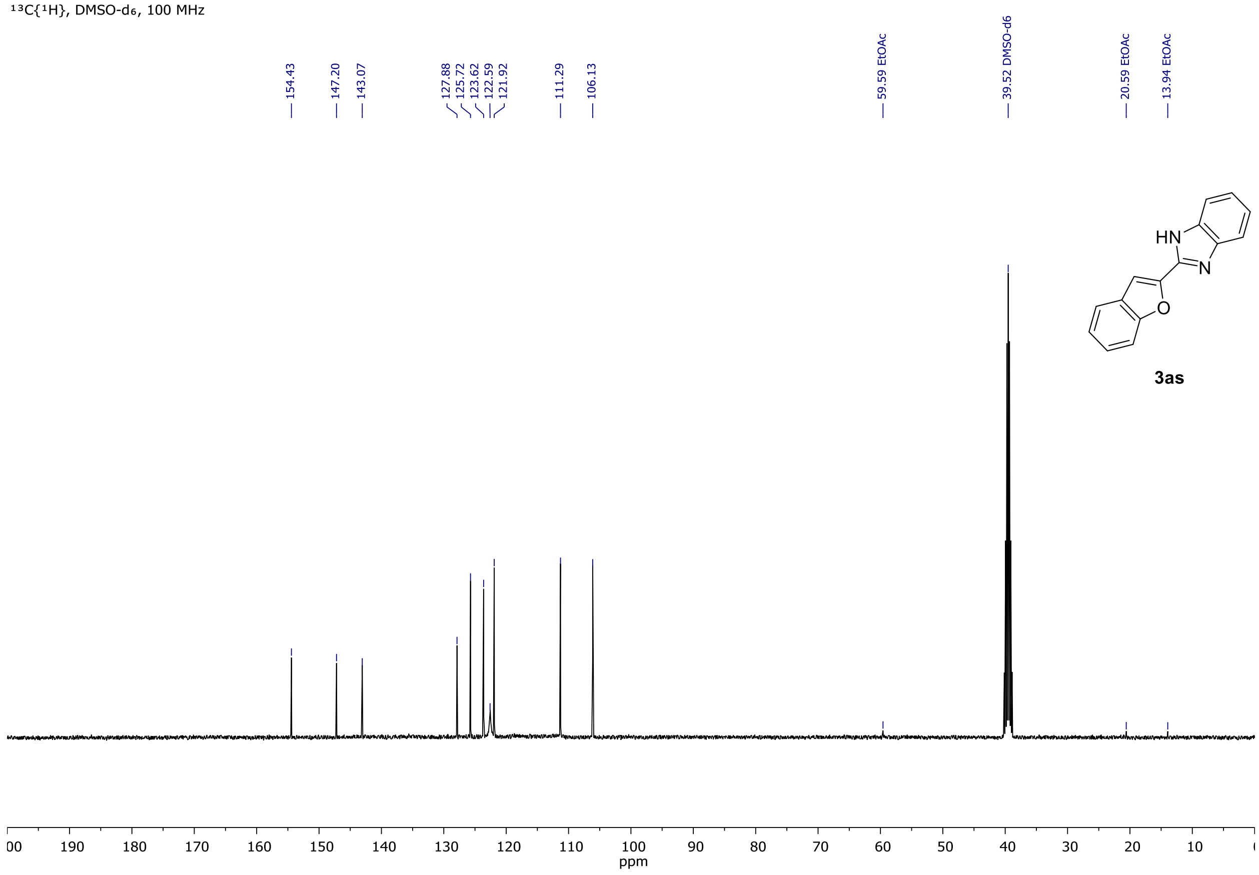
¹³C{¹H}, DMSO-d₆, 100 MHz
154.43
147.20
143.07
127.88
125.72
123.62
122.59
121.92
111.29
106.13
59.59 EtOAc
39.52 DMSO-d6
20.59 EtOAc
13.94 EtOAc
HN
N
O
3as
ppm

^1H, DMSO-d$_6$, 400 MHz

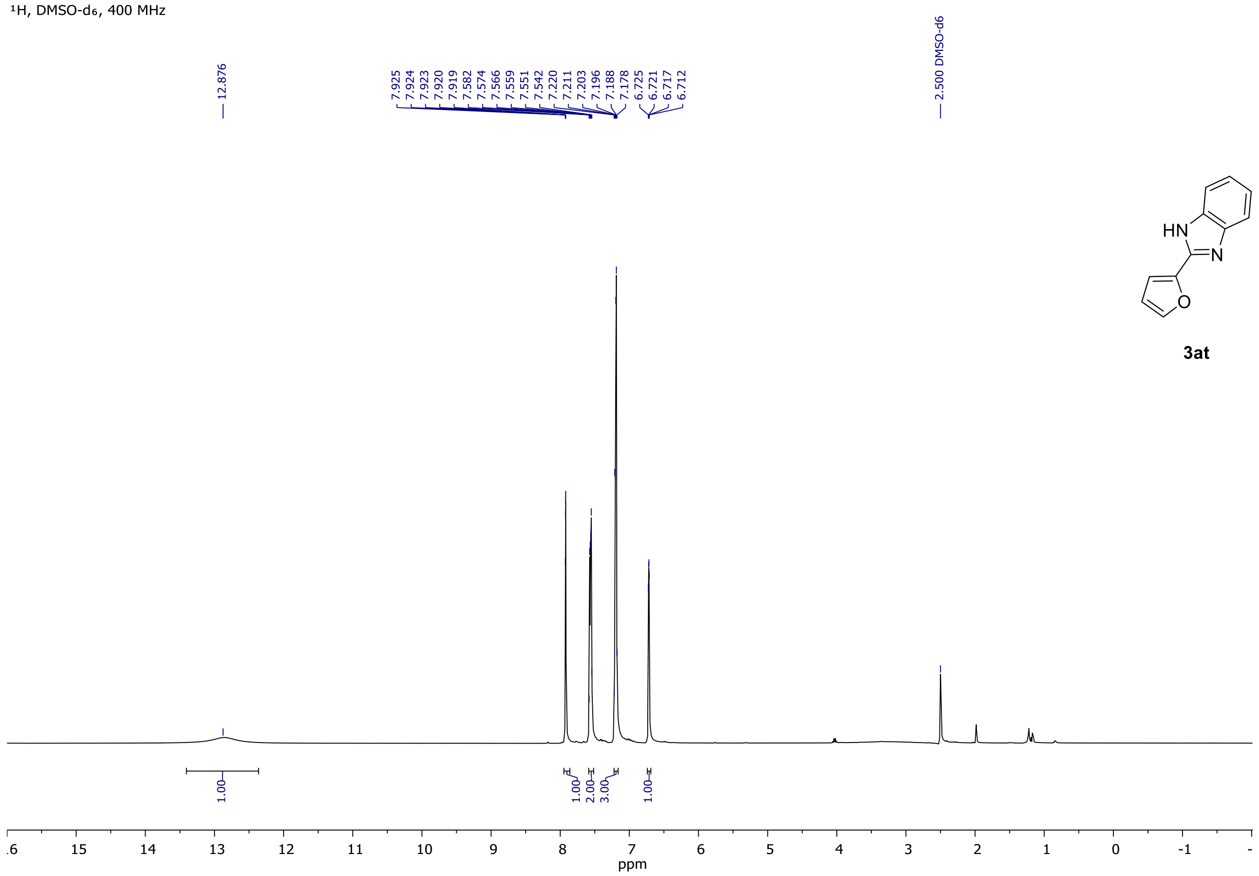

$^{13}C\{^1H\}$, DMSO-d$_6$, 100 MHz

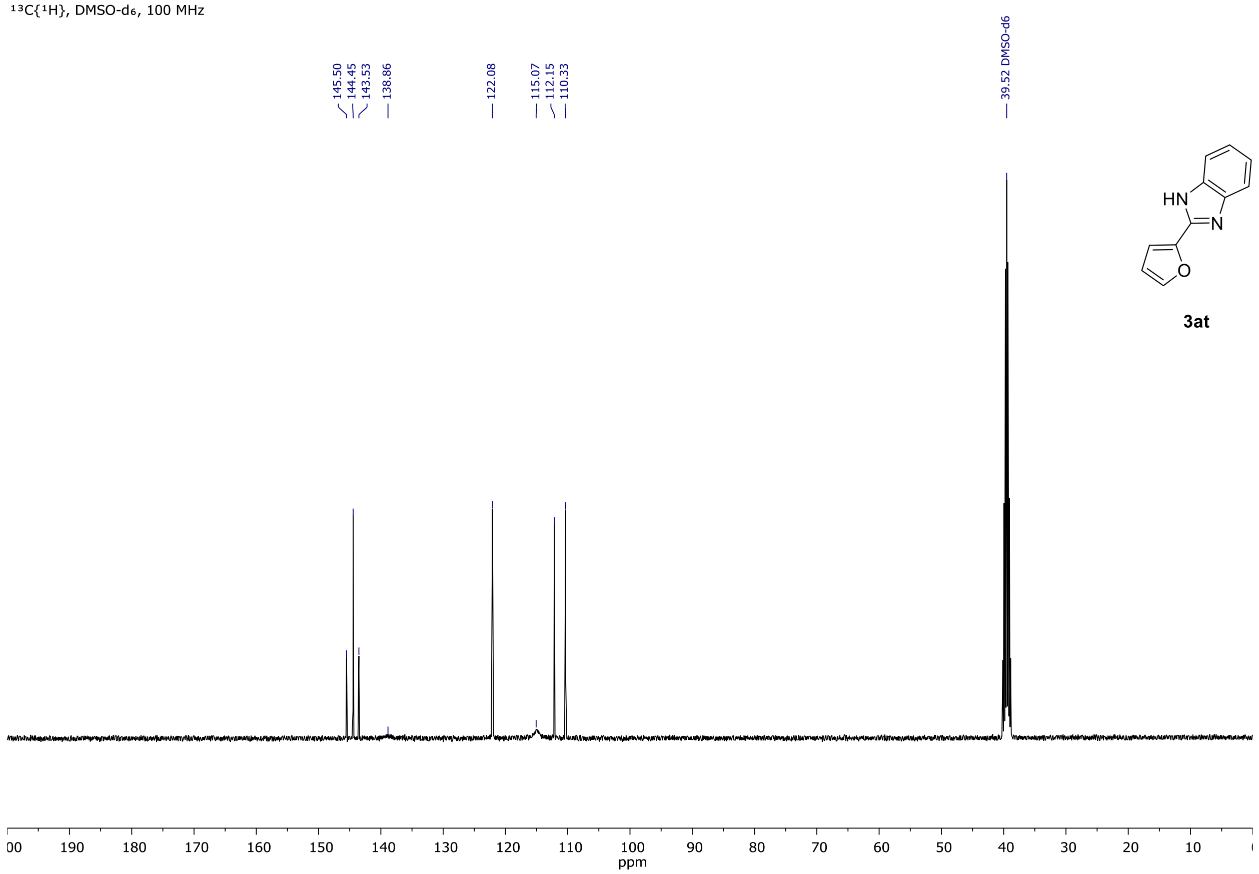

¹H, DMSO-d₆, 400 MHz

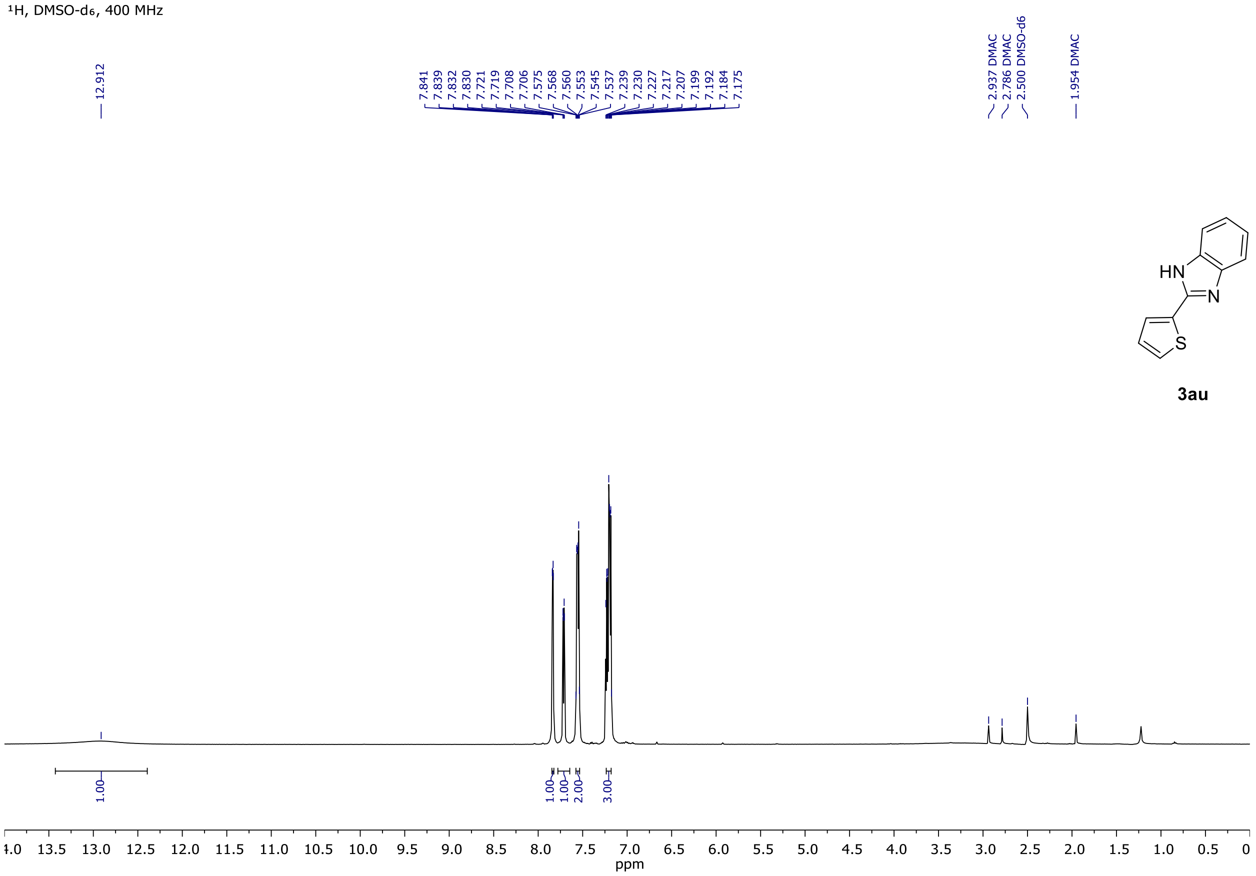

¹³C{¹H}, DMSO-d₆, 100 MHz

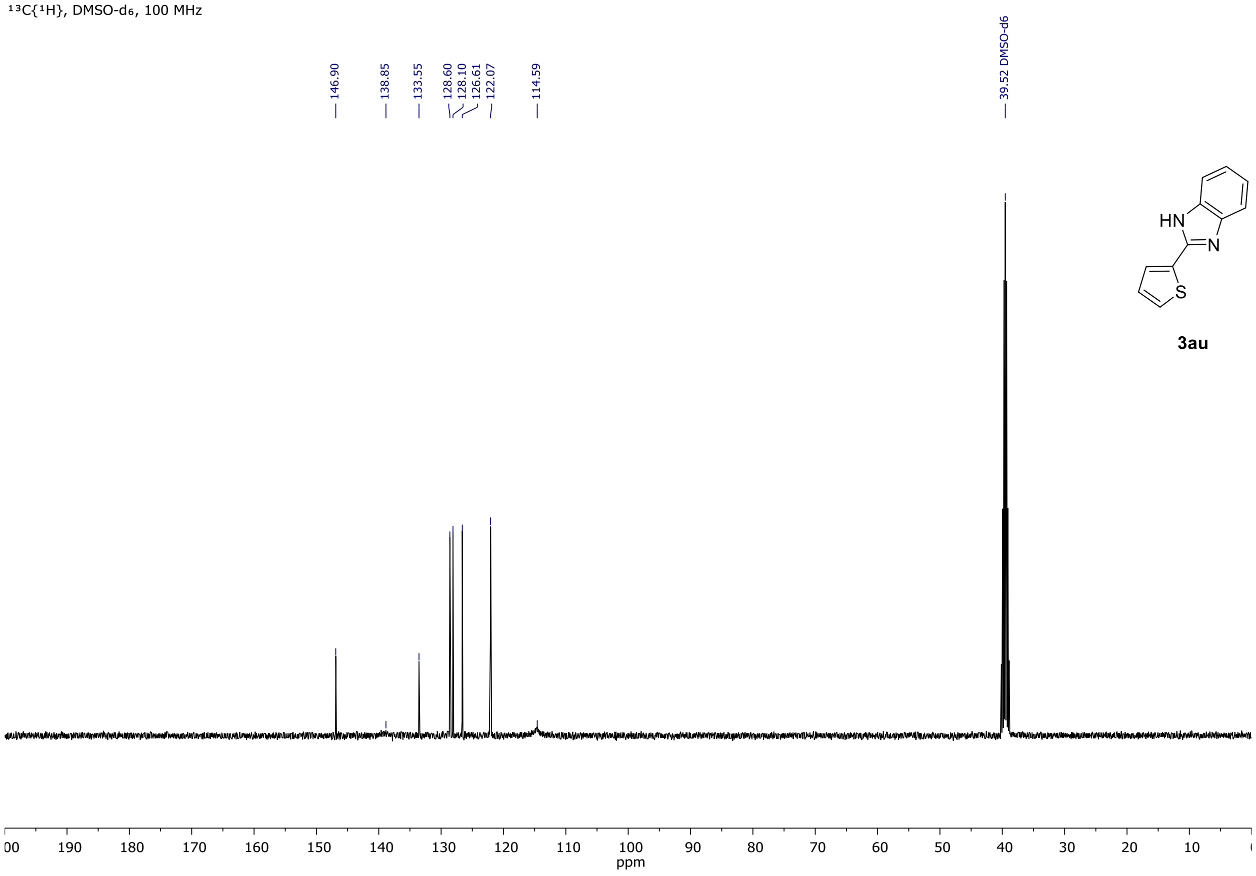

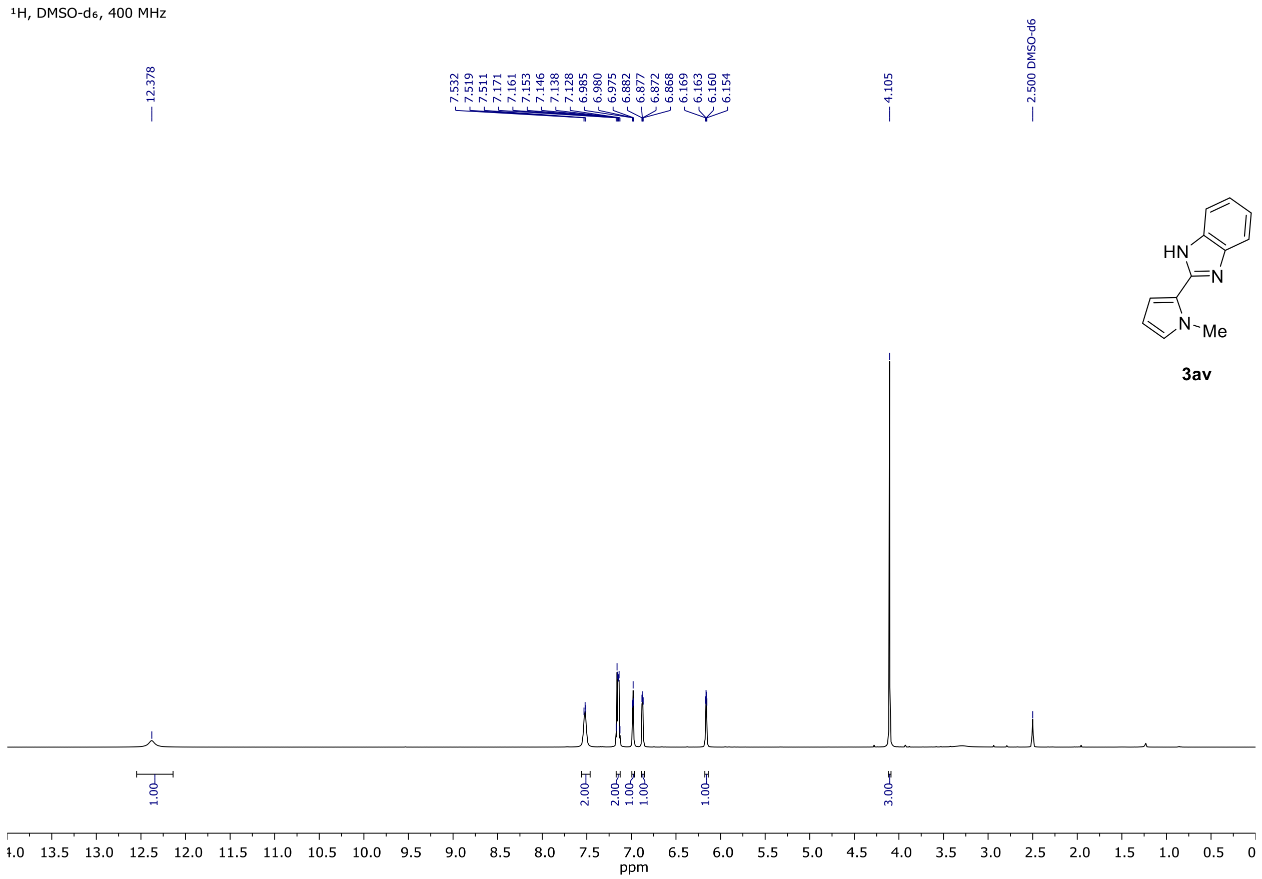

¹H, DMSO-d₆, 400 MHz
12.378
7.532 7.519 7.511 7.171 7.161 7.153 7.146 7.138 7.128 6.985 6.980 6.975 6.882 6.877 6.872 6.868 6.169 6.163 6.160 6.154
4.105
2.500 DMSO-d6
1.00 2.00 2.00 1.00 1.00 1.00 3.00
HN
N
N
Me
3av
ppm

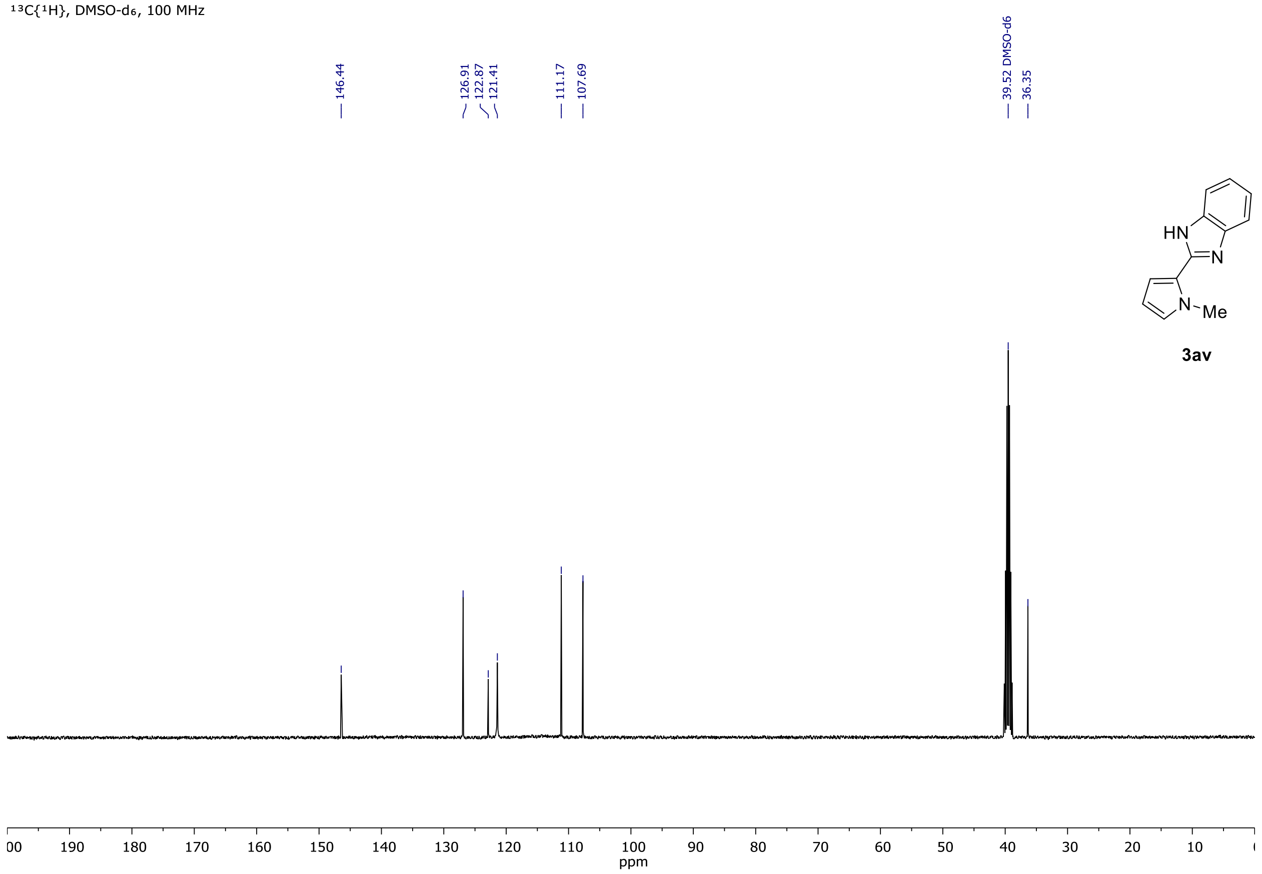

¹³C{¹H}, DMSO-d₆, 100 MHz
146.44
126.91 122.87 121.41
111.17 107.69
39.52 DMSO-d6
36.35
HN
N
N
Me
3av
ppm

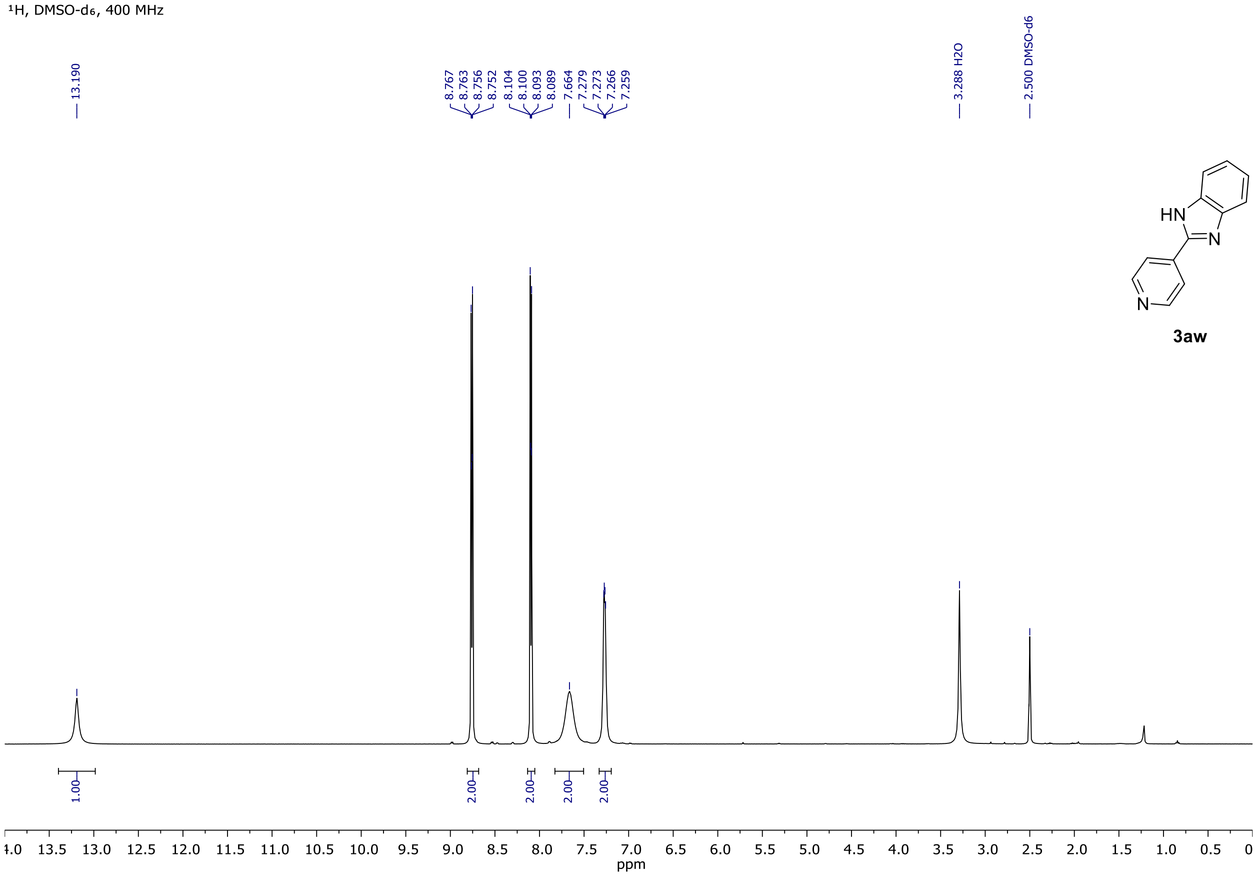
¹H, DMSO-d₆, 400 MHz
13.190
8.767
8.763
8.756
8.752
8.104
8.100
8.093
8.089
7.664
7.279
7.273
7.266
7.259
3.288 H2O
2.500 DMSO-d6
HN
N
N
1.00
2.00
2.00
2.00
2.00
ppm

3aw

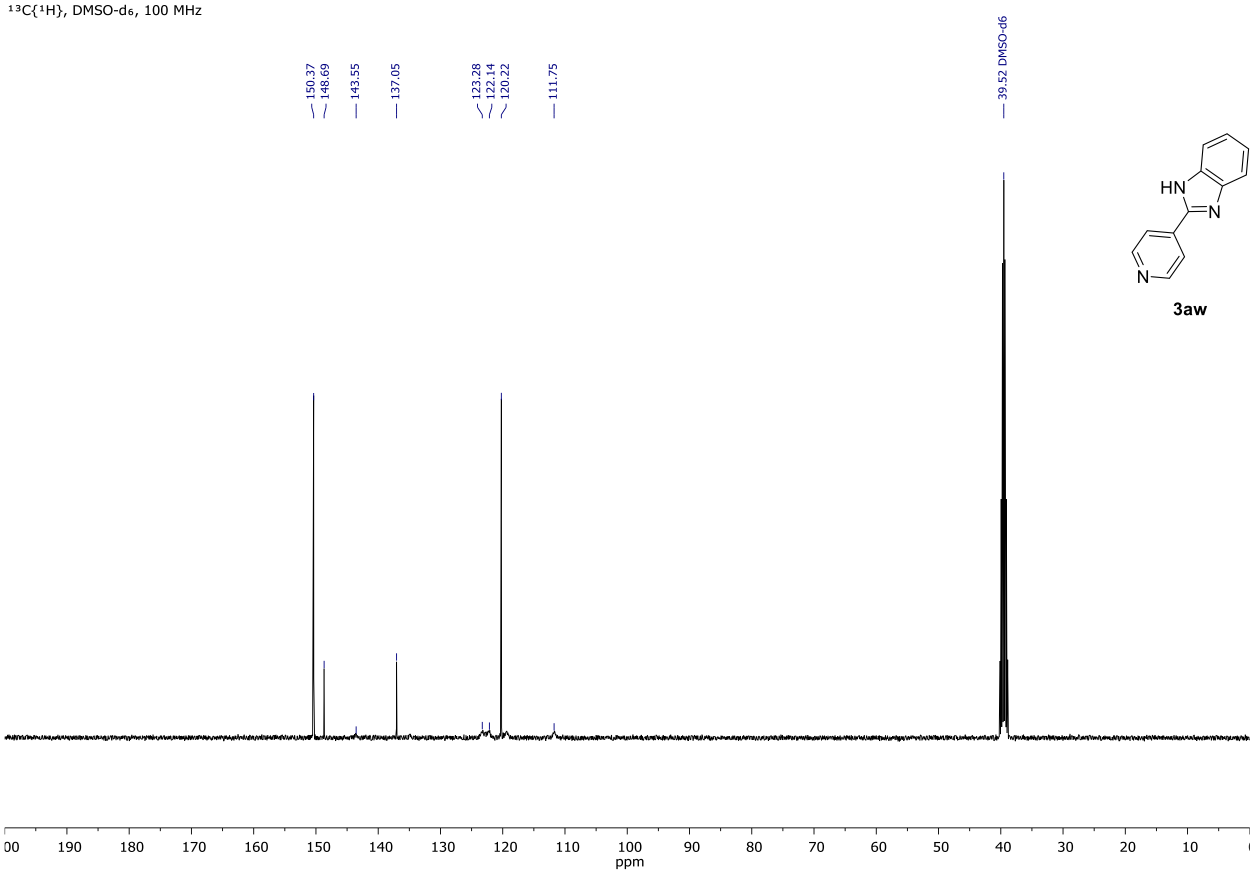
¹³C{¹H}, DMSO-d₆, 100 MHz
150.37
148.69
143.55
137.05
123.28
122.14
120.22
111.75
39.52 DMSO-d6
HN
N
N
ppm

3aw

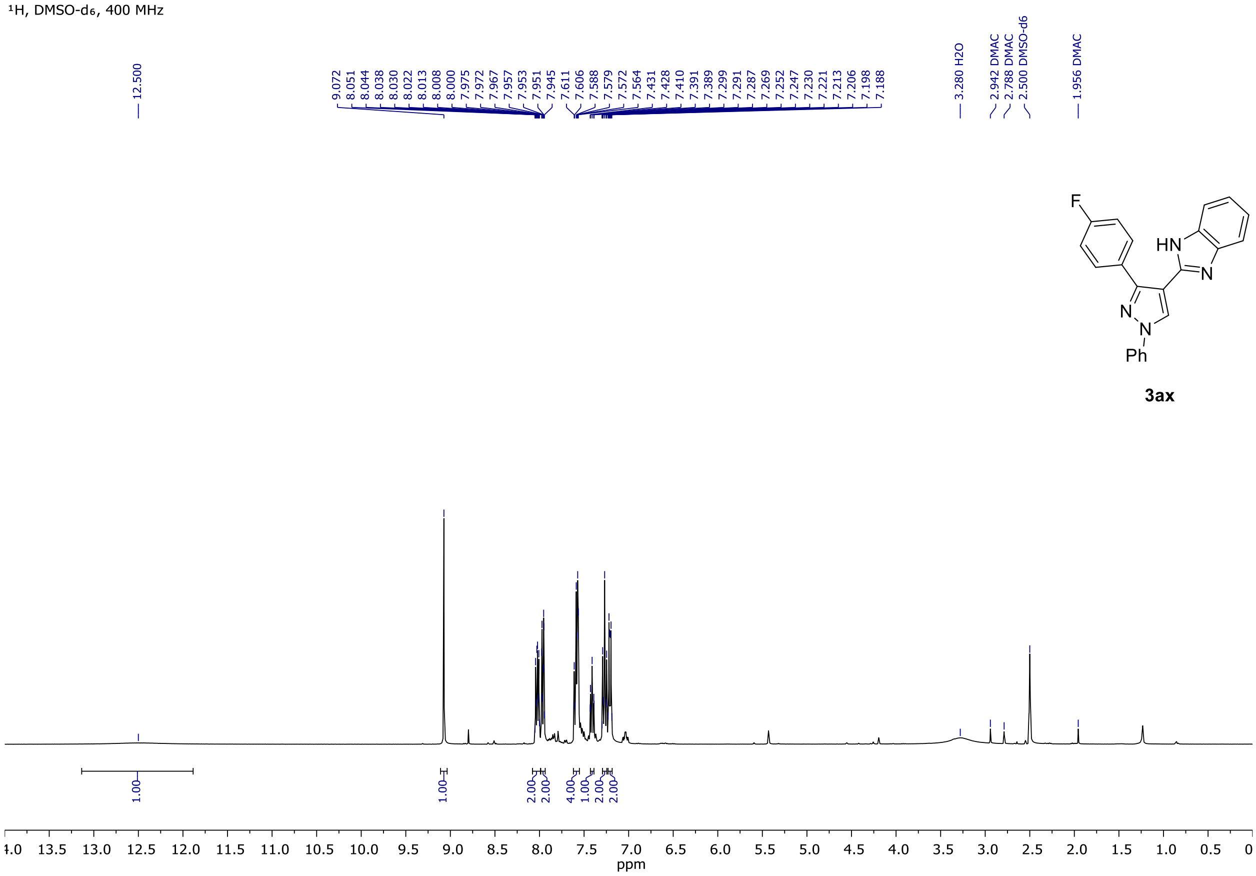

¹H, DMSO-d₆, 400 MHz
12.500
9.072 8.051 8.044 8.038 8.030 8.022 8.013 8.008 8.000 7.975 7.972 7.967 7.957 7.953 7.951 7.945 7.611 7.606 7.588 7.579 7.572 7.564 7.431 7.428 7.410 7.391 7.389 7.299 7.291 7.287 7.269 7.252 7.247 7.230 7.221 7.213 7.206 7.198 7.188
3.280 H2O
2.942 DMAC
2.788 DMAC
2.500 DMSO-d6
1.956 DMAC
F
HN
N
N
N
Ph
3ax
1.00
1.00
2.00
2.00
4.00
1.00
2.00
2.00
2.00
14.0 13.5 13.0 12.5 12.0 11.5 11.0 10.5 10.0 9.5 9.0 8.5 8.0 7.5 7.0 6.5 6.0 5.5 5.0 4.5 4.0 3.5 3.0 2.5 2.0 1.5 1.0 0.5 0
ppm

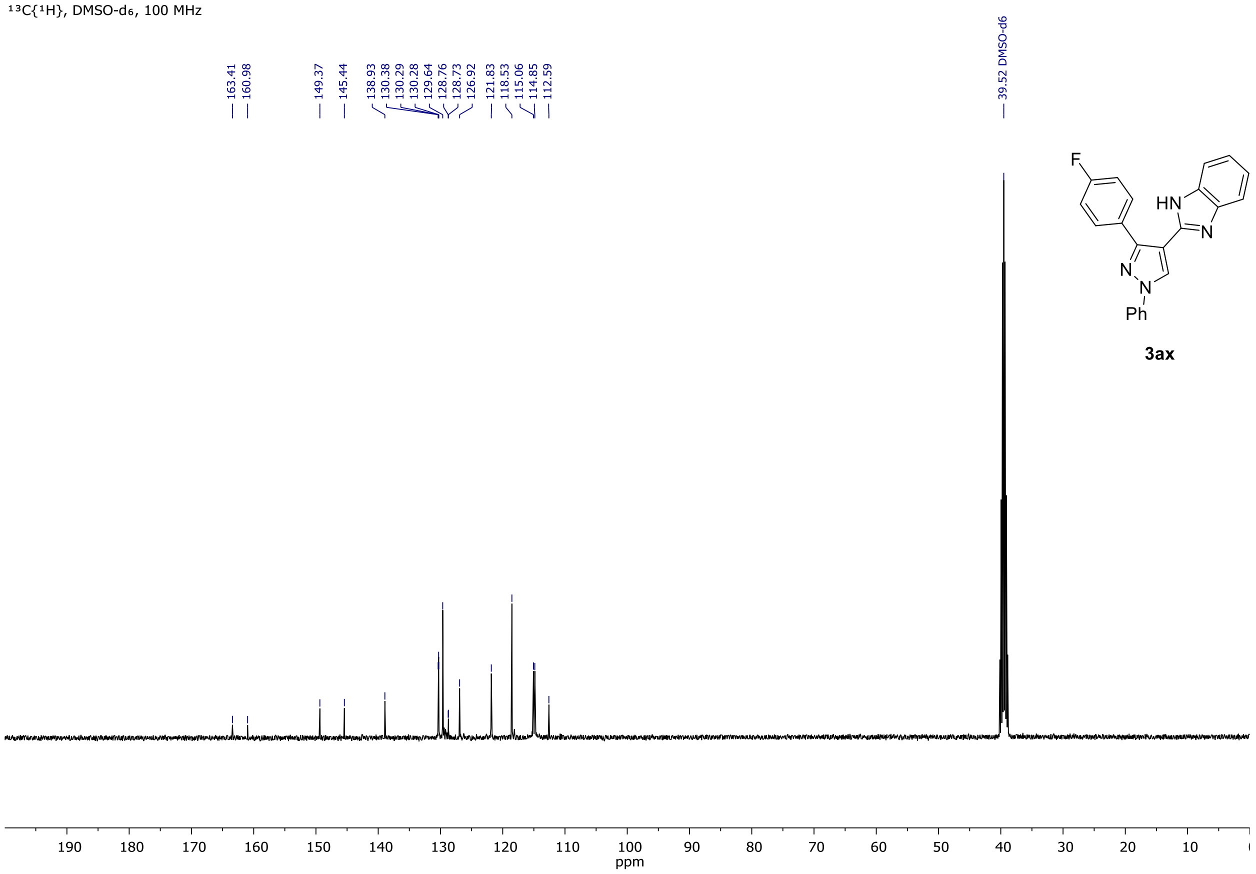

¹³C{¹H}, DMSO-d₆, 100 MHz
163.41 160.98 149.37 145.44 138.93 130.38 130.29 130.28 129.64 128.76 128.73 126.92 121.83 118.53 115.06 114.85 112.59
39.52 DMSO-d6
F
HN
N
N
N
Ph
3ax
190 180 170 160 150 140 130 120 110 100 90 80 70 60 50 40 30 20 10 0
ppm

^1H, DMSO-d_6, 400 MHz

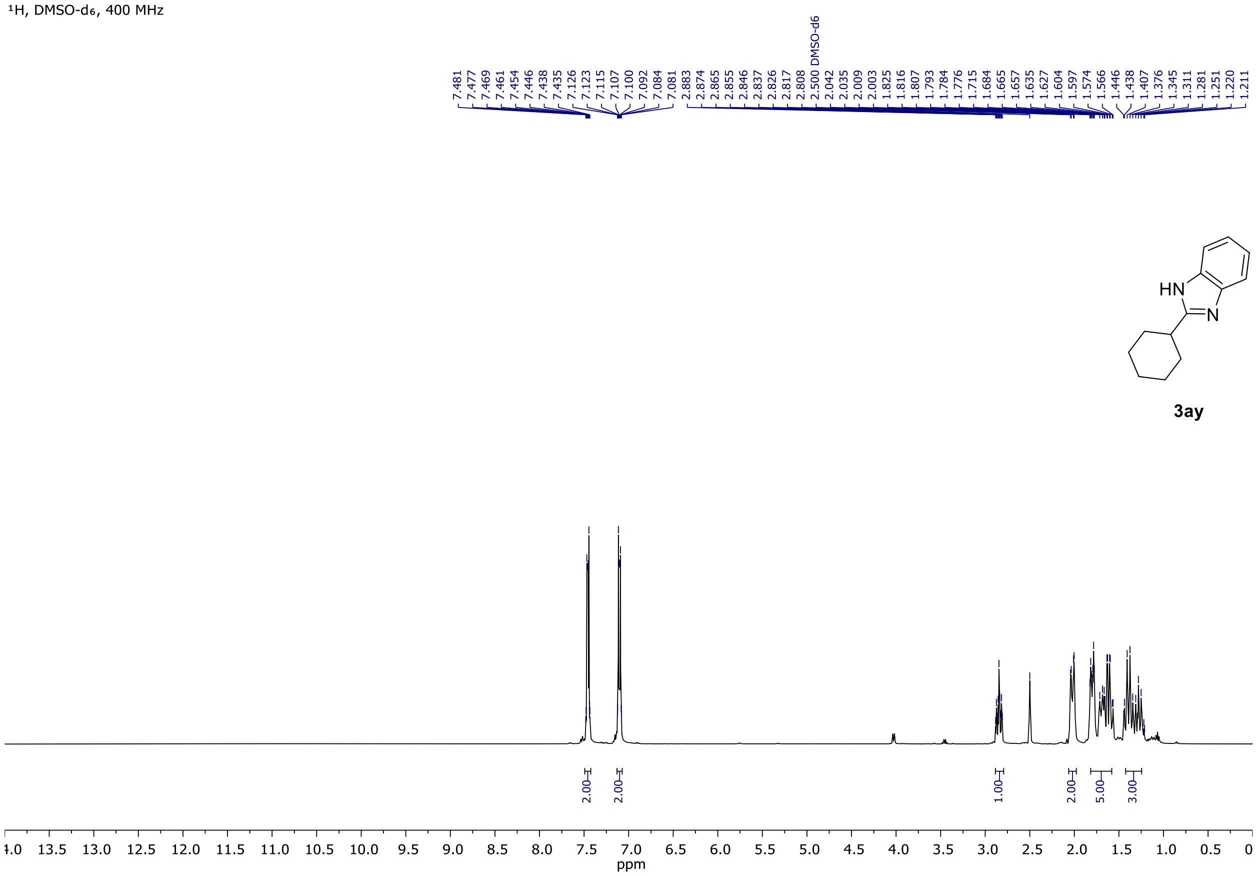

$^{13}C\{^1H\}$, DMSO-d_6, 100 MHz

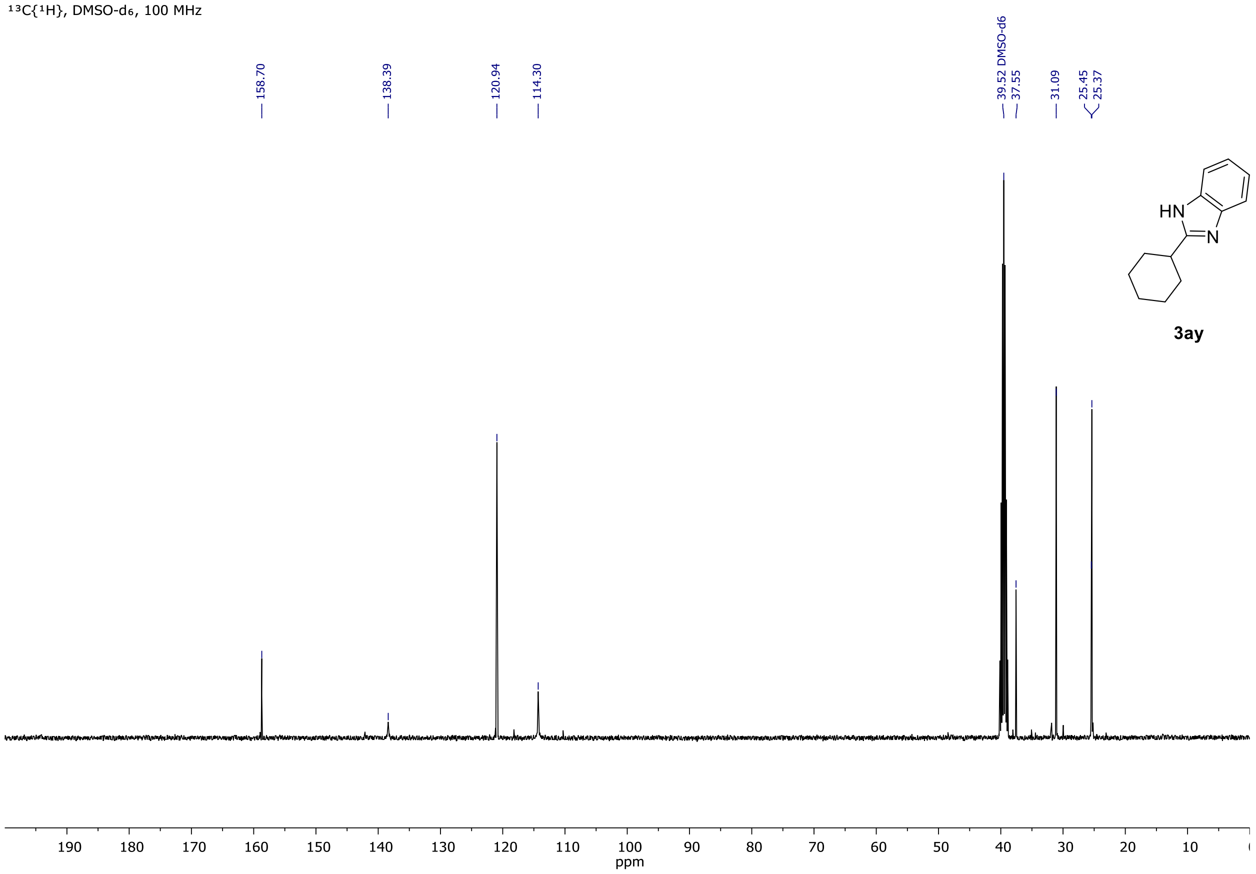

¹H, DMSO-d₆, 400 MHz

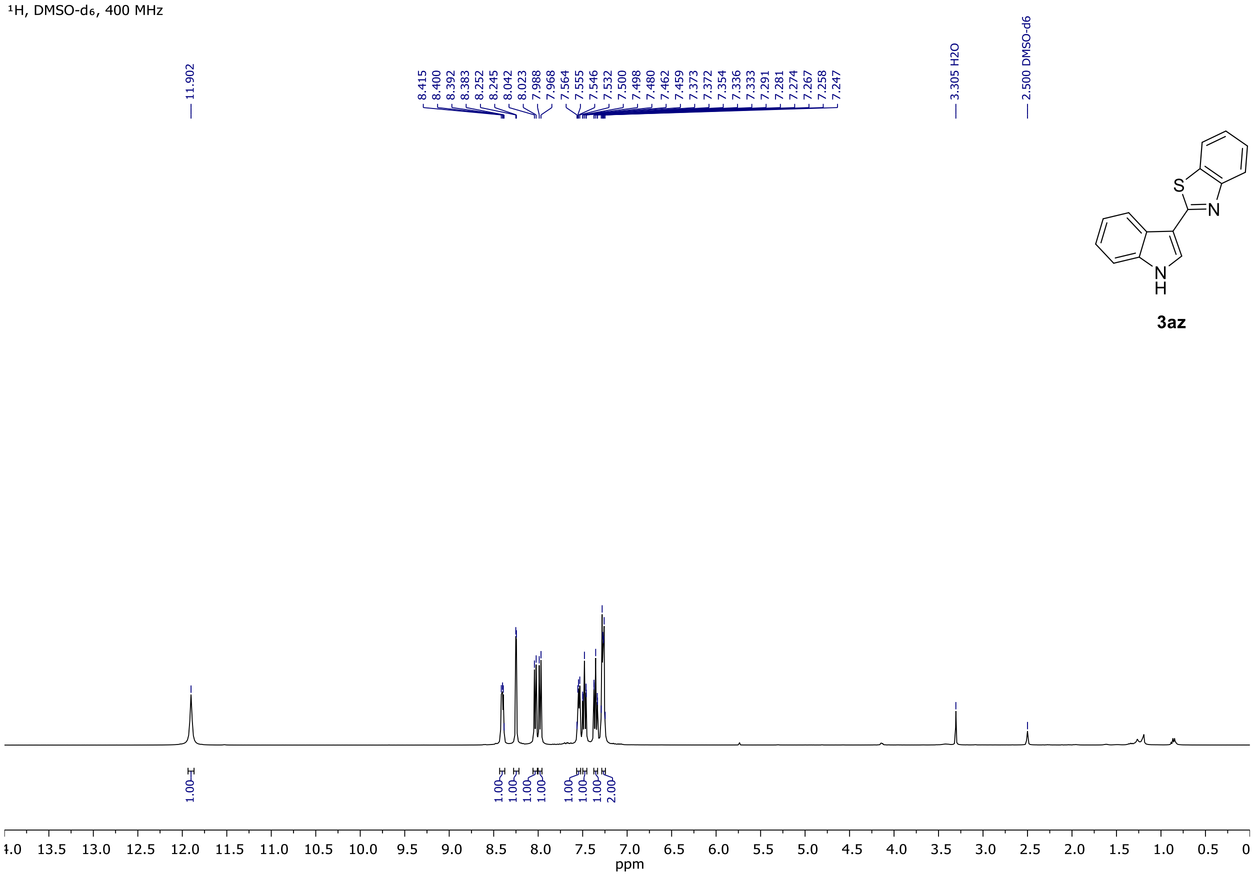

$^{13}C\{^1H\}$, DMSO-d₆, 100 MHz

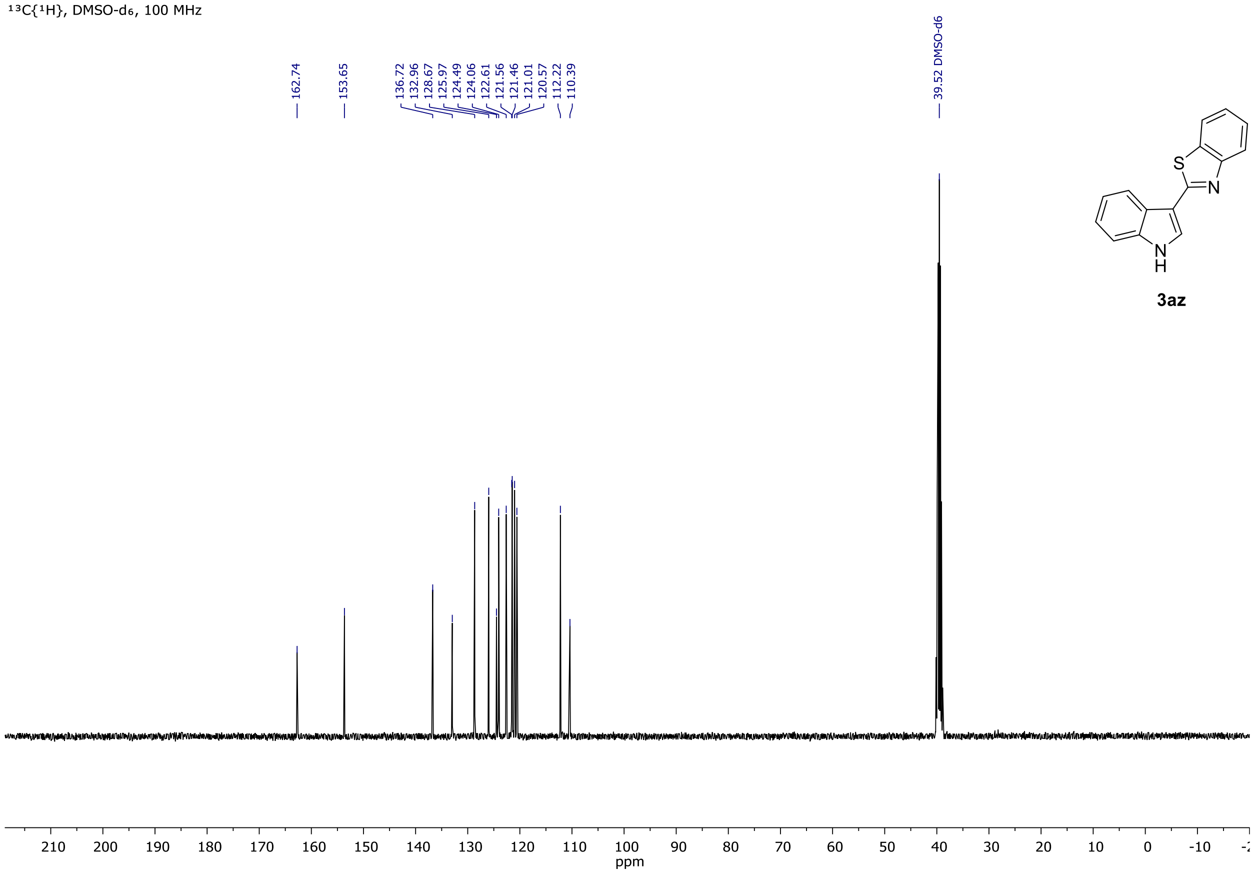

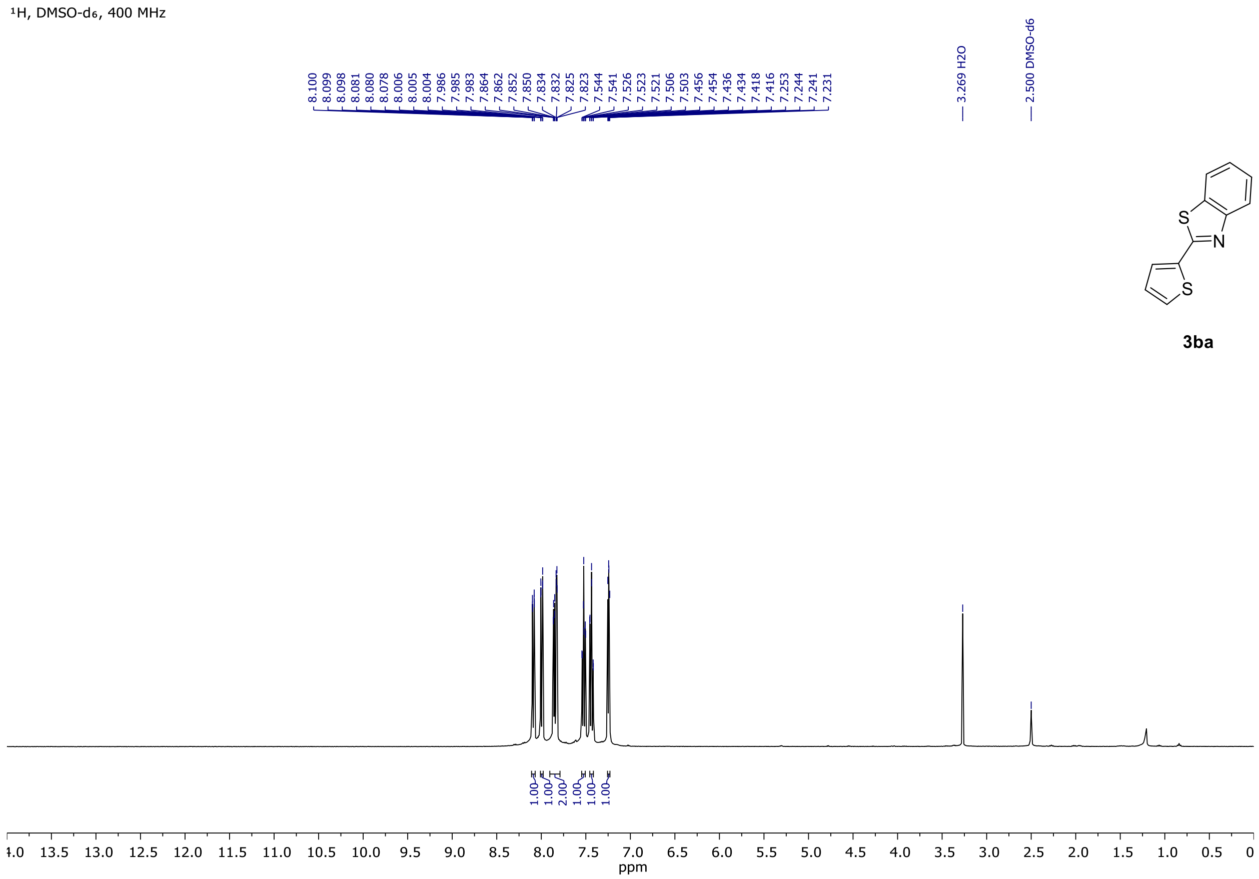
¹H, DMSO-d₆, 400 MHz
3.269 H2O
2.500 DMSO-d6
3ba
ppm

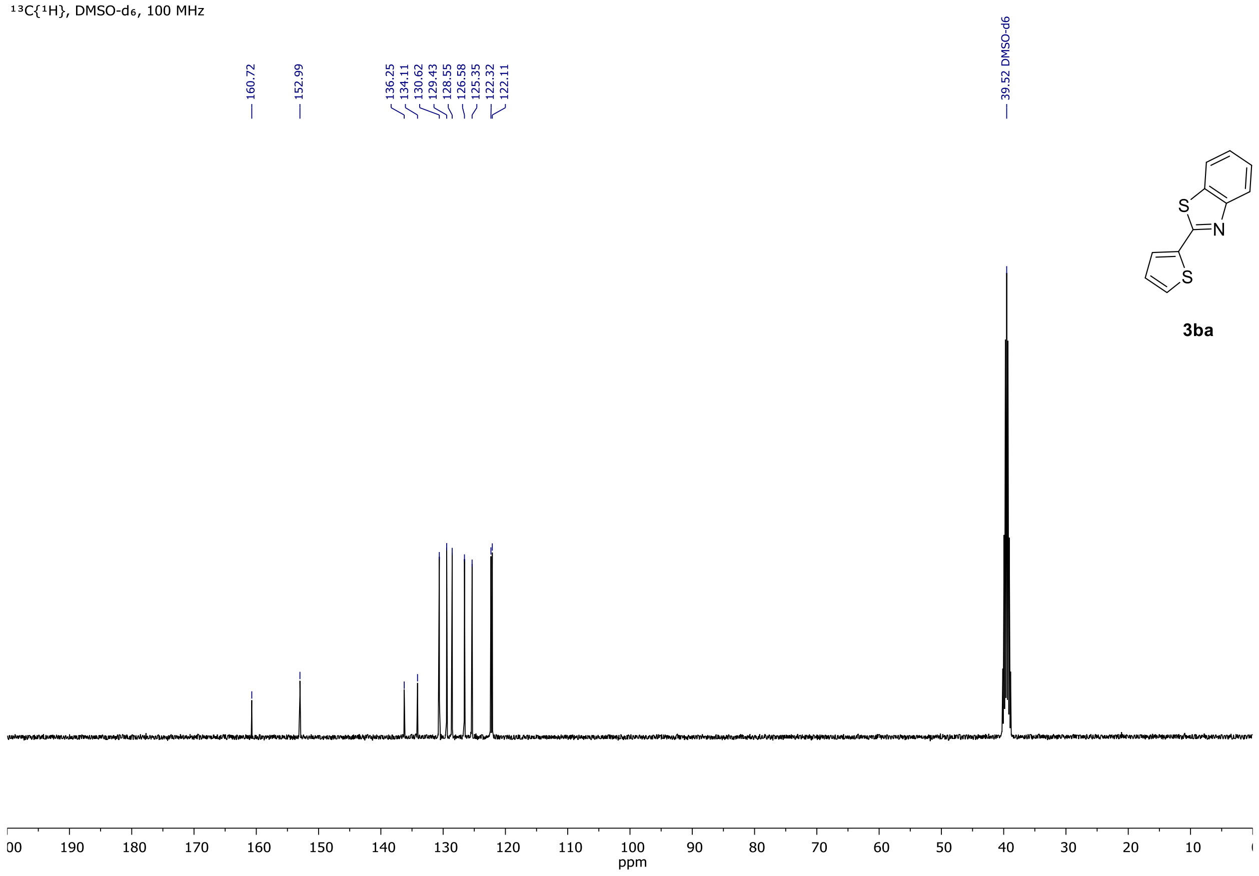
¹³C{¹H}, DMSO-d₆, 100 MHz
160.72
152.99
136.25
134.11
130.62
129.43
128.55
126.58
125.35
122.32
122.11
39.52 DMSO-d6
3ba
ppm

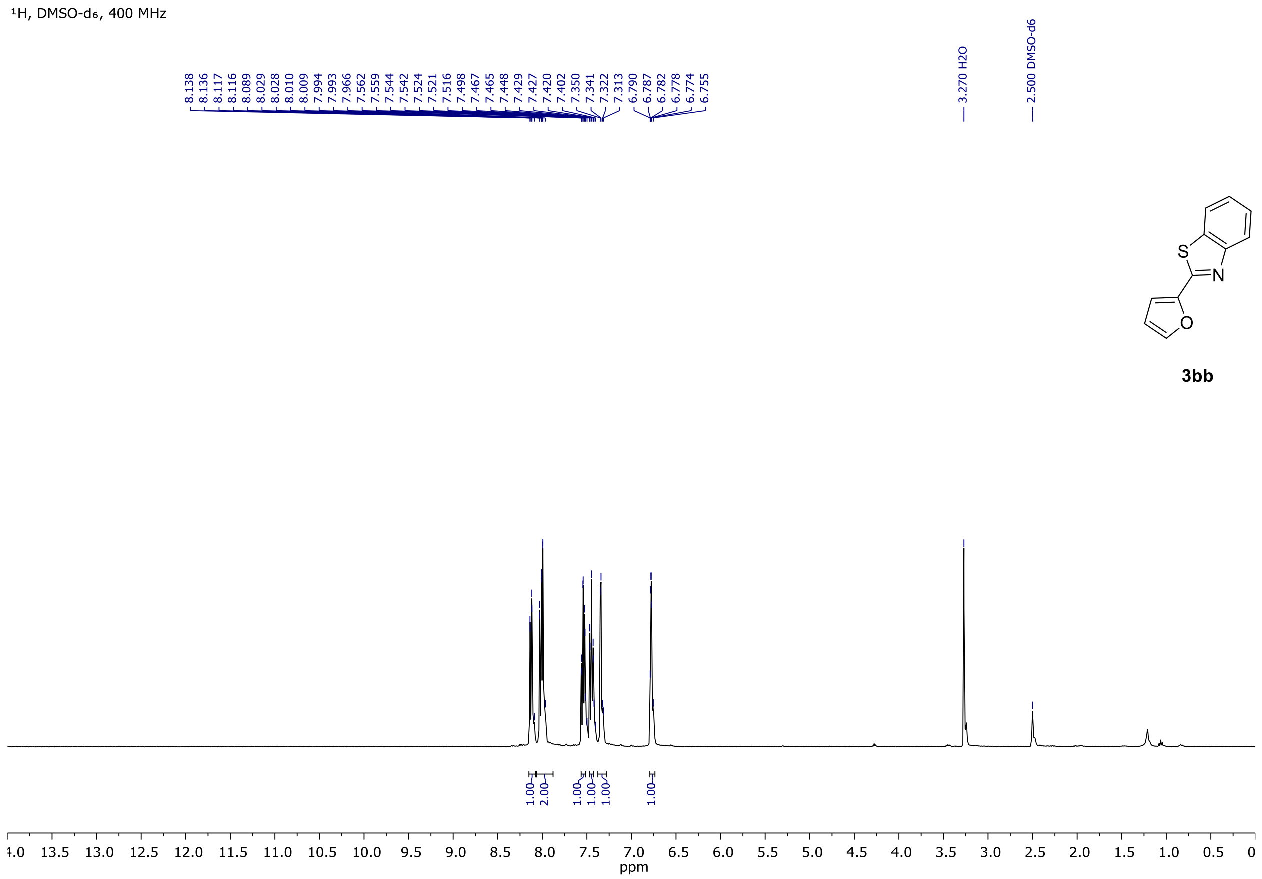
¹H, DMSO-d₆, 400 MHz
3.270 H2O
2.500 DMSO-d6
3bb
ppm

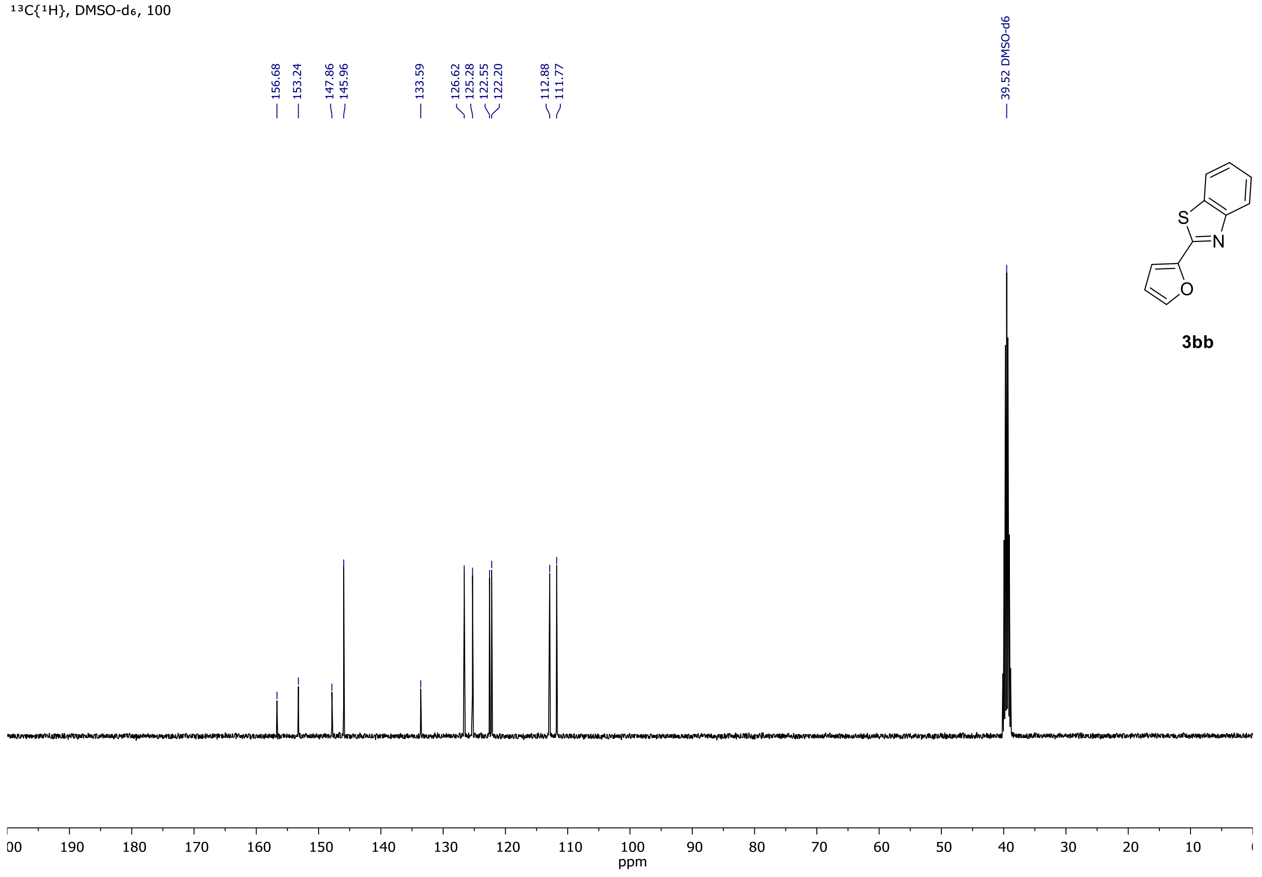
¹³C{¹H}, DMSO-d₆, 100
156.68
153.24
147.86
145.96
133.59
126.62
125.28
122.55
122.20
112.88
111.77
39.52 DMSO-d6
3bb
ppm

¹H, DMSO-d6, 400 MHz

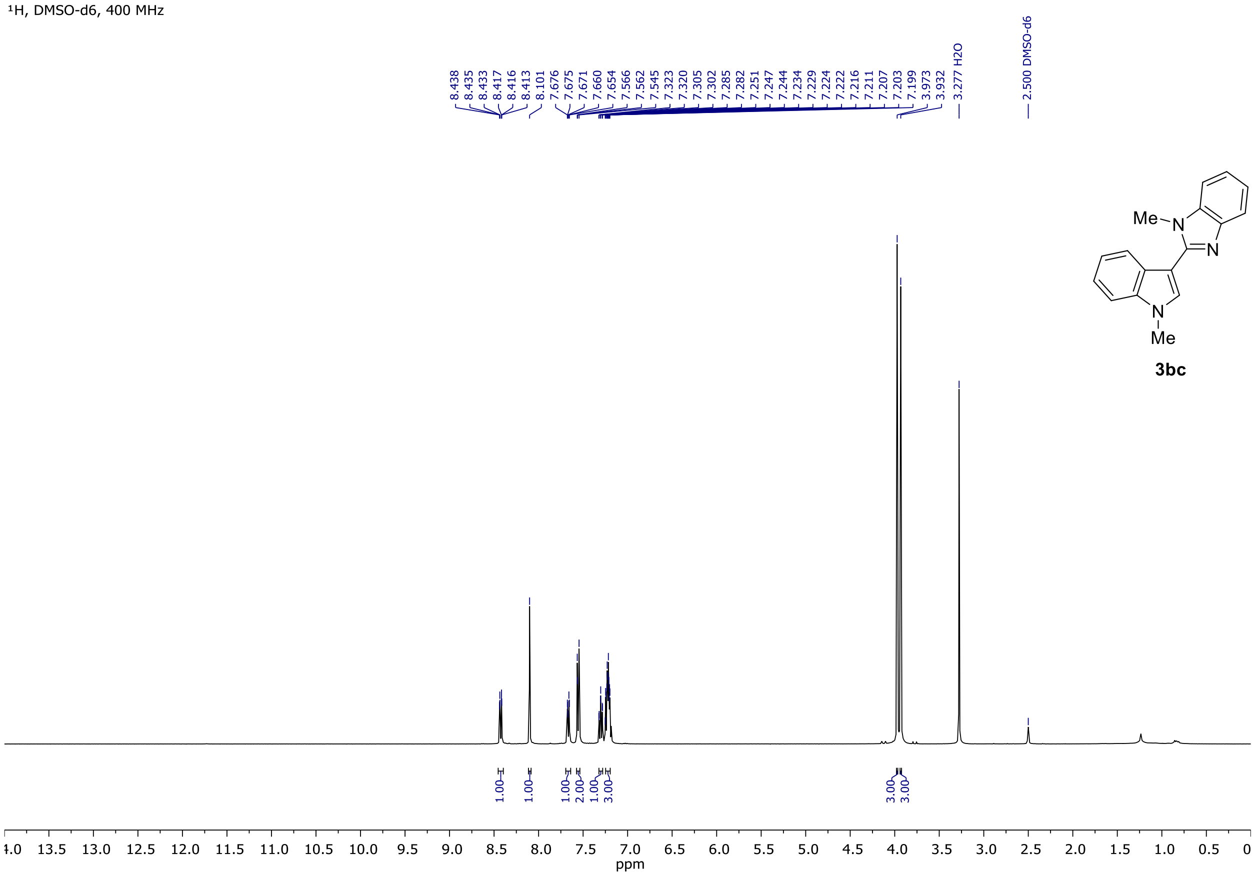

3bc

$^{13}C\{^{1}H\}$, DMSO-d₆, 100 MHz

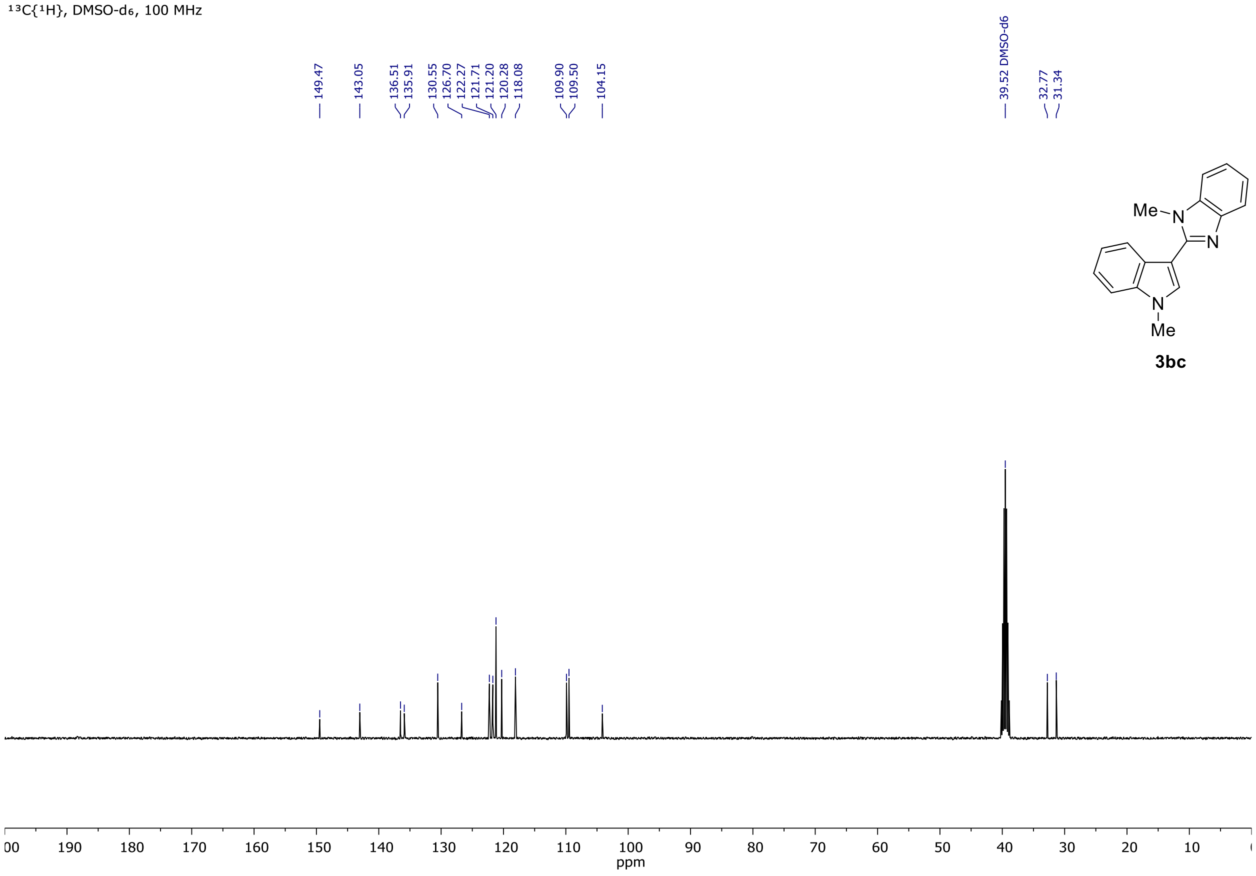

3bc

¹H, DMSO-d6, 400 MHz

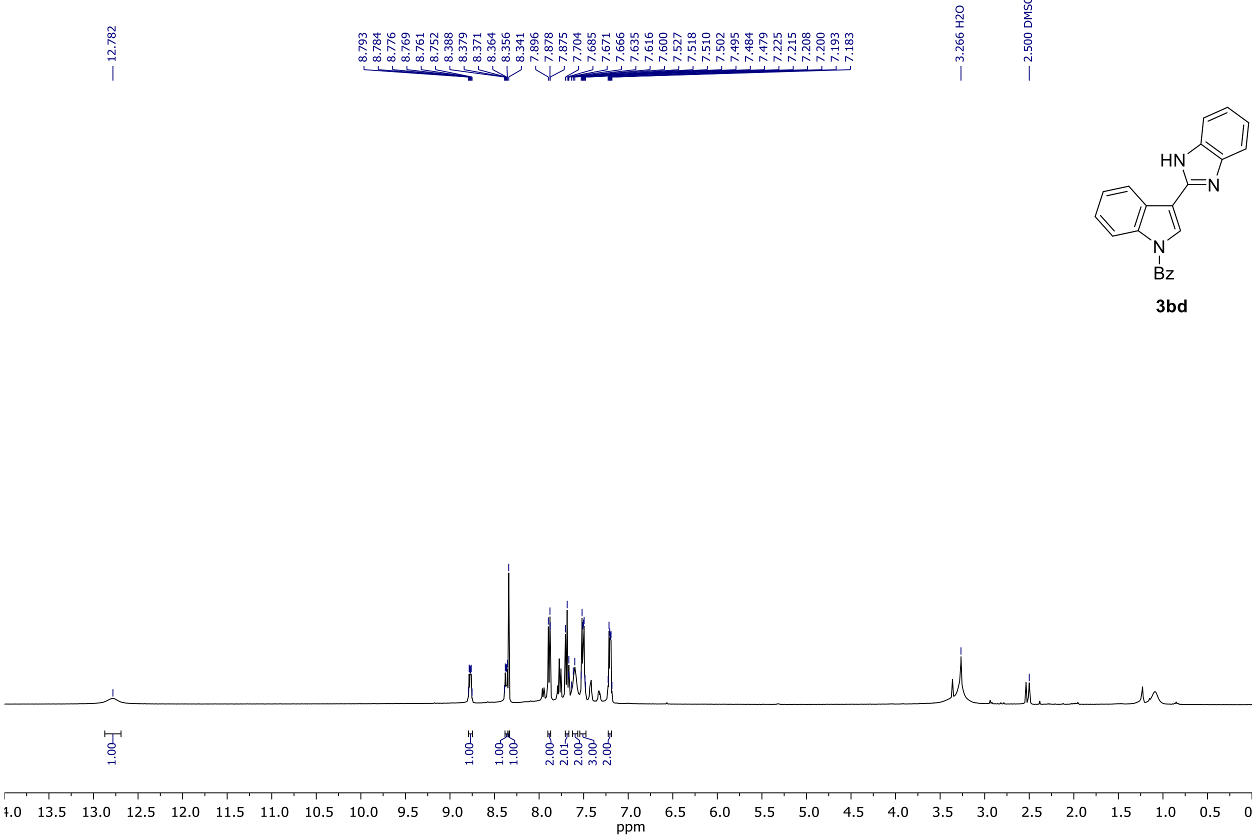

3bd

$^{13}C\{^{1}H\}$, DMSO-d_6, 100 MHz

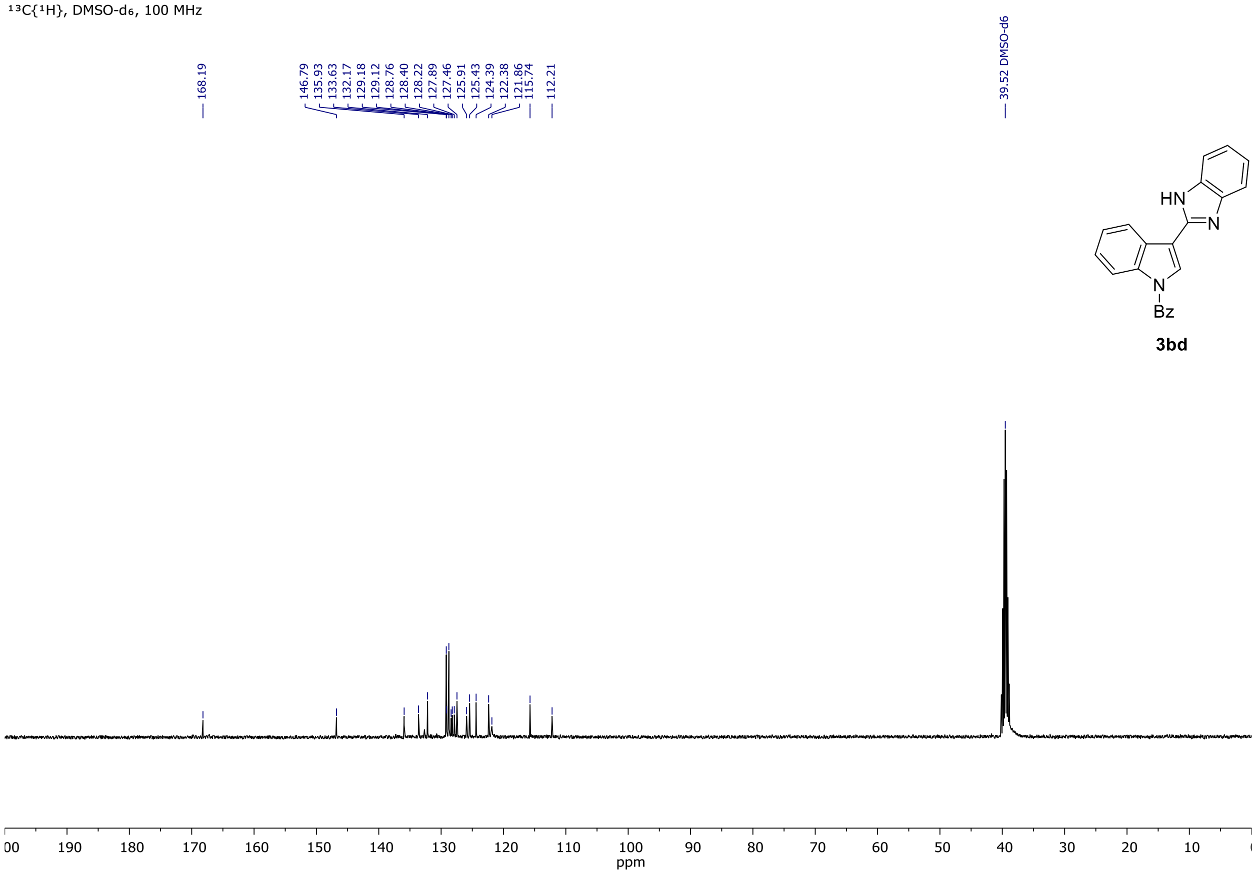

3bd

^{1}H, DMSO-d_6, 400 MHz

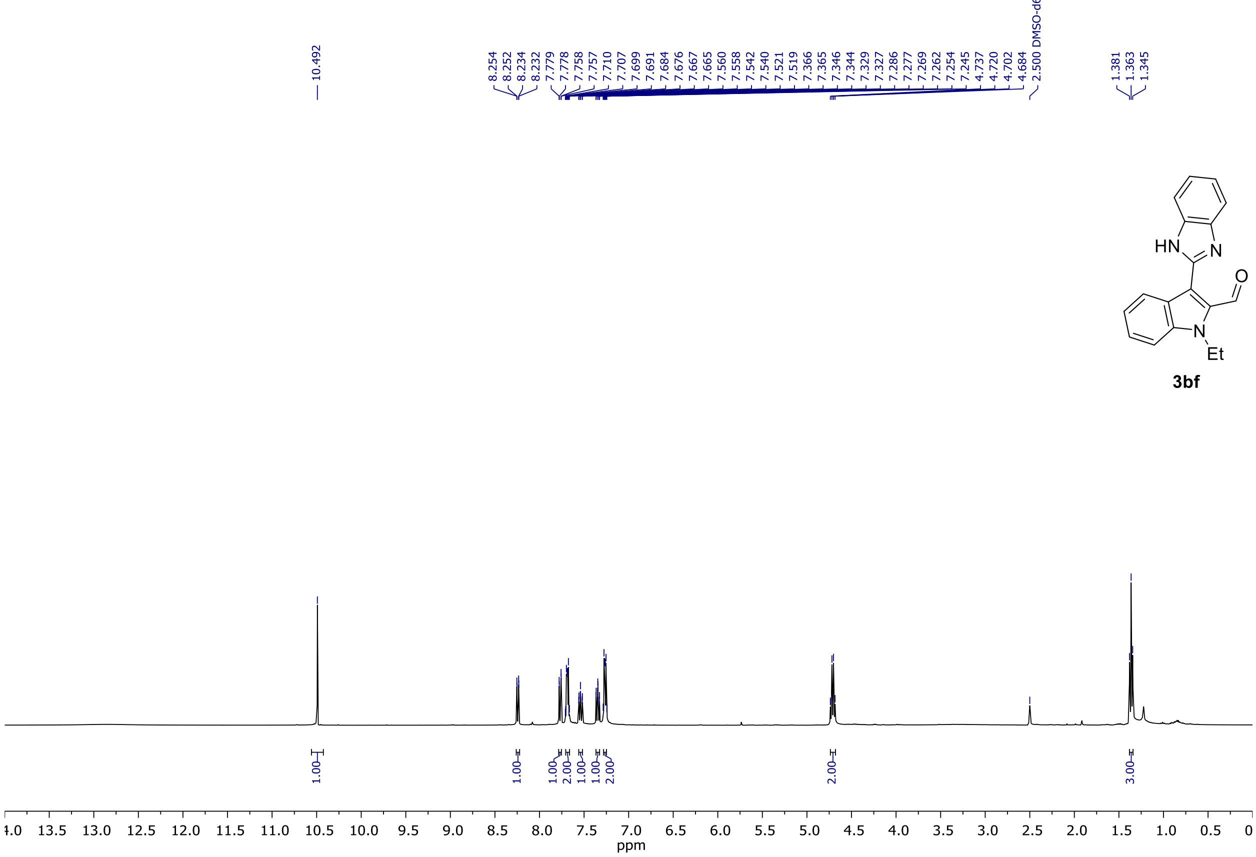

$^{13}C\{^{1}H\}$, DMSO-d_6, 100 MHz

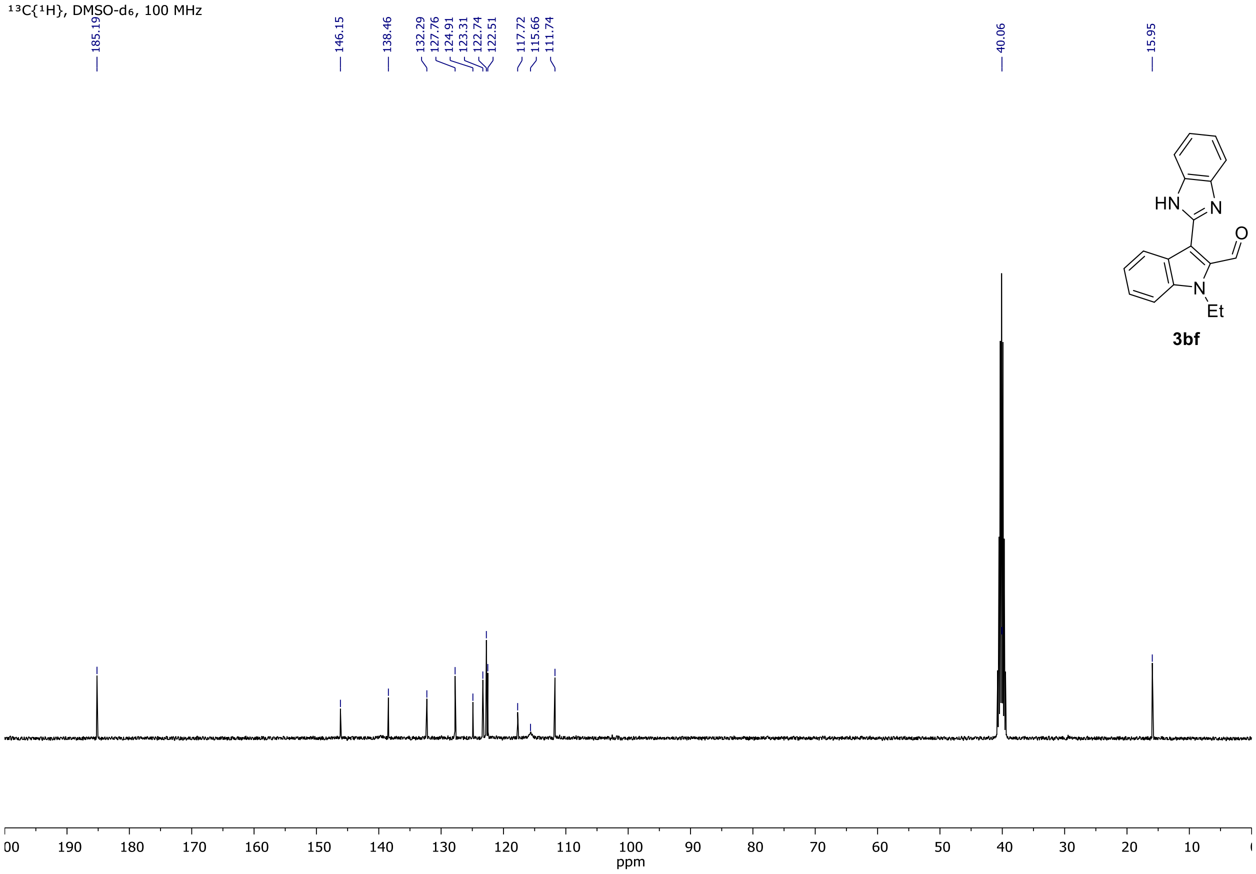

Copies of HRMS of new compounds

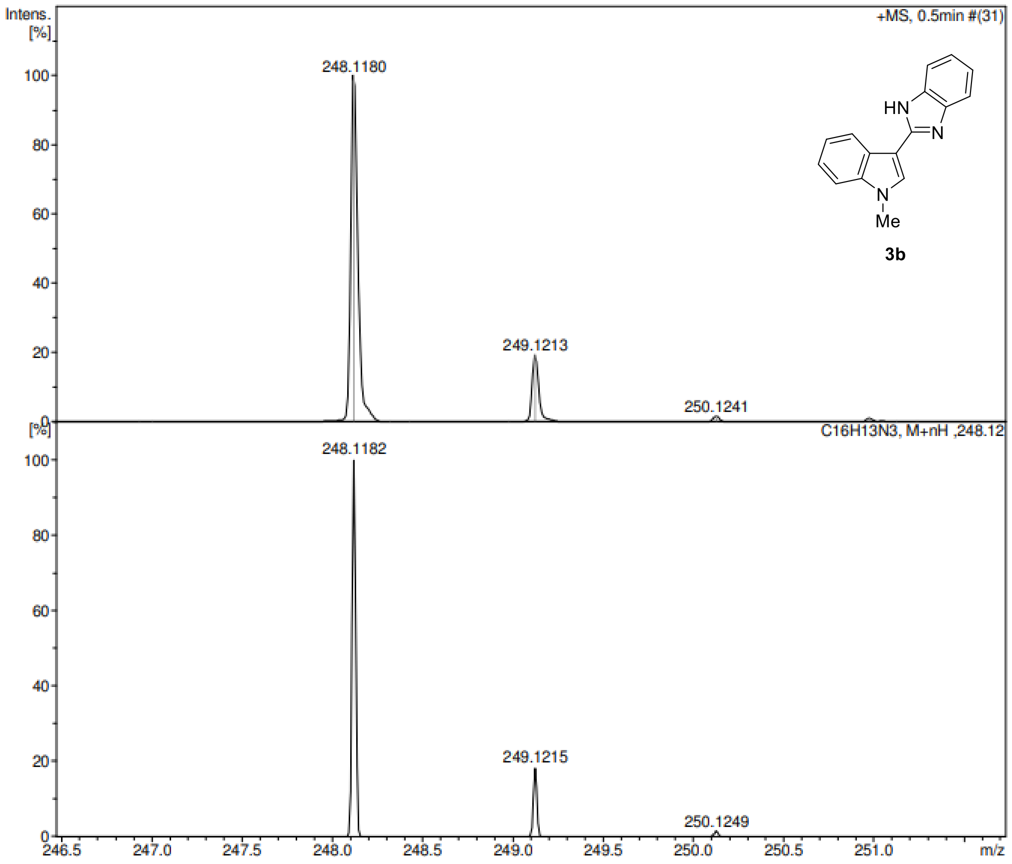

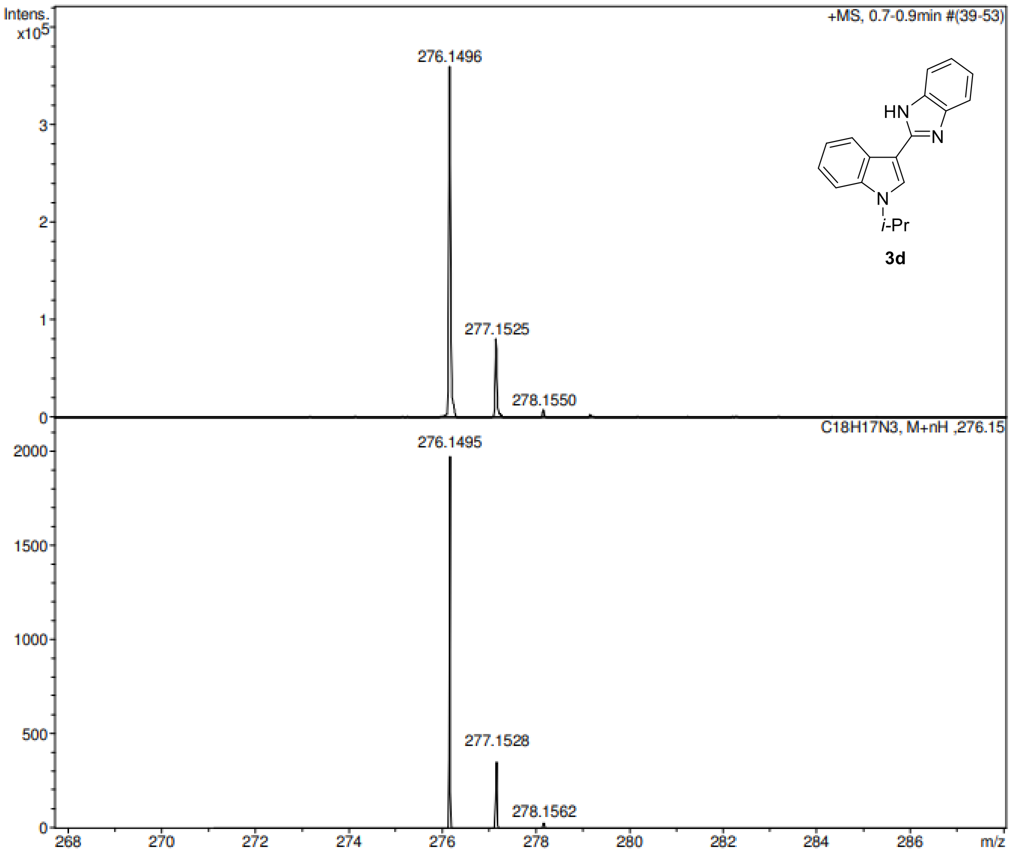

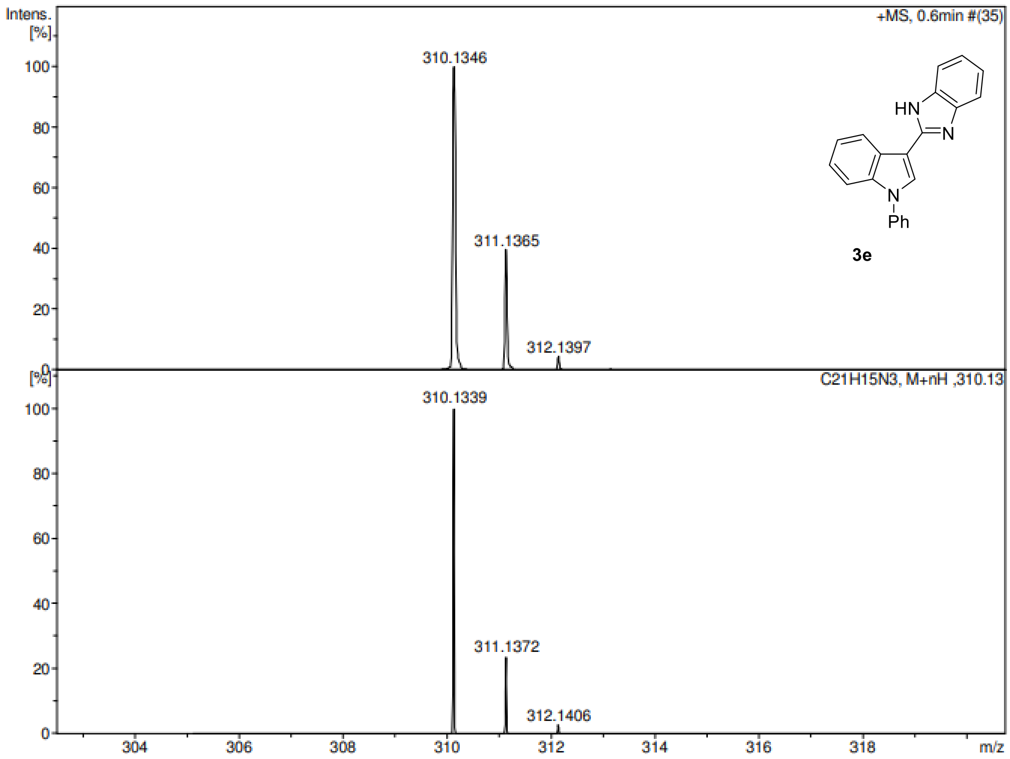
Intens. [%]
+MS, 0.6min #(35)
310.1346
311.1365
312.1397
HN
N
N
Ph
3e
C21H15N3, M+nH ,310.13
310.1339
311.1372
312.1406
m/z

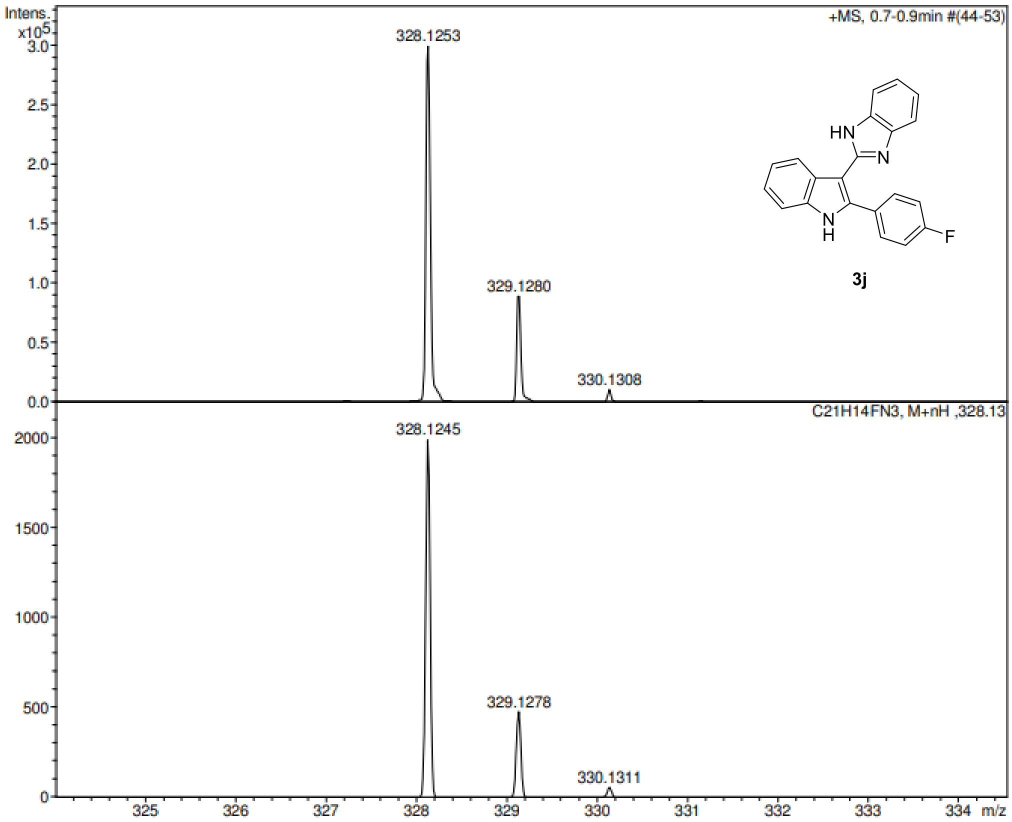
Intens. x10^5
+MS, 0.7-0.9min #(44-53)
328.1253
329.1280
330.1308
HN
N
N
H
F
3j
C21H14FN3, M+nH ,328.13
328.1245
329.1278
330.1311
m/z

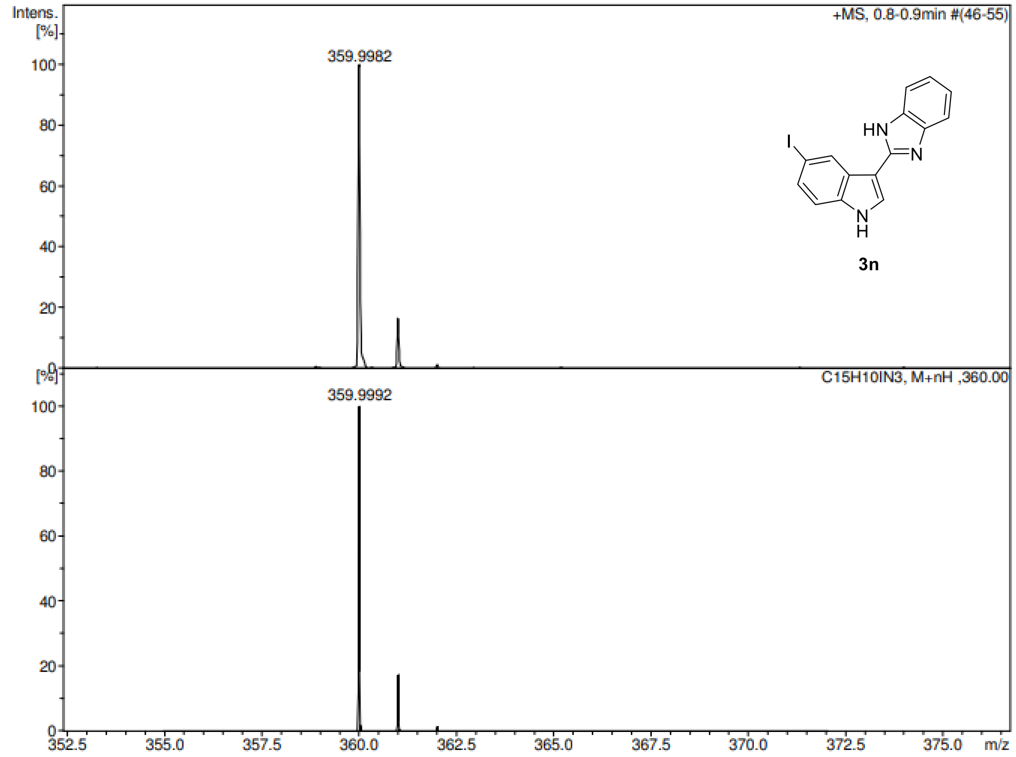
Intens. [%]
+MS, 0.8-0.9min #(46-55)
359.9982
C15H10IN3, M+nH ,360.00
359.9992
352.5
355.0
357.5
360.0
362.5
365.0
367.5
370.0
372.5
375.0
m/z
HN
N
I
N
H
3n

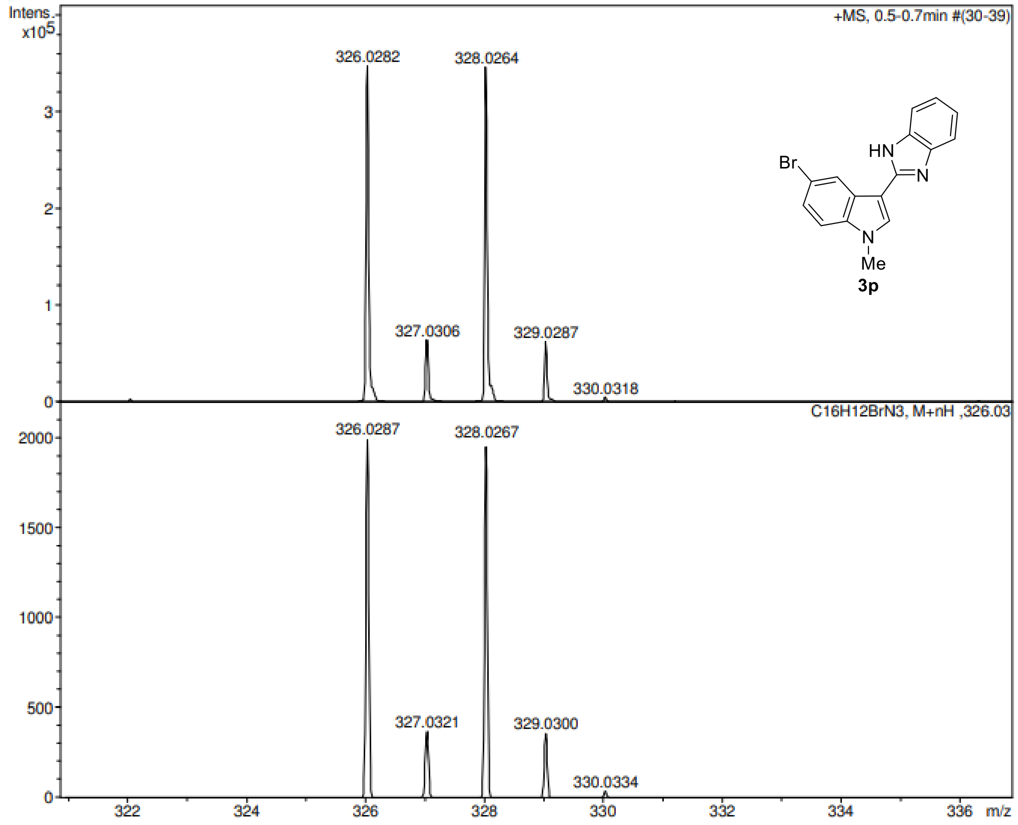
Intens. x10^5
+MS, 0.5-0.7min #(30-39)
326.0282
328.0264
327.0306
329.0287
330.0318
C16H12BrN3, M+nH ,326.03
326.0287
328.0267
327.0321
329.0300
330.0334
322
324
326
328
330
332
334
336
m/z
Br
HN
N
N
Me
3p

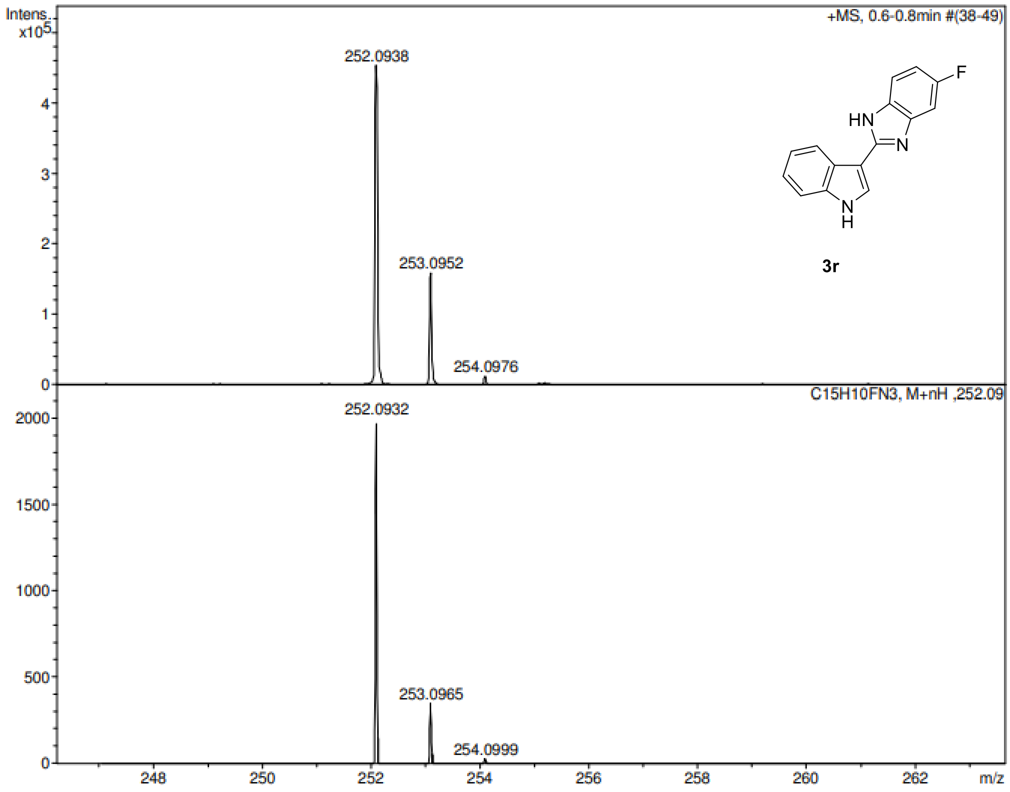

Intens.
x10^5
+MS, 0.6-0.8min #(38-49)
252.0938
253.0952
254.0976
3r
C15H10FN3, M+nH ,252.09
252.0932
253.0965
254.0999
248
250
252
254
256
258
260
262
m/z

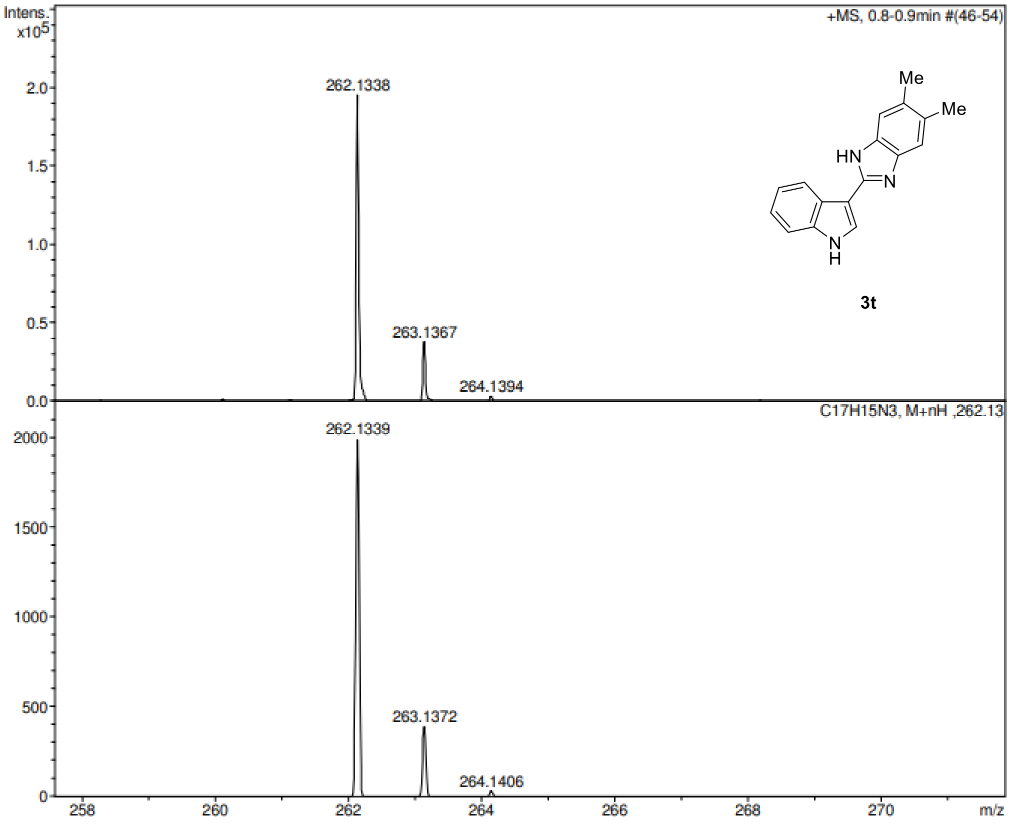

Intens.
x10^5
+MS, 0.8-0.9min #(46-54)
262.1338
263.1367
264.1394
3t
C17H15N3, M+nH ,262.13
262.1339
263.1372
264.1406
258
260
262
264
266
268
270
m/z

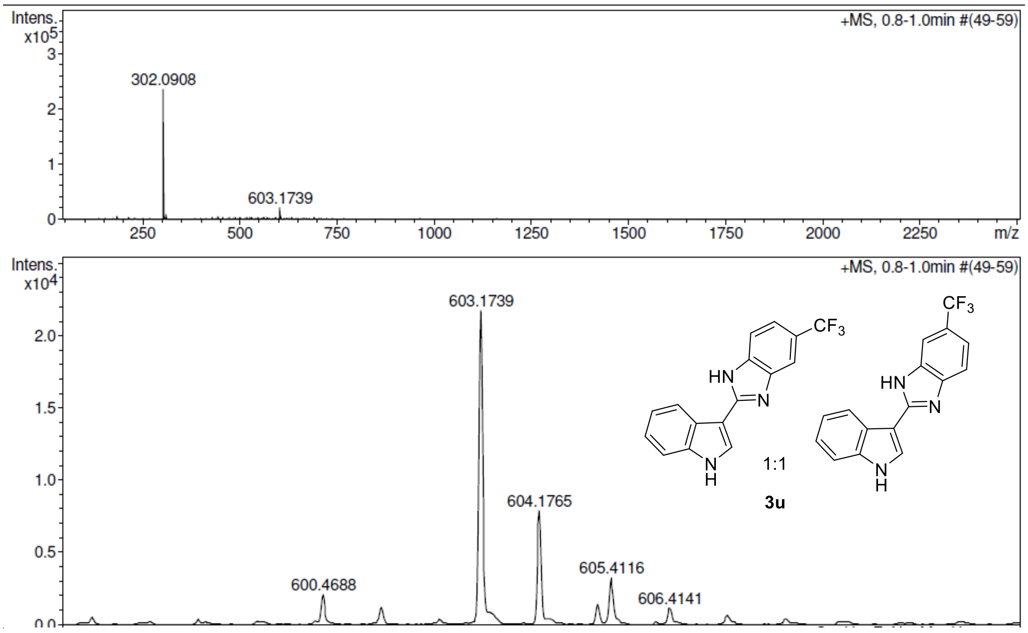
Intens.
x10^5
+MS, 0.8-1.0min #(49-59)
302.0908
603.1739
m/z
Intens.
x10^4
+MS, 0.8-1.0min #(49-59)
603.1739
604.1765
600.4688
605.4116
606.4141
CF3
HN
N
H
1:1
3u

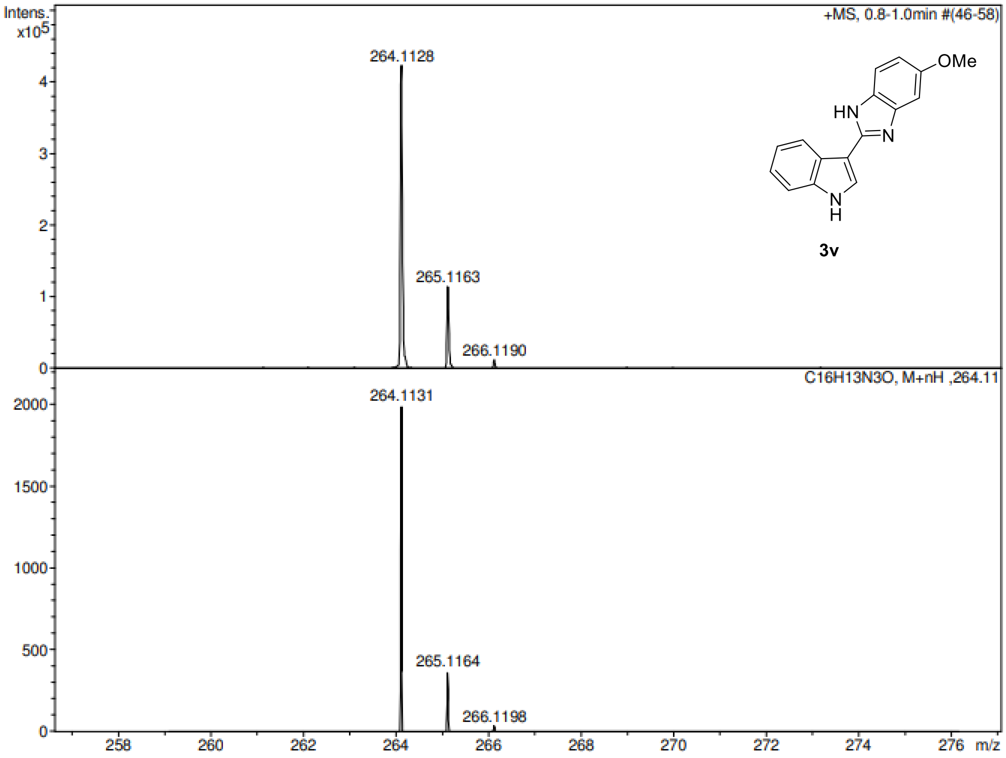
Intens.
x10^5
+MS, 0.8-1.0min #(46-58)
264.1128
265.1163
266.1190
OMe
HN
N
H
3v
C16H13N3O, M+nH ,264.11
264.1131
265.1164
266.1198
m/z

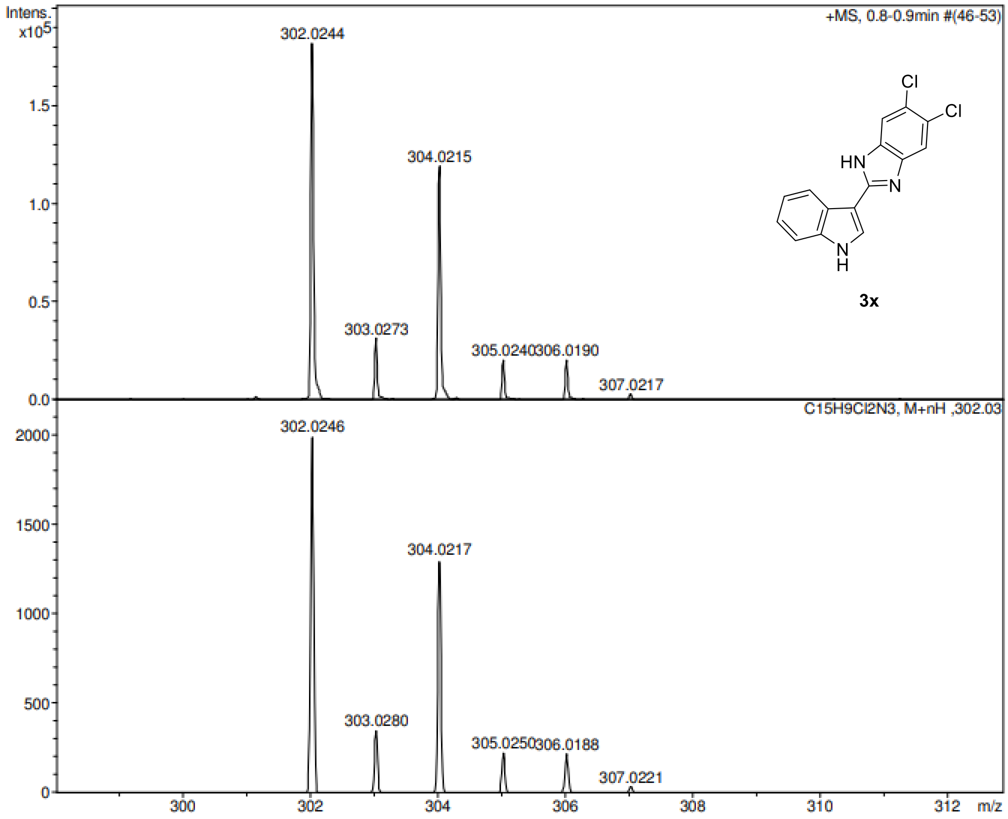
Intens.
x10^5
+MS, 0.8-0.9min #(46-53)
302.0244
304.0215
303.0273
305.0240
306.0190
307.0217
Cl
Cl
HN
N
N
H
3x
C15H9Cl2N3, M+nH ,302.03
302.0246
304.0217
303.0280
305.0250
306.0188
307.0221
m/z

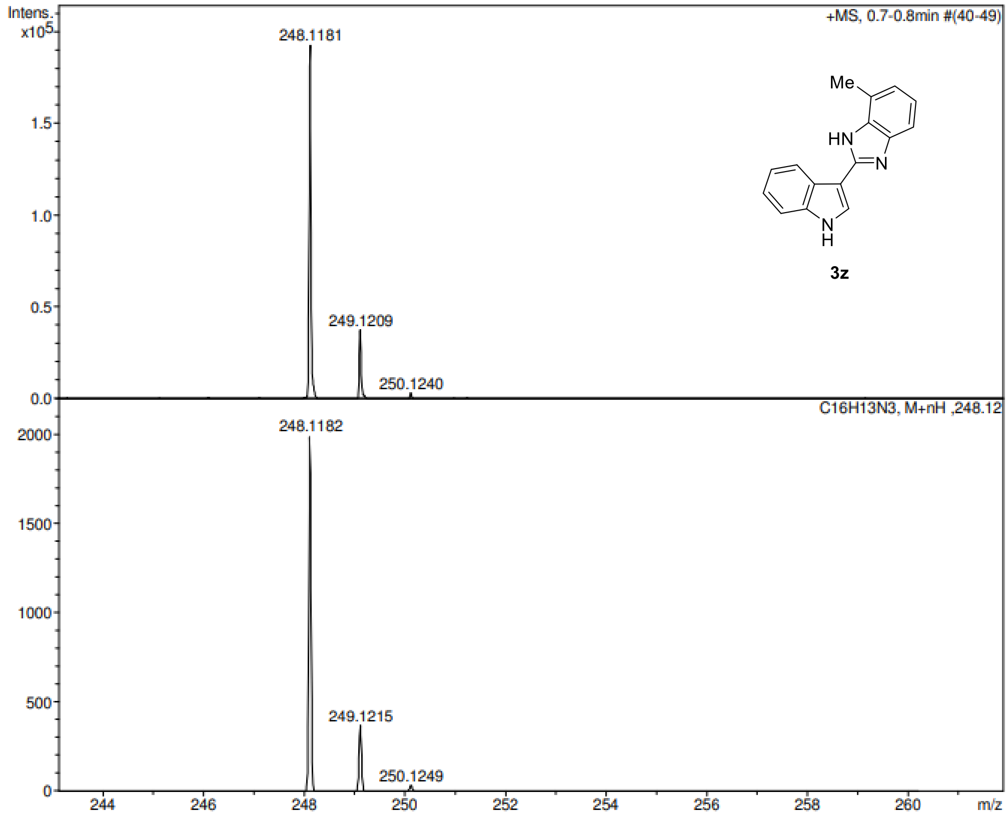
Intens.
x10^5
+MS, 0.7-0.8min #(40-49)
248.1181
249.1209
250.1240
Me
HN
N
N
H
3z
C16H13N3, M+nH ,248.12
248.1182
249.1215
250.1249
m/z

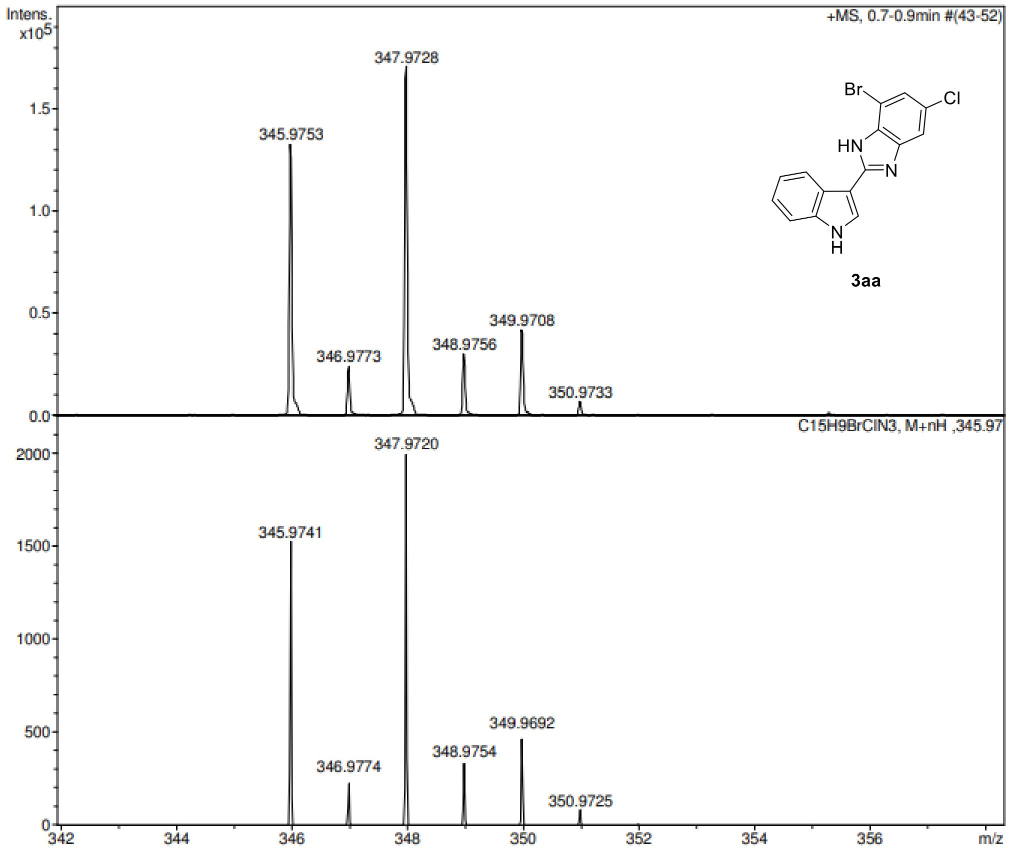

Intens.
x10^5
+MS, 0.7-0.9min #(43-52)
347.9728
345.9753
346.9773
348.9756
349.9708
350.9733
Br
Cl
HN
N
N
H
3aa
C15H9BrClN3, M+nH ,345.97
347.9720
345.9741
346.9774
348.9754
349.9692
350.9725
m/z

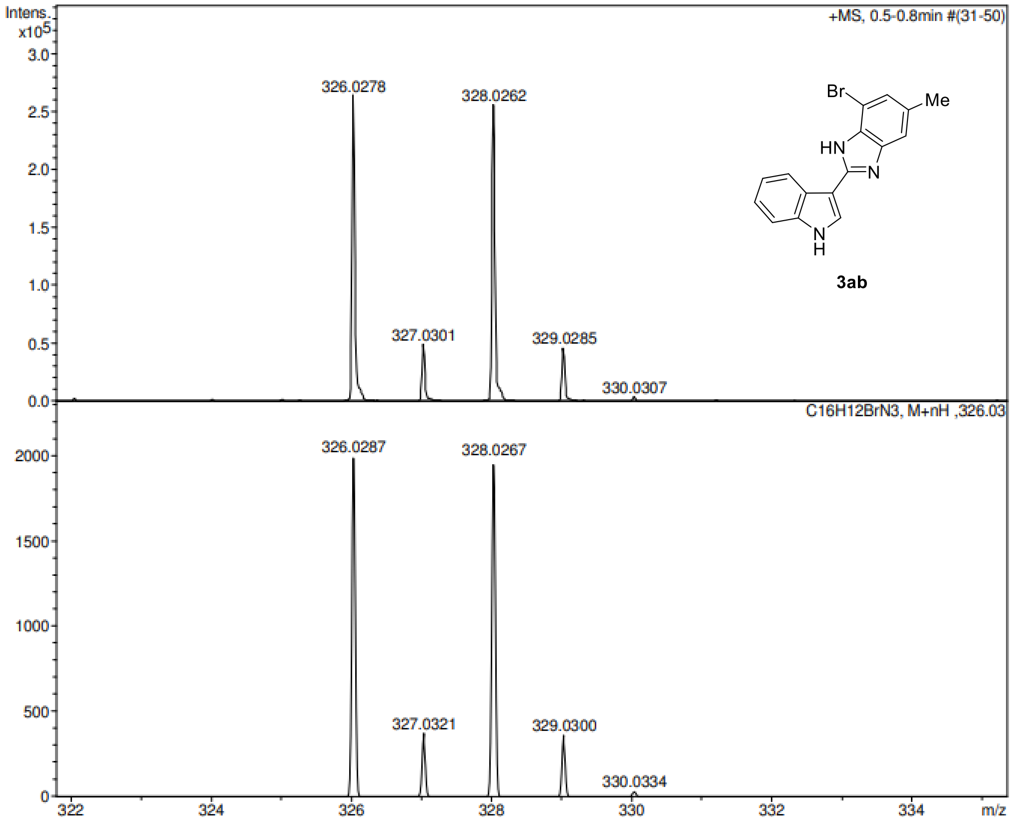

Intens.
x10^5
+MS, 0.5-0.8min #(31-50)
326.0278
328.0262
327.0301
329.0285
330.0307
Br
Me
HN
N
N
H
3ab
C16H12BrN3, M+nH ,326.03
326.0287
328.0267
327.0321
329.0300
330.0334
m/z

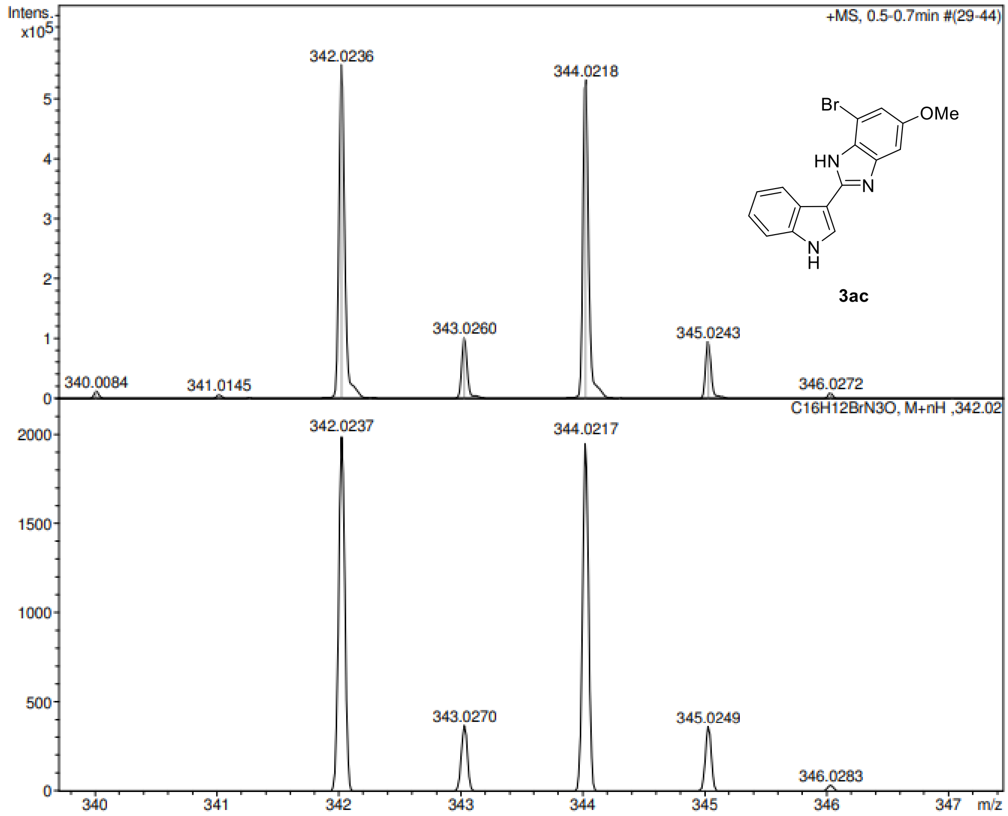

Intens. x10^5
+MS, 0.5-0.7min #(29-44)
342.0236
344.0218
343.0260
345.0243
340.0084
341.0145
346.0272
Br
OMe
HN
N
N
H
3ac
C16H12BrN3O, M+nH ,342.02
342.0237
344.0217
343.0270
345.0249
346.0283
m/z

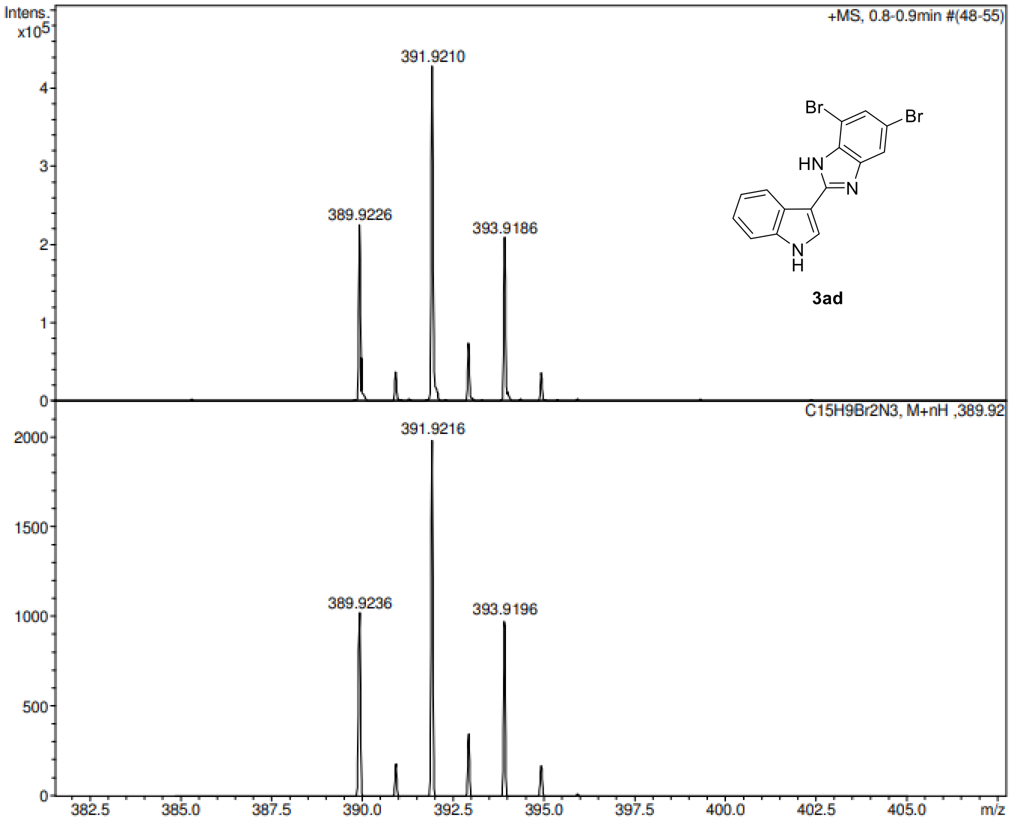

Intens. x10^5
+MS, 0.8-0.9min #(48-55)
391.9210
389.9226
393.9186
Br
Br
HN
N
N
H
3ad
C15H9Br2N3, M+nH ,389.92
391.9216
389.9236
393.9196
m/z

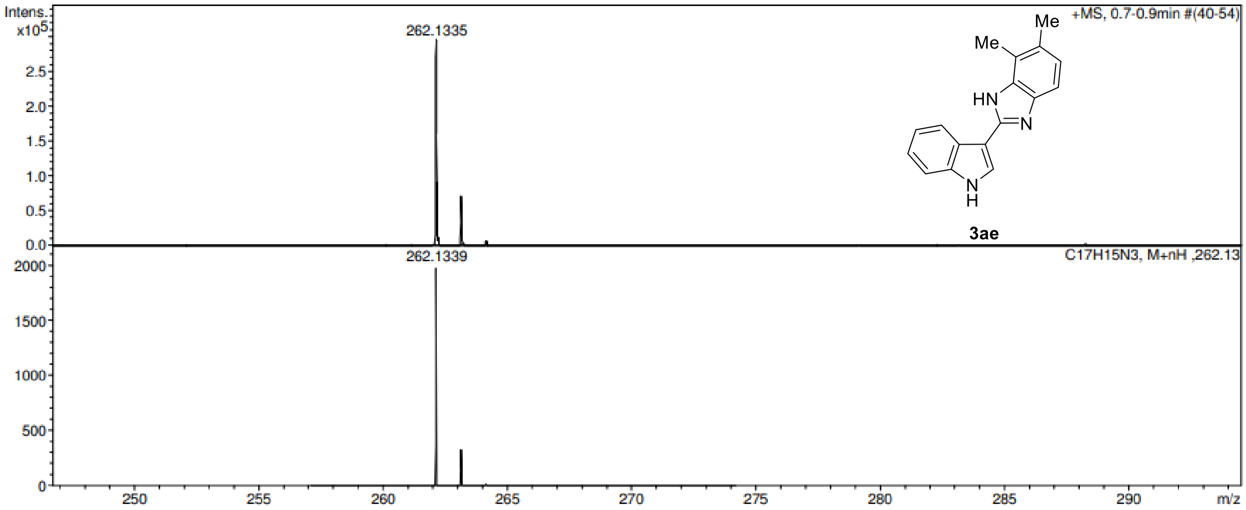
Intens.
x10^5
262.1335
+MS, 0.7-0.9min #(40-54)
Me
Me
HN
N
N
H
3ae
262.1339
C17H15N3, M+nH ,262.13
m/z

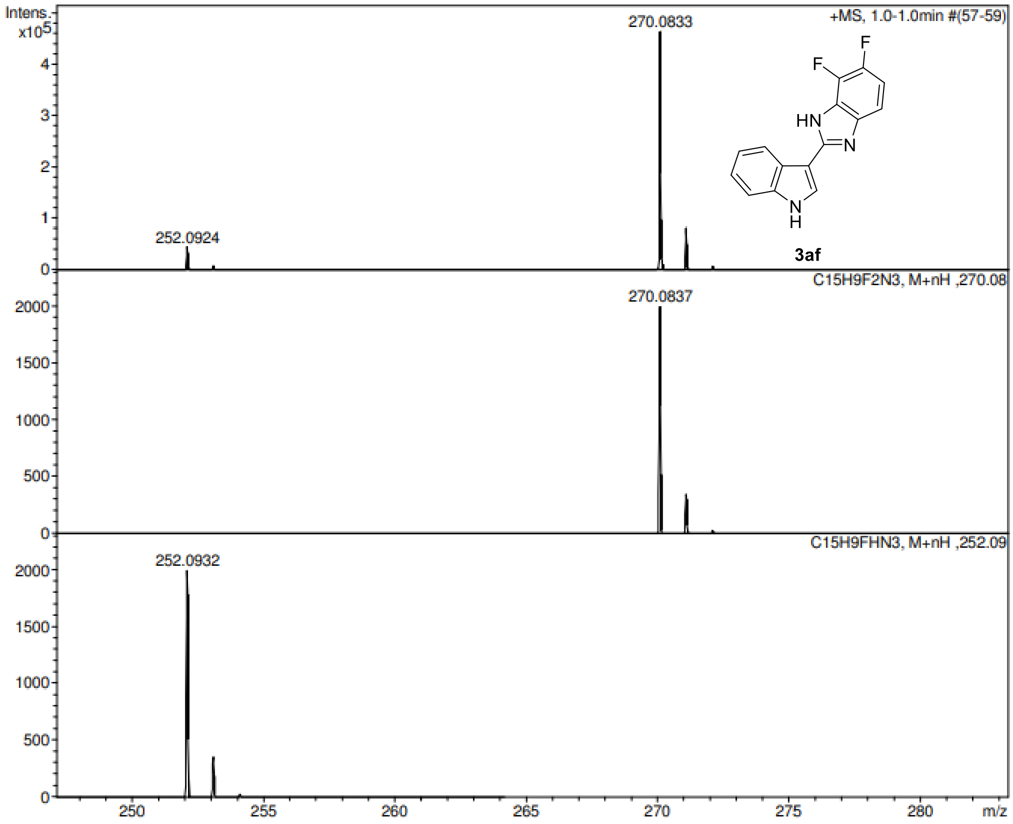
Intens.
x10^5
270.0833
+MS, 1.0-1.0min #(57-59)
F
F
HN
N
N
H
252.0924
3af
C15H9F2N3, M+nH ,270.08
270.0837
C15H9FHN3, M+nH ,252.09
252.0932
m/z

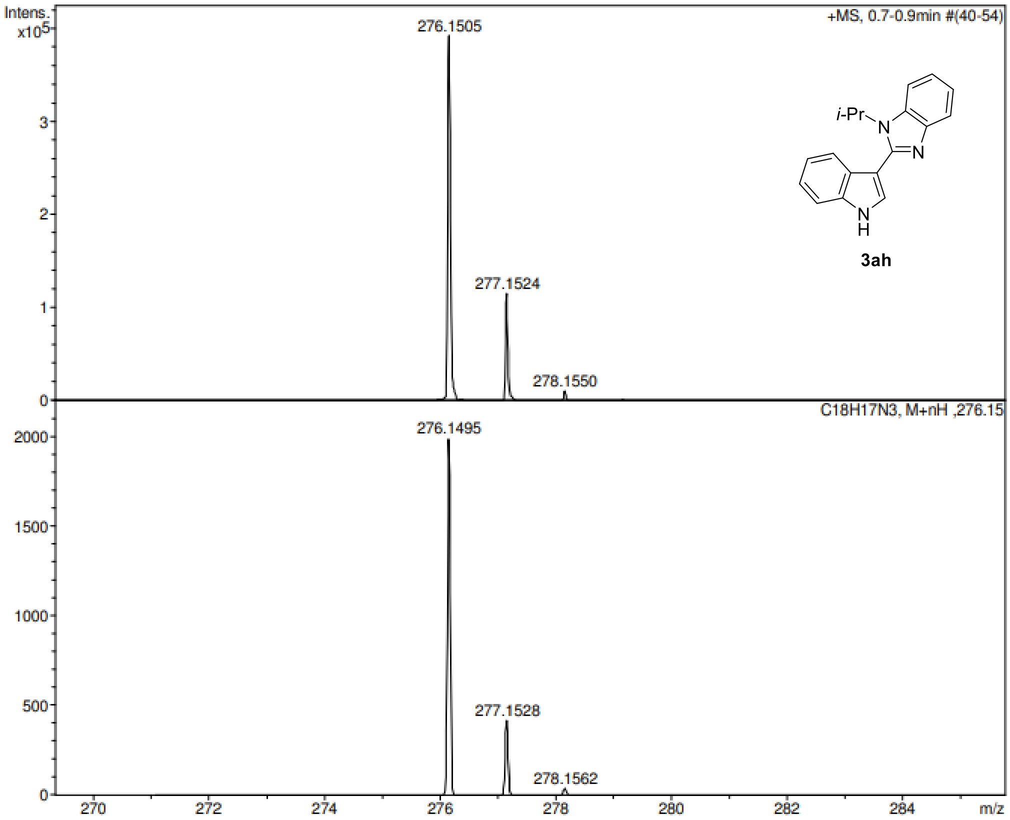
Intens.
x10^5
+MS, 0.7-0.9min #(40-54)
276.1505
277.1524
278.1550
C18H17N3, M+nH ,276.15
276.1495
277.1528
278.1562
m/z
i-Pr
N
H
3ah

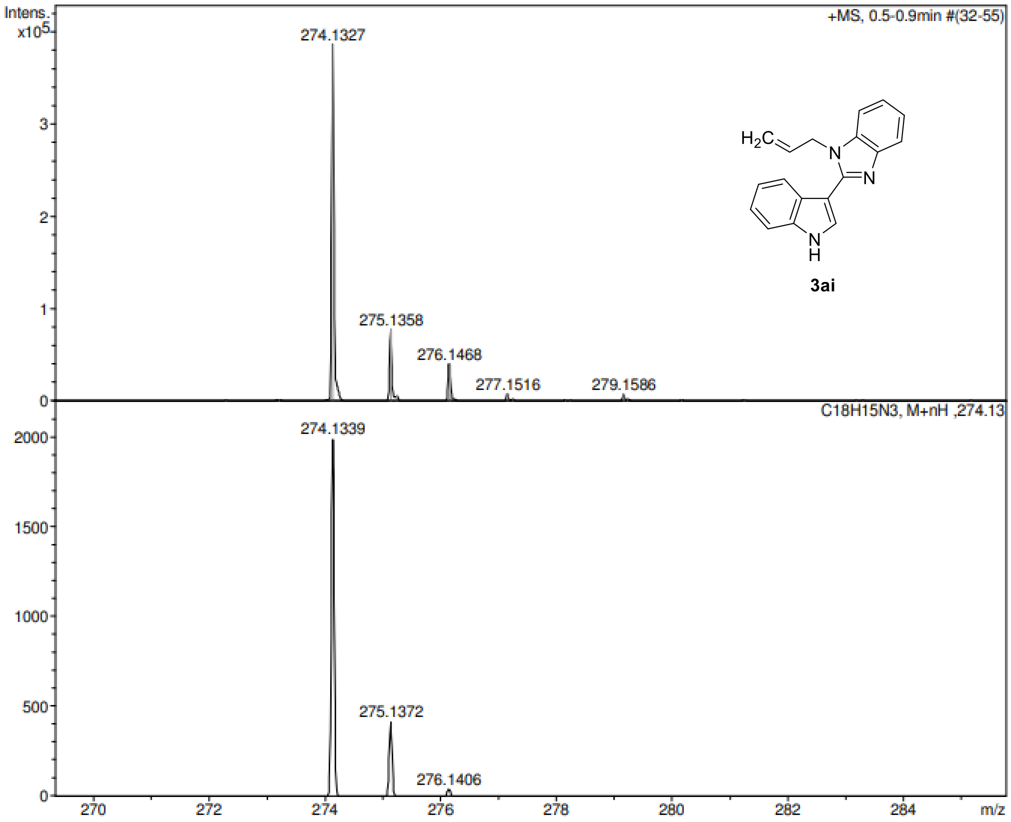
Intens.
x10^5
+MS, 0.5-0.9min #(32-55)
274.1327
275.1358
276.1468
277.1516
279.1586
C18H15N3, M+nH ,274.13
274.1339
275.1372
276.1406
m/z
H2C
N
H
3ai

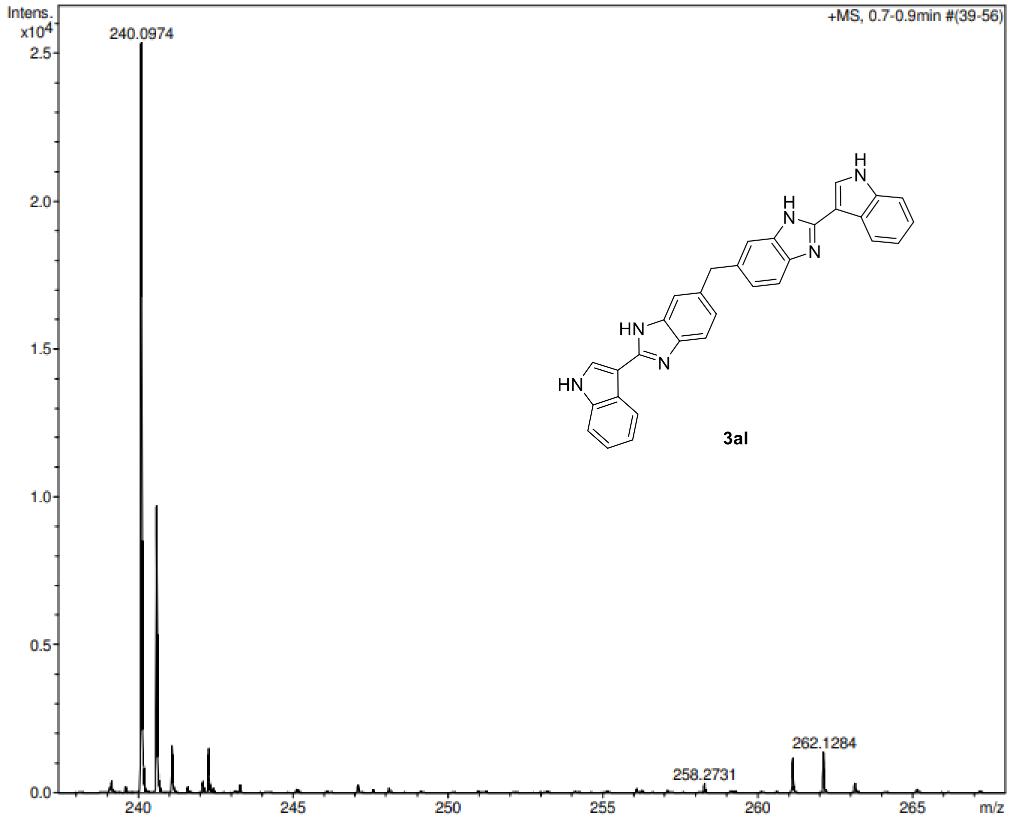
Intens.
x10^4
+MS, 0.7-0.9min #(39-56)
240.0974
258.2731
262.1284
m/z

3al

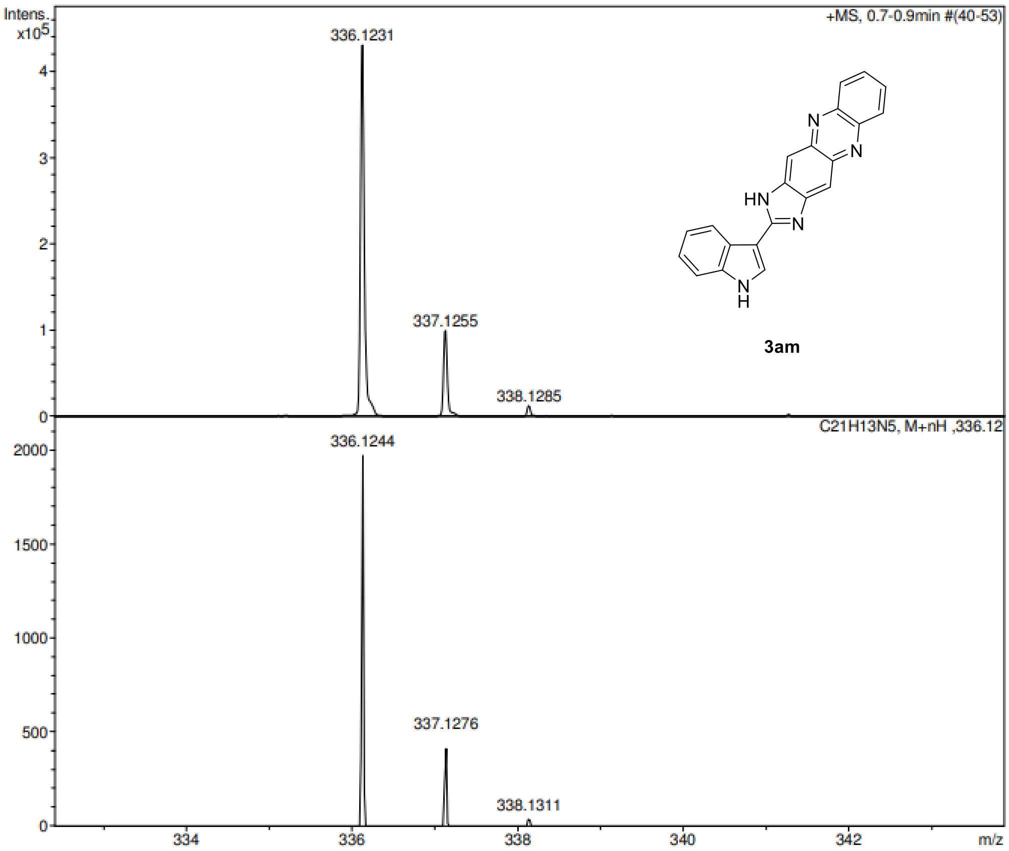
Intens.
x10^5
+MS, 0.7-0.9min #(40-53)
336.1231
337.1255
338.1285
C21H13N5, M+nH ,336.12
336.1244
337.1276
338.1311
m/z

3am

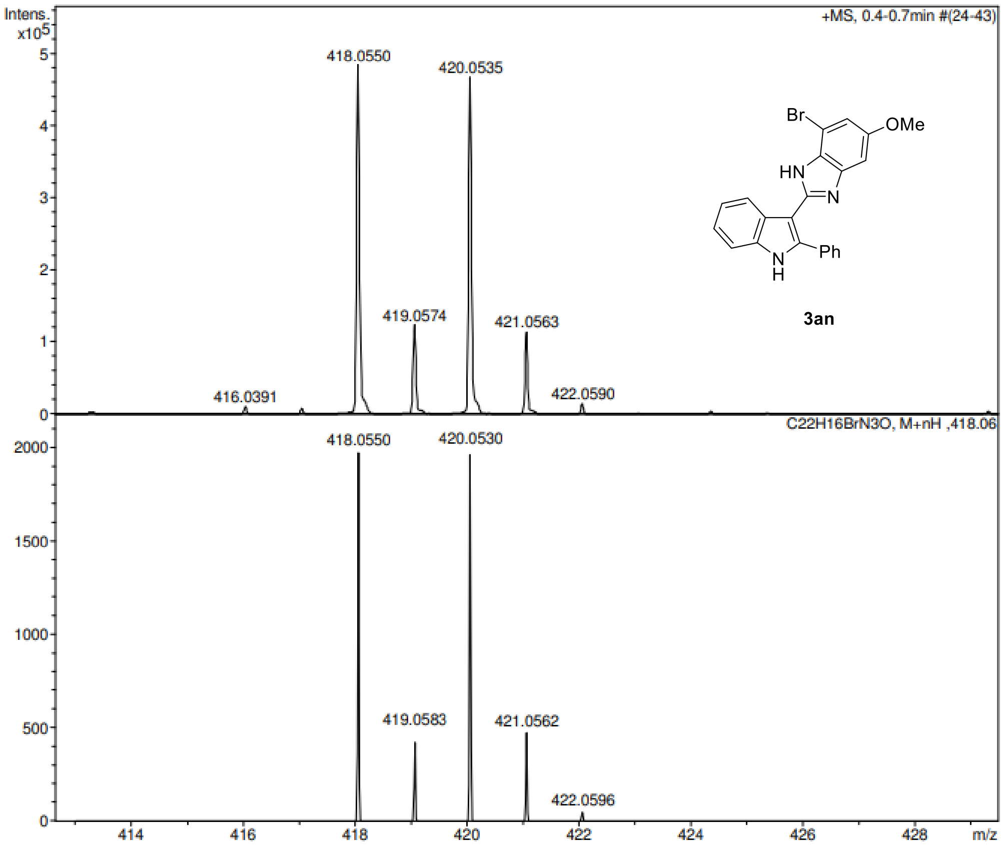
Intens.
x10^5
+MS, 0.4-0.7min #(24-43)
418.0550
420.0535
419.0574
421.0563
416.0391
422.0590
Br
OMe
HN
N
Ph
N
H
3an
C22H16BrN3O, M+nH ,418.06
418.0550
420.0530
419.0583
421.0562
422.0596
m/z

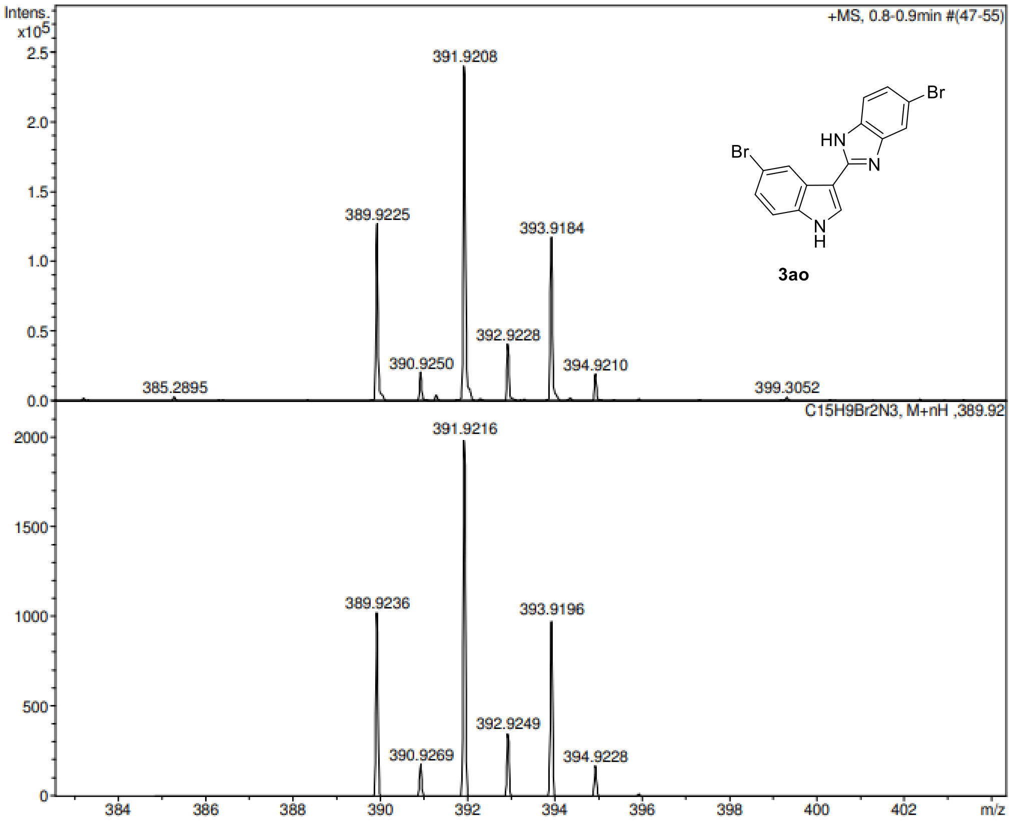
Intens.
x10^5
+MS, 0.8-0.9min #(47-55)
391.9208
389.9225
393.9184
392.9228
390.9250
394.9210
385.2895
399.3052
Br
HN
N
Br
N
H
3ao
C15H9Br2N3, M+nH ,389.92
391.9216
389.9236
393.9196
392.9249
390.9269
394.9228
m/z

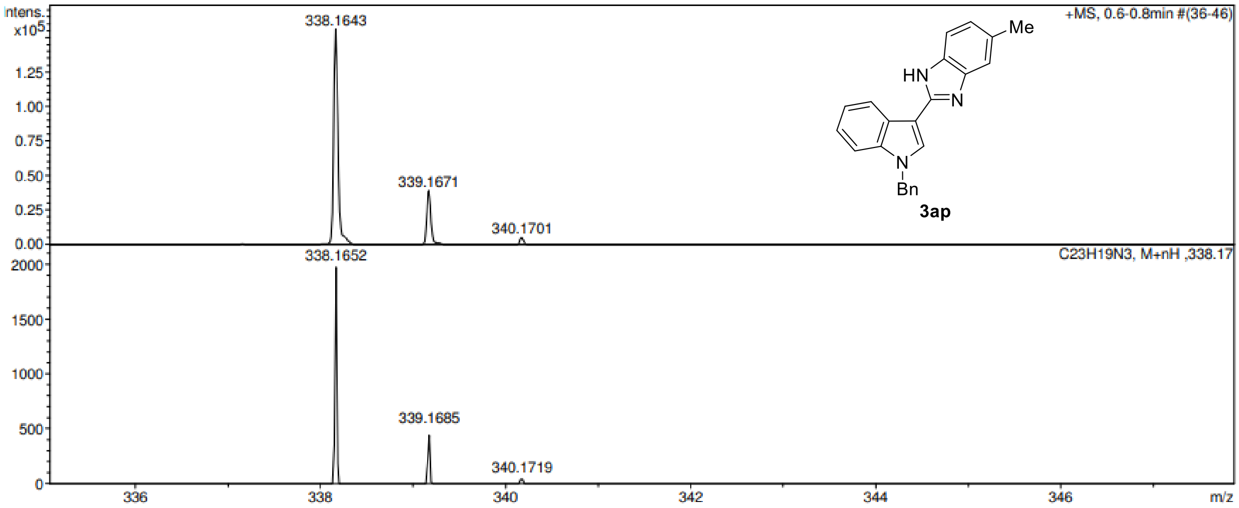

Intens.
x10^5
+MS, 0.6-0.8min #(36-46)
338.1643
339.1671
340.1701
Me
HN
N
N
Bn
3ap
C23H19N3, M+nH ,338.17
338.1652
339.1685
340.1719
m/z

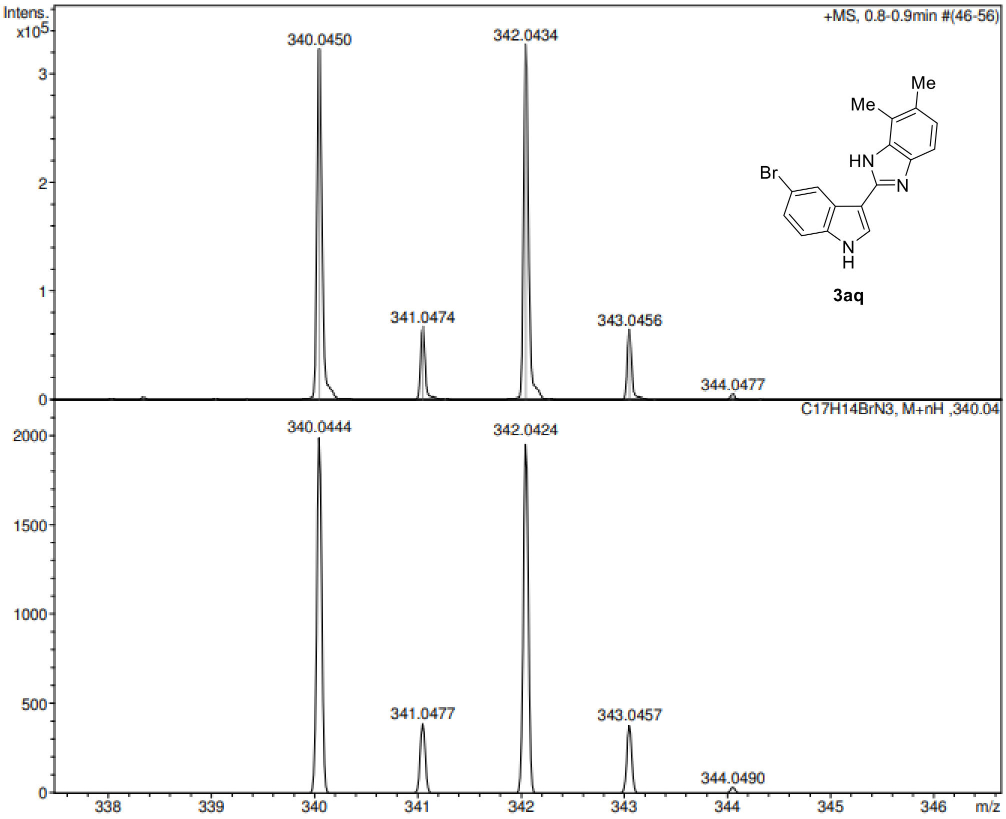

Intens.
x10^5
+MS, 0.8-0.9min #(46-56)
340.0450
342.0434
341.0474
343.0456
344.0477
Me
Me
HN
N
Br
N
H
3aq
C17H14BrN3, M+nH ,340.04
340.0444
342.0424
341.0477
343.0457
344.0490
m/z

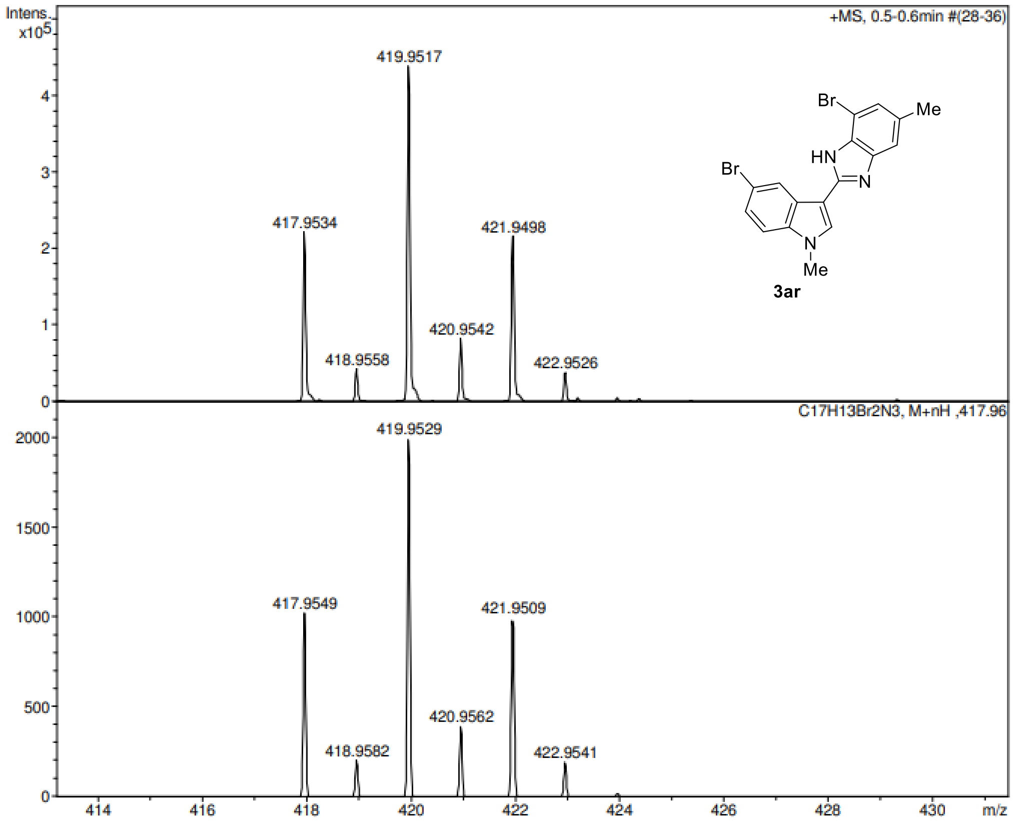

Intens.
x10^5
+MS, 0.5-0.6min #(28-36)
419.9517
417.9534
421.9498
420.9542
418.9558
422.9526
Br
Me
HN
N
Br
N
Me
3ar
C17H13Br2N3, M+nH ,417.96
419.9529
417.9549
421.9509
420.9562
418.9582
422.9541
m/z

X-ray crystallography data

Table S2: Experimental details for 2-(1*H*-indol-3-yl)-6,7-dimethyl-1*H*-benzo[*d*]imidazole (**3ae**, CCDC 2293846).

Crystal data	
Chemical formula	$C_{17}H_{15}N_3 \cdot C_4H_8O_2$
M_r	349.42
Crystal system, space group	Monoclinic, $P2_1/c$
Temperature (K)	295
a, *b*, *c* (Å)	12.440 (2), 19.105 (3), 17.182 (4)
β (°)	109.69 (2)
V ($Å^3$)	3844.7 (13)
Z	8
Radiation type	Mo $K\alpha$
μ (mm^{-1})	0.08
Crystal size (mm)	0.3 × 0.25 × 0.12
Data collection	
Diffractometer	New Xcalibur, Ruby
Absorption correction	Multi-scan<br>*CrysAlis PRO* 1.171.42.74a (Rigaku Oxford Diffraction, 2022) Empirical absorption correction using spherical harmonics, implemented in SCALE3 ABSPACK scaling algorithm.
T_{min}, T_{max}	0.545, 1.000
No. of measured, independent and observed [$I > 2\sigma(I)$] reflections	24753, 9266, 4384
R_{int}	0.042
$(\sin \theta/\lambda)_{max}$ ($Å^{-1}$)	0.691
Refinement	
$R[F^2 > 2\sigma(F^2)]$, $wR(F^2)$, S	0.086, 0.297, 1.02
No. of reflections	9266
No. of parameters	550
No. of restraints	140
H-atom treatment	H atoms treated by a mixture of independent and constrained refinement
$\Delta\rho_{max}$, $\Delta\rho_{min}$ (e $Å^{-3}$)	0.54, −0.37

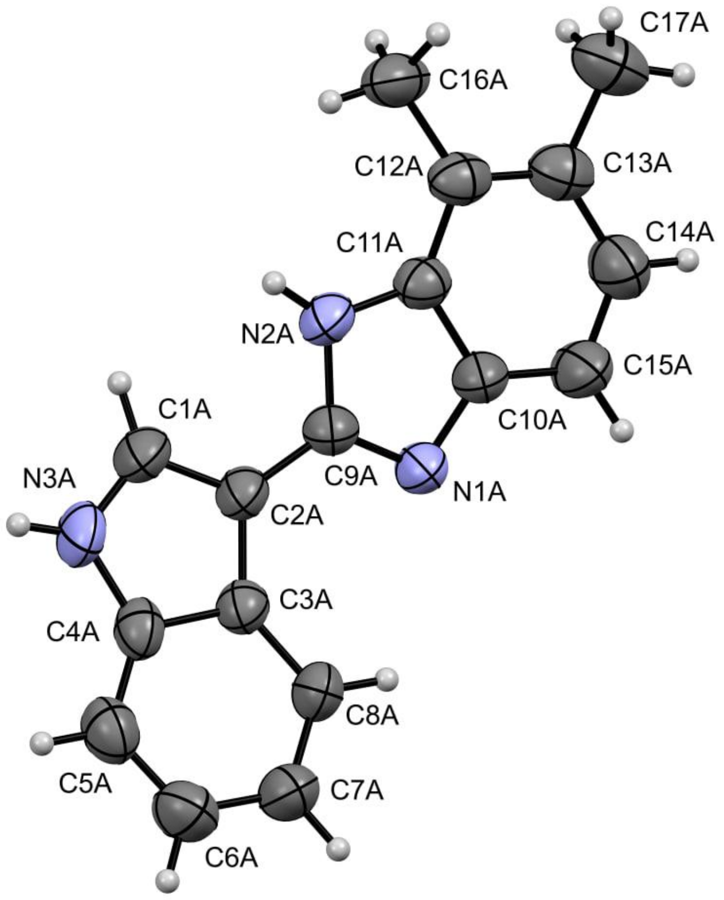

Figure S2. Structure of the 2-(1*H*-indol-3-yl)-6,7-dimethyl-1*H*-benzo[*d*]imidazole (**3ae**, CCDC 2293846) according to the X-ray diffraction data; non-hydrogen atoms are shown as thermal vibration ellipsoids with a probability of 50%.
